# Acknowledgment to the Reviewers of *International Journal of Molecular Sciences* in 2022

**DOI:** 10.3390/ijms24032105

**Published:** 2023-01-20

**Authors:** IJMS Editorial Office

**Affiliations:** MDPI AG, St. Alban-Anlage 66, 4052 Basel, Switzerland

High-quality academic publishing is built on rigorous peer review. *International Journal of Molecular Sciences* was able to uphold its high standards for published papers due to the outstanding efforts of our reviewers. Thanks to the efforts of our reviewers in 2022, the median time to first decision was 16 days and the median time to publication was 35 days. Regardless of whether the articles they examined were ultimately published, the editors would like to express their appreciation and thank the following reviewers for the time and dedication that they have shown *IJMS*:
A. DorenKun TianA. G. SobolevaKun WangA. GanesanKun YangA. K. M. Mahmudul HuqueKundi YangA. M. DunaevKundlik GadhaveA. N. M. Mamun-Or- RashidKung-Woo NamA. N. MisraKunhao YuA. Ruiz-SauriKunihiko NakaiA. S. LukatkinKunihiro TsuchidaA. Stephen HashmiKunihiro TsukasakiA. V. ErkinKunihiro YamaokaA. V. RakovKunimichi NiibeAamir RainaKunka Mohanram RamkumarAaron AlfordKunle OkaiyetoAaron James TaborKun-Lin TsaiAaron M. NeimanKun-Ling TsaiAaron MendezKun-Yi HsinAaron MuthKun-Yun YehAaron NilsenKuo-Ching MeiAarti BainsKuo-Liang ChiangAarti GuptaKurata MoritoAarti NagayachKuroda YosukeAarti SharmaKürşat ErAarushi SharmaKurt A. JellingerAasif HelalKvӗta KalíkováAayudh DasKwabena Frimpong Manso OpuniAbani Kanta NaikKwaku Dad Abu-BonsrahAbayomi OkeKwang Poo ChangAbazar GhorbaniKwang Suk LimAbbas A. Abbaszadeh ShahriKwang Youl LeeAbbas KhanKwang-Hoon ChunAbbas MalandishKwang-Jin ChoAbbas RahdarKwang-Zin LeeAbbe Maleyki Mhd JalilKwanuk LeeAbbirami ElangovanKweon YuAbd El-Latif HeshamKwok-Kuen CheungAbdallah Tageldein MansourKwon-Ho HongAbdel AissatKy Young ParkAbdel Ali BelaidiKyeong-Man KimAbdel Majid A. AdamKyla D. OmilusikAbdelali HannoufaKyle BurghardtAbdelaziz AmraniKyle PoulsenAbdelfattah El MoussaouiKylie D. RockAbdelfattah M. SelimKylie J. WattsAbdelhabib SemlaliKym LowryAbdelilah BeljebbarKyohei FurukawaAbdel-Majid KhatibKyohei KinAbdessamad El KaoutariKyoji MoritaAbdolmajid AlipourKyojiro N. IkedaAbdolrahman KhezriKyoko Tsukiyama-KoharaAbdolrahman Shams NateriKyong-Ah YoonAbdolreza HosseindoustKyoung Sang ChoAbdorreza Naser-MoghadasiKyoung-Seok RyuAbdou Azaque ZouréKyriaki KyriakidouAbdul Alim Al-BariKyriaki SidiropoulouAbdul Bari Bari ShahKyu LimAbdul BasitKyu Yeon HanAbdul HameedKyubong JoAbdul HannanKyuho KangAbdul JalalKyung Jong WonAbdul Kader JailaniKyung Pyo KangAbdul RazzaqKyung Taek RimAbdul RehmanKyung-A HwangAbdul Rehman PhullKyungha LeeAbdul RohmanKyung-Mee ParkAbdul SalamKyung-Min KimAbdulfatah A. YusufKyung-Mok ParkAbdulhameed Al-GhabkariKyung-Sook ChoiAbdulkarim HasanKyung-Wan BaekAbdulkarim Jafar KarimKyu-Sung KimAbdullah AlotaibiL. N. StepanovaAbdullah AssiriL. RuffiniAbdullah Md. SheikhLa Hoang AnhAbdullah TariqueLabrini V. AthanasiouAbdullahi AlhassanLachlan A. JollyAbdus SamadLacramioara OchiuzAbdus SoburLacramioara PopaAbeda JamadarLada E. PetrovskayaAbedalrhman AlkhateebLadislav DokládalAbeer ElkadyLaetitia AymericAbegaz Tizazu AndrgieLaetitia ChauviereAbel BronkhorstLaibao ZhangAbel Po-Hao HuangLaila GasmiAbha JainLaila ZikoAbhay K PandeyLaima ČesonienėAbhijeet JoshiLajos NagyAbhijit ChakrabortyLakmini SenavirathnaAbhijit ChatterjeeLakshmaiah SreeramaAbhijit KaleLakshmanakumar KinthadaAbhijit SarkarLakshmikanta MondalAbhilash PadavannilLalit BatarAbhilasha Kandahalli Venkataranga NayakaLalit SehgalAbhilasha SinhaLalith PereraAbhimanyu SarkarLamar O. MairAbhinav JainLambertus Van Den HeuvelAbhinav JoshiLamees HegazyAbhinav KaushikLamiaa M. A. AliAbhinava MishraLan ChenAbhinaw KumarLan Wei-LapierreAbhinay ThakurLan ZhangAbhirup DasLane L. ClarkeAbhirup PatraLang RaoAbhisek BhattacharyaLanglois BenoîtAbhishek ChandraLannie O'KeefeAbhishek ChauhanLantao GouAbhishek DeyLanuza Alaby Pinheiro FaccioliAbhishek GuptaLara BaticicAbhishek KumarLara ConsoleAbhishek Kumar SinghLara CostantiniAbhishek MishraLara K. AbramowitzAbhishek TyagiLara MassaiAbhishesh MehataLara PerrymanAbid OueslatiLara Saftić MartinovićAbid UllahLara Valiño-RivasAbigail C. LayLarbi RhaziAbigail SuwalaLarisa AnghelAbijeet MehtaLarisa LitvinovaAbimbola E. OluwalanaLarisa RyskalinAbitha SukumaranLarisa TsarkovaAbolfazl NarmaniLarisa VolovaAboozar MonavarfeshaniLarissa BalabanovaAbouzar NajafiLarry CroftAbraham Escobedo-MoratillaLarry J. GrabauAbraham O. SamsonLarry L BartonAbraham Wall MedranoLars AagaardAbril GijsbersLars NorgrenAbu HazafaLars NyAbu Hena Mostafa KamalLars Petter JordheimAbu Imran BabaLars PlateAbul Kalam AzadLars SöderströmAca JovanovićLars VerschurenAchille AnselmoLars-Peter KamolzAchira RoyLassaâd BelbahriAchuthamangalam B. MadhankumarLasse LindahlAchyut DahalLassina BarroAda PopoloLászló AlmásyAdalberto VieyraLászló BakacsyAdam B. EdwardsLaszlo Barna IantovicsAdam BialesLaszlo BorosAdam BrysiewiczLaszlo BudayAdam C SoloffLászló BudayAdam CudowskiLászló CsambalikAdam DobrowolskiLászló DrahosAdam FramptonLászló DuxAdam HogendorfLászló EgyedÁdám HorváthLaszlo FesusAdam J. KriegLászló HegedűsAdam J. KrysztofiakLászló HéjaAdam J. LukaszewskiLászló JicsinszkyAdam Johan BergrenLaszlo KalmanÁdám JuhászLászló KölesAdam KawalekLászló Kozma-BognárAdam KernLaszlo ProkaiAdam KlocperkLaszlo SmellerAdam KowalczykLászló SzalayÁdám KunLaszlo SzilagyiAdam LesnerLászló SzilágyiAdam LiwoLászló SzilákAdam M. HawkridgeLaszlo TretterAdam OkorskiLászló VécseiAdam P. LightfootLászló ZimányiAdam PomorskiLata SinghAdam PyzikLatifa BouissaneAdam ReichLatifa Rbah-VidalAdam RomanLaura AcquasalienteAdam SieradzanLaura AlanizAdam SikoraLaura Ancuţa PopAdam Thean Chor LeowLaura AndolfiAdam W. WhisnantLaura Aracely Contreras-AnguloAdam ZmysłowskiLaura AudìAdarsh K. GuptaLaura BergantiniAddolorata CorradoLaura BonfiliAdebowale OgunjirinLaura BonsiAdedolapo OjoawoLaura BrownAdeel MalikLaura C. Zanetti-DominguesAdel Al-GheethiLaura CarrassaAdel ElmoselhiLaura CastellettiAdela NacerLaura CerqueiraAdela PinteaLaura CiapponiAdelaide MirandaLaura CivieroAdele ChimentoLaura CotoiAdele PapettiLaura Cristina CeafalanAdele StewartLaura D'AlfonsoAdelina F. DumitrașLaura De LellisAdelina VladLaura E. CarsonAdeline DivouxLaura ElomaaAdenilda Honorio-FrançaLaura FananiAdeniyi Michael AdebesinLaura FavettaAdesh SainiLaura Fernández-SánchezAdewale O. FadakaLaura GambariAdhimoolam KarthikeyanLaura GattiAdhityo WicaksonoLaura Gheucă SolovăstruAdi PinkasLaura GiacomelliAdil HussainLaura GianottiAdil MarjaouiLaura GutiérrezAdina NegreaLaura HanoldAdina SantanaLaura J. BlairAdinolfi ElenaLaura J. EllettAditi BhargavaLaura K. S. ParnellAditi JainLaura KasmanAditi PrabhakarLaura KrestyAditi SatputeLaura ListenbergerAditi SwarupLaura LocatelliAditya GanjuLaura LossiAditya S. YerramilliLaura M. G. MeemsAditya SarodeLaura M. HackAditya SharmaLaura MaziluAdnan AkhterLaura Medina-PucheAdnan AynaLaura MenoAdnan IsmaielLaura MercurioAdnan KhanLaura Miller ConradAdnan MustafaLaura MoralesAdolfo Aracil-MarcoLaura Mora-LópezAdolfo B. PomaLaura Moreno-MartínezAdolfo Francesco AttiliLaura Muinelo-RomayAdolfo Maria TambellaLaura N. BorodinskyAdolfo PomaLaura N. BurgaAdolfo Rivero-MüllerLaura ParmaAdrià ArboixLaura PerimanAdrià Gil-MestresLaura PolitoAdrian AntosikLaura RagonaAdrian ArendowskiLaura RestaniAdrian ArrietaLaura SabatinoAdrian Bogdan ȚiguLaura SalvadoriAdrian C. BrennanLaura Sánchez-MuñozAdrian CãtineanLaura Sterian Terian WardAdrian ChlandaLaura TiberioAdrian CumminsLaura TrandafirAdrian Florin GalLaura V. KlotzAdrián GarridoLaura VicenteAdrian KellerLaura VitielloAdrian MorenoLaura ZsigmondAdrian NeaguLaura-Cristina RusuAdrian OoLaure YatimeAdrian ȘpacLaurel SillerudAdrian SturzaLaureline BerthelotAdrian TeacăLauréline RogerAdrian Velázquez-CampoyLauren Des MarteauxAdrian ZającLauren DoumaAdriana BasileLauren K. WarehamAdriana Bos-MikichLauren RichardsonAdriana Burlea-SchiopoiuLaurence Bresson-BepoldinAdriana Casao GascónLaurence Camoin-JauAdriana Cunha NevesLaurence DinanAdriana De Jesus Inácio BelasLaurence LagneauxAdriana DerejkoLaurence Panicot-DuboisAdriana DussoLaurent CombettesAdriana E. MieleLaurent DemizieuxAdriana FilipLaurent DufosséAdriana GrigorașLaurent M SachsAdriana HanganLaurent MetzingerAdriana IspasLaurent MullerAdriana M. CalvoLaurent RiouAdriana MikaLaurentia Nicoleta GalesAdriana MonroyLaurentiu PirteaAdriana MorarLaurentiu RusenAdriana PlesaLauria MassimilianoAdriana R. RodriguesLauro Velazquez-SalinasAdriana TrifanLaurye Van MaeleAdriana VinhasLavinia Cosmina ArdeleanAdriana Vulpoi LazarLavinia Florina CălinoiuAdrianne BendichLavinia RutaAdriano AraujoLawrance ChandraAdriano DuattiLawrence A. VernettiAdriano Jimenez-EscrigLawrence E. GoldfingerAdriano MollicaLawrence H LashAdrie VerhoevenLawrence M WolfAdriele Prina-MelloLawrence MabasaAdrien ThurotteLawrence RothblumAdriene Ribeiro LimaLawrence SowersAdrienn MarkovicsLaxman MainaliAdrienne K. SalmLaxminarayanan KrishnanAdugna WoldesemayatLayla Al-NakkashAdwait GodboleLayla PiresAekkhaluck IntharuksaLe Phuong NguyenAe-Kyung YiLe XuAenim PaeLe YuAeyal RazLea D. BennettAfjalus SirajLea HošnjakAfonso Rocha Martins AlmeidaLea LojkovaAfroditi KrokidaLéa LucianiAfsha TabassumLea ScherschinskiAfshan Fathima NawasLea SpindlerAfsheen RazaLeandro De León GuerraAftab AlamLeandro Exequiel LuceroAftab BashirLeandro SantiagoAfua Adjeiwaa MensahLeandro SastreAfzal HussainLebaka Veeranjaneya A. ReddyAfzal KhanLechuang ChenAfzal MisraniLeda GuzmánAgata CampisiLee Ann Laurent-ApplegateAgata FabiszewskaLee Bulla, JrAgata Faron-GóreckaLee J. MartinAgata GabryelskaLee M. GravesAgata GadaletaLee SukhyangAgata GozdzLee Wei LimAgata GóźdźLee-Ann H. AllenAgata Grazia D'AmicoLeela Raghava Jaidev ChakkaAgata Jabłońska-TrypućLeena KadamAgata KaczmarekLei BaoAgata LeszczukLei DingAgata MichalakLei FangAgata NawrockaLei GuoAgata SoluchLei HeAgata StanekLei HuangAgata SzulcLei LiAgata TyczewskaLei NieAgata WawrzyńczakLei WanAgata ZmijewskaLei WangAgata ZnamirowskaLei XiAggelos AvramopoulosLei YanAgnė ŠulčiūtėLei ZhangÁgnes CséplőLei ZhaoAgnes KlarLeif KariÁgnes KlusóczkiLeif-Hendrik BoldtAgnes PurwidyantriLeigh PlantAgnès RötigLeila AllahqoliAgnes SchröderLeila JahangiriAgnes SzappanosLeila Marek-CrnjacAgnes SzepesiLeila VieiraAgnes TelbiszLeila YoussefianÁgnes TelbiszLeili Saeednejad ZanjaniAgnese Chiara PippioneLeilson RibeiroAgnese De MarioLeire PedrosaAgnese SecondoLejla MedzikovicAgneta SimionescuLela BuckinghamAgnieszka A. GoliczLela VukovićAgnieszka A. KaczorLena StrömAgnieszka A. PilarskaLena StudtAgnieszka B. BialkowskaLenaic LartigueAgnieszka BanasLenard FarczadiAgnieszka BęśLene H. PetersenAgnieszka BiałekLenka KlčováAgnieszka BossowskaLenka StixovaAgnieszka BronowskaLeny JoseAgnieszka DomkaLeo A. Van Der PolAgnieszka DrozdzikLeo MassariAgnieszka E. DacaLeo VeenmanAgnieszka Ewa StępieńLeon Garland ColemanAgnieszka FiszerLeon HigleyAgnieszka FogelLeon SeifertAgnieszka GalantyLeon StraubAgnieszka GerkowiczLeona GilbertAgnieszka GrzegorczykLeona Leisova-SvobodovaAgnieszka I. Piotrowicz-CieślakLeonard AtanaseAgnieszka Jagiello-GruszfeldLeonard B. MaggiAgnieszka Jagiełło-GruszfeldLeonard Barnabas EbinezerAgnieszka JeleńLeonardo Alexandre Peyré-TartarugaAgnieszka KorgaLeonardo BascoAgnieszka KosowskaLeonardo BencivengaAgnieszka KrawczenkoLeonardo BrunettiAgnieszka KrupaLeonardo Catalano-IniestaAgnieszka Kubicka-TrzskaLeonardo CesanelliAgnieszka KuźniarLeonardo Giordano PaternoAgnieszka KwiatekLeonardo L. FrutteroAgnieszka KyziołLeonardo Lo PrestiAgnieszka Łapczuk-KrygierLeonardo OrnellaAgnieszka LatosinskaLeonardo Pessoa FélixAgnieszka Lecka-AmbroziakLeonardo PuppulinAgnieszka LejmanLeonardo RestaAgnieszka Łupicka-SłowikLeonardo VelascoAgnieszka Magdalena RygielLeonel PereiraAgnieszka Marasek-CiolakowskaLeonhard MüllauerAgnieszka MatuszewskaLeonid A. KulikovAgnieszka MlynarskaLeonid GurevichAgnieszka MostowskaLeonid IonovAgnieszka MroczekLeonid V. KulikAgnieszka Münster-WandowskiLeonor Puchades-CarrascoAgnieszka NawrockaLeontina C. GurguAgnieszka NiedzielaLeopold EckhartAgnieszka Owczarczyk-SaczonekLeopoldo FornerAgnieszka PartykaLeopoldo StaianoAgnieszka Piastowska-CiesielskaLesław JuszczakAgnieszka PiegatLeslie CaronAgnieszka PilarskaLeslie Chavez-GalanAgnieszka PiwkowskaLeslimar Rios-ColonAgnieszka PłażekLeszek KarlińskiAgnieszka PotęgaLeszek KubiszAgnieszka RobaszkiewiczLeszek SzablewskiAgnieszka SkarzyńskaLeszek WanatAgnieszka SkowrońskaLeszek ZaraskaAgnieszka SliwinskaLeticia CalahorraAgnieszka SzewczykLeticia González-MayaAgnieszka SzymaszekLeticia Z. MoreiraAgnieszka Tomczyk-WarunekLetizia PalombaAgnieszka TomkowiakLevan ChkhartishviliAgnieszka Ulatowska-JarżaLevini MsimbiraAgnieszka WąsikLevon KhachigianAgnieszka Witalisz-SieprackaLewko BarbaraAgnieszka WnukLeyi WeiAgnieszka ZabłockaLeyland FraserAgnieszka ZagórskaLeyre MadariagaAgnieszka Zembron-LacnyLeyre ZubiriAgnishwar GirigoswamiLi ChaiAgostino BucaloLi FuAgostino CasapulloLi GuoAgostino OgnibeneLi JinAgre PaterneLi LiAgus LiandiLi LinAgustín Aibar-AlmazánLi LuoAgustín HernándezLi QibinAgustín L. ArceLi WeiAgustin ZapataLi XiaoAgustina De FrancescoLi YaoAgustino Martínez-AntonioLi ZhangAhad SiddiquiLi ZhouAhamad AlqudahLia H. CampbellAharon AzaguryLiad HindenAhmad Abd-El-AzizLiang ChenAhmad AlbaghdadiLiang GuoAhmad AlsayedLi-Ang LeeAhmad ArabkoohsarLiang LiuAhmad ArzaniLiang MaAhmad Bazli RamzLiang Oscar Qiang QiangAhmad G HegaziLiang SunAhmad IrfanLiang Ting LinAhmad Jawad SabirLiang WangAhmad Khusairi AzemiLiang XuAhmad MirzaLiang YeAhmad SaltiLiang YiAhmad Sohrabi KashaniLiang ZhangAhmed A SalehLiang ZhaoAhmed A. MohammedLiang ZhongAhmed Abdal DayemLiangcan HeAhmed AbdeenLiangchun LiAhmed Al‐KarmalawyLiangfa GeAhmed Al-KattanLiangjun XiaAhmed Al-SabiLian-Huan WeiAhmed BakillahLianmei HuAhmed Edan DhamadLianwu FuAhmed El-FiqiLíbia Zé-ZéAhmed ElkamhawyLibin ZhangAhmed ElmarakbyLibin ZhouAhmed F.M. El-MahdyLibo HeAhmed HajibLibo Yu-TaegerAhmed HassanLibo ZhangAhmed HieawyLibor KvitekAhmed HikalLiborija Lugović-MihićAhmed IbrahimLibusa SikurovaAhmed KamelLichao SunAhmed Kh. MeshaalLi-Ching ChangAhmed M. Abd-ElgawadLiching ChenAhmed M. Abu-DiefLicio CollavinAhmed M. El-ZohryLicun WuAhmed M. NaglahLidia BłaszczykAhmed M. YoussefLidia CantacorpsAhmed MahalLidia CicconeAhmed MedianiLidia GaffkeAhmed MehanyLidia GavicAhmed Mohammed Abu-Dief MohammedLídia GonçalvesAhmed MohsenLidia Hanna MarkiewiczAhmed NabawyLidia LarizzaAhmed Neamat-AllahLidia LimontaAhmed NoreldinLidia LomovatskayaAhmed RakibLidia Polkowska-KowalczykAhmed SalmanLidia Szulc-DąbrowskaAhmed ShaabanLidija BegovićAhmed TabbabiLidija GradišnikAhmet AyazLidija TodorovicAhmet Emre EskazanLieza OdendaalAhmet Emre YaprakLiezhao LiuAhmet KertmenLifang LiAhsan HameedLifat RahiAhsan JavedLi-Fen HuangAi DuLiga WuriAida De Arriba-ArnauLigang ChenAida MetoLige TongguAiden EblimitLigen LinAidin Bordbar-KhiabaniLihong LiAidin ForoutanLihong ZhaiAigerim SoltabayevaLihua ChenAihua WuLihua GanAikaterini PistikiLihua WangAike JeuckenLi-Hung ChenAiko NagayamaLijiang YangAile TammLijuan FengAilyn FadriquelaLilach SoreqAimee ColbathLilah Rahn-LeeAimee EgglerLili LiAimin ChangLili TanAimin JiangLili YangAimin LiuLili ZhouAi-Min WuLilia CroitorAinara SaralegiLilia GutierrezAinara Vallejo-IllarramendiLilian SchmidtAinhoa AlberroLiliana GarneataAinhoa Iglesias-AraLiliana KiczakAirat KayumovLiliana MamminoAisha FarhanaLiliana Mititelu TartauAisha SyedaLiliana Ostopovici-HalipAishwarya GokuldassLiliane N. TandziAishwarya GurumurthyLiliang JinAishwarya IyerLilianna ChecinskaAistė BulavaitėLilianne BeolaAiti VizziniLiliya FrolovaAitor RementeriaLiliya VasilevaAiva PlotnieceLilla TuriákAjay DixitLilli WinterAjay KesharwaniLily ArruéAjay KumarLimin WuAjay OblaLiming YangAjay PalLimor BaruchAjay S. MathuruLin HuangAjay SharmaLin JiangAjay Suresh AkhadeLin LiAjay Vikram SinghLin QiuAjaz A. LoneLin SangAjaz AhmadLin SongAjaz AhmedLin WangAjeet SinghLin XiAjinkya KawaleLin ZhangAjit GhoshLin ZhaoAjit RayLina BaranauskieneAjit RoyLina BernaolaAjit SinghLina Bezdetnaya-BolotineAjmal KhanLinawati SutrisnoAjmer Singh GrewalLinbo ZhaoAkanksha SeghalLinchong SunAkanksha SinghLinda BíróAkanksha ThawaniLinda BonenAkansha M. ShahLinda DiehlAkari Nakamura-UtsunomiyaLinda HollandAkash GuruLinda J. JohnstonAke SjöholmLinda PetijováAkhilandeshwari RavichandranLinda S. Forero-QuinteroAkhilendra Kumar MauryaLinda SchulerAkhilesh RaiLinda SmitAkhilesh YadavLinda ZottiAkhlaq MuhammadLindsay MarshallAkhtar AkhtarLindsay T. MichaloviczAkhtar AliLindsay TullochAkhtaruzzamanLindsey C PerkinAkihide OhkuchiLindsey Van HauteAkihide RyoLing LiAkihiko HiyamaLing TangAkihiko ItoLing WangAkihiko YamamotoLing YuAkihiro NaritaLing ZhuAkihito TsubotaLingbing KongAkikazu ShinyaLing-Chu ChangAkiko KashiwagiLinglin XieAkiko KozakiLingling MiaoAkiko MaedaLingwen DingAkiko MizokamiLing-Wen DingAkila WeerasekeraLingxia ZhaoAkilavalli NarasimhanLingxiao XieAkinobu OchiLingxin ChenAkinobu OtaLingzhi HongAkinori YabukiLinhao RuanAkio UenoLinlin GuoAkira HaraLinlin XuAkira HondaLinna AnAkira KitamuraLinqiang PanAkira MatsuuraLinshu LiuAkira MorikawaLinto ThomasAkira NakajimaLintong YangAkira NakanishiLinyi ZhuAkira NishimuraLinyuan MaAkira ObanaLinyue TongAkira ShinoharaLionel FrangeulAkira SugawaraLionel GamarraAkira TakamataLionel HebbardAkira TsuchiyaLipin JiAkira UmemuraLiping ChenAkira YamasakiLiping GuAkira YoshimiLiping WangAkira YoshinariLiqiang WangAkitaka ItoLiqing ZangAkito NakagawaLira GaysinaAkito TanedaLirong WangAkiya JourakuLisa BaumannAkiyoshi KawaokaLisa CangioliAklank JainLisa EbiharaAkoad Mohamed ElhassanLisa GiacomelliÁkos GellértLisa GouldÁkos KukiLisa Harrison-BernardÁkos LukátsLisa PangAkos MesterhazyLisa PetrellaAkram AshamesLisa Privette VinnedgeAkram ZamaniLisa SalvatoreAkriti SrivastavaLisa StrasserAkshata R. NaikLisa TimmonsAkshay GoelLisa Van Den BroeckAkshay KumarLisanework AyalewAkshay PandeyLisbet BrandiAla YarominaLise PingaultAlaa El-Din A BekhitLisethe MeijerAlaguvel ValliammaiLisheng HeAlain ChapelLishi XieAlain CouvineauLishu ShaoAlain Favre-RéguillonLishu WangAlain PerretLisong ChenAlain SarauxLister NicholasAlak MannaLi‐Ting MaAlam Md BadrulLiu LiuAlamelu BharadwajLiu YangAlami AnouarLiubov GorbachevaAlan HarperLiudmil AntonovAlan J. WhitmarshLiudmila Gerasimova-MeigalAlan PalmerLiudmila KhrustalevaAlana ThackrayLiudmyla V. GarmanchukAlarico ArianiLiuqi GuAlasdair J. NisbetLiverana LaurettiAlba CorellLivia CarrascalAlba García-RodríguezLivia D'AngeloAlba SilipoLivia ManzellaAlbena MomchilovaLivia PerfettoAlbert BassonLivio VitielloAlbert FlotatsLiviu DutaAlbert GiraltLiviu Mihai IrimiaAlbert GuskovLiviu SacarescuAlbert KireevLivius V D'UscioAlbert MoralesLiwei ChenAlbert PoaterLi-Wei LinAlbert Ribas-AgustíLiwei YangAlbert RizvanovLixia YueAlbert Sole GuitartLiya XuAlbert WölflerLiyun LiuAlberta BergamoLizabeth AllisonAlberto A. AmarillaLjilja TorovićAlberto AngioniLjiljana Mladenovic-SegediAlberto BartoloméLjubica CaldovicAlberto Canfran-DuqueLjubisa NikolicAlberto Comesaña-CamposLloyd DemetriusAlberto Dávalos HerreraLloyd FrickerAlberto Enrico MaraoloLobna ElkhadragyAlberto GianinettiLogan BanadygaAlberto J. Martín-RodríguezLogan VossAlberto Lo GulloLogan W. ColeAlberto López-LeraLoganathan PonnusamyAlberto MaccariLoic HilliouAlberto MacchiLoic LemonnierAlberto MantovaniLoka Raghu Kumar PenkeAlberto MellaLokesh Koodlur SannegowdaAlberto Molares VilaLokesh KumarAlberto Ouro VillasanteLokesh NarayananAlberto Ouro VillasanteLola Rueda-RuzafaAlberto Pérez-MediavillaLolita KursvietieneAlberto Rodríguez-ArchillaLong Chiau MingAlberto Sánchez-EstradaLong HanAlberto VerrottiLong JinAlbina TummoloLong XuAlbino BacollaLong ZhangAlbino CarrizzoLongbiao GuoAlbino EccherLonggang WangAlbrecht BindereifLong-Liu LinAlbrecht PiiperLong-Sen ChangAlbrecht ReichleLongteng TangAldo A Rodrıguez-MenchacaLong-Wa ZhangAldo CombarizaLongying ZhaAldo De SabataLoni BerkowitzAldo NicosiaLopez GianlucaAldo PezzutoLõpez-Candales AngelAldo RoccaroLora Petrie-HansonAldona KasprzakLorant JanosiAlec SimpsonLorant LakatosAlejandra Gutierrez-GuerreroLoredana BergandiAlejandro A. TapiaLoredana F. CiarmielloAlejandro BugarinLoredana MarcolongoAlejandro F. PradoLoredana MarcuAlejandro Gonzalez OriveLoredana RicciardiAlejandro Islas-JácomeLoredana Sabina Cornelia ManolescuAlejandro Miguel Figueroa-LópezLoredana TammaroAlejandro Olivares-HernándezLoredana UrsoAlejandro RomeroLorelei Irina BrasoveanuAlejandro Ruiz-GarcíaLoren E. SmithAlejandro SaettoneLoren HonaasAlejandro Samhan-AriasLorena Elena MeliţAlejandro Santos-LozanoLorena GurrieriAlejandro SanzLorena López FerrerasAlejandro Schcolnik-CabreraLorena Martín-AguilarAleksandar DjordjevicLorena PadillaAleksandar M. JeremicLorena PizarroAleksandar MehandzhiyskiLorena TorroniAleksandar S. TotLorena UrbanelliAleksandar ShkondrovLorena ZentilinAleksandar StojsavljevićLorenz AdlungAleksandar TotLorenz S. NeuwirthAleksander BurilovLorenza VantaggiatoAleksander FilarowskiLorenzo A. CingolaniAleksander HejnaLorenzo DadaltAleksander MahnicLorenzo DepauAleksander MendykLorenzo FarinaAleksander ShtilLorenzo FranceschettiAleksander ShyichukLorenzo GuazzelliAleksandr A. RubelLorenzo LippiAleksandr Andreevich BaykovLorenzo MagrassiAleksandr ArzamasovLorenzo MalatinoAleksandr BulaevLorenzo MantiAleksandr KazachenkoLorenzo PasottiAleksandr OreshonkovLorenzo PeruzziAleksandr PoliakovLorenzo PolimenoAleksandr S. KazachenkoLorenzo Rivas-GarcíaAleksandra AdamskaLorenzo StacchiniAleksandra AsaturovaLoreto BoixAleksandra Bielawska-PohlLoretta TuostoAleksandra BobaLori A. FischerAleksandra CzumajLori S. SullivanAleksandra Duda-ChodakLorina Badger-EmekaAleksandra FucicLoris ZamaiAleksandra Gilis-JanuszewskaLory Saveria CrocèAleksandra Glapa-NowakLothar BreckerAleksandra GlogowskaLotten RagnarssonAleksandra GolobLou MetherellAleksandra Grząbka-ZasadzińskaLouie Mar A. GangcuangcoAleksandra JaroszLouis IrvingAleksandra JoticLouis Kuoping ChaoAleksandra JovanovicLouis-Charles BélandAleksandra Karczmarska-WódzkaLouise Bjørkholt AndersenAleksandra KlimczakLouise HillerAleksandra KocotLouise Van Der WeydenAleksandra KołotaLouis-Marie BloyetAleksandra KoraćLoukas KanetisAleksandra KotyniaLourdes Cortes-DericksAleksandra KristoLourdes LledóAleksandra KrólickaLourdes MounienAleksandra KrólikowskaLourdes Villa-TanacaAleksandra M. BondžićLovro LamotAleksandra Maria StaszakLu AnAleksandra MaršavelskiLu LiAleksandra Nikolić-KokićLu Ping TanAleksandra PawlakLu WangAleksandra Platt-SamorajLuan De Souza LeiteAleksandra RanitovićLuana Beatriz Dos Santos NascimientoAleksandra RodackaLuana LuginiAleksandra RutkowskaLuana TonioloAleksandra RyłLuba TchertanovAleksandra S. KristoLubecka KatarzynaAleksandra Sałagacka-KubiakĽubica ĎudákováAleksandra SiekierzynskaLubica RauovaAleksandra SknepnekLubka RoumeninaAleksandra StaroszĽubomíra TóthováAleksandra SzczepankiewiczLubos CipakAleksandra SzczepkowskaLubov TimchenkoAleksandra SzydlowskaLuc J. MartinAleksandra SzydłowskaLuc LeybaertAleksandra UzelacLuc MaroteauxAleksandra WardzyńskaLuc SweversAleksandra Wisłowska-StanekLuc TéotAleksei A. TitovLuca AmbrosinoAleksei MachulkinLuca AmbrosioAleksei V. TrofimovLuca ArcariAleksei Vorob'EvLuca BonfantiAleksej ZarkovLuca BrambillaAleksey A. VedyaginLuca BurroniAleksey ChaulinLuca CiceroAleksey D. DrozdovLuca De ToniAleksey M. VishnyakovLuca Di GianfrancescoAleksey Michailovich ChaulinLuca FalzoneAleksey V. YakovlevLuca FontanesiAleksey V. ZaitsevLuca FreddiAleksey ZaitsevLuca GentilucciAlena Opalkova SiskovaLuca GiacomelliAlena ŠiškováLuca Guglielmo PradottoAleš AmbrožičLuca JovineAles CveklLuca Lo NigroAleš JerinLuca MagistrelliAleš StražeLuca MorandiAleš VokurkaLuca NervaAlessandra AloisiLuca OngaroAlessandra BigiLuca PaciniAlessandra BosuttiLuca PersaniAlessandra CarrubbaLuca PesceAlessandra ColciagoLuca PinziAlessandra De MatteisLuca Proietti De SantisAlessandra ErrigoLuca RaiteriAlessandra FerramoscaLuca RevelliAlessandra GamberucciLuca RoncucciAlessandra GhigoLuca SalaAlessandra GirottiLuca Salvatore MengaAlessandra LeoneLuca SancinetoAlessandra M. MartoranaLuca SementaAlessandra MarconiLuca SerenelliAlessandra MarescaLuca SigalottiAlessandra Maria VitaleLuca SimulaAlessandra MezzelaniLuca SoraciAlessandra MurabitoLuca Steardo, Jr.Alessandra P. PrincivalleLuca TadiniAlessandra PaganoLuca TianoAlessandra PalmaLuca UlianichAlessandra PierangeliLucas D. DiasAlessandra PisciottaLucas R. SmithAlessandra PiscitelliLuchang ZhuAlessandra PullieroLucia AnsaniAlessandra SorienteLucia BettediAlessandra StacchiottiLucia BiasuttoAlessandra TolomelliLucia BrodosiAlessandra VerardiLucia BuccarelloAlessandro A. JammalLucia CarboniAlessandro AlaimoLucia CataniAlessandro AngeliniLucia ČernákováAlessandro AnselmoLucia CondorelliAlessandro AntonelliLucia CoppoAlessandro BertoliLucia D'AccoltiAlessandro BonardiLucía FernándezAlessandro CannavoLucía Fernández CasanovaAlessandro CaputoLucia FrittittaAlessandro CastorinaLucía García-OrtegaAlessandro ChiolerioLucia GozzoAlessandro De SireLucia La SalaAlessandro D'UrsoLucia LandiAlessandro FaticaLucia LatellaAlessandro FeolaLucia MarcocciAlessandro FratiLucía Méndez LópezAlessandro GalaniLucia MerolleAlessandro GambellaLucia MorbidelliAlessandro GhezzoLucia MuracaAlessandro GranitoLucia RoccoAlessandro GrinzatoLucia ScisciolaAlessandro IeraciLucia StančiakováAlessandro LandiLucia TonucciAlessandro LeparuloLucía Yepes-MolinaAlessandro LucchesiLucian CopoloviciAlessandro MalaraLuciana Bolsoni LourençoAlessandro MalobertiLuciana BordinAlessandro MarianiLuciana GalettoAlessandro MarroneLuciana Pereira RangelAlessandro MeduriLuciana PisaniAlessandro MinhoLuciana RennaAlessandro OttaianoLuciane Alarcão Dias-MelicioAlessandro PalleschiLuciano BossoAlessandro PasseraLuciano GalantiniAlessandro PedrettiLuciano PironeAlessandro PerrellaLucie FischerovaAlessandro PezzellaLucien BovetAlessandro PoggiLucija GosakAlessandro PratesiLucinda V. ReisAlessandro RizzoLucio BarileAlessandro RolfoLucio Della GuardiaAlessandro RosaLucio Diaz-FloresAlessandro SalatinoLúcio FrigoAlessandro ScalaLucio PastoreAlessandro SgambatoLucjan JacakAlessandro SinigagliaLucjan StrekowskiAlessandro TozziLucjusz ZaprutkoAlessandro VolonterioLucrezia AversaAlessia ArcaroLucrezia BottalicoAlessia CastellinoLucyna Pomierny-ChamiołoAlessia CogatoLucyna WoźniakAlessia FabbriLudmila A FrankAlessia FaggianLudmila A. KasatkinaAlessia FilipponeLudmiła Grzybowska-SzatkowskaAlessia PaganelliLudmila KazdovaAlessia RemiganteLudmila L. SemenychevaAlessio AlesciLudmila PaškováAlessio Alessio OttavianiLudmila Prokunina-OlssonAlessio AndreoniLudmila V. PuchkovaAlessio ArdizzoneLudmila ZylinskaAlessio BiagioniLudovic AillotAlessio BonucciLudovic TrefondAlessio BucciarelliLudovic TricoireAlessio G. MorgantiLudovica CacopardoAlessio GnerucciLudwig E. HoelzleAlessio NogheroLuh SuriatiAlessio PaladiniLui Pauline Po YeeAlessio ReggioLuigi BellocchioAlessio ScarafoniLuigi BennardoAlessio TallaritaLuigi BrunettiAlevtina Mikhailovna SavichevaLuigi CappannoliAlex Al KhouryLuigi CatacuzzenoAlex AptLuigi De BellisAlex C. VargheseLuigi De MasiAlex Cleber Improta-CariaLuigi DonatoAlex ComptonLuigi FortunaAlex DavenporLuigi GrecoÁlex FerrerLuigi LeanzaAlex GregorieffLuigi MaranoAlex J. B. KreutzbergerLuigi MeccarielloAlex J. RathboneLuigi MessoriAlex KornkeLuigi MilellaAlex MichelsLuigi NapolitanoAlex Pauvolid-CorrêaLuigi PetramalaAlex TroitskyLuigi RosatiAlex Valadez-GonzálezLuigi RussoAlexa KlettnerLuigi SantacroceAlexander A. KorlyukovLuigi VitaglianoAlexander A. ShtilLuigia BruglieraAlexander A. StakheevLuigia D. De FalcoAlexander A. TyurinLuigia PazzagliAlexander AlijahLuigia TrabaceAlexander B. MohsenyLuis A. GonanoAlexander Batista DuharteLuis ÁguilaAlexander BaykovLuis Alberto Sanchez VargasAlexander BerezinLuis Alcides Olivos-OréAlexander BetekhtinLuis AlvesAlexander BotzkiLuis Andre MendesAlexander BrillLuis Ángel Rubio San MillanAlexander BunevLuís António Paulino PassarinhaAlexander DeutschLuis ApoloniaAlexander Dimitrov ShinkovLuis Augusto Eijy NagaiAlexander E. KabakovLuis C. BarrioAlexander E. VinogradovLuís C. N. Da SilvaAlexander FominLuis Contreras-MedinaAlexander GardnerLuis Daniel Goyzueta MamaniAlexander GasparskiLuis Del Pozo-YaunerAlexander GorokhovskyLuis Del ValleAlexander HawlitschkaLuis E. HernándezAlexander I. KokorinLuis Eduardo M. QuintasAlexander I. TitkovLuis EnjuanesAlexander IgnatovLuis Exequiel IbarraAlexander J. KastaniotisLuis Felipe Pérez-RomeroAlexander Ju. DudnikovLuis Fernando Macedo Di CristofaroAlexander KartashovLuis Fernando Magaña SolísAlexander KonevLuís Fernando PariziAlexander KornienkoLuís Fernando SchützAlexander KrivoruchkoLuis FrancoAlexander KyrychenkoLuis G. Sequeda-CastañedaAlexander LarinLuis González MacdowellAlexander LjubimovLuis GoyaAlexander LomzovLuis M. Alonso-ColmenarAlexander MackerellLuís M. S. LouraAlexander MironovLuis M. ValorAlexander OmelyanchikLuis ManceraAlexander OtahalLuis Manuel Montaño RamírezAlexander P. ByeLuis MasanaAlexander PanovLuis MonteiroAlexander PicoLuis MontenegroAlexander PogrebnjakLuis O. Soto-RojasAlexander PonomarevLuís Pedro Ferreira RatoAlexander RappLuis PeñalozaAlexander RemelsLuis Perez Garcia-EstañAlexander RingLuís PintoAlexander S. NovikovLuís Pinto Da SilvaAlexander SapozhnikovLuís R. RaposoAlexander SchrammLuis ReyAlexander ShcherbakovLuis RodrigoAlexander ShpakovLuis VarelaAlexander SidorenkoLuisa AgnelloAlexander SimakinLuisa Di StefanoAlexander SinyukovLuisa JordaoAlexander SobolevLuisa LampignanoAlexander SurinLuisa LanfranconeAlexander TamalunasLuisa LunaAlexander Th WuLuisa Maria FerreiraAlexander TsouknidasLuísa Mendes-JorgeAlexander TyshkovskiyLuisa Warchavchik HugerthAlexander Udal’TsovLuisa ZuccoloAlexander V. Artem’EvLuise LopesAlexander V. BaboshaLuiz Felipe D. PasseroAlexander V. GrishinLuiz Fernando Romanholo FerreiraAlexander V. KarchavaLuiz H. C. AssisAlexander V. KulikovLuiza Marek-JózefowiczAlexander V. MaltsevLuka Alexander ClarkeAlexander V. NosovLukas DedAlexander V. TimoshenkoLukas FojtAlexander V. VeselovskyLukas HlebaAlexander VasilyevLukáš KratochvílAlexander Von AppenLukáš KubalaAlexander VoronkovLukas LacinaAlexander WidiapradjaLukáš NajdekrAlexander Y. TsygankovLukas PfeiferAlexander YakovlevLukas RichteraAlexander ZhgunLukas RycekAlexander.P VoroninLukas StalpersAlexandr Alexandrovich KolobovŁukasz A. PoniatowskiAlexandr KazachenkoŁukasz BalewskiAlexandr KokovLukasz KozlowskiAlexandr V. BelousovŁukasz ŁopusiewiczAlexandra A. PopovaŁukasz M. MilanowskiAlexandra Antunes MastrobertiLukasz MalekAlexandra B. ShintyapinaŁukasz MałekAlexandra Balbir-GurmanLukasz NiedzwieckiAlexandra BelayewŁukasz PajchelAlexandra BogomazovaŁukasz PałkaAlexandra BurluiLukasz RadosinskiAlexandra CiorîțăLukasz S. BorowskiAlexandra Cristina MunteanuŁukasz SobottaAlexandra DamerauLukasz SzarpakAlexandra DubrovinaŁukasz SzczukowskiAlexandra I. GallerŁukasz SzeleszczukAlexandra KalogerakiŁukasz SzymañskiAlexandra KhlyustovaŁukasz WojtylaAlexandra KretzŁukasz ZapałaAlexandra NunesŁukasz ZdrojkowskiAlexandra P. MarquesLuke J. MortensenAlexandra PauloLuke J. NeyAlexandra PeregrinaLuke ParkitnyAlexandra Peregrina LavínLuminita CrisanAlexandra Pérez-SerraLuminita Gabriela MarutescuAlexandra PotekhinaLuminita GhervaseAlexandra RibaritsLuminita MarinAlexandra Ripszky TotanLuminita PaduraruAlexandra RoiLung-Chien ChenAlexandra S. SilchenkoLusheng FanAlexandra Simon-GruitaLu-Sheng HsiehAlexandra WormitLu-Ting KuoAlexandre AbhervéLutz NährlichAlexandre BoyerLutz SchmittAlexandre G. De BrevernLutz TautzAlexandre GoncalvesLutz WollinAlexandre Gonzalez-RodriguezLuyao ZhengAlexandre HinzpeterLuyckx KimAlexandre KauskotLuz Berenice López-HernándezAlexandre LamasLuz María Paucar-MenachoAlexandre M. P. BotasLydia Giménez-LlortAlexandre Ninhaus-SilveiraLydia M. CastelliAlexandre QuintasLydia VisserAlexandre SmirnovLynda A. MorrisonAlexandre SpechtLyndsey MuehlingAlexandre VetcherLynn M. PezzaniteAlexandrina BurlacuLysangela R AlvesAlexandrina NanLysett WagnerAlexandrina S. NikovaLyuba VarticovskiAlexandros BrotisLyuben ZagorchevAlexandros G. SykarasLyubov E. SalnikovaAlexandros PapachristodoulouLyudmila Bel'SkayaAlexandros TataridasLyudmila E. MuravnikAlexandru A. SaboLyudmila GulyaevaAlexandru BejinariuLyudmila I LarinaAlexandru BucurLyudmila KhrabrovaAlexandru BurlacuLyudmila N. ZhichkinaAlexandru Mihai GrumezescuLyudmila RachekAlexandru PopaLyudmila S. DolmatovaAlexei ChukhlovinM Asun CanteraAlexei KamshilinM Pilar DoconAlexei SlepzofM. Amirul IslamAlexei Terent’EvM. Anwar IqbalAlexei Yu LupatovM. ArulperumjothiAlexey A. BogdanovM. Azizur RahmanAlexey A. IvanovM. Bruce MaciverAlexey AfoninM. C. GuerreraAlexey AlekseevM. Carmo LançaAlexey BondarM. D. SalmanAlexey BoykoM. Dulce EstêvãoAlexey ChubarovM. E. PembleAlexey EgorovM. Elena Martín PalmaAlexey ErmakovM. Esther GallardoAlexey F. TopunovM. Furkan BurakAlexey G. KolmakovM. Hasan MohajeriAlexey G. MittenbergM. I. OjovanAlexey KovalM. Ina ArnoneAlexey KuzmichM. Isabel IsmaelAlexey MalyginM. JameelAlexey MeshkovM. Julia BujánAlexey MorgounovM. P. PeppelenboschAlexey N. SkvortsovM. Pilar Vázquez-TatoAlexey NazarovM. Quadir SiddiquiAlexey NikulinM. R. MohammadabadiAlexey SafonovM. R. MozafariAlexey SarapultsevM. S. EliwaAlexey SokolovM. Saadeh SuleimanAlexey SukhorukovM. Sonia FreireAlexey TerskikhM. SteenmanAlexey V. FeofanovM. Z. JankovicAlexey V. IvanovMª Rosario LuquinAlexey VasilchenkoMa. Del Rocio Banos LaraAlexey Yu SemenovMaarit SuomalainenAlexey ZaitsevMaarit TiirikainenAlexios A. AlexopoulosMaayan WaldmanAlexios Vlamis-GardikasMac GardnerAlexis GalanisMacarena Gomez-LiraAlexis GrandeMacarena MarínAlexis M ZiembaMacdonald WickAlexis NzilaMaciej BernackiAlexis StamatikosMaciej BuśkoAlfio BattiatoMaciej CieplakAlfonso Fernández-ÁlvarezMaciej DaniszewskiAlfonso Gutiérrez-AdánMaciej DawidowskiAlfonso MartinezMaciej GagatAlfonso Olaya AbrilMaciej GrzybekAlfonso ZambonMaciej JarzebskiAlfred GoldbergMaciej JedlińskiAlfred TauberMaciej KamaszewskiAlfredo BellonMaciej KochanowskiAlfredo CaturanoMaciej KrasnodębskiAlfredo CiccodicolaMaciej MaciejczykAlfredo Cruz-GregorioMaciej NastajAlfredo Cruz-RamirezMaciej PrzybyłekAlfredo G. CasanovaMaciej Robert KrawczyńskiAlfredo GorioMaciej SałagaAlfredo GueraMaciej SkotakAlfredo IandoloMaciej SkrzypczakAlfredo MartínezMaciej SzaleniecAlfredo MinguelaMaciej TarnowskiAlfredo PalopMaciej WiatrAlfredo Téllez ValenciaMaciej WitwickiAlfredo Téllez-ValenciaMaciej WoźnyAlgimantas TamelisMaciej ZdunAli AbghariMaciej ZielińskiAli AhmadMadalina CiobanuAli AkalinMadalina DumitrescuAli AnwarMadalina Irina MitranAli AygünMadalina SandulescuAli ChaariMadalina Simona BaltatuAli DehshahriMădălin-Iancu EnacheAli DoryabMadan K. KharelAli Ekrem YesilkanalMadara AuzenbahaAli ImranMadaswamy S. MuthuAli IrfanMaddalena Del GalloAli MahtaMaddalena ParafatiAli OzcanMadelaine RomainAli R. JazirehiMadhab Kumar SenAli Reza KamaliMadhavan NampoothiriAli SadeghianmaryanMadhavi TripathiAli SaeedMadhu GoyalAli SakMadhu OusephAli Shaan Manzoor GhummanMadhu Sudhana SaddalaAli Tan Kee ZuanMadhuja SamaddarAli ZarrabiMadhulika PradhanAlia TelliMadhumita ChatterjeeAlica PizentMadhusmita PanigrahyAlican GulsevinMadhuvanthi VijayanAlice AlessenkoMadison C. B. PatonAlice BerardoMadson AlmeidaAlice ConigliaroMaedeh GhasemiAlice CortesiMaegen BorzokAlice G. WillisonMafalda LaranjoAlice GrossiMafalda PintoAlice GualerziMafalda SantosAlice M TurnerMagali ReyAlice MeroniMagda H. AbdellattifAlice MicheluttiMagda PálAlice PievaniMagdalena BaymakovaAlice S. PereiraMagdalena BogutaAlice VerticchioMagdalena BrodaAlicia Fernández-San MillánMagdalena CalAlicia HeathMagdalena Cristina StanciuAlicia Mayeuf-LouchartMagdalena CzemierskaAlicia OteroMagdalena Dziagwa-BeckerAlicia R. BerardMagdalena E Paweł'KowiczAlicia Rodríguez-BarberoMagdalena GawłowskaAlicia RoqueMagdalena GierszewskaAlicja ChrzanowskaMagdalena GłąbAlicja Dąbrowska-KugackaMagdalena GórnickaAlicja KluczykMagdalena GrillAlicja M. KmiecikMagdalena HurkaczAlicja TymoszukMagdalena IorgaAlida Kindt-DunjkoMagdalena KaraśAlin CiobicaMagdalena KostrzewaAlin Laurenţiu TatuMagdalena KowalewskaAlina BoraMagdalena Krajewska-WłodarczykAlina ConstantinMagdalena KrauzeAlina Elena MoacaMagdaléna KrulováAlina Georgescu (Serb)Magdalena Kurnik-ŁuckaAlina González-QuevedoMagdalena LaskowskaAlina KafelMagdalena Lebiedzinska-ArciszewskaAlina KuryłowiczMagdalena LisAlina LierschMagdalena MachowskaAlina PaunescuMagdalena Maj-ZurawskaAlina PykaMagdalena Markowicz-PiaseckaAlina R. IzatulinaMagdalena MititeluAlina TanaseMagdalena NoszczyńskaAlina WagiranMagdalena OćwiejaAlina WilkowskaMagdalena OrzechowskaAlina-Andreea ZimtaMagdalena OsialAline Maria Da SilvaMagdalena PłotkaAline ProbstMagdalena RadovicAline Rangel-PozzoMagdalena RatajczakAline Silva Mello CésarMagdalena RauschAline Yen Ling WangMagdalena SałdykaAlireza Nezamzadeh-EjhiehMagdalena StaniszewskaAlireza ZarebidokiMagdalena StaszczakAlisa SokolovskayaMagdalena StobieckaAlisha PrasadMagdalena SzaryńskaAlison AndersonMagdalena SzeligaAlison CareyMagdalena SzopaAlison DominguesMagdalena SzymanskaAlison J. ForheadMagdalena WójciakAlistair J. GunnMagdalena Wójcik-JagłaAlistair V NunnMagdalena WołoszyńskaAlistair Vw NunnMagdalena Wróbel-KwiatkowskaAliyu MohammadMagdalena WronaAlja ZottelMagdalena ZdanowiczAlka GuptaMagdalena ZiąbkaAlka JaggessarMagdalena ZielinskaAlkmini T. AnastasiadiMagdalena ZieliñskaAlla F FominaMagdalena ŻukAlla KrasikovaMagdalena ŻurawekAlla MirgorodskayaMagdalena ŻychowskaAlla MitrofanovaMagdalene J. SeilerAlla SalminaMagdolna NagyAllah DittaMagdolna SzantoAllan CaplanMagesh MuthuAllan GoldsteinMaggie BartlettAllan Klynger Da Silva LobatoMaggie LamAllan Martín Carrillo-BaltodanoMagne BørsetAllison B ReissMagnus KjaergaardAllison DidychukMagnus SchmidtAlma P PerrinoMagriet A Van Der NestAlmerinda Di VenereMaha CouchaAlmudena FernándezMahadevamurthy MuraliAlmudena MartiMahar FatimaAlois CizekMahboob AlamAloisio AnnamariaMahdi ZavvarAlok K. VermaMahdieh MehrpouriAlok PaulMahendran SekarAloke DasMaher Abd El-NabiAlon SilberbushMahesh AttimaradAlson TimeMahesh KasthuriAlsu SaifitdinovaMahesh Kumar JoshiAltaf MohammedMahesh NarayanAltaira D. DearbornMahesh RachamallaAlthaf ShaikMahesh Raj NepalAlvarado-Ibarra MarthaMahi M. MohiuddinAlvaro ArdilesMahima SharmaÁlvaro Astasio PicadoMahipal KesawatÁlvaro Conrado Ucero HerreríaMahipal Singh KesawatÁlvaro Del RealMahmood AhmedÁlvaro F. FernándezMahmood BaraniAlvaro González‐DominguezMahmoud Al-KhrasaniAlvaro Hermida-AmeijeirasMahmoud BendaryAlvaro MaciasMahmoud ElfakyAlvaro MallagarayMahmoud ElsayedÁlvaro Martínez-CamarenaMahmoud GhasemnezhadÁlvaro MeanaMahmoud HuleihelÁlvaro MourenzaMahmoud KandeelAlvaro OrtegaMahmoud MadkourÁlvaro Plaza ReyesMahmoud MirzaeiÁlvaro Santana-GarridoMahmoud NasrAlvaro YogiMahmoud NasrollahzadehAlyx TaylorMahmoud SofyAmadeus DobrescuMahmoudreza HadjighassemAmador Menéndez-VelázquezMahmud HasanAmaia ArranzMahmudul HassanAmaia RodríguezMahnaz Bahri KhomamiAmal El-ShalMahsa DabaghAmal NarayananMahwash MukhtarAmalendu GhoshMai WatanabeAmalia Floriou-ServouMaia De LucaAmalia GordanoMaicol ManciniAmalia S. JuradoMaidul IslamAmalia StefaniuMaija CastrenAman KumarMaija NissinenAman SharmaMaija TenkanenAmanda Carroll-PortilloMaik GollaschAmanda Cuevas-SierraMaike KrauseAmanda GendersMaikel Castellano-PozoAmanda GleixnerMainul HaqueAmanda IglesiasMaíra RodriguesAmanda L. FuchsMaire PetersAmanda LumsdenMaitane AsensioAmanda PacholakMaitree SuttajitAmani Al-OthmanMaizbha Uddin AhmedAmany MohamedMaja BoczkowskaAmany RagabMaja C. PagnaccoAmany TawfikMaja Cokarić BrdovčakAmar SinghMaja D. NešićAmarajothi DhakshinamoorthyMaja Herak BosnarAmarendra N. MisraMaja HitlAmaresh C . PandaMaja Jazvinšćak JembrekAmaresh RanjanMaja Krstić RistivojevićAmarinder SinghMaja LudvigsenAmarinder Singh SinghMaja MarušičAmarish YadavMaja Matoša KočarAmbareen KausarMaja MatulicAmber Parry-StrongMaja MatulićAmber StratmanMaja MiskulinAmbrogio P. LonderoMaja PotokarAmbrož KregarMaja SabolAmedeo LonardoMaja ŠantakAmedeo VetereMaja TomičićAmeeduzzafar ZafarMajdy IdreesAmeer Fawad ZahoorMajed AlabdaliAmeera AlsadeqMajed H WakidAmelia J. HessheimerMajid Rasool KamliAmelia Maria GamanMaki TokumotoAmelia Ramon-LopezMakiha FukudaAmena AliMakiko Mochizuki-KashioAmer AhmedMakio TakedaAmer DababatMakoto EndoAmerico VianaMakoto HaradaAmerigo GiudiceMakoto HasegawaAmeya D GondhalekarMakoto HashimotoAmie MoyesMakoto HirakawaAmila SuraweeraMakoto KawamukaiAmin DerouicheMakoto MakishimaAmin FatoniMakoto MatsushitaAmin MojiriMakoto NodaAmin RabieiMakoto SugayaAmin ShahrokhiMakoto TsunodaAmin ShavandiMakram MerimiAmin ZakeriMaksim Leonidovich FilipenkoAminallah TahmasebiMaksim RebezovAmir Amiri-YektaMaksym M. FizerAmir MahboubiMaksymilian ProndzynskiAmir MonfaredanMalathi ArumugamAmir RostamiMalbert Charles-HenriAmir S. YazdiMalcolm PageAmir Sasan Mozaffari NejadMalcolm Scott DuthieAmir ShemiraniMalek Ben Ahmed AdouniAmirah GozaliMałgorzata AdamiecAmir-Babak Sioofy-KhojineMałgorzata BiałekAmir-Houshang ShemiraniMałgorzata BlatkiewiczAmirkianoosh KianiMałgorzata BrindellAmirul IslamMalgorzata BurekAmit AdhikaryMałgorzata ChlabiczAmit HalderMałgorzata CytryńskaAmit K. MaitiMalgorzata CzyzAmit KhuranaMalgorzata DominoAmit KumarMałgorzata DudaAmit Kumar SahaMałgorzata E. ZakrzewskaAmit P. BhavsarMalgorzata GajewskaAmit PalMałgorzata GieryńskaAmit SinghMałgorzata GodalaAmit TripathiMałgorzata GraczykAmit VikramMałgorzata GrembeckaAmitava AdhikaryMalgorzata GrzesiakAmitava MoulickMałgorzata GrzesiukAmitava MukherjeeMałgorzata GrzybAmiya PatraMalgorzata GutkowskaAmjad BalangeMalgorzata HolynskaAmjad ElmashalaMałgorzata Janicka-RussakAmjad KhanMałgorzata JarończykAmmad Ahmad FarooqiMałgorzata JeleńAmmar Abdullkader BoudakaMałgorzata K. Włodarczyk-BiegunAmmar BaderMałgorzata KapralAmmar KadiMałgorzata KlimekAmmar TahirMalgorzata KolpaAmna AnjumMalgorzata KorzeniowskaAmnon BrzezinskiMałgorzata Kotula-BalakAmol NankarMalgorzata Krok-BorkowiczAmosy M'KomaMalgorzata KucinskaAmr FoudaMałgorzata KujawskaAmr GamalMałgorzata Lewandowska-SzumiełAmr M. AbdelhamidMalgorzata LobockaAmram EshelMałgorzata ŁochyńskaAmrendra K AjayMalgorzata MajAmrendra MishraMalgorzata Maria JerzakAmresh PrakashMałgorzata MatysekAmrinder SinghMałgorzata MiastkowskaAmrit KaphleMalgorzata NaleczAmrita MandalMałgorzata Natalia MojsakAmrita PaiMałgorzata Nattich-RakAmrita SalviMałgorzata NykielAmritlal MandalMałgorzata OchotaAmro AbulilaMałgorzata Pietrowska-BorekAmro AmaraMalgorzata Polz-DacewiczAmro M. SolimanMałgorzata Polz-DacewiczAmulya YaparlaMałgorzata Pomorska-MólAmy BrowerMałgorzata PrzybyłoAmy E. ChadwickMałgorzata RobakAmy E. Wiberley-BradfordMałgorzata RogalińskaAmy MessersmithMałgorzata ŚliwinskaAmy WinshipMałgorzata StarowiczAmy XuMałgorzata StpiczyńskaAn Eng LimMałgorzata SzczukoAna AlcudiaMałgorzata SztankeAna Babic PerhocMałgorzata SzymiczekAna BelchiorMałgorzata Tatarczak-MichalewskaAna Belen Garcia-RedondoMałgorzata TokłowiczAna BettencourtMalgorzata WaleronAna BorotaMałgorzata WaleronAna BudimirMałgorzata WisłowskaAna ButrónMalgorzata Witkowska-ZimnyAna C. GonçalvesMałgorzata WojciechowskaAna C. Martins CavacoMalgorzata Zakłos-SzydaAna CanadasMałgorzata ZimowskaAna CandiotaMaliha ZahidAna Carla BatissocoMalik AydinAna CaruntuMalik HamaidiaAna CarvalhoMalika FaouziAna CicvaricMalin SandbergAna Clara Abadía-MolinaMalin WennströmAna Cláudia Correia CoelhoMalinee SriariyanunAna Corte-RealMalini MukherjeeAna Cristina GonçalvesMalkeshkumar PatelAna Cristina QueirósMallika ValapalaAna Cristina RegoMallique QaderAna CrnkovicMallorie PoetAna CuendaMalte SgoddaAna D. SimonovićMamata SinghviAna DascaluMamatha GarigeAna DekanicMami MurakamiAna Do ValeMamoru IsemuraAna Filipa PiresMamoru TakedaAna Florencia Vega-BenedettiMamta Patel NagarajaAna Henriques MotaMan LiuAna I ArrobaMan P. HuynhAna I. ArrobaMan ZhouAna I. FloresMana MukherjeeAna I. MatesanzManabu KatoAna Isabel Álvarez-MercadoManabu NatsumedaAna Isabel EsquifinoManal FawzyAna Isabel FernandezManal SabryAna Isabel Gracia LostaoManasi TamhankarAna Isabel López SeséManda YuAna Isabel PadrãoMandakhbayar Nandin-ErdeneAna Isabel PrietoManel CampsAna Isabel SanchoManendra Babu LankadasariAna J. Chucair-ElliottManesh NautiyalAna Jimenez-GarciaManfred KircherAna KramarManfred PhilippAna LeahuManfred SagerAna Leticia Fornari CapraraMani AlikhaniAna Lúcia CardosoManickam SugumaranAna Lúcia LuísManikandan MuthuAna Luísa De Sousa-CoelhoManikandan RamasamyAna Luisa Garcia OliveiraManikant TripathiAna LuzioManinder SinghAna M. Carmona-RibeiroManindra TiwariAna M. GeerManish AdhikariAna M. TomásManish AroraAna Margarida PereiraManish CharanAna Margarida SilvaManish KumarAna Margraida AraújoManish SinghAna Maria CastrucciManish TripathiAna Maria Dos Santos Rosa Da CostaManish Vijaya KumarAna Maria EijanManisha BajpaiAna Maria GheorghiuManisha NigamAna María Gimenéz-ArnauManit NuinoonAna María Navarro IncioManjeet SinghAna Maria PutzManju M. ChandraAna Maria RosuManju Rawat SinghAna Maria UdreaManjulatha MekapoguAna Marija Jagatić KorenikaManjunatha S. MuttigiAna MaroteManjurul HaqueAna Marta De MatosManlio TolomeoAna Marta PereiraManmeet BhallaAna Martín-SánchezManohar KodavatiAna MolinaManohar KugajiAna MonteiroManoj AmrutkarAna Moreno-ÁlvarezManoj B MenonAna MornarManoj ChoudharyAna NicolauManoj GargAna OliveiraManoj K. SekhwalAna OmenatManoj KulkarniAna PaleiManoj KumarAna Paula PiedadeManoj Kumar TripathiAna Paula RamosManoj PandeyAna Paula SantosManoj ThapaAna Pérez De CastroManojkumar ArthikalaAna PilipovicManomi PereraAna PintoManoshi GayenAna PretoManping JiaAna Quelle-RegaldieMans BroekgaardenAna R. Machado-SantosMansi SharmaAna RamosMansoor AlshehriAna Rita CalixtoMansoor HussainAna Rita CarlosMansoore EsmailiAna Rita Costa-PintoMansour A. AlghamdiAna Rita Elias BrásMansour HaddadAna Rita FerreiraMantas ZiaunysAna Rosa Rincón-SánchezMantasha IdrisiAna SampaioManu KumarAna SilvaManu PrasadAna Sofia Leitão CarvalhoManu RangachariAna Sofia VallésManuel A. MaliqueoAna Sofia VinhasManuel A. MatamorosAna SousaManuel Álvarez-RodríguezAna UrzainquiManuel António Rodrigues TeixeiraAna ValadoManuel AurelianoAna Vrsalović-PresečkiManuel BelliAnabela BorgesManuel BlázquezAnabela VeigaManuel CantosAnaclet NgezahayoManuel Durán-PovedaAnaïs Pitto-BarryManuel Espinosa UrgelAnalia TomatManuel GarrosaAnalisa PastoreManuel Gutierrez-AguilarAnaluce Canha-GouveiaManuel López-LópezAnamaria BechirManuel PiñeroAnamaria BrozovicManuel RamírezAna-Maria IordacheManuel SalzmannAnamaria JurcauManuel Sanchez-MartinAnamaria SavuManuel WilbringAna-Maria TănaseManuela BartoliniAna-Maria UdreaManuela BogAna-Marija DomijanManuela BüttnerAnamika PandeyManuela CervelliAnand AnbarasuManuela ChiperAnand DesaiManuela CipollettiAnand K. RohatgiManuela CurcioAnand Prakash SinghManuela LeriAnand SharmaManuela MalatestaAnand SinghManuela MatosAnand ThirupathiManuela MontanaroAnandharajan RathinasabapathyManuela NeumanAnandram VenkatasubramanianManuela OliveiraAnandrao Ashok PatilManuela RabaglioAnani K. AfanouManuela Ramos SilvaAnanta Prasad ArukhaManuela RolliniAnas AhmadManuela SabatinoAnas M Abdel RahmanManuela ViolaAnas ShamsiManuela ZonfrilloAnastasia A MinervinaManveen SethiAnastasia A. AnashkinaManzoor RatherAnastasia A. PonomaryovaMao LiAnastasia D. PournaraMaqsood MalikAnastasia FrolkovaMar BennasarAnastasia G. MitseaMar Carmona-AbellanAnastasia NazarovaMar Carrión CaballoAnastasia NijnikMar InfanteAnastasia ProdromidouMar QuiñonesAnastasia SpiliopoulouMara BrancaccioAnastasia SveshnikovaMara GyöngyvérAnastasia TsingotjidouMara MarongiuAnastasia V. BochenkovaMara MassimiAnastasiia K. KimeklisMara MazzoniAnastasiia KotlyarovaMara NoveroAnastasiia PetushkovaMāra PilmaneAnastasiia S. BoikoMarasanapalli ArunasreeAnastasios AlatzasMarat EzhovAnastasios GotziasMarat MinlebaevAnastasios LymperopoulosMarat R. KhaliluevAnastasios MarkopoulosMarawan AbdelbasetAnastasios MelisMarc A. IliesAnastasiya A. SokolovaMarc BartoliAnastasiya SolovievaMarc BeltràAnastasiya V. PetrovaMarc De La RocheAnastasiya Yurievna GlagolevaMarc DorenkampAnastasya AnashkinaMarc EkkerAnatoly DvurechenskiiMarc FahrnerAnatoly V. ZherdevMarc FuchsAnatoly VereshchaginMarc GilbertAnbarasu KannanMarc HilhorstAnbazhagan SathiyaseelanMarc L. PuseyAnbukkarasi MuniyandiMarc Le VéeAnca BobircaMarc LussierAnca DinischiotuMarc MarescaAnca DoceaMarc MauryAnca FilimonMarc PawlitzkiAnca GafencuMarc PoirotAnca Giorgiana GrigorašMarc Van ZandvoortAnca Maria CimpeanMarc W. T. WertenAnca MazareMarc ZimmerAnca Mihaela Pantea StoianMarc-André LécuyerAnca Niculina CadinoiuMarcel Aguilella-ArzoAnca Oana DoceaMarcel AmichotAnca RoseanuMarcel BermudezAnca Roseanu ConstantinescuMarcel HeersAnca StanaMarcel KrzanAnca UngurianuMarcel Matei DudaAnca ZanfirescuMarcel Pascal BeierAnca-Roxana PetroviciMarcel SchmidtAnchal SharmaMarcel SchulzAn-Chen ChangMarcel TawkAnda BarkaneMarcela DendisováAndaleb KholmukhamedovMarcela PereiraAnder VergaraMarcela RodrigueroAnder Vergara AranaMarcelina MalinowskaAnders HoferMarcella BarbarinoAnders KallnerMarcella NeriAnders Lade NielsenMarcella NunziatoAnders LarssonMarcella RealeAnders Løbner-OlesenMarcella TazzariAnders MellemgaardMarcella Zampoli TroncarelliAnderson GeorgeMarcello CandelliAndi AlijagićMarcello CasaAndleeb KhanMarcello CasertanoAndrás BallaMarcello CeciAndras BikovMarcello CondorelliAndras LekoMarcello GovoniAndrás MihályMarcello IasielloAndrás PappMarcello LocatelliAndrás RabMarcello MaggioliniAndrás SzabóMarcello ManfrediAndrás SzászMarcello MocciaAndraz DovnikMarcello NaccaratoAndraẑ DovnikMarcello TagliaviaAndré A. SantosMarcelo A. LimaAndré Alves DiasMarcelo Alves Da Silva MoriAndré Conceição PereiraMarcelo AssisAndré Da CostaMarcelo CatalanAndre Eccel VellwockMarcelo Cavenaghi Pereira Da SilvaAndré GerberMarcelo CorreiaAndré HazoutMarcelo DornelasAndre Kajdacsy-BallaMarcelo EhrlichAndre LechelMarcelo MaruchoAndre Luiz Pasqua TavaresMarcelo Paes BarrosAndré M TravessaMarcelo Palma SirciliAndré M. CantinMarcelo Sousa SilvaAndré MeloMarcelo Tigre MouraAndré MenkeMarcia HiriartAndre SilvaMárcia Regina CominettiAndré SimãoMarcia SaraivaAndrea AlexandreMarcin ArciszewskiAndréa Alice Da SilvaMarcin CzerwinskiAndrea Almeida MiyasakaMarcin CzerwińskiAndrea Anna SowislokMarcin CzopAndrea AntosovaMarcin FeldoAndrea AstolfiMarcin GnybaAndrea AtreiMarcin Gra̧ZAndrea BalduitMarcin HeljakAndrea BalerciaMarcin KosmalskiAndrea BalliniMarcin KremieniewskiAndrea BattistoniMarcin KucińskiAndrea BeccioliniMarcin LipowczanAndrea BianconiMarcin ŁyszkiewiczAndrea BilgerMarcin MichalakAndrea BorghiniMarcin NicośAndrea BotturiMarcin OzarowskiAndrea BuneaMarcin OżarowskiAndrea ButeraMarcin RozalskiAndrea CaporaleMarcin RóżewiczAndrea CitarellaMarcin SajekAndrea CopettaMarcin SamiecAndrea CuppMarcin SchmidtAndrea D'AmatoMarcin SiwekAndrea De PieriMarcin SochalAndrea DefantMarcin W. BańburaAndrea Del CampoMarcin WarminskiAndrea Di CristoforiMarcin WekwejtAndrea Elio SprioMarcin WłochAndrea FerrignoMarcin Wojciech LisAndrea FinMarcin ZarowskiAndrea GalisovaMarcin ZiemniakAndrea GalliMárcio F. SimãoAndrea GerbinoMarco A. Obregón-MendozaAndrea GhiroldiMarco A. RamosAndrea GianniniMarco AllinoviAndrea GiansantiMarco Antonio Hernández-LepeAndrea GilioliMarco BarchiAndrea Giuseppe MaugeriMarco Bauzá ThorbrüggeAndrea HartwigMarco BiggiogeraAndrea Henriques PonsMarco BoAndrea Herrero-CerveraMarco BorsariAndrea Hoelbl-KovacicMarco BruschiAndrea HoffmannMarco C. CavacoAndrea HuwilerMarco CalabròAndrea L. DicarloMarco CambiaghiAndrea L. HildebrandMarco CamperaAndrea LuvisiMarco CandeiasAndrea M. PotocnyMarco CapriniAndrea MarcantoniMarco CarlottiAndrea Maria GuarinoMarco CarusoAndrea MarkovinovicMarco CavagliàAndrea MartinelliMarco ChinoAndrea MezzettaMarco ColombiniAndrea MohrMarco CordaniAndrea MulliriMarco D’AbramoAndrea MünsterbergMarco DacremaAndréa Novais Moreno-AmaralMarco Dal MaschioAndrea PaganoMarco De MartinoAndrea Paola Rojas GilMarco E. M. PelusoAndrea PapaitMarco EspositoAndrea PelusoMarco FioreAndrea PepperMarco FoganteAndrea Pereira RegnerMarco FoliniAndrea PerrelliMarco FracassettiAndrea PiccinMarco G. AlvesAndrea Pietropolli CharmetMarco G. SchiavoneAndrea PozziMarco GaviraghiAndrea RagusaMarco GentileAndrea RaymondMarco GerdolAndrea ResoviMarco GovoniAndrea RomaniMarco InvernizziAndrea Roxanne J. AnasMarco Juárez-EstradaAndrea SanchiniMarco KochAndrea SartoriMarco La MarraAndrea ScharfMarco LelliAndrea SevcovicovaMarco M. OchsAndrea SistiMarco MainardiAndrea SpallarossaMarco MalferrariAndrea SpiniMarco MangiagalliAndrea StoccoroMarco MartinoAndrea TarozziMarco Matteo CicconeAndrea TelekMarco OrecchioniAndrea TrevisanMarco OrlandoAndrea UrbaniMarco PalumboAndrea VecchiolaMarco PaolantoniAndrea VergalloMarco PelinAndrea VolanteMarco PessoaAndrea VolpeMarco PiccoliAndreadi AikateriniMarco PortelliAndréanne LupienMarco R. CelioAndreas EbertMarco RacchiAndreas EigentlerMarco RinaudoAndreas EisenreichMarco RiosAndreas G. ChiocchettiMarco RohdeAndreas GrützkauMarco RossiAndreas H. H. KottmannMarco SavareseAndreas Haahr LarsenMarco ScaviniAndreas Herbert TeuschlMarco SchiavoneAndreas HerbstMarco ScortichiniAndreas IhleMarco SegattoAndreas Ioannis PapadakisMarco SisignanoAndreas JägerMarco TatulloAndreas JekleMarco TerreniAndreas KoulourisMarco TorresAndreas KoupparisMarco ZannottiAndreas KruegerMarco ZarattiniAndreas KuhnMarc-Olivier DeguiseAndreas LambertzMarconi Batista TeixeiraAndreas LösslMarcos Antonio González-LópezAndreas MautnerMarcos Arturo Martínez-BanaclochaAndreas MerdesMarcos Edgar HerkenhoffAndreas OuranidisMarcos Soto-HernándezAndreas PetryMarcus BahraAndreas RitterMarcus CoratAndreas RoosMarcus DymondAndreas S. KalogirouMarcus LettauAndreas SchlatterMarcus StoetzerAndreas SchmutzlerMarei SammarAndreas SchwingshacklMarek BiesiadeckiAndreas StengelMarek BolanowskiAndreas WeberMarek BrenišinAndreea BorleaMarek D. KoterAndreea CosoveanuMarek DługoszAndreea IacobMarek DrozdzikAndreea-Iren SerbanMarek FolAndreea-Teodora IacobMarek J. ŁosAndreev YaroslavMarek KieliszekAndrei AnghelMarek KonopAndrei ColitaMarek KoutnýAndrei EgorinMarek KowalczykAndrei I. KhlebnikovMarek LecewiczAndrei KramerovMarek LiszewskiAndrei KrasilinMarek MuriasAndrei PotapovMarek OrłowskiAndrei S. RodinMarek PiątkowskiAndrei SurguchovMarek PokornyAndrei Vasile NastutaMarek PruszynskiAndrei Victor SanduMarek RuchałaAndreia De CarvalhoMarek RusinAndreia GarcêsMarek ŠebelaAndreia M. SilvaMarek SkrzypskiAndreia N. CarvalhoMarek UrbanskiAndreia RosatellaMarek WesołowskiAndreia RuivoMarek WisniewskiAndrej CokanMarek WojnickiAndrej FrolovMargaret GradovaAndrej ThurzoMargaret H. ChangAndrej ZupanMargaret HurleyAndreja JakasMargaret L. AxelrodAndrés Calderín GarcíaMargaret L. BritzAndrés D. KleinMargaret OttavianoAndrés G Vidal-GadeaMargaret RyanAndrés García MonteroMargaret SimonianAndrés J. CortésMargaret StefaterAndres MäeMargaret T. T. Wong-RileyAndres Sanz GarciaMargarida Duarte AraújoAndrés TrostchanskyMargarida F. B. SilvaAndrew AllanMargarida FardilhaAndrew B. GipsonMargarida Gama-CarvalhoAndrew ChantryMargarida SantanaAndrew ClaytonMargarita A. GoldbergAndrew D. CartmillMargarita A. SazonovaAndrew D. GreenhalghMargarita A. SazonovaAndrew EagleMargarita AguileraAndrew Edet EkpenyongMargarita D. ApostolovaAndrew FribleyMargarita E. NeganovaAndrew FryMargarita IvanovaAndrew G. MatveenkoMargarita KambourovaAndrew Graham SmithMargarita MartínAndrew Guy MessengerMargarita NeganovaAndrew I. M. GreerMargarita PapatheodoridiAndrew J. DingleyMargarita TenopoulouAndrew J. SimkinMargherita CerroneAndrew KesnerMargherita CorrentiAndrew L. GundlachMargherita EufemiAndrew LeaskMargherita G. De BiasiAndrew LowellMargherita IzziAndrew M. WatersMargherita MaioliAndrew MakanyaMargherita NeriAndrew MallettMargherita PuppoAndrew MccaddonMargit BalázsAndrew MillardMargot ZöllerAndrew OzgaMari ChiritoiuAndrew PaluchMaria A. BaturovaAndrew Robert BurkeMaria A. MariggiòAndrew RuysMaria A. RadanovaAndrew Ryan ChinMaria A. VulfAndrew S. DayMaria Addolorata BonifacioAndrew SpiersMaria Adelaide CaligoAndrew SungMaría Agustina BattistoneAndrew ThompsonMaria AkhmanovaAndrew W. TaylorMaria AlbrizioAndrew WestwellMarià AlemanyAndrew WhiteMaria AmbrosioAndrew WillettsMaria Amparo AssisAndrew WoodMaria AndreassiAndrey A. YurchenkoMaría Ángela Burrell BustosAndrey A. ZamyatninMaría Ángela OlivaAndrey B. ShcherbanMaria Angela Zaccarelli-MarinoAndrey ButovchenkoMaria Angeles Caballero-MoraAndrey ElchaninovMaria Angeles RojoAndrey GlotovMaria Angelica MiglinoAndrey KechinMaría Antonia Sánchez-RomeroAndrey LavrovMaria AntoniadouAndrey MarkovMaria Antonietta CiardielloAndrey P. ZhdanovMaria Antonietta PanaroAndrey PlotnikovMaria Antonietta RagusaAndrey R. SuprunMaría Auxiliadora Dea-AyuelaAndrey SarikovMaria Bacci GiacomoAndrey SimbirtsevMaria BagattiniAndrey SinjushinMaria BarbolinaAndrey SolovyevMaria BartosovaAndrey TsaturyanMaria BastianiniAndrey V. BorodaMaria Beatrice MorelliAndrey V. GolovinMaria Beatrice PassaniAndrey V. ZhelankinMaria BenllochAndrey VoshkinMaria Bernabeu AznarAndrey Y. VinokurovMaria BitontiAndrey Zamyatnin, Jr.Maria BogdanAndri Kartasasmita RiauMaria ButakovaAndriana C. KalioraMaría C ArufeAndrii DomanskyiMaría C. Jiménez-MartínezAndrii GrygansyiMaria C. KantereAndrii SlonchakMaria CantonettiAndrii VelychkovychMaria CapovillaAndris DišlersMaría Carmen Díaz-Sala GaleanoAndris ZeltinsMaría Carmen MarquésAndriy KovalenkoMaria Carola FioreAndriy SynytsyaMaria Cecilia Mendonca-TorresAndromachi TzaniMaria ChernyaevaAndroniki KretsovaliMaria Chiara FontanellaAndruta MuresanMaria Chiara GattoAndrzej BajguzMaria Chiara MontiAndrzej BozekMaria ColluAndrzej CendrowskiMaria Cristina BudaniAndrzej K. CiechanowiczMaria Cristina CaroleoAndrzej ŁapińskiMaria Cristina CuriaAndrzej P. HermanMaria Cristina GauzziAndrzej PawlikMaria Cristina PetraliaAndrzej PilcMaría D. CouceAndrzej PlichtaMaria D. MayanAndrzej PolańczykMaria Da Graça P. Morgado S. NevesAndrzej SemczukMaria DapkeviciusAndrzej SlominskiMaria De FalcoAndrzej SzutowiczMaria De La Paz SánchezAndrzej ŻakMaria De Los Reyes Gámez-BelmonteAndrzej ZiębaMaría De Lourdes Garza-RodríguezAndy M.C. LauMaría De Lourdes Moreno AmadorAndy Po-Yi TsaiMaria Del BenAndy T. Y. LauMaría Del Carmen López De Las HazasAndy Wai Kan YeungMaría Del Carmen Vargas-GarcíaAndy Wei-Ge ChenMaría Del Mar Ramírez FernándezAndy-Mark ThunnissenMaría Del Pilar CordovillaAndzhela BulanovaMaria Del Refugio Castañeda-ChavezAnelia HorvathMaría Delgado EstebanAneliya MilanovaMaría Delmans Flores-ChávezAnell OlivosMaria DimarogonaAnella SavianoMaría Dolores Llobat BordesAneta AleksovaMaria Dolores MauricioAneta BalcerczykMaria Dolores Rivero-PérezAneta CierzniakMaría Dolores Robustillo FuentesAneta Cymbaluk-PloskaMaria Dolores Vázquez-CarreteroAneta Cymbaluk-PłoskaMaría Elena Padín-IruegasAneta LorekMaria Elena PisanuAneta Ostróżka-CieślikMaria Eleni SakavitsiAneta PopovaMaría Elisa MilanesioAneta RogalskaMaria Elizabeth TiritanAneta SłodekMaria Emilia BrassescoAneta Szudy-SzczyrekMaria Esther RodriguezAneta SzymańskaMaría Eugenia Jaramillo FloresAneta TarczewskaMaria ExindariAnette Gjörloff-WingrenMaría F. MontenegroAng LiMaria FazzariAnge Mouithys-MickaladMaria Fe Lanfranco GallofreAngel AlvarezMaria Fernanda PessoaAngel Cuesta MartínezMaría Florencia GallelliAngel Darío González-DelgadoMaria Francesca CardoneAngel E. LozanoMaria Francesca PiazzaAngel GalabovMaria Francesca SfondriniAngel GonzalezMaria FranziniÁngel HernándezMaria Gabriella GabrielliAngel J. MatillaMaría GalánAngel L. OrtegaMaria GavriilakiÁngel LlamasMaria GerakariÁngel Luis García-OtínMaria Giovanna CilibertiÁngel MantecaMaria Giovanna RiparbelliAngel MurcianoMaria Giovanna ScioliAngel NuñezMaria Giuseppina MianoAngel Romero ColladoMaría GonzálezAngel Vizoso-VázquezMaría González-Del PozoAngela AbruzzoMaria GoulielmakiAngela BisioMaria GrammatikopoulouAngela Bruna MaffioneMaria Grazia AmmendoliaAngela BrunettiMaria Grazia BottoneAngela CapocefaloMaria Grazia CagettiAngela Carrió-SeguíMaria Grazia CattaneoAngela CastoldiMaria Grazia Di CertoAngela CesaroMaria Grazia MolaAngela CorvinoMaria Grazia RomanelliAngela Dalia RicciMaria Grazia SignorelloAngela Di SommaMaria Grazia ZizzoAngela EliaMaria GreabuÁngela Fontán-LozanoMaria HarjaAngela MccallMaria Helena CasimiroAngela MeccarielloMaria Helena FernandesAngela MessinaMaría Hernández-SánchezAngela MontoneMaría Iniesta CuerdaAngela OttoMaría Iniesta-CuerdaAngela Palumbo PiccionelloMaría Isabel P. BehrensAngela PennacchioMaría Isabel RodríguezAngela Quispe-SalcedoMaría Isabel Torres LópezAngela RizzoMaria IskraÁngela RojasMaría J. PeralAngela RosenbohmMaría Jesús Alvarez CuberoAngela SousaMaría Jesús HerreroÂngela Toshie Araki YamamotoMaría Jesús PeriagoAngela TramontiMaria Joana PintoAngelantonio MinafraMaria João FerreiraAngela-Patricia HernándezMaria João MenesesAngeli EurydiceMaria João MorenoAngelica GiulianiMaria João NunesAngélica María Sabogal-GuáquetaMaria João RamalhoAngelica PernaMaria João RamalhosaAngelica Plata-RuedaMaria Joao SaraivaAngélica Saraí Jiménez-OsorioMaría José Bautista-LlamasAngélica Villarruel-LópezMaria Jose BelliniAngelika Baranowska-ŁączkowskaMaría José BendekAngelika ChroniMaria José Da Silva FernandesAngelika KmitaMaria Jose De Jesus ValleAngelika Tkaczyk-WlizłoMaria Jose FerrandizAngelika V. TimofeevaMaria José Gómez-TorresAngeliki AngelidiMaria José Gonzalo PascualAngeliki BrouzgouMaria José HernaizAngeliki ChroniMaria José Pérez AlvarezAngeliki KatsafadouMaria KantereAngelina Lo GiudiceMaria KhrenovaAngelina NunziataMaria KomelkovaAngeline RouersMaria KoufakiAngélique SourMaria L. MateusAngelo BellinviaMaria L. PerepechaevaAngelo CastelloMaria L. SemenovaAngelo FacchianoMaria LagarkovaAngelo G. TorrenteMaria Laura ErminiAngelo GismondiMaria Laura IddaAngelo MonguzziMaria LavdanitiAngelo PalmigianoMaria LebedevaAngelos SikalidisMaria LeporeAngharad Vernon-RobertsMaria LinderAngus YuMaria Lisa GaravagliaAnh D. VuMaría Llorián-SalvadorAnh Tung PhamMaria LomovaAniello FrancescoMaria LoriteAniello MaieseMaría Lourdes Alarcón FortepianiAnife AhmedovaMaria LucaAnik YuestiMaria Lucia Valeria De ChiaraAnika ToorieMaria Ludovica SaccàAnikó BozsikMaria Luigia VommaroAniko GorbeMaría Luisa CastellóAnil ChekuriMaría Luisa VizueteAnil DhawanMaria Luiza S. MelloAnil KumarMaría Luna-LunaAnil Kumar JonnalagaddaMaría Luz CouceAnil Kumar PatelMaria Luz Rodriguez-MendezAnil Kumar SinghMaría M. Morales Suárez-VarelaAnil Kumar YadavMaria MaaresAnil SinghMaria Maddalena MarrapodiAnil TharappelMaría Magdalena Garijo ToledoAnil VermaMaria MaistoAnila IqbalMaria Manuela GasparAnilkumar GopalakrishnapillaiMaria MarcheseAnimesh PanMaria Margarida AntunesAnimesh SamantaMaría Margarita Canales-MartínezAnindit MukherjeeMaria MarinescuAnindya GangulyMaria MartinsAnindya Sundar RayMaria MazzoniAnirban ChakrabortyMaria MeniniAniruddha DasMaria MenshakovaAnirudh SrivastavaMaria MeringoloAnis M. LimamiMaria Miklasińska-MajdanikAnis ShahMaria MonticelliAnish MukherjeeMaría Moreno-Del ÁlamoAnisha D’SouzaMaria MycielskaAnisoara CimpeanMaria N. BalatskayaAnita BoelenMaria NarożnaAnita Emmerstorfer-AugustinMaría Negrete-GarcíaAnita FranczakMaria NikolovaAnita HaegiMaria OuzounovaAnita KovácsMaria P. RubtsovaAnita Lyssek-BorońMaria PalmieriAnita MikolajczykMaria PanagopoulouAnita MikołajczykMaria Paola BertuccioAnita Roth-NebelsickMaria PapadakiAnja KarlstaedtMaria PaparoupaAnja Kathrin JaehneMaria Pardo-FiguerezAnja Kathrin WegeMaría Paz Villegas PérezAnja KolaričMaría PérezAnja Krieger-LiszkayMaria Pia FelliAnja MottokMaria Pia FerrazAnja R. A. PalmansMaria Pia RiccioAnja SaalbachMaria PieriAnja SchmidtMaría Piñeiro-RamilAnja WilmesMaria PoptsovaAnjali GeethadeviMaria PratAnjali KusumbeMaria Rayane Correia De OliveiraAnjanapura V. RaghuMaria ReifAnjaparavanda NarenMaria Rita BiancoAnju KumariMaria Rita CiteroniAnke OsterlohMaria Rita FabbriziAnket SharmaMaria RoccaAnkica SekovanićMaria RodriguezAnkit ChhodaMaria RohmAnkit GaneshpurkarMaria Rosaria AcocellaAnkit GangradeMaria Rosaria De FalcoAnkit ManglaMaria Rosaria De MiglioAnkit RochaniMaria Rosaria RicciardiAnkit SabharwalMaria Rosaria RuggieroAnkit ShroffMaria Rosaria RuscianoAnkit SrivastavaMaria Rosaria VarìAnkita BurmanMaria RouliaAnkita J. SachlaMaria RussoAnkita PunethaMaria S MuntyanAnkona BanerjeeMaría S. ÁlvarezAnkur KaulMaria Salomé GomesAnkur SoodMaria Salomé PaisAnn B. MoserMaria SandoviciAnn C. FoleyMaria Saveria Gilardini MontaniAnn G. MatthysseMaria ShadrinaAnn HorsburghMaria SimonenkoAnn LiebertMaria SiniaginaAnn SimpsonMária ŠkrabišováAnna A. KulminskayaMaria SkrzyszowskaAnna AjdukMaria SpletterAnna ArteseMaria StangouAnna AspesiMaria Stefania SpagnuoloAnna AtlanteMaria StrianeseAnna AureliMaria SummaAnna AviñóMaria SundvallAnna BaldisserottoMaria SvedaAnna BaranMária SzekeresAnna BaraniakMaria TalmonAnna BarbaroMaria Teresa FiorenzaAnna BarileMaría Teresa Soto-NavarreteAnna BastrzykMaria Teresa VietriAnna Bednarska-CzerwińskaMaria TsiarliAnna BelcarzMaria V. VedunovaAnna BielenicaMaria Valeria CataniAnna Bilska-KosMaria ValievaAnna Bilska-WilkoszMaríA Verónica BaezAnna BizońMaria Villa SerranoAnna BlazewiczMaria Virginia SoldovieriAnna BrancatoMaría Virumbrales-MuñozAnna BrzeckaMaria ViscomiAnna BurdzińskaMaría VitaleAnna C. HearpsMaria Vittoria BaroneAnna CalarcoMaria Vittoria CubellisAnna CapozziMaria Wieteska-MiłekAnna CariboniMaria Yu KonoshenkoAnna CarranoMaria Zavala-CernaAnna CieślińskaMaria ZverevaAnna Cleta CroceMariacristina FiliceAnna ColellMariadhas Valan ArasuAnna Czajkowska-KośnikMariadoss Arokia Vijaya AnandAnna CzarnaMariaelena CaboniAnna CzarneckaMariaelena RepiciAnna D. SerefkoMaría-Esperanza CerdánAnna Di FioreMariafrancesca CascioneAnna Di MatteoMariafrancesca ScaliseAnna Di NardoMariagrazia RossiAnna Di SalleMaríajesús Rodríguez-YoldiAnna Di SessaMaría-José ArgenteAnna Drelich-ZbrojaMaria-Jose TrucoAnna DrzazgaMariam GaidAnna Duda-MadejMariamelia StanzioneAnna EiringMarian BresticAnna ElkinaMarian BurduceaAnna ErmundMarian ButuAnna EspositoMarian JaskułaAnna Esteve-CodinaMarian SzebenyiAnna Fajdek-BiedaMariana B. SpesiaAnna FialováMariana BusilaAnna FilipekMariana CamargoAnna GałązkaMariana CastroAnna GocMariana CristeaAnna GolebiewskaMariana E. GhicaAnna GrendaMariana FioriAnna Grzywa-CelińskaMariana FloriaAnna GullerMariana MarquesAnna GumieniczekMariana RochaAnna Helena MazurekMariana SantosAnna Jakubska-BusseMariana SponchiadoAnna Jamroz-Wis̈NiewskaMariana VarnaAnna JankowskaMariana-Atena PoianaAnna JanowiczMariangela Di DonatoAnna K. WalduckMariangela ManciniAnna Kablak-ZiembickaMariann HarangiAnna KałużaMarianna A. PatrauchanAnna Karin Larsson-CallerfeltMarianna CarinciAnna KarnkowskaMarianna E WeenerAnna Kasielska-TrojanMarianna FabiAnna KasprzykMarianna Flora TomaselloAnna Katarzyna WronskaMarianna KapetanouAnna KazachenkoMarianna MazzaAnna KicińskaMarianna RanieriAnna KitaevaMarianna SantonastasoAnna KleczkaMarianna TriantouAnna KlinkeMarianne BurbageAnna KołtonMarianne ChapleauAnna KorzekwaMarianne KollerAnna Kostecka-GugalaMariano Andres Loza-CollAnna KozłowskaMariano Francesco CaratozzoloAnna Krzywonos-ZawadzkaMariano García-ArranzAnna KulikMariano LagunaAnna KurenkovaMariano OstuniAnna La TeanaMariantonietta Di StefanoAnna LewinskaMaria-Paola ParonettoAnna LinkiewiczMariarita De FeliceAnna Lisa IorioMariarosaria ConteAnna LokshinMariarosaria De PascaliAnna LopataMariarosaria IncoronatoAnna Lucia TorneselloMariasole PerroneAnna M. EhlersMariateresa RossiAnna M. MastrangeloMariavittoria VerrilloAnna MadejskaMaribel Forero-CastroAnna Makuch-KockaMaribel Ovando-MartínezAnna MalashichevaMaribel Plascencia-JatomeaAnna MarabottiMarica MeroniAnna Maria BassiMaricarmen Recillas-MotaAnna Maria Birkl-TöglhoferMaricel PodioAnna Maria FrustaciMaricica PacurariAnna Maria NuzzoMaricica StoicaAnna Maria PirasMaricla GalettiAnna Merwid-LądMarie BipfubusaAnna MeyfourMarie C BénéAnna MichalczykMarie DavídkováAnna MichnoMarie Hélène David CordonnierAnna MichopoulouMarie HronkováAnna Mietelska-PorowskaMarie SjögrenAnna MilczarekMarie Thérèse VanierAnna MolesMarieann HögmanAnna N. BerlinaMarie-Christine JonesAnna NegroniMarie-Christine Kiefer-MeyerAnna NordenströmMarie-Christine OnutaAnna NowinskaMarie-Christine SimonAnna OgrodowczykMarie-Joelle VirolleAnna OrlovaMariel DonzeauAnna OrłowskaMarie‑Lise MaddeleinAnna PaioliMariella D’AngiòAnna Palko-LabuzMariella Della ChiesaAnna PanekMariella DonoAnna PannaccioneMarie-Pierre GolinelliAnna Paola CarrecaMariia AndrianovaAnna Paradowska-StolarzMariia ZhukovaAnna PartykaMarija Gavrovic-JankulovicAnna ParusMarija GredicAnna PawełczykMarija MarkovićAnna PawlikMarija MazorAnna Pérez-BosqueMarija MihajlovicAnna PetruczynikMarija MilanovićAnna PiaseckaMarija StojadinovicAnna PieniazekMarija TakicAnna PietrzakMarijana CurcicAnna PilutinMarijana PetkovićAnna PittalugaMarijana PonjavicAnna Pokryszko-DraganMarijana StojanovicAnna Renata DembskaMarijo BuzukAnna Rita BiliaMarika ComegnaAnna Rita BizzarriMarika CordaroAnna Rita MigliaccioMarika LanzaAnna RonowskaMarika MassaroAnna RoschkeMariko FujisawaAnna RysiakMariko Hara-ChikumaAnna SansoneMariko OkamotoAnna SantarsieroMariko SaitoAnna SaponeMarilena Antunes-RicardoAnna SebestyénMarilena CarboneAnna SerefkoMarilena RonzanAnna ShcheglovaMarilia BarrecaAnna ShchennikovaMarilisa LeoneAnna ShmakovaMarin KovačićAnna SiemiątkowskaMarin MicutzAnna SkwierawskaMarin ŞenilăAnna SobiepanekMarin UgrinaAnna SpagnolettaMarina AunapuuAnna SpallettiMarina BouchéAnna Sroka-BartnickaMarina C. CostaAnna Stanislawska-SachadynMarina CaldaraAnna StasiakMarina CampioneAnna StefanskaMarina DonovaAnna StepienMarina FericAnna SzakielMarina FilimonovaAnna Szczerba-TurekMarina GomzikovaAnna Tabęcka-ŁonczyńskaMarina GottikhAnna TangMarina HolyavkaAnna Tankiewicz-KwedloMarina I. GarinAnna TevyashovaMarina IoveneAnna TrubetskayaMarina K. IbragimovaAnna Turska-SzewczukMarina KarpenkoAnna TutusausMarina KhodanovichAnna V. KlepikovaMarina KrjuchkovaAnna V. TsepokinaMarina LambertiAnna V. TsyganovaMarina MariniAnna Valeria SamarelliMarina PadkinaAnna VaskuMarina PisanoAnna VologzhaninaMarina PiscopoAnna WalczewskaMarina PriscoAnna WawrzyńskaMarina RoginskayaAnna WilkaniecMarina Ruxandra OteleaAnna Winiarska-MieczanMarina S. DukhinovaAnna WojciechowskaMarina SagudAnna WołowiczMarina SantaellaAnna WrzosekMarina Santiago FrancoAnna WysokińskaMarina SelionovaAnna Y. AksenovaMarina ShavelkinaAnna Zalewska-JanowskaMarina SofiaAnna ŻołnierczykMarina Šprem GoldštajnAnna_Antsiferova AntsiferovaMarina Stamenković-RadakAnnabel BlasiMarina Svetec MiklenićAnnabel S. BerthonMarina V BaidakovaAnnabella PignataroMarina V. DunaevaAnna-Irini KoukkouMarina Villanueva-PazAnnalisa BarbieriMarina VukojeAnnalisa BernareggiMarina Zaric KonticAnnalisa ChiavaroliMarina ZoccolaAnnalisa Di RuscioMarinela BostanAnnalisa GiandaliaMarino VilovićAnnalisa MarcuzziMarinos KallikourdisAnnalisa PaceMario A. PagnottaAnnalisa PaoloneMario AbateAnnalisa PecoraroMario Alberto Burgos-AcevesAnnalisa PinsinoMario AriattiAnnalisa SantucciMario C. De TullioAnnamaria AprileMario CaggianoAnna-Maria BarciszewskaMario CaranteAnnamaria LocascioMario CioceAnnamaria ManciniMario D' AcuntoAnnamária PallagMario D'AcuntoAnnamaria PratelliMario DiazAnnamaria ZsakaiMárió GajdácsAnnapina RussoMário Gomes-PereiraAnnarica CalcabriniMario H. BarrosAnnarita NappiMario LaganovićAnnarita StringaroMario Luca MorieriAnnat RaiterMario MilicevicAnne AndersonMario OlleroAnne BernhardtMario PaganoAnne BlaisMario Pedraza-ReyesAnne BlanchardMario Perez SayansAnne BonnieuMario PlebaniAnne FasslMario RaspantiAnne GellerMario RendaAnne HaleniusMario RothbauerAnne Harduin-LepersMário S. RodriguesAnne ImbertyMario SalmeriAnne J. AndersonMário SousaAnne Karine LagendijkMario U. MondelliAnne Lise FerraraMario ValentinoAnne M. WilsonMario Valerio Velasco-GarciaAnne Skaja RobinsonMario Vincenzo RussoAnne VirsolvyMario ZorattiAnneka JoachimsthalerMario ZuritaAnneke HorstmanMariola DutkiewiczAnne-Laure Chateigner-BoutinMariola HerbetAnnemarie E. LaumaeaMariola KozłowskaAnne-Marie GalowMariola M. BłaszczykAnnemarie WentzelMariola NapiórkowskaAnne-Sophie WoznyMariola Sliwinska-MossonAnnette AudigéMariola SłowińskaAnnette BrandtMariola StaniakAnnette KaiserMariola Świderek-MatysiakAnnette KuehnMarion EhrichAnnette MartinMarion HutinelAnnette NassuthMarion LaudetteAnnia GalanoMariona RiusAnnibale PucaMarios AgelopoulosAnnibale VersariMarios G. KrokidisAnnick DuboisMāris KlavinsAnnie Frelet-BarrandMarisa GranatoAnnie JoubertMarisa MadridAnnika LindblomMarisa ManzanoAnnika MohrMarisa P. SárriaAnnika ÖhrfeltMarisa ShiinaAnnina Salome VischerMarisel Romina TuttobeneAnnkristin HeineMarisela VélezAnnunziata MauroMarish OerlemansAnoop AlexMarissa Di PietroAnoop ArunagiriMarit Nilsen-HamiltonAnoop KumarMaritza N. Puray-ChavezAnoop RawatMarius BumbacAnselmo MoriscotMarius Eugen CiureaAnsgar PoetschMarius PrelipceanuAnshika GoenkaMarius ZahanAnshika JainMariusz Aleksander BromkeAnshu AlokMariusz BidzińskiAnshumali MittalMariusz DylagAnshuman MishraMariusz HartmanAnssi PeuronenMariusz KlenckiAntal RockenbauerMariusz KusztalAnte IvankovićMariusz MadejAnthi KarnaouriMariusz MichalczykAnthony BoscoMariusz MitorajAnthony Burgos-RoblesMariusz MojzychAnthony ClarkeMariusz SacharczukAnthony HaighMariusz SikoraAnthony J. BaucumMariusz StasiolekAnthony J. BerdisMariusz Tomasz DziadasAnthony J. FischerMariya A. KuznetsovaAnthony M. C. BrownMariya IvanovskaAnthony MathironMariya V. SukhanovaAnthony MillarMariya VorobyevaAnthony MossMariyana AtanasovaAnthony MukwayaMarja Rytkönen-NissinenAnthony Percival-SmithMarjan MohammadiAnthony SadlerMarjan SabbaghianAnthony William ColemanMarjatta RaudaskoskiAnthony ZulliMarjorie G. ZaudererAnthoula GaigneauxMarjorie JonesAntimo CutoneMarjorie PickAntionette L. WilliamsMark Andrew WhiteAntje Büttner-TeleagăMárk AntalAntje DietrichMark BirrellAntje GroscheMark BuzzaAntoine A. F. De VriesMark ClemensAntoine CaillonMark CooperAntoine MartinMark CraggAntoine MartinezMark D. BakerAntoine NaemMark E. DumontAntón Barreiro IglesiasMark Edmund SmithAnton E. ShikovMark ForsythAnton GáplovskýMárk FráterAnton GoncharovMark G HoogendijkAnton HuberMark G. MoloneyAnton KiselevMark G. WaughAnton KorbutMark HanudelAnton KováčikMark JennessAnton KutikhinMark KrystalAnton ManakhovMark L. WeissAnton P. TyurinMark LawsAnton PetcherskiMark S. BorjaAnton PopovMark S. CraggAnton PospelovMark S. ScherAnton S. TsybkoMark S. TaylorAnton TyurinMark SuterAnton V. DolzhenkoMark T. KozlowskiAnton ZubrikMark TitusAntonella AccardoMark W HankinsAntonella ArgentieroMark W. BeattyAntonella CaccamoMark WoodfordAntonella CaniniMark WunderlichAntonella CaputoMark ZiemannAntonella D' AnneoMarkéta PernisováAntonella FerranteMarko GerićAntonella FrassanitoMarko KumrićAntonella GuzzonMarko LehtonenAntonella LisiMarko LucijanićAntonella ManniniMarko MilerAntonella MessoreMarko R. CincovićAntonella NaiMarko S. MarkovAntonella PaladinoMarko SamardžijaAntonella Ragnini-WilsonMarko UsajAntonella RosiMarkos PetousisAntonella RussoMarkus BergerAntonella ScorzielloMarkus BlaessAntonella TomassettiMarkus DiefenhardtAntonello E SpinelliMarkus EitleAntonello SantiniMarkus FendtAntonet SvircevMarkus GahleitnerAntoni BanaśMarkus KrämerAntoni C. MitusMarkus KrebsAntoni MorralMarkus LinhartAntoni WiedlochaMarkus MandlAntonia CianciulliMarkus NeuditschkoAntonia FollenziMarkus OppelAntonia KondouMarkus RäsänenAntonia M. Jiménez-MonrealMarkus RiesslandAntonia MancusoMarkus ScholleAntonia PatrunoMarkus ThammAntonia ResslerMarkus WilhelmiAntonia RibesMarleen AnsemsAntonieta Guerrero-PlataMarlena Gąsior-GłogowskaAntonietta BernardoMarlena GodlewskaAntonietta FrancoMarlena ZyśkAntonietta SantoroMarlene DreuxAntonija Jurak BegonjaMarlies GijsAntonina ArgoMarloes DekkerAntonina LavrentievaMarlon Henrique E. Silva CardosoAntonina SidotiMarlon Moscoso-MartínezAntonina SorokanMaros SoldanAntonino AlessiMarques OrianaAntonino GermanaMars G SharapovAntonino MorabitoMarsha PierceAntonino NatalelloMarta AdamiakAntonino PassanitiMarta Alegret JordaAntonino TestaMarta Andres-MachAntonino TuttolomondoMarta ArczewskaAntonio AndresMarta BalaskoAntonio BaldiniMarta CarvalhoAntonio BellastellaMarta De AngelisAntonio BernadMarta De ZottiAntonio BlancoMarta Denel-BobrowskaAntonio BrunoMarta Diaz-DelcastilloAntonio Campos-CaroMarta Dziedzicka-WasylewskaAntonio CapezzaMarta Elena Losa IglesiasAntonio CarrieriMarta F EstradaAntonio CaruzMarta FerraroniAntonio Casado-DíazMarta FijałkowskaAntonio CelliniMarta GaburjákováAntonio Chaves-SanjuanMarta García-MontojoAntonio CicioneMarta García-RomeraAntonio Costa De OliveiraMarta GerasymchukAntonio Del VecchioMarta GietlerAntonio Di StasiMarta GomarascaAntonio Di StefanoMarta Gutiérrez-RodrñiguezAntonio EvidenteMarta HabanovaAntonio FacciorussoMarta KarazniewiczAntonio FernandezMarta KepinskaAntonio FerranteMarta KiezunAntonio FortunatoMarta KoblowskaAntonio G. SolimandoMarta KopańskaAntonio GaballoMarta KotAntonio GarciaMarta KsiazczykAntonio Gilson Barbosa De LimaMarta KwapiszAntonio GiordanoMarta LaranjoAntonio Giovanni SolimandoMarta LemieszekAntonio GiovinoMarta LlansolaAntonio González-BulnesMarta LualdiAntonio GrecoMarta MarchettiAntonio J. HerreraMarta MarmiroliAntonio José GuillotMarta Marszalek-GrabskaAntonio LacquanitiMarta MartínezAntonio LaghezzaMarta MascaraqueAntonio LaxMarta MenegazziAntonio LeoMarta Miotke-WasilczykAntonio Lopez-BeltranMarta Montes ResanoAntonio LucacchiniMarta MorenoAntonio M. Pico AlfonsoMarta MoskotAntonio MaccioMarta MusiołAntonio MacciòMarta NardiniAntonio ManentiMarta NevesAntonio MassaMarta Nowacka-ChmielewskaAntonio MassaroMarta Obara-MichlewskaAntonio MauceriMarta OleszekAntonio Mendes-SousaMarta OpalińskaAntonio MichelucciMarta OsrodekAntonio MolinaMarta PadovanAntónio MonteiroMarta PardoAntonio Olivas-MartinezMarta PlanasAntonio OrlacchioMarta Reguera-GomezAntonio PalazzoMarta RieraAntonio Palumbo PiccionelloMarta Ruíz-OrtegaAntónio PauloMarta RusekAntonio PlacidoMarta RybskaAntonio PortolesMarta S. CarvalhoAntonio PortugalMarta SarkozyAntonio Quintero-RamosMarta Sofía ValeroAntonio RaffoneMarta SuárezAntonio RapacciuoloMarta Szandruk-BenderAntonio ReboredaMarta SzekalskaAntonio RecchiutiMarta ToczekAntonio Rodriguez-MorenoMarta TomczykAntonio RosatoMarta Tsirigotis-ManieckaAntonio RossiMarta Vázquez-GómezAntonio Sanchez-AmatMarta Vazquez-VilarAntonio SantagostiniMarta WanarskaAntonio Sarria-SantameraMarta WłodarczykAntónio Sebastião RodriguesMarta ZacconeAntonio SerranoMarta Żebrowska-NawrockaAntonio Simone LaganàMarta ZolaAntonio StarčevićMarten John EdwardsAntonio TeixeiraMarten TrendelenburgAntonio TiberiniMartha Alejandra Morales-SánchezAntonio TrovatoMartha Gabriela Gaxiola-CortesAntonio Vidal-PuigMartha Patricia Gallegos-ArreolaAntonio ZandonaMartha Rocío Moreno-JimenezAntonios GiannopoulosMartha S. SandyAntonios M. MakrisMarthe J. HowardAntonius (Tom) OomensMartin A. EstermannAntony AlbertMartin BaborAntony AnanthMartin BakerAntony Ceasar Stanislaus AntonyMartin BakhtAntony KamMartin BartasAntony MemboeufMartin BirkettAntreas KantarosMartin BrezaAntt Htet WaiMartin ČernýAntu DasMartin ChanAnu RahalMartin CheungAnubama RajanMartin Christian MichelAnugya BhattMartin DorfAnuj AhujaMartin E. BlohmAnuj KumarMartin EberhardtAnuj RanjanMartin FellnerAnuja PatilMartin GoslingAnujit SarkarMartin HammerAnukana BhattacharjeeMartin HollandAnúp PandíțhMartin HugAnup ParchureMartin JaburekAnup S. PathaniaMartin JanickoAnupam DhasmanaMartin Jozef PéčAnupam MukherjeeMartin KelloAnupriya K HaridasMartin Krøyer RasmussenAnuraag BoddupalliMartin L. DuennwaldAnuradha BhukelMartin MajerníkAnurag MisraMartin MaronekAnurag PassiMartin MassányiAnusak KerdsinMartin MollenhauerAnusha AdityaMartin MoradAnushka GuptaMartin P. PlayfordAnushree AcharyaMartin PaparellaAnuska V. AndjelkovicMartin PecAnvesh DasariMartin PéčAnwar A. SayedMartin PešlAnwar UsmanMartin PeterAnying SongMartin PootAo ShiMartin PrusinkiewiczAphrodite TsaballaMartin PsenickaApichart AtipairinMartin RasporApoena RibeiroMartin RootApolonia SieprawskaMartin RuthardtApostolos ApostolakisMartin S StaegeApostolos Georgiou PappasMartin S. PavelkaApostolos I. TsolakisMartin SalzmannApostolos IoakeimidisMartin SchepelmannApostolos PapachristosMartin SchlaepferApostolos ZaravinosMartin SchmidtAppu RathinaveluMartin Schmidt-HieberApurba Krishna BhattacharjeeMartin StimpfelAraci Maria Da Rocha RondonMartin SztachoArafat Abdel Hamed Abdel LatefMartin WittArancha Llama-PalaciosMartin ZachariasArantxa AceraMartin ZiegerArantza InfanteMartina AlvarezAránzazu Zarzuelo CastañedaMartina AulittoArash RamedaniMartina B. BöttnerArati SridharanMartina BancirovaAravind JukantiMartina BaraldoAravindan JayaramanMartina BerettaAravinthkumar JayabalanMartina BonaciniArbind PrasadMartina ChiuArcady MarkelMartina De SienaArcady PutilovMartina FaraldiArcangelo LisoMartina GabrielliArchana MishraMartina GálikováArchana PrabaharMartina HrastArchita Venugopal MenonMartina KirchnerArdiansyah TaufikMartina KleberAreeful HaqueMartina MaggiAref Abbasi MoudMartina PfefferArgha MondalMartina PianoArghya GhoshMartina ŘezáčováArghya RayMartina RossiArgiris KolokithasMartina SchmidtAri HashimotoMartina Srajer GajdosikAri HietalaMartina TorricelliAri KoivistoMartina VrankićAri MeersonMartina ZíkováAri SchafferMartine Jandrot-PerrusAria BaniahmadMartine PrévostAriadne CruzMartyna Ewa Maszota-ZieleniakAriana GattMaruti UppalapatiArianna BellucciMarwa A. A. FayedArianna De MoriMarwa HanafiArianna LatiniMarwa MatboliArianna PalladiniMarwa TallawiArianna ServiliMarwan KwokArianna VigniniMary Ann SteppArie B. VaandragerMary B. GoldringAriela BenigniMary BeilbyArif RobinMary Beth Browning MonroeAriharan ArjunanMary BoardArijit NathMary C. MajArijita SarkarMary G. GarryArin MarchesiMary J MeeganArina PonomarenkoMary J. Van SchooneveldArindam BhattacharjeeMary K. CrowArindam Ghosh MazumderMary KavurmaArinder K. AroraMary L KraftArio De MarcoMary LawsAris TsalouchosMary M. Y. WayeArisa UekiMary Ni LochlainnAristeidis ChiotellisMary SevignyAristidis GaliatsatosMaryam ArdalanAristidis TsatsakisMaryam AzlanAristotelis LymberopoulosMaryam Borhani-HaghighiAristotle G. KoutsiarisMaryam HajiabbasArjan VissinkMaryam MoazeniArjun AdhikariMaryam Mobed-MiremadiArjun Prasad TiwariMaryam MoshrefArjun SapkotaMaryam Mozafarian MeimandiArjun SenguptaMaryam N. Al-NasserArkadij SobolevMaryam TahvildariArkadiusz ArtyszakMary-Lorène GoddardArkadiusz DziedzicMaryna StasevychArkadiusz LubasMarzena BiałekArkadiusz MatwijczukMarzena LaskowskaArkadiusz MiazekMarzena MackowiakArkaitz MucientesMarzena Mogielnicka-BrzozowskaArkasubhra GhoshMarzena ParzymiesArleta DrozdMarzena Sujkowska-RybkowskaArmand AbergelMarzena WarchołArmanda E. SantosMarzena WątekArmanda RodriguesMarzena Włodarczyk-StasiakArmando A. Rayas-AmorMarzena WójcikArmando Andres Roca SuarezMarzia CottiniArmando CardosoMarzia OgnibeneArmando CaseiroMarzia VasarriArmando CocaMarzieh SalimiArmando NegriMarziliano NicolaArmando PatrizioMaša Skelin KlemenArmando Pérez TorresMaša ŽdralevićArmando SenaMasaaki TamuraArmando Varela-RamirezMasaaki WachiArmandodoriano BiancoMasabumi ShibuyaArmenio BarbosaMasafumi HorieArmin KrausMasafumi NodaArmin MešićMasafumi TanakaArmond DaciMasafumi YohdaArnab BhattacharjeeMasahiko YamaguchiArnab NayakMasahiro ChataniArnau HerveraMasahiro FujimuroArnaud AlvesMasahiro FunahashiArnaud BianchiMasahiro HitomiArnaud BondonMasahiro IkedaArnaud DelobelMasahiro ImamuraArnaud GissotMasahiro IwamotoArnaud MailleuxMasahiro NakashimaArnd BaumannMasahiro NiikuraArnóbio Antonio Da Silva JúniorMasahiro NishiharaArnold BolomskyMasahiro OjimaArnoldo Aquino-GálvezMasahiro YamasakiArnon HennMasahiro YoshikawaArnon KarniMasahito ShimojoArokia Mariyadoss VijayaanandMasahito YamagataAron WellerMasakazu G. IwaizumiArooma MaryamMasakazu SugishimaArpád DobolyiMasakazu TanakaÁrpád DobolyiMasaki FujitaÁrpád Ferenc KovácsMasaki HataArpad KarsaiMasaki NagayaArpad Mihai RostasMasaki OkamotoÁrpád SzállásiMasaki UenoÁrpád SzékelyMasakiyo YatomiÁrpád TósakiMasako TodaArpita ChatterjeeMasami NozakiArpita KulkarniMasami YoshimuraArpna KumariMasamichi IshiaiArrigo CiceroMasamichi KamihiraArsalan AhmedMasamichi NishiguchiArsen S. MikaelyanMasamitsu KanadaArseniy E. YuzhalinMasanobu MinoArseniy YuzhalinMasanori FujiiArshad AlhasnawiMasanori HashimotoArshad JalalMasanori KoideArshi KhanamMasanori KuriharaArtem BadasyanMasanori Masanori HijiokaArtem D. RogachevMasanori MurakamiArtem G. LadaMasanori SaitoArtem MetlinMasanori YoneyamaArtem S. KasianovMasao OtaArtem Yu ManyakhinMasao YamaguchiArthur I. NovikovMasaoki KohzakiArthur LustigMasaomi YamaneArthur MerkelMasaru MatsuiArthur P. ArnoldMasaru TanakaArthur P. GrollmanMasashi FujitaniArthur ShapiroMasashi KawamiArtur BanachMasashi KurodaArtur Filipe RodriguesMasashi MukohdaArtur GalimovMasashi ToyodaArtur GiełdońMasatake AsanoArtur KhannanovMasato MaesakoArtur KowalikMasatoshi OhnishiArtur MayerhoferMasatoshi YamaguchiArtur MuszyńskiMasayasu KuwaharaArtur PałaszMasayuki FujitaArtur RatkiewiczMasayuki HashimotoArtur RibeiroMasayuki SatakeArtur SłomkaMasayuki TakahashiArtur Tadeusz KrzyżakMasayuki WakiokaArtur TurekMasfueh RazaliArtūras ŽalgaMasindi MphaphathiArturo BevilacquaMasmudur M. RahmanArturo Edgar Zenteno-GalindoMasoud Ahmadi-AfzadiArturo Flores-PliegoMasoud RahmatiArturo HernandezMasoume AmirkhaniArturo RomanoMasoumeh Kourosh AramiArul VeerappanMassimiliano AmmirabileArulkumar NagappanMassimiliano BianchiArulsamy KulandaisamyMassimiliano CadamuroArumugam SangiliMassimiliano ChettaArun ButreddyMassimiliano CordaroArun Kumar Hanumana Gouda PatilMassimiliano CorsoArun Kumar RajendranMassimiliano EspositoArun P. WiitaMassimiliano GaetaArun Prabhu RameshbabuMassimiliano GalluzziArun RajasekaranMassimiliano PetriniArun SendilnathanMassimiliano RenziArun ShankarMassimiliano RunfolaArun UpadhyayMassimiliano RuscicaArunaksharan NarayanankuttyMassimiliano ScalvenziArunas RamanaviciusMassimiliano ValentiniArunava GhoshMassimo BergerArunava RoyMassimo BertaminiArunika DasMassimo BracciArutselvan NatarajanMassimo BrogginiArvin Haj-MirzaianMassimo CapocciaArvind GulbakeMassimo CarossaArvind KumarMassimo CocchiArvind Kumar ShuklaMassimo ColettaArvind MisraMassimo D'ArchivioArvind NegiMassimo Di GiulioArvind NuneMassimo E. MaffeiArwa ShahinMassimo GanassiAsad JanMassimo GazzanoAsadur RahmanMassimo IorizzoAsaf BilgoryMassimo La RosaAsaf MarcoMassimo LibraAsanka Sajini YapaMassimo ManginoAseem ChawlaMassimo MariottiAshaq AliMassimo NabissiAshay D. BhatwadekarMassimo ReconditiAshenafi F. BeyiMassimo RomaniAshfaq A. MarghoobMassimo TorreggianiAshfaq AhmadMassimo TrottaAshfaqur RehmanMassimo TusconiAshish AgrawalMassimo ValotiAshish JainMassimo ZevianiAshish KabraMasumi SuzuiAshish NoronhaMatan ShelomiAshish SrivastavaMatas JakubauskasAshkan BighamMateescu CarmenAshkan RashadMatej VosnjakAshkan ZandiMateja GermAshley J SniderMateja J. PrimožičAshley L. PitzerMater MahnashiAshok ChaudharyMateus J. SudanoAshok IyaswamyMateus Webba Da SilvaAshok KumarMateu-Sanz MiguelAshok PatowaryMateusz BujkoAshok YadavMateusz DulskiAshot SargsyanMateusz FicekAshraf Al-BrakatiMateusz GliwińskiAshraf ElsayedMateusz KowalikAshraf KhalifaMateusz ŁucAshraf M. OmarMateusz PiętAshraf N. AbdallaMateusz SpałekAshraf VirmaniMateusz WaliczekAshraful Islam MollaMatheus PolettoAshu JohriMathew AlexanderAshutosh KumarMathew BloomfieldAshutosh P. DubeyMathias MontenarhAshutosh PandeyMathias TrempAshutosh RaiMathieu Di MiceliAshutosh TripathiMathieu EdelyAshwani KumarMathieu QuinodozAshwini ChauhanMathieu SeppeyAshwini MalladMathilde Body-MalapelAsia Fernandez CarvajalMathilde MunierAsif AliMatias MosqueiraAsif RazaMatias SoiferAsim Bikas DasMatija RijavecAsim Kumar BepariMatjaž HladnikAsiya MustafinaMatjaž KopačAsker KhapchaevMatjaž KristlAskin BirgulMats W. JohanssonAsko UriMats-Olof MattssonAsli YilmazMatsuda KazunoriAsma AyazMatt KipperAsma MaqboolMatt LewinAsma RessaissiMatt MarlowAsmerom SengalMatteo BonomoAspinas ChapwanyaMatteo BordiAssaf A. GiladMatteo BulatiAssunta BertacciniMatteo CastronovoAssunta PozzuoliMatteo CeccarelliAssunta SellittoMatteo ChiappediAsta ZubrieneMatteo FabbriAstrid BrullMatteo FerroAstrid ObermayerMatteo GiovarelliAstrid Paulitsch-FuchsMatteo GuidottiAstrid WeißMatteo LenerAstrida VelenaMatteo MoriAsuman GedikbasiMatteo NioiAtashi SharmaMatteo PaciniAtaul IslamMatteo PavoniAtefeh SoloukMatteo RiccòAthanasia MouzakiMatteo RipaAthanasia PapazafiropoulouMatteo RipamontiAthanasia VarvaresouMatteo SaccoAthanasios AlexandrisMatteo SorgeAthanasios KatsourasMatthew BeardAthanasios KoliogiorgosMatthew BeneschAthanasios MilionisMatthew BrichacekAthanasios PapakyriakouMatthew BrodyAthanasios SalifoglouMatthew ButlerAthanasios TiliakosMatthew D BensonAthar AnsariMatthew D. BergAtheer ZgairMatthew D. StachlerAtiah H. AlmalkiMatthew DayAtish MukherjiMatthew E. R. ButchbachAtiyeh M AbdallahMatthew ForsbergAtoany Nazareth Fierro RadillaMatthew GageAtrouli ChatterjeeMatthew James BranchAtsuki NaraMatthew L. FaronAtsunori FukuharaMatthew L. FergusonAtsushi B. TsujiMatthew McmillinAtsushi HijikataMatthew P. PadulaAtsushi HosuiMatthew PetersonAtsushi SuzukiMatthew StrubAtsushi YamanakaMatthew T. WolfAtsushi YamashitaMatthew YousefzadehAtte Von WrightMatthias BegemannAttila AszodiMatthias BrosAttila BecskeiMatthias CarlAttila BokorMatthias David ErlacherAttila BoricsMatthias EckhardtAttila BraunMatthias GaestelAttila Egri-NagyMatthias GeyerAttila FábiánMatthias GimpelAttila FarkasMatthias GoebelerAttila G. VéghMatthias HahnAttila Gábor SzöllősiMatthias KapplerAttila Gergely VéghMatthias LampingAttila JakabMatthias NeesAttila KovácsMatthias NéesAttila L. ÁdámMatthias PlöchlAttila OláhMatthias StopeAttila TothMatthias WeiderAttila VányolósMatthias WilmAttila ZsarnovszkyMatthieu Pierre PlatreAttila ZsolnaiMatthieu VignesAttilio AnzanoMattia BartoliAttiq Ur RehmanMattia Di NunzioAtukuri DorababuMattia Di PaoloAtul DhallMattia Falchetto OstiAtul SharmaMattia MorandiAude Magérus-ChatinetMattia MoriAude Sophie NassifMattia QuattrocelliAudi Francesca SetiadiMattia ToniAudic YannMatúš ČomaAugust WrotekMatus DurdikAugustas PivoriunasMatus SykoraAugustin M. OfiteruMaud FriedenAugustin Mouinga-OndéméMaulik VyasAurel MaximMaulin PatelAurel Popa-WagnerMaura LodoviciAurelia Cristina NechiforMaura Téllez-TéllezAurelia VisaMaureen H. V. FernandesAurelian Cristian BoscorneaMauricio A RetamalAurélie LedreuxMauricio BustosAurélie PetitMauricio Comas-GarciaAurélie PhilippeMauricio Homem-De-MelloAurélie SchoubbenMauricio L. CafieroAurélien DeniaudMaurício Temotheo Temotheo TavaresAurelio Luna MaldonadoMaurine NeimanAurelio ScavoMaurizio AlimandiAurora ArghirMaurizio BadianiAurora SavinoMaurizio BattinoAurora StorlazziMaurizio CrestaniAurora VassanelliMaurizio DelvecchioAurore CarréMaurizio EliaAurore Claude-TaupinMaurizio Enea PicarellaAustin L. LagrowMaurizio ForteAvathamsa AthirasalaMaurizio G. AbrignaniAvi DombMaurizio MargaglioneAvi PrielMaurizio MartiniAvijit PramanikMaurizio MulasAvik DuttaMaurizio MuracaAvinash Chandra PandeyMaurizio PaciAvinash KadamMaurizio PascadopoliAvinash KumarMaurizio PolanoAvinash MishraMaurizio RemelliAvinash PremrajMaurizio RomanoAvinash SinghMaurizio SabbatiniAvisek MajumderMaurizio TonettiAvishay ElisMauro BelliAvneesh KumarMauro CancianAvo KarusMauro CeccantiAwdhesh Kumar MishraMauro CentrittoAwdhesh MishraMauro ColucciaAxel FreischmidtMauro FinicelliAxel H. SchönthalMauro FrecceroAxel KatzMauro GiuffrèAxel KünstnerMauro MandrioliAxel PagenstecherMauro MontalbanoAxel PramannMauro SalviAxel SchlagenhaufMauro Sergio Ferreira SantosAxel SteigerMauro ViganòAyae Sugawara-NarutakiMautusi MitraAyako SakamotoMawieh A HamadAyalew Ligaba-OsenaMaw-Sheng LeeAyan DattaMax HübnerAyat RashaidMax PiffouxAyataka FujimotoMaxim A. GureevAyaz ShahidMaxim IvanovAydin Bordbar-KhiabaniMaxim L. BychkovAyelén ToroMaxim M. KorshunovAyhan HoruzMaxim RudminAykut Arif TopcuMaxim Stanislavovich MakarenkoAylin M. DeliormanliMaxim V. BerezovskiAyman El-AliMaxim V. MusalovAyman ElsamanoudyMaxime CahuzacAyman MahmoudMaxime PichonAyman W El-HattabMaxime RobinAymara MasMaximilian SeidlAyrat U. ZiganshinMaximilian ZeydaAyse AyhanMaximiliano Luis CacicedoAysegül AksanMaya DesaiAysel OzpinarMaya DymovaAyslan Castro BrantMaya M. ZaharievaAytan MusayevaMaya RubtsovaAyuob AghanejadMaya SchuldinerAyush RanawadeMayank ChoubeyAyyalusamy RamamoorthyMayanka AwasthiAyyanar SivananthamMayo TsuboiAzat BilalovMayra PaolilloAzat GabdulkhakovMaytham HusseinAzat TipeevMayumi KomineAzeddin El BarnossiMayumi NishiAzeem UllahMayur DokeAzhar RasulMayur K. LadumorAziz AmineMayur ParmarAziz BachaMayur VirarkarAziz KarakayaMayura VeeranaAziz KhanMayya RazgonovaAzizollah KhormaliMazahar MoinAzra ÐulovićMazen AlmehmadiAzza MostafaMazen SalehAzza NaikMazhar HussainAzzurra StefanucciMazharul IslamB Ten HakenMd Abdul HakimB. L. NgcoboMd AdnanBabak BakhshinejadMd Ahasan HabibBabak E SaraviMd Ashik UllahBabak EslamiMd Jahoor AlamBabak KhoramianMd Jamal UddinBabak PakbinMd Kausar RazaBabalwa JackMd Khadem AliBabar UsmanMd Mustafizur RahmanBabhrubahan RoyMd Muzammel HossainBabita Kumari VermaMd Najib AlamBabur Z. ChowdhryMd Saiful IslamBach Quang LeMd Shahariar ChowdhuryBahaa YoussifMd Soriful IslamBahai JiaoMd Tahjib-Ul-ArifBahar PatlarMd-Zahid AkhterBahareh AzimiMeaghan HancockBahman AnvariMeco GiuseppeBahram SalehMeena KumariBaiba K. GillardMeenakshi AhluwaliaBaiba SvalbeMeenakshi S. KagdaBaiquan LiuMeera G. NairBaisong LuMeerambika MishraBaji ShaikMegan BeetchBal Krishna ChaubeMegan L. FalsettaBal L. LokeshwarMeghna GuptaBala Krishna PrabhalaMegumi HonjoBalachandar VellingiriMehdi AlikhaniBalachandran ManavalanMehdi BamorovatBalajee RamachandranMehdi KabaniBalaji NagarajanMehdi Mogharabi-ManzariBalakrishnan KirubasankarMehdi MontazerBalamurali Krishna VasamsettiMehdi RezaeiBalamurugan RamatchandirinMehdi SafdarianBalapal BasavarajappaMehdi Younesi-HamzekhanluBalasubrahmanyam AddepalliMehmet Akif BozBalawant KumarMehmet Enes ArslanBalazs FeherMehmet GülBalázs MargitMehmet GültasBalik DzhambazovMehmet GürsoyBálint NagyMehmet Ilyas CosacakBálint TamaskovicsMehmet M AltintasBanzeer Ahsan AbbasiMehmet SarierBao HongliangMehmet SenelBao JiaMehmet YamanBaoan SongMehmet YilmazBaohua ChenMehra HaghiBaohua XuMehran GhaemiBaojian ZhuMehrdad KhatamiBaomin DaiMehri HadinezhadBaomin WangMehtap P. KaraBaoming LiuMei HongBaosheng ZengMei ZhaoBaoyu ChenMeichen PanBapan PramanikMei-Chin MongBapi PaharMeicun YaoBappaditya ChandraMeifeng XuBaptiste DemeyMeihui TianBaptiste MailleMei-Jin GuoBaran ErenMeik NeufurthBarbara BlasiMei-Ling YangBarbara BorczakMeiping ZhangBarbara BuffoliMeirong XuBarbara BuldiniMeisam TabatabaeiBarbara CelliniMeitian XiaoBarbara DarišMeixue DongBarbara De FilippisMeiyan WangBarbara Di StefanoMei-Zhen CuiBarbara DziegielewskaMekala VenkatachalamBarbara E. RolfeMel VaughanBárbara FerreiraMelánia BabincováBarbara GhinassiMelanie A. EshelmanBarbara GierlikowskaMelanie B. GillinghamBarbara GierobaMelanie CoombsBarbara HajdukMelanie FoeckingBarbara IlliMelanie KorbeliusBarbara JachimskaMelanie KüspertBarbara JagoszMelanie MärklinBarbara JanaMelanie Ricke-HochBarbara Kloeckener-GruissemMelanie RobitailleBarbara Krochmal MarczakMelanie S. MatosBarbara Kutryb-Zaja̧CMelanie WinkleBarbara LiederMeleksen AkinBarbara LipinskaMelinda FogarasiBarbara LipińskaMelinda HerbathBarbara ŁotockaMelinda SoeungBarbara MalawskaMelinda SzilágyiBarbara MarengoMelissa A. St HilaireBarbara MavroidiMelissa E. StaufferBarbara MontaniniMelissa García-CaballeroBarbara MulloyMelissa H. WongBarbara PanunziMelissa IlardoBarbara PawłowskaMelissa J. WalkerBarbara PieczykolanMelissa Lee WilsonBarbara PipanMelissa Michelle CadelisBarbara PucelikMelissa RaynerBarbara RossiMelissa RichardBarbara Ruszkowska-CiastekMelissa S TottenBarbara SawickaMelissa VosBarbara ScelsaMelody ClarkBarbara Shukitt-HaleMeltem Ezgi DurgunBarbara SkerlavajMelvin E. KlegermanBarbara SotteroMelvin HaydenBarbara SymanowiczMelvin OliverBarbara Szeiffova BacovaMelvin Y. RinconBarbara Van AschMemoona RamzanBarbara WhitmanMenake PiyasenaBarbara WiewióraMeng LinBarbara WirleitnerMeng WangBarbara ZaapalaMeng WuBarbara ZdzisińskaMeng ZhangBarbara ZiółkowskaMeng ZhuBarend Hj De GraafMengcheng ShenBarend W. FlorijnMengfei LiBargel HendrikMenghsiao MengBaris UzildayMenghsiung HsiehBarry HofferMenglong XuBarry MilavetzMengmeng HuangBarry PaulMengmi ZhangBarry R. PalmerMengqi HuangBarry RosenMengquan YangBarry WuMeng-Tsan ChiangBart De GeestMengxi ShenBart JanssenMengyang FengBartlomiej BarczynskiMenno Van Der VoortBartłomiej GórskiMepur RavindranathBartłomiej GrygorcewiczMercè Pallàs LliberiaBartłomiej KostMercedes FernandezBartlomiej KrazinskiMercedes Garcia-GilBartłomiej PochwatMercedes Jiménez-RosadoBartlomiej TroczkaMercedes SerranoBartłomiej ZapotocznyMerlin NanayakkaraBartolome SabaterMert VuralBartolomé SabaterMervin YoderBartosz Adam FryczMeta SternišaBartosz BojarskiMetis HasipekBartosz GurzędaMeysam KeshavarzBartosz MałkiewiczMezanur RahmanBartosz PawlińskiMhairi MorrisBartosz TrzaskowskiMi Kyung ParkBaruh PolisMi LiBasem A. AhmedMia HorowitzBashayer R. Al-MubarakMia Madel AlfajaroBasheer MannasahebMian Abdur Rehman ArifBashir LawalMian HuangBashir UddinMian NavedBasil MathewMiao ChuBasini GiuseppinaMiao FeiBaskar VenkidasamyMiao YuBaskaran PalanivelMiaojin ZhouBaskaran Stephen InbarajMiaw-Ling ChenBassam AbomoelakMicael GonçalvesBassma ElwakilMicael WiderströmBastiaan J. BraamsMichael A. FirerBastian PopperMichael A. JacksonBastian SchirmerMichael A. ShestopalovBayodé Roméo AdégbitèMichael Anton BauerBeat VögeliMichael AppellBeata Borowiak-SobkowiakMichael ArandBeata Butruk-RaszejaMichael ArkasBeata DworakowskaMichael ArulBeata GabryśMichael B. FischerBeata Greb-MarkiewiczMichael BanfBeata Hukowska-SzematowiczMichael BertocchiBeata KaletaMichael BlankeBeata Kalska-SzostkoMichael BlumerBeata M. Gruber–BzuraMichael Bording-JorgensenBeata Majkowska-MarzecMichael BoschmannBeata ModzelewskaMichael C SeedsBeata Mrozikiewicz-RakowskaMichael C. BeverlyBeata NaumczukMichael CalcuttBeata NowakMichael CampbellBeata OlejnickaMichael DanilenkoBeata PajakMichael DaviesBeata Sarecka-HujarMichael DietrichBeáta SperlaghMichael EllisonBeata SzeflerMichael F. HynesBeáta SzolnokiMichael FasulloBeata Wielgus-KutrowskaMichael FleischhackerBeate Brand-SaberiMichael G. WellerBeate RinnerMichael GareljaBéatrice C. DurandMichael GelbBeatrice DufrusineMichael GelinskyBeatrice FerrariMichael GöbelBeatrice KnudsenMichael GoodsonBeatrice MacchiMichael HausmannBeatrice PyMichael HenselBeatrice ScazzocchioMichael J. DuryeeBeatriz Benitez-TemiñoMichael J. EmanueleBeatriz Buentello-VolanteMichael J. FranklinBeatriz Candás-EstébanezMichael J. StoutBeatriz Ester García-GómezMichael J. ThomsonBeatriz LimaMichael J. YoungBeatriz MerinoMichael Jacob IoelovichBeatriz Millan-MaloMichael Jordan RowleyBeatriz QuiñonesMichael KirbergerBeatriz Sabater-MuñozMichael KohlerBeatriz Salinas RodríguezMichael KravchikBeatriz Sánchez-ParraMichael KubeBeatriz San-MiguelMichael LeitgesBebiana CondeMichael Leonidas ChikindasBecky TalynMichael LevinBee K TanMichael LichtenauerBee Kang TanMichael LiebmanBeen-Ren LinMichael LowBeenu Moza JalaliMichael MaukschBegoña CánovasMichael McmurrayBegoña Renau-MorataMichael MorganBegoña Villar-ChedaMichael N. KammerBegüm OkutanMichael NaumannBehnam GhasemzadehMichael OglesbeeBehzad M. ToosiMichael P. KrahnBeing-Sun WungMichael P. MarkeyBéla IvánMichael P. SchmidtBéla KajtárMichael PoteserBela KocsisMichael PuschBéla Nagy, Jr.Michael R. CragerBela PappMichael R. LadomeryBéla PappMichael R. PaillasseBelaghihalli N. GnaneshMichael RedingBelém Sampaio-MarquesMichael RisnerBelén AltavaMichael RoseBelen BeginesMichael RothBelén Callejón LeblicMichael RubartBelkhiri LotfiMichael RützlerBello MartinianoMichael S. ReidBen C. L. ChanMichael SandstromBen De Lacy CostelloMichael SanguinettiBen MeinenMichael SchirmerBenazir SiddeekMichael SchmittBence RaczMichael SchweizerBenedict B. BenignoMichael SehornBenedikt LinderMichael SergeevBenesh JosephMichael ShermanBeniamin Oskar GrabarekMichael StrugBenito A YardMichael SwansonBenjamí Oller-SalviaMichael TellierBenjamin AnsaMichael TsangBenjamin BenzonMichael V. UgrumovBenjamín Cartes-SaavedraMichael Van DykeBenjamin Chidi OnyeaguchaMichael VogelBenjamin ClarksonMichael W. BeckBenjamin D. BrooksMichael W. HessBenjamin DelpratMichael WeichhausBenjamin E. TurkMichael WempeBenjamin GantenbeinMichael YarusBenjamin J. WylieMichael YoussefBenjamin KendzioraMichaela A. BotiBenjamin KochMichaela BosákováBenjamin L. KingMichaela FiliouBenjamin L. WoolbrightMichaela ŠiklováBenjamin LacroixMichaela WenzelBenjamin LindseyMichail KlontzasBenjamín PinedaMichail OrfanoudakisBenjamin R. CaroneMichailidis MichailBenjamin RocheMichal AlexovičBenjamin RollesMichał BiernackiBenjamin VerretMichał BijakBennett D. ElzeyMichał BurdukiewiczBenno WeigmannMichal CagalinecBenny RyplidaMichal CeglowskiBenoît BizimunguMichal DabrowskiBenoit DenizotMichał GondekBenoit DesnuesMichal J. DabrowskiBenoit GauthierMichał J. SobkowiakBenoît GuillotMichal JesetaBenoît HeinrichMichal JurášekBenoît MermazMichał K. ZarobkiewiczBenoit MiottoMichal KidawaBenson OtarighoMichał KolasaBenye LiuMichał KolińskiBerenice PalaciosMichal KorostynskiBerenika M. Szczȩśniak-SiȩgaMichał KulusBergonci TábataMichał LatalskiBeril AltunMichał MączewskiBernadeta ChyrchelMichal NiemczakBernadeta SzewczykMichał NowickiBernadette Emoke TelekyMichał OczkowskiBernadette KálmánMichal OrdakBernadette M. HenaresMichał OtrębaBernadette M.M. ZwaansMichał P. WasilewiczBernard HaendlerMichał PikułaBernard LeungMichal PravenecBernard NaafsMichal PyzikBernard PossidenteMichal ŘezankaBernard Pui Lam LeungMichal S. KarbownikBernard RoelenMichal S. NowakBernard Van Den BergMichal SarulBernardo Brito PalmaMichal ShteinbergBernardo ChavesMichal SlanýBernardo Gonçalves AlmeidaMichal SmahelBernat CrosasMichal ŠmídaBernd FritzschMichal SobkowskiBernd GahrMichał Stefan LachBernd HelmsMichal SzeremetaBernd KuhnMichal WozniakBernd RodemannMichał ZałȩckiBernd SchöllhornMichał ZarobkiewiczBernd StratmannMichalak-Tomczyk MagdalenaBernd WissingerMichał-Goran StanišićBernhard BiersackMichalis LiontosBernhard GibbsMichel DionBernhard H.F. WeberMichel NieuwoudtBernhard Michael PolzerMichel PfefferBernhard MichalkeMichel PicardoBernhard RauchMichel RavelonandroBernhard SchreflerMichel VignesBernhard ZdárskyMichela BattistelliBert BroneMichela CampoloBert CallewaertMichela CastagnaBert FoquetMichela FerrucciBerta Cillero-PastorMichela GabelloniBerthold HocherMichela IannoneBert-Jan H. Van Den BornMichela NotarangeloBertold MarienMichela PozzobonBertold RennerMichela PuglieseBertrand BlondeauMichela RigoniBertrand C. W. TannerMichela RossiBertrand KaefferMichele BernasconiBertrand LiagreMichele CastelliBertrand TchanaMichele CiullaBerwin Singh Swami VethaMichele Cosimo SantoroBesma Sghaier-HammamiMichele Crozatier-BordeBeth BragdonMichele D'AngeloBeth FuchsMichele De BortoliBeth M. ClevelandMichele Di CosolaBethany GrayMichele FaralliBetina BiagettiMichele FioreBettina BlaumeiserMichele FornaroBettina WilmMichele Francesco Maria SciaccaBetty Revon LiuMichele GraciottiBeverly MockMichele KlainBhaben ChowardharaMichele LaiBhagat NeetaMichele LoewenBhagavathi SundaramMichele MalaguarneraBhagwat NawadeMichele ManfraBhagwati GuptaMichele MazzantiBhagyashree JoshiMichele PerniBhagyashri BurguteMichele PinelliBhagyesh SarodeMichele RussoBhaja Krushna PadhiMichele SalemiBhakti BasuMichele SamajaBhama RamkhelawonMichele SavianoBhanu TewariMichele VisentinBharani Krishna Mynampati ArunadithyaMichel-Edwar MickaelBharat H. JoshiMichelle AfkhamiBharat MadanMichelle Swanson-MungersonBharat MishraMichelle TallquistBharath Kumar VilluriMichelle VincendeauBharathi DevarajMichelle Whirl CarrilloBharathi Raja RamadossMichelle YooBharati NeelamrajuMichiel Van EijkBhaskar BiswasMichihiko UsuiBhasker RadaramMichio KomaiBhaswati BhattacharyaMichiyo SuzukiBhaswati SenguptaMickael BlaiseBhavani NatarajanMicol FalabellaBhavna MuraliMidturu SrinivasBhawik Kumar JainMieczyslaw ScendoBhoj GautamMieszko WieckiewiczBhola PradhanMieszko WięckiewiczBhoomika MathurMiguel A Garcia-GonzalezBhoomika PatelMiguel A OrtegaBhupendra KoulMiguel A. OrtegaBhupinder SinghMiguel A. PrietoBhuvana Shyam DoddapaneniMiguel AlcarazBhuvanesh Sukhlal KalalMiguel Alejandro Lopez-RamirezBi MaMiguel Angel Alcántara-OrtigozaBi ShuguangMiguel Ángel Hernández-OñateBi WuMiguel Ángel LujánBiagio BaroneMiguel Ángel Martínez‐TéllezBiagio DidonaMiguel Ángel Mendoza-CatalánBiagio PincheraMiguel Ángel MerchánBiagio RaponeMiguel Angel RamirezBiagio SantellaMiguel Ángel Rodríguez-MillaBianca ColonnaMiguel Ángel Sogorb SánchezBianca E. ŐszMiguel Ángel Torralba-CabezaBianca GalateanuMiguel Armengot-CarcellerBianca Maria TihăuanMiguel ArroyoBianca Maria ZaniMiguel De VegaBianca R. AlbuquerqueMiguel EstravísBianca SchneiderMiguel F SeguraBianca SchrulMiguel Flores-BellverBianca VezzaniMiguel Gisbert-GarzaránBianchi MéndezMiguel HuesoBibekananda KarMiguel IdoateBibha ChoudharyMiguel Jesus Nunes RamosBibhisan RoyMiguel LaraBibi KhanMiguel Mazorra-ManzanoBibiane Steinecker-FrohnwieserMiguel MouratoBice ContiMiguel Navarro-AlarconBidisha MitraMiguel OlivasBidisha RoyMiguel Perez-AmadorBihua NieMiguel Ponce-VargasBijay BeheraMiguel Rebollo-HernanzBijayalaxmi MohantyMiguel RibeiroBijender KumarMiguel Tavares PereiraBiji Theyilamannil KurienMiguel TeixeiraBiju ThomasMiguel Vicente-ManzanaresBilal AftabMiguel VidalBilal RasoolMiguel ViñasBilge Guvenc TunaMiha MrazBiljana BalenMihaela BacalumBiljana BujanovicMihaela Balan-PorcarasuBiljana Đ. GlišićMihaela CosnitaBiljana M. BojovićMihaela GhitaBiljana VučkovićMihaela HostiucBill DennyMihaela IlieBill J. TawilMihaela NiculaeBilqees BanoMihaela TurtoiBimal GhimireMihaela Violeta GhicaBimal JanaMihaescu GrigoreBimal KoiralaMihai CenariuBimal Kumar GhimireMihai NitulescuBin JiangMihai OaneBin LinMihai OlteanBin LiuMihai Stefan MuresanBin MaMihai TibuBin WuMihail D. CroitoruBindu SubhadraMihail Lucian BirsaBing YangMihail Virgil BoldeanuBing ZhouMihajlo RisteskiBing ZhuMihalj PošaBingchun ZhaoMihaly MezeiBingde ZhengMi-Hee KwackBingsen ZhangMihir ShettyBingyun SunMihnea ZdrengheaBinoy MaitiMiikka-Juhani HonkaBinsen LiMiing-Tiem YongBinu Kandathilparambil SasiMijat BožovićBinu PrakashMi-Jung KwonBirgit LeitingerMika SakamotoBirgit M. PruessMikako FujitaBirgit ScharfMike Dyall-SmithBirgitt SchüleMikel García-PugaBirgitte OlsenMikhail DivashukBirthe B. KragelundMikhail DrobizhevBiruk A. FeyissaMikhail FirsovBiserka RelicMikhail G. AkimovBishnubrata PatraMikhail KislinBishoy Y.A. El-AaragMikhail KotovBissera PilichevaMikhail M. KostikBiswadeep DasMikhail M. Vorob'EvBiswajit BhattacharyyaMikhail N. BerlovBiswajit PadhyMikhail Nikolaevich LyulyukinBiswajit PramanickMikhail Oliveira LeastroBiswanath JanaMikhail PetrovBiswarup SahaMikhail RyazantsevBjoern BauerMikhail ShepelevBjørn Arild HattelandMikhail ShestakovBjörn DahlbäckMikhail V. DubininBjörn TampeMikhail V. KhvostovBjørnar HasselMikhail V. ShubaBjörn-Philipp DiercksMikhail V. VenerBlaine MooersMikhail VysokikhBlanca CárdabaMikhail Y. InyushinBlanca Hernández-LedesmaMikhail Y. SyromyatnikovBlanca Ocampo-GarcíaMiki KushimaBlandine DavailMiki RahatBlanka Szekely-SzentmiklosiMikihisa UmeharaBlas Lotina-HennsenMikihito KajiyaBlaž LikozarMikio HayashiBlaž StresMikio TanabeBłażej GrodnerMikko O. LaukkanenBłażej KudłakMiklos KerteszBlazej RubisMiklós KubinyiBłażej RuszczyckiMiklós MézesBlessing Atim AderibigbeMiklós NagyBlythe D. ShepardMiklós PoórBo DongMikó ÉvaBo GaoMikołaj DziurzyńskiBo HuMikyoung ParkBo Hyon YunMila GugnoniBo JiangMila RadanBo LiMilad HadidiBo LiuMilad MoloudizargariBo PangMilad RasouliBo Ram BeckMilagros MedinaBo WangMilan ČížBo XueMilan JakubekBoas PuckerMilan Kumar LalBoaz BarakMilan SkalickýBoaz G. OliveiraMilan TomaBoaz MoharMilan UrbanBoaz NachmiasMilana KulakovaBob HowellMilardi DaniloBob LauderMilena A. VegaBobin ChenMilena Álvarez-ViñasBobirca FlorinMilena CavicBo-Cheng TangMilena GeorgievaBodhisattwa BanerjeeMilena IwaszkoBogdan A. SavaMilena RizzoBogdan Cătălin ȘerbanMilena ŚciskalskaBogdan DorofteiMilena SorrentiBogdan IliescuMilena TrajkovićBogdan KazmierczakMili DasBogdan L. LesyngMilica AcimovicBogdan LewczukMilica Atanacković KrstonošićBogdan Marian SorohanMilica BozicBogdan SevastreMilica Miljković-TrailovićBogdan SlencuMiljana KecmanovićBogdan SmykMilorad VujicicBogdan SoceaMilos LazarevicBogdan Stefan VasileMilos PjanicBogdan-Mircea MihaiMiloslava FojtováBoguslawa LuzakMilton GroppoBohan ZhangMilton RoyBohumil FafilekMilva PepiBojan BožićMilvia ChiccaBojan KnapMin ChenBojan MitićMin GuanBojan ZaricMin Heui YooBojana MilutinovicMin HuiBojin BojinovMin Jeong HongBoldeanu Mihail VirgilMin Kyu KangBoleslaw KarwowskiMin ParkBonaccorsi Di Patti Maria CarmelaMin Soon ChoBonhomme LudovicMin WangBon-Jae GuMin XieBonnie L. BalzerMin YaoBono LučićMin Young LeeBoon Chin TanMina RaileanuBoon-Seng WongMinal ThackerBoopathi SubramaniyanMin-Chieh ChuangBor Luen TangMîndra Eugenia BadeaBor-Chyuan SuMindy I. DavisBoris DudicMindy LevineBoris FurtulaMine BekarBoris KablarMine KöktürkBoris MinaevMineko TeraoBoris SkryabinMinerva Codruta BadescuBoris SlobodinMineyoshi AoyamaBoris TenchovMing ChenBoris U. StambukMing ChuBoris VauzeillesMing Der LinBorislav AngelovMing DingBorja Belda-PalazónMing Hsien ChanBorja Herrero De La ParteMing Hsien TsaiBor-Show TzangMing LiBorutaitė VilmantėMing LuoBoryana Nikolova-MladenovaMing MaBorys DzyubaMing QuBorys PrzemyslawMing Shiuan SohBostan NazishMing YangBouayad NoureddinMing ZhangBoxuan ShenMingang HaoBoya LiuMing-Che LiuBoyan GrigorovMingchong YangBoyang LiMingfang QianBoyong KimMing-Fu WangBožena CukrowskaMinghao GongBožena Ćurko-CofekMing-Hon HouBożena JarząbekMing-Hsi ChiangBożena KróliczewskaMing-Hsin YangBożena Pawlikowska-PawlęgaMinghui CaoBożena Romanowska-DixonMing-Ju HsiehBożena TyliszczakMingjun LiuBozhao LiMing-Jun TsaiBozhidar StefanovMingkang SunB̧Ožo KŕušlinMing-Kang TsaiBrach PostonMingku ZhuBrad A. FrekingMing-Kuei ShihBrad BarnettMing-Kuem LinBrad DavidsonMinglei GuoBrahim AissaMingli WangBrahim BouizgarneMinglian WangBrajesh Kumar SinghMingmin LuBrandon HendersonMingming TongBrandon J. BeddingfieldMing-Quan GuoBrandon L. PearsonMingqun LinBrandon LewisMingsheng ChenBrandon Lucke-WoldMing-Shiou JanBrandon Peter Lucke-WoldMingsong KangBranislava TeofilovicMing-Tse KuoBranka Hrvoj-MihicMing-Tsz ChenBranka Šošić-JurjevićMing-Wei LinBrankica FilipicMing-Wei WangBrant G. WangMingxing ChengBratko FilipičMingxing QianBrenda KirklandMingxing XieBrenda M. AlexanderMingxu YouBrett L. HurstMingxun WangBrett M. MitchellMingyao LiuBrian BalogMingyao YingBrian BennettMingyi ChenBrian BirchMing-Yi ShenBrian BothnerMingyi WangBrian CeresaMing-Yii HuangBrian D. AdamsMingze SunBrian J. HaasMingzhuo LiBrian JonesMinhajul ArfeenBrian KayMinhao XieBrian McdonaghMinh-Dung TruongBrian MckayMinhong ShenBrian ShawMinjie ZhangBrian SimsMinjun YangBrian WardMin-Kyu KimBriana K. ShimadaMinodora DobreanuBriana R. De MirandaMinoo RassoulzadeganBrianna D. YundMinoru GotohBrice WilsonMinsu JungBrielle RosaMintu MeenaBrígida Ribeiro PinhoMinyi TianBrigitta TóthMinyong LiBrigitte HantuschMiodrag GužvićBrigitte Le Magueresse-BattistoniMiodrag JanicBrijesh SinghMiquel Armengot-CarbóBrijeshkumar PatelMiquel Torrent-SucarratBrînduşa Alina PetreMir Ajab KhanBrindusa TiperciucMi-Ran ChoiBritani BlackstoneMiranda MeleBritta EggersMiranda SertićBritta KleinMircea NasuiBrock HumphriesMircea TampaBrook L NunnMireia MallandrichBruce A. PfefferMireia TondoBruce CornellMirela CordeaBruna BrandaoMirela Ivančić ŠantekBruna DenizMirela LozićBruna MionMirela MihailaBruna VisniauskasMirela-Fernanda ZaltariovBruno António CardosoMirella Pupo Santos SantosBruno AzzaroneMiren AltunaBruno BaršićMiri KrupkinBruno CerraMiriam HickeyBruno ChrcanovicMiriam ZacchiaBruno D'AgostinoMiriam-Rose MenezesBruno FaveryMirian Graciela Da Silva Stiebbe SalvadoriBruno FiondaMiriana D’AlessandroBruno FonsecaMirja AhmmedBruno GuigasMirjam B. ZeiselBruno GuillotinMirjana JerkicBruno ImbimboMirjana StojkovićBruno Jorge ColaçoMirko H.H. SchmidtBruno KiefferMirko ManchiaBruno MeloniMirko ManettiBruno Paes De MeloMirko MaturiBruno Pereira CrulhasMirko TrillingBruno PontesMirna JovanovicBruno R. B. PiresMirna Perez-MorenoBruno Rafael Pereira LopesMirolyuba IlievaBruno Ramos-MolinaMirona Palczewska-KomsaBruno SterliniMiroslav BarančíkBruno TassoMiroslav GlasaBruno TiribilliMiroslav HýblBruno VilloutreixMiroslav MachalaBrutch NinaMiroslav MensikBryan Andrew KillingerMiroslav N. NenovBryan D. BrysonMiroslav PohankaBryan D. CrawfordMiroslava Dušková-SmrčkováBryan FullerMiroslava El FrayBryan M. WongMiroslava KacaniovaBryan MathisMiroslava NedyalkovaBryan StegelmeierMiroslava RakocevicBryan TroxellMirosław AndrusiewiczBryan WongMiroslaw GalazkaBryn D. MonneryMirosław GiurgBuddhadev LayekMiroslaw JablonskiBudimir MijovićMirosław KwiatkowskiBudsaraporn NgampanyaMirosław RatkiewiczBuǧra KarasuMirosława ChwilBulat RamazanovMiroslawa CichorekBulat SaifutdinovMiroslawa PuskullogluBulent AhishaliMirta MilicBülent ErginMirta MilićBum-Yong KangMirunalini RavichandranBurcu CelebiogluMiryam MullerBurcu GumuscuMirza HasanuzzamanBurghardt WittigMirza PojskicBurkhard FranzMirza PojskićBurkhard MöllerMisa TakahashiBushra ShahidaMisbahuddin M RafeeqBu-Yeo KimMisha PerouanskyBuyun TangMishra JyotsnaByeong-Seo CheongMiso SabovicByeong-Wook SongMitchell PalmerBylgja HilmarsdottirMitesh DwivediByoung Kuk JangMitesh PatelByung Joo KimMithalesh Kumar SinghByung Jun ChoMititelu-Tartau LilianaByung Kil ChooMitja MitrovicByung Ran SoMitra Safavi-NaeiniByung Soo KimMitra Shojania FeizabadiByung-Chang SuhMitsuhiko AsakawaByunghee HanMitsuo JisakaByung-Hoon JeongMitsuru HarukiByungki JangMitsuru IshikawaByung-Soo KooMitsuru NakazawaByungyong AhnMitsuru NenoiC. James ChouMitsuru OhishiC. Jane WelshMiura KenjiC. Leigh BroadhurstMiyo MoritaC. Michael GreenliefMizanur RahamanC. Sieiro SantosMizu JiangCaetano DoreaMk JollyCai RobertsMladen AndjićCaifei ZhangMladen ParadžikCaitlin MitchellMoamen Salah S. RefatCaitlyn BowmanMobashir MohammadCale D. FahrenholtzModestas ŽiliusCălin Gabriel FloareMoema HausenCalin Mircea GhermanMohamad Al-SheikhlyCalla M. GoekeMohamad NavabCalogero CarusoMohamad Nurul IslamCalogero FioricaMohamed A. A. AhmedCalvin Lloyd ColeMohamed A. El-EsawiCalvin Yu Chian ChenMohamed A. HassanCam DonlyMohamed AbdelgawadCamelia CoadaMohamed AbdelgiedCamelia Elena LuchianMohamed AbdelwahabCamelia QuekMohamed Abdullah JaberCamelia Sorina StancuMohamed Adel HammadCamelia UngureanuMohamed AliCamelia Vidita GurbanMohamed Ali BenabderrahimCameron P. BrackenMohamed Amine JmelCamilla Albertina Dantas De LimaMohamed Bechir Ben HamidaCamilla MatassiniMohamed DamejCamille EhreMohamed El-AassarCamille GuillereyMohamed El-MissiryCamille MaloufMohamed ElmonemCamillo PortaMohamed El-SherbinyCamillo RosanoMohamed G. ShehataCamilo López-CristoffaniniMohamed Gamal MohamedCamilo Zamora-LedezmaMohamed H. Abdel-RahmanCan CuiMohamed HassanCan HuangMohamed Mahmoud HassanCan JiangMohamed MohanyCândida Teixeira TomazMohamed O. RadwanCanran WangMohamed Osman RadwanCara GottardiMohamed Raafat El-GewelyCara J. WestmarkMohamed S. NafieCara TimpaniMohamed SaidCarina CruchoMohamed SalahCarina DehnerMohamed SallaCarina Gabriela UasufMohamed ShehataCarine ChassainMohamed SheteiwyCarine MaisseMohamed SolimanCarl FratterMohamed Z. M. SalemCarl Randall HarrellMohamed ZayedCarl TrindleMohamed ZommitiCarla BazzicalupiMohamed ZoughaibCarla Bianca Luena VictorioMohamed-Amine HassaniCarla CannizzaroMohamed-Bassem AshourCarla CicalaMohammad Adel GhiassCarla CruzMohammad Afsar UddinCarla Di StefanoMohammad AlfatahCarla F. SousaMohammad Al-HatamlehCarla FerreriMohammad AnsariCarla FiorentiniMohammad Arif AshrafCarla GentileMohammad ArifuzzamanCarla LopesMohammad Asif SherwaniCarla LuciniMohammad Azam AnsariCarla MasalaMohammad BashashatiCarla MirandaMohammad BayanCarla MucignatMohammad El KhatibCarla PalumboMohammad Fahad UllahCarla PeregoMohammad H. AbukhalilCarla PollastroMohammad H. SemreenCarla RaggiMohammad Hadi SekhavatiCarla S. G. P. QueirósMohammad HassanCarla Sofia Garcia FernandesMohammad Hassan BaigCarla Solé CañadasMohammad Hojjat-FarsangiCarlemi CalitzMohammad Imran KhanCarlo AprileMohammad IrfanCarlo BergaminiMohammad KhalidCarlo BieńkowskiMohammad Mahboob AlamCarlo BredaMohammad Mehdi ArabCarlo CampanaMohammad Mehdi OmmatiCarlo Cosimo CampaMohammad MehdizadeCarlo DiaferiaMohammad Mirazul IslamCarlo Ettore PellicciariMohammad MofradCarlo GalliMohammad MohammadianpanahCarlo GaniniMohammad NajlahCarlo MannoMohammad Nazmul HasanCarlo Maria CarbonaroMohammad OvesCarlo MateraMohammad QneibiCarlo MischiatiMohammad Reza MorshedlooCarlo MorelliMohammad Reza SepandCarlo PerriconeMohammad Rostami-NejadCarlo PozziMohammad SadraeianCarlo RonsiniMohammad SayariCarlo SalernoMohammad Shah JahanCarlo SantambrogioMohammad ShahidCarlo TicconiMohammad Taghi AlebrahimCarlo TrombettaMohammad Tahir SiddiquiCarlo VenturaMohammad Tajul IslamCarlos A. IglesiasMohammad Tarequl IslamCarlos A. M. CarvalhoMohammad Tavakkoli YarakiCarlos A. Martínez-HuitleMohammad ThaljiCarlos Alberto Avila-OrtaMohammad Zahirul IslamCarlos Alejandro Granados-EchegoyenMohammad Zaki AhmadCarlos Alfonso Álvarez-GonzálezMohammad ZamaniCarlos CaroMohammad Zuhaib QayyumCarlos CaulinMohammad ZulkifliCarlos Correa-NettoMohammadali Khan MirzaeiCarlos D. GrasaMohammadhossein DabaghiCarlos David Grande TovarMohammadian MehdiCarlos De NoronhaMohammadjavad MohammadiCarlos Eduardo Da Silva OliveiraMohammadreza ShokouhimehrCarlos EscalanteMohammed EmranCarlos FarinhaMohammed GagaouaCarlos García-EstradaMohammed H. MohammedCarlos GeraldesMohammed HassanCarlos GonzalezMohammed HawashCarlos González-FernándezMohammed Imad MalkiCarlos Guerrero-SanchezMohammed M. AhmedCarlos Gutierrez-MerinoMohammed RhaziCarlos I. ArbizuMohammed ZayedCarlos J CarrascoMohammod AminuzzamanCarlos LoncomanMohan BabuCarlos M. CorreiaMohandoss SonaimuthuCarlos MarcuelloMohd AdnanCarlos Martins GomesMohd AliCarlos MedinaMohd AsgherCarlos Miguel Henriques FerreiraMohd Ezuan Bin KhayatCarlos Miguel MartoMohd Farhanulhakim Mohd Razip WeeCarlos PalmeiraMohd ImranCarlos Pérez-Albacete MartínezMohd Khairuddin Bin Md ArshadCarlos Pérez-MonterMohd Nurazzi NorizanCarlos Perez-PlasenciaMohd SaeedCarlos Perez-StableMohd Salahuddin Mohd BasriCarlos RamosMohd ShahbaazCarlos Rocha-De-LossadaMohd Zulkifli SallehCarlos Rodriguez-NogalesMohd. MuddassirCarlos RosadoMohind C. MohanCarlos RosasMohini GuleriaCarlos SanzMohit ChawlaCarlos TéllezMohit K MidhaCarlos Vicario-AbejónMohmed Isaqali KarobariCarlos VilaMohsen AkbarianCarlos Yesid SotoMohsen ElsharkawyCarlotta PontremoliMohsen HesamiCarme CaellesMohsen KarimiCarmel MothersillMohsen Motie-ShiraziCarmela De SantoMohsen Yoosefzadeh NajafabadiCarmela Dell'AversanaMohsin JafriCarmela Maria MontoneMohsin KhanCarmela PaolilloMohsin MaqboolCarmela RicciardelliMohsina KhanCarmela SantangeloMohtadin HashemiCarmela ZizzoMoises Armides Franco-MolinaCarmelo La RosaMoises Di SanteCarmelo RomeoMoisés Tolentino B. Da SilvaCarmen AvaglianoMoito IijimaCarmen CadillaMojgan AlaeddiniCarmen Cagigas FernándezMojgan RastegarCarmen DinizMojtaba AnsariCarmen DobreaMojtaba MokariCarmen Dora Méndez-HernándezMojtaba MoradiCarmen EspinósMojtaba ZiaeeCarmen GianfraniMok-Ryeon AhnCarmen GutiérrezMolla MengistCarmen HerenciaMolly YaoCarmen López SánchezMomčilo GavrilovCarmen MannellaMominur RahmanCarmen Matás-ParraMona F. A. DawoodCarmen NeamtuMona Hamdi HaronCarmen Nicoleta PurdelMona K. E. QushawyCarmen Ortega-SantosMona M. FreidinCarmen PanaitescuMona SaadeldinCarmen PredaMona SalehCarmen RamírezMonali NandymazumdarCarmen Rojas-MartínezMonder MartaCarmen SantosMong-Heng WangCarmen ScieuzoMonia Di PreteCarmen SolcanMonica AlmeidaCarmen Steluţa CiobanuMonica BucciantiniCarmen TeránMónica BullejosCarmine FinelliMonica ButnariuCarmine GuarinoMonica CanepariCarmine IzzoMonica CiveraCarmine MerolaMonica CurròCarmine OstacoloMonica De CaroliCarmine PiconeMonica FlorescuCarmine StolfiMonica GulatiCarol Chitko-MckownMònica IglesiasCarol ImbrianoMonica Lopes FerreiraCarol MaddoxMónica María Umaña ZamoraCarol WadhamMonica MariniCarole HelisseyMonica MironescuCarole SousaMonica MoniciCarolina Alonso GonzálezMonica MontopoliCarolina Cristina JancicMonica MuratoriCarolina D. SchinkeMonica ProboCarolina DaviesMónica SerranoCarolina DonàMonica Tatiana Parada-SanchezCarolina FerreiraMónica TeixeiraCarolina Francelin RovarottoMónica TéllezCarolina G. PoitevinMonica ValentovicCarolina HagbergMonica Y. LeeCarolina S. VinagreiroMonica YabalCarolina Sánchez-RodríguezMonika BarathovaCarolina YáñezMonika BeszterdaCaroline BorderonMonika BleszynskiCaroline EvansMonika BorkowskaCaroline MüllerMonika CzerwinskaCaroline MysiorekMonika E. RacCaroline N. DealyMonika FajferCaroline Pellet-ManyMonika FijakCaroline RanMonika FurkoCaroline SubraMonika Hułas-StasiakCaroline Weinstein-OppenheimerMonika KatariaCarolyn A. EcelbargerMonika KijewskaCarolyn M. KlingeMonika KniotekCarrie HouseMonika KopećCarrie LongMonika KovačevićCarrie ShawberMonika KoziełCarsten CarlbergMonika KujdowiczCarsten DoscheMonika LesickaCarsten HerskindMonika Les̈KiewiczCarsten PedersenMonika LewinskaCarsten TheissMonika MichaelisCarsten WeissMonika MioduchowskaCarsten ZeilingerMonika PapieżCasey LangdonMonika PawłowskaCasey NestorMonika PituchaCasper SchalkwijkMonika RadlinskaCassandra HarapasMonika Sakowicz- BurkiewiczCassandra L. CliftMonika SkorupaCassandra TerryMonika Śmiga-MatuszowiczCassiano Felippe Gonçalves-De-AlbuquerqueMonika SobocanCassie S. MitchellMonika SzeligaCastro-Junior Célio JoséMónika SzűcsCatalin I. PruncuMonika SzulińskaCătălin MaximMonika Szymańska-CzerwińskaCatalin TiliscanMonika WimmerCatalin TutaMonika WujecCatalin ZahariaMonique Tháıs Costa FonsecaCatalina Natalia Cheaburu YilmazMoniruddoza AshirCatalina Valdés-BaizabalMonirul IslamCatarina CamposMonish MakenaCatarina MarreirosMonish Ram MakenaCatarina RamosMonokesh Kumer SenCatarina S. H. JesusMonsurat LawalCatarina SeabraMontana VedranaCatarina SilvaMontecucco FabrizioCatarina TeixeiraMonther A. KhanfarCatello Di MartinoMontse GuardiolaCaterina FedeMontserrat AgutCaterina Maria GambinoMontserrat TobajasCaterina MorabitoMonwadee WonglapsuwanCaterina PalleriaMood MohanCaterina PeggionMoo-Ho WonCaterina RufoMoon Il KimCaterina TomaMoon Ley TungCatherina BeckerMoon Nyeo ParkCatherine A. ReardonMoon-Sung KangCatherine ClellandMootaz SalmanCatherine CoiraultMor Nadia Lurie-WeinbergerCatherine Colas Des Francs-SmallMoreno DuttoCatherine DavisMorgan BrisseCatherine Faivre-SarrailhMorgan GallazziniCatherine GhoshMorgan HamonCatherine JaureguiMorihito TakitaCatherine MichauxMorris ManolsonCatherine MulliéMortaza KhodaeiaminjanCatherine Vénien-BryanMorteza Hasanzadeh KafshgariCatherine ViguiéMosab KaseemCatherine VilpouxMoses AyoolaCatherine Weikart YeckelMoshe ReuveniCatherine YzydorczykMoshe ShemeshCátia DominguesMossab Y SaeedCatia ScassellatiMostafa AbotalebCatrin TylMostafa Ahmed AbooufCecil StushnoffMostafa BakhtiCécile Baudoin-DehouxMostafa ElachouriCecile Bienboire FrosiniMostafa El-MiligyCécile OuryMostafa GoudaCécile PhilippeMostafa RadyCécile PochonMostafa Yuness Abdelfatah MostafaCécile VignalMotohiro KameiCecilia BattistelliMotohiro NishidaCecilia Beatrice ChighizolaMotohiro TaniCecilia ChevalMotonari KondoCecilia ChiriacMotoshige YasuikeCecilia ColettiMoumita DattaCecilia D’AlessioMoureq AlotaibiCecilia GunnarssonMoustafa GabrCecilia Jiménez-MallebreraMoustafa SarhanCecilia MannironiMoustafa T. MabroukCecilia MariniMoya Anne HayCecilia NunesMoyasar AlhamoCecilia PozziMozaniel OliveiraCecília R.C. CaladoMr Naimi-JamalCecilia SpedalieriMrigya BabutaCecilia XimenezMrinal BhattacharjeeCedric ChaverouxMrinmoy GhoshCédric DelevoyeMrutyunjay PanigrahiCedric LabayMubashir AhmadCedric MoroMubshar HussainCédric PoleselloMudasir Bashir GugjooCeferino Maestú UnturbeMudasir RashidCeleste ManfrediMudassar Iqbal ArainCelestine N. ChiMuhammad A. AlsherbinyCelestino SarduMuhammad A. JawadCelia BoukadidaMuhammad AdnanCélia C. G. SilvaMuhammad Adnan KhanCelia CruzMuhammad Ahsan AsgharCelia Fernández RubioMuhammad AjazCelia GarauMuhammad Ajmal BashirCélia Maria Cássaro StrunzMuhammad Ajmal ShahCelia RavelMuhammad AkhtarCélia SoaresMuhammad Amjad NawazCélia VenturaMuhammad Ashiq FareedCeline CaseysMuhammad AsifCéline KosterMuhammad AslamCelso P. De MeloMuhammad AzamCemil ColakMuhammad Bashir KhanCésar Augusto João Ribeiro Muhammad Bilal Bilal ShakoorCesar Augusto MedinaMuhammad FaheemCesar CeballosMuhammad Faisal SiddiquiĆesar CobaledaMuhammad HumayunCésar Fernández-SánchezMuhammad Ijaz KhanCesar Lopez-CamarilloMuhammad ImranCésar MatteiMuhammad Imran MalikCésar QuirozMuhammad IqhrammullahCesare C. BerraMuhammad Irfan SiddiqueCesare SirtoriMuhammad Junaid RaoCéu CostaMuhammad KamranCevat EriskenMuhammad Kashif ShahidCezar ComanescuMuhammad KhanCezary WojtyłaMuhammad KhurramChad BrabhamMuhammad Massub TehseenChad StahlMuhammad MunirChadin KulsingMuhammad Muzamil KhanChae Moon HongMuhammad NaeemChaeuk ChungMuhammad Naeem SattarChahrazade El AmriMuhammad NawazChaitanya KurhadeMuhammad NazirChaiyaboot AriyachetMuhammad NomanChaiyavat ChaiyasutMuhammad QamarChakraborty SohiniMuhammad RazaChalermpong SaenjumMuhammad Raziq Rahimi KoohChalid AssafMuhammad RiazChamari WijesooriyaMuhammad SajjadChamila GunathilakeMuhammad Shahid NadeemChampa RatnatungaMuhammad Shahzad AslamChanchal MandalMuhammad Shahzad KamalChanchal Sur ChowdhuryMuhammad ShakeelChander Shekhar MukhopadhayayMuhammad ShakirChander Singh DigwalMuhammad ShoaibChandra BoosaniMuhammad Siddique AfridiChandra MadasuMuhammad SohailChandra MishraMuhammad Sohail ZafarChandra Mohan SinghMuhammad SoyfooChandra Sekhar YadavalliMuhammad Tahir AkramChandrabose SelvarajMuhammad Tahir Ul QamarChandrakala Aluganti NarasimhuluMuhammad Tariq JavedChandrasekhar KottakotaMuhammad UllahChandrashekhar VoshavarMuhammad UmairChandru SubramaniMuhammad UmerChang ChenMuhammad Usman MunirChang Dong YeoMuhammad UzairChang Gue SonMuhammad Wahab AmjadChang GuoMuhammad WaseemChang Han LeeMuhammad ZafarChang Hoon LeeMuhammad Zahid MumtazChang KimMuhammad Zulhaziman Mat SallehChang LiMuhammed AtamanalpChang Su LimMuhammed Naseem AkhtarChang Won ChoiMuhammed Shafeekh MuyyarikkandyChang Yeon YuMuhammet KarakavukChang Young JangMuhhamad SajidChang-Han ChenMuhittin KulakChanghong YangMukesh JainChanghui ShenMukesh KumarChang-Hun HuhMukesh Kumar SriwastvaChanghwan AhnMukesh P. YadavChang-Hyu BaeMukund BhandariChang-Hyun KimMukund TantakChanghyun RohMulazim Hussain AsimChangjun DingMulugeta M. ZegeyeChangli ZhaoMumtaz AnwarChang-Liang ChenMumtaz V. RojianiChangling HuMunazza KiranChanglong WenMunekazu KomadaChangmin PengMunenori KitagawaChangmo HwangMuneyoshi OkadaChangquan LingMun-Ho RyuChang-Ro LeeMunir AktasChang-Seok LeeMunkyong PaeChangshin YoonMura Jyostna DeviChangtian PanMurad MollaevChang-Tze YuMurali KoramutlaChang-Wei HsiehMurali MamidiChangwei QiuMurali Mohan AyyanathChangwon YangMurali MunirajuChang-Yi WuMuralidharan ManiChanhui LeeMurat AladağChanin SillapachaiyapornMurat AycanChanpreet SinghMurat BingulChantal A ColesMurat DogruChantal Wicky CollaudMuratani MasafumiChanung WangMuriel AbbaciChan-Yen KuoMuriel ElhaiChao ChenMuriel Le RomancerChao LiangMuriel PriaultChao XieMurni Nazira SarianChao ZhangMurray PattersonChao-Cheng HuangMurtaza KhanChao-Hui YangMurugan VeerapandianChao-Kai HsuMuruganandan ShanmugamChaoling ChenMurugesan ChandrasekaranChaoqun HuangMurugesan VelayuthamChaoqun YaoMusa KavasChao-Yu GuoMustafa Ojonuba JibrinChaozhong ZhangMustafa ShukryChara SimitziMustapha Cherkaoui MalkiCharalampos SiristatidisMustapha NajimiCharan SinghMusuc Adina MagdalenaCharat ThongprayoonMuthaiah ShellaiahCharikleia StefanakiMuthia ElmaCharis L. HimedaMuthu SathishCharis LiapiMuthu ThiruvengadamCharith Raj Adkar-PurushothamaMuthukumar KaruppasamyCharles AllenMuthukumar RamanathanCharles D. RiceMuthuramalingam PandiyanCharles G BaileyMuthusamy MuthusamyCharles HarringtonMuthuvel JayachandranCharles HunterMuttalip GündoğduCharles L. GleenMuyun XuCharles LochenieMuzamil Yaqub WantCharles M MacveanMya Myat Ngwe TunCharles M. LawrenceMyeong-Hyeon WangCharles Nathan HancockMyeongsang LeeCharles NortonMyong Jo KimCharles RinaldoMyoung-Sook LeeCharles ShulerMyoung-Sook ShinCharles W. SchindlerMyriam AbboudCharlie T. HodgmanMyriam CuadradoCharlotte Von GallMyron G. BestCharng-Cherng ChyauMyron L. ToewsCharupong SaengboonmeeMyron SzewczukCharusluk ViphavakitMyrto KasioumiChase W. NelsonMythili DileepanChathura AbeywickramaN R DilleyChatragadda H. RameshN. Jeelan BashaChayanaphat ChokradjaroenN. S. RepkinaCheah Yoke KqueenNa AbdullahCheang KhooNa WangChe-Chang ChangNabeel AhmedChee Kong YapNabil A. HasonaChe-Hsin LeeNada LabajovaCheil MoonNada M. MostafaChellappagounder ThangavelNadeem KhanChen BaiNadeem UllahChen CaiNadège BellanceChen ChenNadège JaminChen ChuNadège KindtChen Huei LeoNadezhda A. GermanChen ZhaoNadezhda A. GolubkinaChenchen ZhaoNadezhda BokachCheng LiuNadezhda Borisovna TereninaCheng LuanNadezhda GolubkinaCheng YanNadezhda MaksimovaCheng-Chang ChangNadezhda NebogatikovaCheng-Chung YongNadezhda OrlovaChengfei ZhangNadezhda SachivkinaChenghan LiNadia AlatrakchiChenghao ChenNadia Cobo-VuilleumierCheng-Hsien LiuNadia D’AmbrosiCheng-Hsiu LuNadia Elghobashi-MeinhardtCheng-Hsu WangNadia IntanCheng-I LeeNadia JahroudiChengjiang RuanNadia LampiasiChengjie ChenNadia NajafiChenglei LiNádia OsórioChenglin MoNadia RucciCheng-Lin WuNadia ZaffaroniChenglong LianNadica KovačevićCheng-Maw HoNadine BaumgartChengmin JiangNadine DarwicheCheng-Shun LuNadine HeinCheng-Te LinNadine JagerovicCheng-Tien WuNadine Mestre-FrancésChenguang LouNadine WiesmannChengwen SunNadir BettacheChengxin ZhangNadir Nadir ZamanCheng-Yang ChouNadira Y. YuldashevaCheng-Yang HuangNadja FreundCheng-Yoong PangNadja HellmannChengyue WuNadja LehwaldChenhan ZhongNae-Cherng YangChen-Huan YuNafees A. KhanChenjiang YouNaga Babu ChinnamChenlin HuNaga Venkata Sabarinath NeerukondaChenlu GaoNagalingam RajakumarChennaiah AndeNagaraja S. BalakathiresanChenqi TaoNagaraju KerruChen-Si LinNagaraju SakkaniChenyang LiNagavendra KommineniChen-Yong LinNagendra ChauhanChen-Yu KaoNagendra Kumar KaushikChenyu SunNagendrakumar Balasubramanian SinganalurCheol HwangboNagesh MaileCheol-Su KimNagwa Ali SabriCheorl-Ho KimNahed HussienCherednichenko KirillNahla S. El-ShenawyCherie LeeNaihan XuCherng Ru HsuNai-Jen ChangCheryl CeroNaila Francis Paulo De De OliveiraCheryl Ingram-SmithNaim MitreChester CooperNaimeh BahriChet RamNajeeb UllahChhanda BoseNaji KharoufChi Chien LinNakamura YoshikazuChi Chiu WangNakao KuboChi Chun WongNałȩcz-Jawecki GrzegorzChi ZhaoNalu Navarro-AlvarezChia-Che ChangNam Deuk KimChia-Chi WangNam HuynhChia-Chu ChangNam Sook KangChia-Chun TsengNaman VatsaChia-Her LinNamdev S. TogreChia-Hua LiangNamgee JungChia-Hung ChouNam-Gu HerChia-Hung LeeNamita MisraChia-Min LinNamrata DhakaChian Ju JongNan JiangChiang Yu-ChungNan JinChiang-Hung ChouNan XuChiao-Yin SunNan YangChiara AgnolettoNan ZhaoChiara B. M. PlataniaNanae HarashimaChiara BernardiniNancy GavertChiara BiselliNancy Weiland-BräuerChiara BrignoleNanda Kumar YellapuChiara BrulloNandaraj TayeChiara Di TucciNandor Gabor ThanChiara DonadeiNándor NemestóthyChiara Emma CampiglioNan-Fu ChiuChiara FerraciniNan-Kai WangChiara FocaccettiNanlin WuChiara GaiNannan LaiChiara GamberiNannan LuChiara GiacomelliNaoaki RikihisaChiara Maria MottaNaoaki SakataChiara MigoneNaofumi NagaChiara MilaniNaoki HayashidaChiara MolinariNaoki IkegayaChiara MoltrasioNaoki KanekoChiara Paola ZoiaNaoki KanoChiara PolichettiNaoki KawaharaChiara PortaNaoki TeradaChiara QuarnetiNaoki UmemuraChiara RossoNaoki YamamotoChiara ScaramuzzinoNaoko HattoriChiara StranieriNaoko IkutaChiara ValoriNaoko Ohkama-OhtsuChiara VolaniNaoko TakezakiChia-Te LiaoNaoko YanagisawaChia-Wei ChengNaomi Pode-ShakkedChi-Chen LinNaotaka IzuoChichun HoNaoto HashimotoChi-Chun HoNaoto YagiChider ChenNaoto YonezawaChie KojimaNaoufal El HachlafiChie TomikawaNaoya KishikawaChieh Hsi WuNaoyoshi MaedaChien-Chin ChenNaoyuki KataokaChien-Chung YangNaoyuki TaniguchiChien-Feng LiNapoleão Martins Argôlo NetoChien-Min ChiangNarayan BhatChien-Min LinNarayan BhusalChien-Ming ChaoNarayan ShivapurkarChien-Pang LeeNarayanan Nair Arun KumarChien-Tai HongNarcis TribulovaChien-Yie TsayNaren Gajenthra KumarChien-Yu LinNarendar DudhipalaChi-Fen ChangNarender KumarChiharu NishiyamaNarendra Nath JhaChiharu TsujiNaresh KasojuChih-Chia HuangNaresh KumarChih-Chia SuNaresh Kumar RavichandranChih-Chin HsuNaresh PolisettiChih-Feng HuangNarmina O. BalayevaChih-Hao ChenNarongchai AutsavaprompornChih-Hsin HsuNarsimha MamidiChih-Hsing ChouNaseem AbbasChih-Hsun LinNasim Ahmad YasinChih-Hua TsengNasim Nsooudi NsooudiChih-Hung GuoNasim UllahChih-Jen YangNasrollah Rezaei‐GhalehChih-Kuang ChenNasser AlsalehChih-Kuang WangNatacha DreumontChih-Lang LinNatalia A. IgnatenkoChih-Li LinNatalia A. MuralevaChih-Lin LinNatalia AbramenkoChih-Ming MaNatalia AlzaChih-Ping HsuNatália AnicetoChih-Sheng ChuNatalia ArendarczykChih-Yang WangNatalia BalChih-Yao HouNatalia BaulinaChih-Yu ChangNatalia BelkovaChii-Ruey TzengNatalia BelosludtsevaChii-Shen YangNatalia CheshenkoChi-Jung ChangNatália Cruz-MartinsChiming HuangNatalia DrabińskaChi-Ming LiuNatalia Fernández-BertólezChi-Ming WongNatalia FilippovaChin Yan LimNatalia GladkikhChina NaganoNatalia GrubaChinatsu NishidaNatalia GudzChin-Chean WongNatalia Ivanovna AgalakovaChin-Chen ChuNatalia JatkowskaChinedu Ogbonnia EgwuNatalia KaczyńskaChing-Fong ChangNatalia KasicaChing-Jin ChangNatalia KordulewskaChing-On WongNatalia KoubassovaChing-Yi ChenNatalia KruszewskaChing-Yi WuNatalia KurhalukChinh Tran-To SuNatalia LinkovaChin-Ling ChenNatalia MadetkoChinmay K. MukhopadhyayNatália MarquesChinmay Kumar PandaNatália MartinsChinmay R. SurveNatalia MilerChinmay SatamNatalia MotasChinmay V. TikheNatalia N. NinkinaChinmaya MutalikNatalia N. RudenkoChin-Mu HsuNatalia N. ShestakovaChinna Venkata Subbaiah KadiamNatalia NaryzhnayaChin-Wen ChenNatalia Nowacka-JechalkeChin-Yu LinNatalia ParamonovaChioko NagaoNatalia PorotnikovaChip HahnNatalia RuetaloChiranjeevi KorupalliNatalia Sawka-GądekChi-Rei WuNatalia Soriano-SarabiaChi-Ting HorngNatalia TretyakovaChitiphon ChuaichamNatalia TrpchevskaChitra VenugopalNatalia V. BelosludtsevaChiu-Jung HuangNatalia V. GulyaevaChiung-Hui LiuNatalia V. ShcherbikChiung-Yuan KoNatalia Viktorovna NizyaevaChi-Young WangNatalia Viktorovna ZagoskinaChi-Yu LuNatalia WilkeChi-Yun WangNatalia Yanguas-CasásChlebowska-Śmigiel AnnaNatalie A. GudeChloe MartensNatalie EliaCho Rok JungNatalie H. MatthewsCho Yeow KohNatalie HudsonChokri BayoudhNatalie LaibachChong Yang ChuahNataliia V. ShultsChonghang ZhaoNatalija PolovicChong-Tai KimNatalija Ursulin-TrstenjakChongyang DuanNataliya E. NovikovaChong-Yong LeeNataliya GruntenkoChoo Hock TanNataliya K. KovtonyukChris BusbyNataliya KolosovaChris HelliwellNataliya RomanyukChris Hl LimNataliya YaglovaChris Hong Long LimNatalja EigelieneChris MaltmanNatallia DubashynskayaChris MichielsNatallia V. DubashynskayaChris P. MattisonNatalya A. ShnayderChris ProschelNatalya GalibinaChris R. HarrisNatalya GlushkovaChris YoungNatalya RumyantsevaChrishan RamachandraNatalya V PermyakovaChrissoula VoidarouNatarajan RanganathanChrista BuechlerNataša LjubičićChristelle Adam-GuillerminNataša Marčun VardaChristelle CorauxNataša NastićChristelle GuibertNataša S. StanisavljevićChristian BarbatoNatasa ZarovniChristian BehmNatascha VukasinovicChristian Bertram HübschleNatasha KyprianouChristian BetzelNatassa PippaChristian BleilevensNatella EnukashvilyChristian Bravo-RiveraNatércia ConceiçãoChristian BüchterNathalia PadillaChristian Chapa GonzálezNathalie BardinChristian ChervinNathalie Colloc'HChristian D YoungNathalie MarlinChristian DrouetNathalie MorinChristian Griñán-FerréNathalie Sol-FoulonChristian JelschNathalie ViguerieChristian KochNathan CorrellChristian KosanNathan K. EvansonChristian L. LaboisseNathan L. AbsalomChristian LaboisseNathan LanningChristian LehmannNathan MewtonChristian LorsonNathan P. ManesChristian MarxNathaniel VacantiChristian NehlsNatividad Gómez-CerezoChristian ObermeierNatraj KrishnanChristian OpländerNatsuo UedaChristian PoüsNaureen JaveedChristian SetzNavaneeth Krishna Rajeeva PandianChristian SiadjeuNavapon TechakriengkraiChristian SmolleNavdeep GognaChristian StockNaveed AhmadChristian StockmannNaveen DixitChristian TantardiniNaveen GuptaChristian Tellgren-RothNaveen Kumar C GowdaChristian U. OeingNaveen MekalaChristian WeinbergerNavid AbedpoorChristian WirajaNavid Jubaer AyonChristiane AlbrechtNavid RabieeChristiane FuchsNavid SobhaniChristiane S. HampeNavin KumarChristie S HerdNavin Kumar OjhaChristie Ying Kei LungNavneet MatharuChristina BehrNavraj Kaur SaraoChristina Cortez-JugoNavved AhmedChristina D. SotiropoulouNawaf Al-MaharikChristina E. KostaraNayden G. NaydenovChristina GrimmNayoung KimChristina KalpadakisNazareno PaolocciChristina N. BantiNazario FoschiChristina PiperiNazim NassarChristina ZalaruNazzareno CapitanioChristine BlattnerNazzareno D'AvanzoChristine BurkardNdiko LudidiChristine CézardNebojsa AndricChristine CheungNebojsa IlicChristine H FoyerNebojsa PavlovićChristine HappelNebojsa S. ManojlovicChristine HarendtNecla PehlivanChristine J. FarrNecmi DegeChristine LehnerNeda MilinkovicChristine LerouxNeda SladeChristine Lux‐BattistelliNeelaka Molagoda Ilandarage MenuChristine M. SorensonNeelesh Kumar MehraChristine RondaninoNeelu BatraChristine WhiteNeenu SinghChristo KoleNeeraj JainChristof LenzNeeraj MishraChristof WestenfelderNeeraj SoniChristofer JuhlinNeeta LohaniChristoforos ThomasNefeli LagopatiChristoph BührerNeftalí Ochoa-AlejoChristoph CastellaniNeha NandaChristoph CrocollNehad Ali ShahChristoph GablerNehal ElsherbinyChristoph HeierNeife SantosChristoph KaetherNeil DanielsonChristoph KreerNeil H. StollmanChristoph ReinhardtNeil H. ThomsonChristoph WallnerNeil MabbottChristophe DeroanneNeil Richard MillerChristophe DesmetNeil StrotmanChristophe DurantonNejc UmekChristophe GaillochetNejka PotočnikChristophe HanoNektarios KalyvasChristophe LachaudNela MalatestiChristophe LaurentNeli Kinga OlahChristophe PraudNeli KosevaChristopher A. BeaudoinNeli MintchevaChristopher A. BellNelilma RomeiroChristopher CullisNella PreveteChristopher E. PedigoNelson Da Cruz SoaresChristopher GaffneyNelson FerreiraChristopher GernerNelson MarmiroliChristopher GrothNelson OssesChristopher H. HouseNelson PéRez GuerraChristopher HellenNelson RangelChristopher HillNelzo ErefulChristopher J. ButchNemanja GavrilovChristopher KobierzyckiNemanja VukasinovicChristopher LowryNemany HanafyChristopher LucasNemat AliChristopher M RangerNemeth MarkChristopher MakaroffNenad IgnjatovicChristopher P. MattisonNenad JoksimovićChristopher P. ToselandNeng LiChristopher Paul VennerNeonila V. KononenkoChristopher PayanNerea Iturrioz-RodríguezChristopher R. M. AsquithNerea Méndez-BarberoChristopher SjowallNerina DenaroChristopher SmithNerina GnesuttaChristopher T. SemposNermeen Y. AbassChristopher ThrasivoulouNessy JohnChristopher TorrensNéstor García-RodríguezChristopher VeldkampNeusa Yuriko Sakai ValenteChristopher W. VaughanNeveen A. Soliman ElshakhsChristopher WardellNeven MaksemousChristopher WassonNevena PuacChristos GeorgiouNeville VassalloChristos K. KontosNgbolua Koto-Te-NyiwaChristos KissoudisNguan Soon TanChristos MikropoulosNguyen Hoai NguyenChristos PapaneophytouNguyen Kien Truc GiangChristos StournarasNguyen Nhat LinhChristos T. ChasapisNguyen Quoc Khanh LeChristos V. RizosNguyen Quoc KhuongChrong-Reen WangNguyen Thanh LeChrow Ahmed KhurshidNguyen Van QuanChryso Pefkaros KatsoufisNhien Thi NguyenChrysoula G. OrfanidouNhung H. A. NguyenChrysoula VoidarouNi Luh SurianiChrysovalantis KorfitisNiall M. CorcoranChu ChenNian WangChuan LiNian XiongChuan LiuNibedita PatelChuanbin YangNicholas AshtonChuanchin HuangNicholas B. LarsonChuan-Fa ChangNicholas BradshawChuang-Rung ChangNicholas F. FitzChuangye YaoNicholas FischerChuanhai ZhangNicholas G. JamesChuan-Ho TangNicholas HarmerChuanjin WuNicholas J. BuchkovichChuanming XuNicholas J. HaleyChuanrui ChenNicholas J. PolakowskiChuanyu YangNicholas LeadbeaterChuen-Fu LinNicholas PeakeChuen-Mao YangNicholas PullenChuk Young KimNicholas R. HumChukwuma OgbagaNicholas TolwinskiChul-Su YangNick Arthur AndrewsChun ChenNick KouttabChun GaoNicki PanoskaltsisChun Hung ChuNicklas Heine StaunstrupChun LiuNico TeskeChun Nam LokNico UeberschaarChun PanNicola Alberto ValenteChun WangNicola AlessioChunchia ChengNicola ArmstrongChung WongNicola BorboneChung Y. HsuNicola F. FletcherChungchun WuNicola GullickChung-Hsin WuNicola Hamilton-WhitakerChung-Hui LinNicola HofmannChung-Jung LiuNicola IngramChung-Lin LeeNicola Jean Agnes ScottChung-Ming ChenNicola MargiottaChung-Sung LeeNicola MarottaChun-Gu ChengNicola MarranoChunguang DuNicola MontemurroChunguang YangNicola OreficeChung-Wei KungNicola OrzalesiChun-Han ChenNicola PerrottiChunhua QiaoNicola PimpinelliChunhua SongNicola PozziChunjie TianNicola TasinatoChunjin LiNicola TinariChun-Jung ChenNicolae BacalbaşaChunlei LiNicolae BacalbașaChunlei YangNicolae GicaChunliu ZhuoNicolai A. AksenovChunlong LiNicolas BertrandChunping ZhangNicolas CasadeiChunsheng WuNicolas DumazChun-Te WuNicolas ForayChun-Wei TungNicolas GueroutChun-Wu TungNicolas LebonChunxin WangNicolas NassarChunyang XiongNicolas NègreChun-Ying HuangNicolas RicardChun-Yu LiuNicolas RodriguezChunzhang YangNicolas TalarekChunzhao ZhaoNicolas VoituronChutima ThepparitNicolas VuilleumierCicero ArrigoNicole ArrighiCícero CenaNicole BarraCindy BarnigNicole C. L. NoelCindy BerrieNicole FischerCindy KörnerNicole JuffermansCindy L. H. YangNicole L. BatenburgCinta ZapaterNicole Noren HootenCintia StivalNicole PouliCinzia AntognelliNicole Sommer GrünCinzia CiccacciNicoleta AndreescuCinzia FabriziNicoleta AntonCinzia GiacomettiNicoleta Nicole RaduCinzia SignoriniNicoleta PleşuCinzia VolontéNicoleta RaduCiprian JurcuţNicoletta BianchiCiprian RezusNicoletta Di SimoneCiprian TomuleasaNicoletta PedemonteCiro ImbimboNicoletta UrbanoCiro Leonardo PierriNicoletta ZoppiCiro RinaldiNidal JaradatClaire AcquavivaNidhi Jalan-SakrikarClaire De MoreuilNidhi SharmaClaire DebackNidhish FrancisClaire DumenilNiels Grote BeverborgClaire E. Kendal-WrightNiels HansenClaire F. VerschraegenNiels HellingsClaire FrielNiels J. BrouwerClaire JosseNiels J. NieuwenhuizenClaire Stines‐ChaumeilNiels LorenzenClaire StockerNigel YarlettClaire WadeNighat NoureenClaire-Marie DhaenensNihal El NahhasClara BaldinNiharika SharmaClara IannuzziNihay Laham-KaramClara MarínNiina BhattaraiClara MeanaNik TsotakosClara PenasNika PavlovićClara Yongjoo ParkNikesh GuptaClare HoskinsNikhil K. TulsianClare-Louise TowseNikhil Suresh BhandarkarClarissa Fantin CavarsanNikhilesh JoardarClaude BesmondNikhlesh SinghClaude BoucheixNiki KatsikiClaude Kwe YindaNiki L ReynaertClaudia A. Oviedo-SilvaNiki M Zacharias MillwardClaudia AltamuraNikita LiedienovClaudia Avitia-DomínguezNikita PenkovClaudia BonechiNikita TsvetovClaudia BuergerNiko NjiricClaudia CapurroNikol SulloClaudia Castell-MillerNikola MarakovićClaudia ChilomNikola SekulovskiClaudia ColussiNikola StojanovicClaudia CompagnucciNikola Zlatkov KolevClaudia CristianoNikolai A. LisitsynClaudia CrosioNikolai BoshkovClaudia D. AndlNikolai FattakhovClaudia DalmastriNikolai PaceClaudia Daniele Carvalho NavarroNikolaj TravicaClaudia DellaviaNikolaos A. ChrysanthakopoulosClaudia DettoriNikolaos AndreatosClaudia DeusNikolaos AntonakopoulosClaudia DurantiNikolaos AthanasopoulosClaudia FuocoNikolaos ChalmpesClaudia GenoveseNikolaos CharisiouClaudia GiorgiNikolaos E PatsoukisCláudia GomesNikolaos G. SemaltianosClaudia Gonzalez EspinosaNikolaos GiormezisClaudia González-EspinosaNikolaos LabrouClaudia GravekampNikolaos LiarosClaudia HonischNikolaos MachairasClaudia KeilNikolaos N. LourosClaudia LoretiNikolaos NikoloudakisClaudia Madalena Cabrera MoriNikolaos PapandreouClaudia MancaNikolaos PistamaltzianClaudia Marcela Castillo-GonzalezNikolaos PitsikasClaudia MartiniNikolaos PolitakosClaudia MassarottiNikolaos SiderisClaudia MatthäusNikolaos TsakirpaloglouClaudia Muhle-GollNikolaos TsoureasClaudia Ollauri-IbáñezNikolas HeroldClaudia Parada MoraisNikolaus B WagnerClaudia PisanuNikolaus B. BinderClaudia PommerenkeNikolaus GaßlerClaudia RicciNikolaus KneidingerCláudia Sirlene OliveiraNikolaus ThierfelderClaudia SorbiNikolay AleksashinClaudia Teixeira GuimarãesNikolay BrustovetskyClaudia ToniniNikolay E. ShirokikhClaudia Tovar-PalacioNikolay MakishaClaudia TregnagoNikolay MehterovClaudia Uhde-StoneNikolay P. ShapkinClaudia Z. HanNikolay PolyakovClaudio BernardazziNikolay V. GoncharovClaudio BucoloNikoleta PrintzaClaudio CantùNikolett BódiClaudio CeconeNikolett Kállai-SzabóCláudio Daniel Cerdeira‪Nikos A. Afratis‬Claudio Daniel GonzalezNikos TzortzakisClaudio De LuciaNikunj M. ShuklaClaudio FerranteNikunjkumar PatelClaudio Gabriel Lima‐JuniorNilakshi BaruaClaudio IsellaNilda GallardoClaudio LombardelliNilesh SharmaCláudio MaiaNileshi SarafClaudio MurtaNils BillestrupCláudio RoqueNils BömerClaudio RossiNils Petter KjosClaudio TaloraNils RostoksClaudio Téllez SotoNima SanadgolClaudio TrapellaNimi MarcelClaudiu C. PopescuNina Cabezas-WallscheidClaudiu N. LunguNina Jeppesen EdinClaus KordesNina Kacjan MaršičClaus P. W. ZebitzNina M. SaninaClay LentsNina ReuvenCleide Dos Santos SouzaNinaad LasradoClémence BonnetNing LiClemens K. WeissNing LiuClément BarsNing MaClement MesedaNing QuClément PicardNing ZhaoClementina ManeraNinghai GanClementina MesarosNingning ZhaoClementina SitziaNir QvitClinton C. MacdonaldNiraj LodhiClinton WebbNiraj S TopareClive J. PetryNirajan ShresthaClive MckimmieNirakar SahooClivel CharltonNirala RamchiaryClizia ChinelloNirit BernsteinClizia VillanoNirmal MazumderCluu Cam LocNirmala MavilaClyde F. PhelixNirmalya SahaCodruta SoicaNisansala AbeyrathnaColin BarnstableNisarg ModiColin C. SeatonNisha NairColin CampbellNishad Thamban ChandrikaColin DavidsonNishant KulkarniColin DeaneNishant MittalColin GatesNishant TripathiColin J. SucklingNishikant WaseColin KennyNishikawa Shuh IchiColin P. JohnsonNishit GoradiaColin SharpeNitesh Kumar KhandelwalColin W. MacdiarmidNitesh NandwanaColleen CronigerNitesh ShashikanthColleen DociNithya N. KuttyCollette BromheadNithyananthan SubramaniyamColm O'MorainNitikaColuccini CarmineNitin AmdareČoma MatúšNitin K. ChaudhariConcepció AmatNitin KhandelwalConcepción Vaquero LorenzoNitish GulveConcetta AltamuraNittiya SuwannasomConcetta SantonocitoNityanand SrivastavaConcetta ScimoneNivedita ChatterjeeConcettina CappadoneNiyaz MahmoodiConcettina La MottaNiyazi BulutCongbao KangNkulu Kabange RollyCongshan SunNoa B. Martín-CófrecesCongxu ZhuNoa NovershternConnie M. WeaverNoah MoruzziConor BuckleyNoah SternConsolato SergiNoam LevaotConstance AuvynetNoboruv U. ManabeConstance Sewani-RusikeNobuaki HigashiConstant GillotNobuaki MiuraConstantin ApetreiNobuaki OkumuraConstantin CzekeliusNobuhiro NakanoConstantin N. BaxevanisNobuhito GodaConstantin YanicostasNobuko HosokawaConstantino MartínezNobumichi KobayashiConstantinos DeltasNobuo KondohConstantinos G. TsiafoulisNobuyuki IshigeConstantinos MikelisNobuyuki KawashimaConstantinos PitsiosNobuyuki KimuraConstantinos StathopoulosNobuyuki SakayoriConstanze SchmidtNobuyuki TakahashiConsuelo AmantiniNoe Baruch TorresConsuelo CelestiNoel ThomasConsuelo SerresNoelia Zagalaz-AnulaConxita Jacobs-CacháNoemi Anna PesceCoral SanfeliuNoemí Cárdenas-RodríguezCorey L. AndersonNoemi DirzuCorina CarrancaNoemi EiroCorinna ErnstNoemí EsterasCorinna PreusseNoemi Rotllan VilaCorinne GrangetteNoha AbogreshaCornel BaltaNoha AttiaCornelia BlumeNoha H. HabashyCornelia DietrichNoha I. ZiedanCornelia Neidlinger-WilkeNoha MesbahCornelis Johannes Forrindinis Van NoordenNomso Hintsho-MbitaCorneliu Ioan OpreaNoopur ThakurCorneliu TanaseNoor Baity SaidiCornelius KraselNoor KazimCorrado AngeliniNoor LokmanCorrado PelaiaNoor MuhammadCorrado RomanoNoppawan MoralesCorrado TagliatiNoppol ArunratCorrea-De-Araujo RosalyNor Eddine SounniCorreia Joana SofiaNora ChokrCosmin VanceaNora SzentmaryCosmin-Teodor MihaiNorbert GrafCostantino BalestraNorbert HidvégiCostanza Di ChiaraNorbert JordanCostas DelisNorbert KissCostica CaizerNorbert KociokCotas JoãoNorbert LihiCoudane JeanNorbert StefanCraig ByersdorferNorbert WikonkálCraig E. BrownNorberto FarfanCraig ForrestNoreen F. RossiCraig G. BurkhartNoriaki IijimaCraig HodgesNoriaki SasaiCraig UlrichNoriaki YoshidaCrescenzio SciosciaNorihiko NaritaCriscuolo ElenaNorihiro SatoCrismita DmelloNoriko InadaCristián A AmadorNoriko KitamuraCristian A. Moreno GarcíaNoriko TakegaharaCristian BonviciniNorimasa NakamuraCristian CastilloNorio NakataCristian DelceaNorio SogawaCristian Ferreiro SantisoNorio SuzukiCristian FurauNorio YamamotoCristian Ionut OrasanuNoritaka AdachiCristian MateiNoritaka NakamichiCristian MunteanuNoriyasu HirasawaCristian PersuNorma Julieta Salazar LopezCristian Ramirez-GutierrezNorma OviedoCristian RavariuNorman HäfnerCristian SandovalNorman Scott LitofskyCristian ScheauNorrie PearceCristian SilvestriNoula ShembadeCristian StătescuNour Eddine Es-SafiCristian TieranuNoura DosokyCristian TrâmbițașNoureddine BencheikhCristian Tudor MateaNourolah SoltaniCristian V. A. MunteanuNovak James S.Cristiana Iulia DumitrescuNovina MalviyaCristiana RadulescuNovriyandi HanifCristiana VidaliNripendra Vikram SinghCristiane AguiarNuc PrzemysławCristiane CananNucharin SongsasenCristiano CarbonelliNujira TatunCristina AchimNuno CoelhoCristina AndreaniNuno HermannCristina Anna Maria Lo IaconoNuno M. M. MouraCristina ArrigoniNuno S. OsórioCristina BaraleNuno ValeCristina Bilbao-SieyroNunzia D'OnofrioCristina BlebeaNunzia IaccarinoCristina BottinoNunzia MatroneCristina CarucciNunzio Antonio CacciolaCristina CasalouNupur DasCristina ChircovNuray AcarCristina ClementNurdan TozunCristina ComanNuri BașustaCristina DumitriuNuria De PedroCristina FerrerasNuria Esturau-EscofetCristina ForzatoNúria FarréCristina Gabriela GrigoraşNuria MontesCristina GhianiNuria Mulet-MargalefCristina GluhovschiNuria P Torres-AguilaCristina Gomez-CasadoNuria TornerCristina Gómez-MartínNuriban Valero PachecoCristina HasNurlaila IsmailCristina Iulia MitranNuttida RungratsameetaweemanaCristina LópezNuvia M. SaucedoCristina M. FaillaOadi MatnyCristina M. MenéndezOana CadarCristina MaccalliniOana Cristina ArghirCristina MartinezOana RasogaCristina Mas BarguesOana-Maria BolduraCristina MinnelliObyedul Kalam AzadCristina NadaluttiOctavi Martí-SistacCristina NicaOctavia SabinCristina Nunez GonzalezOctavian BuiuCristina OlgasiOctavian Tudorel OlaruCristina OrtegaOctavian-Gheorghe DuliuCristina PaganoOded LiranCristina PolitiOdília QueirósCristina Puig-SausOfer Nathan GofritCristina QuílezOfir HakimCristina RazquinOgnen Pop-GeorgievskiCristina RodríguezOgul Ersin UnerCristina Sánchez De DiegoOhad MazorCristina SatrianoOh-Kyeong KweonCristina SegoviaOier EtxebesteCristina SobacchiOinam Romesh MeiteiCristina SoléØivind BerghCristina SuñolOkay SaydamCristina Tomás-AlmenarOk-Nam BaeCristina TortoliniOksana LastochkinaCristobal EspinosaOksana LockridgeCristoforo PomaraOksana M. SubachCristoforo SilvestriOksana O. KolachevskayaCrystal R. ArcherOksana SherstnevaCrystal S ShinOksana SorokinaCrystal S. ShinOksana V. SalomatinaCrystall SwarbrickOksana Y. ShtarkCsaba BalázsiOksana ZininaCsaba BojtorOksoo HanCsaba ÉvaOkuwaki MitsuruCsaba HetényiOla A. TarawnehCsaba LantosOlaf LarsenCsaba MattaOlan Jackson-WeaverCsaba VáradiOlatunde DahunsiCsaba VizlerOle Andreas ØkstadCsilla Özvegy-LaczkaOle VangCsongor KissOleg A. RakitinCtirad HofrOleg AlexandrovCui LiOleg BaranovCunmin QuOleg BelovCuong X. NguyenOleg Ivanov KolodiazhnyiCurt D. SigmundOleg KaradutaCurtis OkamotoOleg KovtunCurtise Kin Cheung NgOleg ManaenkovCushla MckinneyOleg MikhailovCustodia FonsecaOleg N. TimofeevCynthia BlantonOleg SerovCynthia GleasonOleg ShuvalovCynthia Marie-ClaireOleg TutanovCynthia RosenthalOleg V. BatishchevCyril AlexeevOleg V. DolotovCyril EleftheriouOleg V. GradovCyril FersingOleg VitrikCyril Glenn P. SatuitoOleh AndrukhovCyril MessaraaOleh PochynyukCyril NicolasOleksandr TkachCyrus MotamedOleksandra TiapkoCzang-Ho LeeOleksii SkorokhodCzeslaw RadziejewskiOlena IvashchenkoD Gareth EvansOlena StabnikovaD. M. S. B. DissanayakaOlesia MakhutovaD. Mohd. Azam AnsariOlesya V. StepanenkoD. S. KishorOlga A. AleynovaD. S. PrabakaranOlga A. KoksharovaD. S. RamakrishnaOlga A. RozentsvetD. V. ShumilinaOlga A. SerenkoDa SunOlga A. SnytnikovaDa Yeon KimOl'Ga A. TarasovaDace GraudaOlga Alexandra Chinita PirrolasDae Youn HwangOlga AnatskayaDae-Hee LeeOlga BaronDaeho ParkOlga BelykhDaekyu SunOlga BoytsovaDae-Seok HwangOlga CalvoDae-Sung LeeOlga DichalaDaewon JeongOlga DlugoszDae-Wui YoonOlga E. GlukhovaDafne BongiornoOlga E. KarpichevaDafne Campigli Di GiammartinoOlga Giménez-PalopDafni Despoina AvgoustakiOlga GordeevaDafydd D. L. JonesOlga HeidingsfeldDa-Gang HuOlga I. YarovayaDagmar KratkyOlga Igglessi-MarkopoulouDagmar Meyer Zu HeringdorfOlga IranzoDagmar RiemannOlga JanouskováDagmar SchinnerlOlga Jovanovic GlavasDagmara KabzińskaOlga KaltsaDagmara KłopotowskaOlga KochetovaDagmara Szmajda-KrygierOlga KopachDagmara WiniarczykOlga KorczeniewskaDagnija LazdiņaOlga KozaderovaDa-Hua WeiOlga KozlovaDaiji EndohOlga KrizanovaDaike TianOlga KronjaDaiki TaniyamaOlga L. VoroninaDaili PengOlga Lilia Garibay-CerdenaresDainis RungisOlga LuzinaDaishi YamakawaOlga MalyshevaDaisuke AriyasuOlga MartzoukouDaisuke FujitaOlga MatveevaDaisuke FunabaraOlga Mikhailovna BazanovaDaisuke HayasakaOlga NetskinaDaisuke HoshinoOlga OstrovskyDaisuke IshiiOlga PagonopoulouDaisuke KobayashiOlga PosukhDaisuke KohdaOlga ProtchenkoDaisuke MikiOlga RechkoblitDaisuke SeoOlga RedinaDaisuke TakayanagiOlga Rojo-PovedaDaisuke TodakaOlga S. FedorovaDaisy HoaglandOlga S. TarasovaDaitoku SakamuroOlga SelyutinaDaiva BironaitéOlga ShamovaDajana LendakOlga ShchaginaDale FeldmanOlga ShevchukDale GreenOlga SztatelmanDaley S. MoreraOlga TrhlíkováDali HuangOlga TsivilevaDali ZhengOlga Tura-CeideDalia Anwar HamzaOlga UrbanekDalia M. KopustinskieneOlga V. GrinevaDalia MedhatOlga V. MorozovaDa-Liang OuOlga Vadimovna KurmyshkinaDalibor ValíkOlga VinogradovaDalila Laoudj-ChenivesseOlga WesołowskaDalila MieleOlga Witkowska-PilaszewiczDalila PettaOlga Y. KorolkovaDalila ScaturroOlga Yu BakulinaDalin ZhangOlga ZimmermannováDalius VitkusOlgica NedicDaman KumariOlgirda BelovaDamar Lopez-ArredondoOlha MykhailenkoDamian GawelOlimpia LaiDamian MikulskiOliver AmbréeDamian NakoniecznyOliver CrookDamian WarzechaOliver GoldbeckDamiana ScuteriOliver GrauerDamiano FantiniOliver GrussDamião Pergentino De SousaOliver H. KrämerDamien ArnoultOliver PatschanDamien CoupeauOliver StrbakDamien GuindoletOliver TuševskiDamien S. K. SamwaysOliver UllrichDamijan KnezOliver Von Bohlen Und HalbachDamijana Mojca JuričOliver ZerbeDamir FabijanićOlivera Mitrović AjtićDamu TangOlivia Francis-BoyleDan AokiOlivia MajerDan ChenOlivier AliDan Cristian RaduOlivier ArmantDan Cristian VodnarOlivier BarisDan HaoOlivier BaudDan QiOlivier DeschaumeDan ShiOlivier DormondDan SongOlivier EscaffreDan VoiculescuOlivier JoubertDan YanOlivier MeuretteDan ZhuOlivier MoralesDana Carina BaiuOlivier PeulenDana Elena PopaOlivier ReynardDana Gabriela FestilaOlivier SireDana HuskensOlivier SorgDana JurkovicovaOlivier Van WuytswinkelDana KühnelOliviero CarugoDana Lucia StanculeanuOliwia Gawlik-KotelnickaDana Maria CopolovicOlufemi Emmanuel BabalolaDana Maria CopoloviciOluwaseun AdeyanjuDana StoianOm Prakash MeenaDanail PavlovOm Prakash NarayanDanay CibrianOmanma AdighibeDancho DanalevOmar Al MassadiDandan GuoOmar BreikDandan SunOmar CauliDandan YangOmar El BounkariDandan ZhuOmar Emiliano Aparicio-TrejoDane WolfOmar Farookh PinjariDangdang LiOmar Guzman-QuevedoDanh D. TruongOmar HewedyDani SatyawanOmar KujanDanial RoshandelOmar PorrasDanica JovićOmar TlibaDanica MichaličkováOmero Bendicto Poli-NetoDaniel A. AbugriOmid AkhavanDaniel ArcanjoOmid HamidiDaniel ArcosOmojola AwogbemiDaniel B. StovallOndrej CehlárDaniel B. YaroshOndrej HolasDaniel BaeckerOndrej KodetDaniel BoegerOndrej KvitekDaniel C. MorrisOndřej PlíhalDaniel CosanoOndrej SimonikDaniel D. ShapiroOndrej SlanarDaniel EllederOndřej SoukupDaniel Enosi TuipulotuOndrej VasicekDaniel Fernández-VillaOnno MeijerDaniel FusterOnofrio LaselvaDaniel GackowskiOphélia Le ThucDaniel García SoutoOr KakhlonDaniel Gonçalves-CarneiroOra IsraelDaniel González-NietoOrestis NousiasDaniel GromadzkiOrhan OzdemirDaniel H. González MaglioOrhan Tansel KorkmazDaniel J WhitcombOriana Lo ReDaniel J. ShiwarskiOriana TrubianiDaniel J. SmitOrietta PicconiDaniel J.V.A. Dos SantosOrit UzielDaniel Jacobo-VelazquezOrkide KoyuncuDaniel JanitschkeOrlando CastellanoDaniel KeeferOrlando Guntinas-LichiusDaniel KronenbergOrnella CalderiniDaniel L. PiskorzOrnella CapellariDaniel LighezanOrnella CominettiDaniel López MaloOrnella MaglioDaniel M. CzajkowskyOrsola Di MartinoDaniel Martinez-FongOrsolya Eszter MolnárDaniel MccartneyOrsolya KekesiDaniel Moreno-AndrésOrsolya Kinga GondorDaniel NeureiterOrsolya TökeDaniel Ortega-CuellarOsama A. ElkashtyDaniel Oruño-SahagunOsama A. KishtaDaniel P. PotaczekOsama G. MohamedDaniel P. ZalewskiOsama MadkhaliDaniel Pedrosa AlvesOsamu AmanoDaniel Penteado Martins DiasOsamu GotohDaniel Pierre PetitOsamu IshidaDaniel PotterOsamu UrakawaDaniel R GonzalezOscar A. Lenis-RojasDaniel R. BushÓscar Cabeza GrasDaniel RamsköldOscar CasisDaniel Robert SchlattererÓscar Conchillo-SoléDaniel RoethOscar CorderoDaniel Romaus-SanjurjoOscar LaoDaniel RoystonOscar LazoDaniel SmržÓscar Lorenzo GonzálezDaniel Sobrido-CameánÓscar M Bautista-AguileraDaniel ŠpoljarićOscar NunezDaniel SzulczykÓscar Osorio-ConlesDaniel T ChristianOskar A. HaasDaniel TaillandierOskar A. PalaciosDaniel TodtOskar SiemianowskiDániel TörőcsikOskar WasielewskiDaniel Torres-LagaresOskar ZakiyanovDaniel UngarOsmar Antonio Jaramillo-MoralesDaniel VaimanOsvalds PugovicsDaniel ValleraOswaldo Valdés LópezDaniel VittetOthmane MerahDaniel W. NixonOtilia CintezaDaniel WilkinsonOtmar HuberDaniel WoldringOttavia GiampaoliDaniel Youssef BargieriOtto W. WitteDaniela Amidžić KlarićOualid EllouzDaniela BraconiOuathek OuerfelliDaniela BuchaimOumaima AmmarDaniela C. VazOvidiu Cristian OpreaDaniela CaccamoOvidiu DimaDaniela CalinaOvidiu OnigaDaniela Carmen AbabeiOvidiu PopDaniela Cristina BergerOvidiu SirbuDaniela Cristina CulitaOvidiu TitaDaniela CurtiOwen LeiserDaniela De ToteroOxana DobrovinskayaDaniela Elena ZavastinOxana TrifonovaDaniela FerrariOya CingozDaniela FlossOyuna KozhevnikovaDaniela GabbiaOzgur BasalDaniela GasparottoOzohanics OliverDaniela GiacomazzaOzren PolasekDaniela GiulianiOzren PolašekDaniela HaluzaP. Bryant ChaseDaniela HanganuP. H. M. LeungDaniela HozborP. Venkateswara RaoDaniela IannazzoPabitra Kumar JenaDaniela ImpellizzeriPablo BlancoDaniela ImperioPablo BoixedaDaniela IsolaPablo García De FrutosDaniela JakšićPablo Giménez-GómezDaniela JežováPablo Hernansanz-AgustínDaniela Joyce Trujillo SilvaPablo Jesús Marín-GarcíaDaniela L. JabesPablo Jiménez-CalvoDaniela L. RebolledoPablo Loza-AlvarezDaniela LeccaPablo MurielDaniela MarascoPablo PowerDaniela Maria MateiPablo StiefelDaniela Maria TanasePablo Thomas-DupontDaniela MarzioniPablo Vivas MejiaDaniela MinerdiPadet SiriyasatienDaniela MiricescuPaiboon JungsuwadeeDaniela MontesarchioPajor LászlóDaniela NovickPak Alexander J.Daniela Opris-BelinskiPak Hin Hinson CheungDaniela Palacios GarcíaPakeza DrkendaDaniela PiancatelliPakieli H. KaufusiDaniela PredoiPalak PatelDaniela QuaglinoPalani ElumalaiDaniela RegazzoPalaniappan AshokDaniela RiganoPalaniraja ThandapaniDaniela RosadoPalanisamy NallasamyDaniela RossiPalash MandalDaniela RovitoPallabita ChowdhuryDaniela SapienzaPallavi ChandraDaniela Simina StefanPallavi ShrivastavaDaniela Sonja NoschPalmieri MichelleDaniela TerraccianoPalmy JesudhasanDaniela UbertiPaloma Tejera NevadoDaniela Valdez-JassoPamela Bond CassidyDaniela ValensinPamela Di GiovanniDaniela ValentiPamela E PotterDaniela Valeria MinieroPamela RossoDaniele BassiPan GaoDaniele Filippo CondorelliPanagiota KoralliDaniele La RussaPanagiota MitrouDaniele LilleriPanagiota XaplanteriDaniele MancardiPanagiotis AthanassiouDaniele MantionePanagiotis EfentakisDaniele MarcelliPanagiotis G. HalvatsiotisDaniele MasaronePanagiotis IoannidisDaniele MercatelliPanagiotis J. SkourasDaniele SilvestriPanagiotis KalaitzisDaniele SolaPanagiotis KarakaidosDaniele SommaggioPanagiotis LianosDaniele TibulloPanagiotis MadesisDaniele TorellaPanagiotis MallisDaniele Ugo TariPanagiotis Nikolaou MoschouDaniele VeclaniPanagiotis PeitsidisDaniele VergaraPanagiotis TheofilisDanielle CarlinPanagiotis TsiartasDanielle H. DrabeckPanayiota L. PapasavvaDaniel-Valentin SavatinPanayiotis LoukopoulosDaniil A. KozlovPanayotis KalozoumisDaniil A. LukyanovPancham Lal GuptaDaniil EurovPanda SantoshDaniil IvanovPandu GangulaDaniil KolokolovPandurang KolekarDaniil N. BratashovPaniz JasbiDaniil N. BratashovPankaj AhluwaliaDaniil OlennikovPankaj BhattDaniil P AksenovPankaj ChaudharyDaniil PopovPankaj GaurDanijel D. MilinčićPankaj SharmaDanijel KarolyiPankaj TrivediDanijela BarićPankita H. PandyaDanijela KrsticPanos PapagiannakopoulosDanijela MiljkovićPanteleimon E. PapakonstantinouDanijela PetrovicPanteleimon LivanosDanijela Ristić-MedićPantu Kumar RoyDanila B. VasilchenkoPao-Chu WuDanila De VitoPaola AmmendolaDanila LimauroPaola AngeliniDanilo BoskovicPaola AntoniniDanilo LofaroPaola BadinoDanilo Martins Dos SantosPaola BagnoliDanilo MilardiPaola BarrajaDanilo Roman-CamposPaola BraghettaDanis VerdeciaPaola BrivioDanish IqbalPaola CuomoDanko MiloševićPaola De CandiaDanlong JingPaola De CiccoDanny DhanasekaranPaola De LucaDanny Van Der HelmPaola De PadovaDanrui NiPaola Di PietroDanuta BranowskaPaola FacherisDanuta Gąsior-PerczakPaola FaraoniDanuta JantasPaola FossaDanuta Januszkiewicz-LewandowskaPaola GavazzoDanuta MiedzinskaPaola GiussaniDanuta NowickaPaola GramaticaDanuta WitkowskaPaola GratteriDaojun LiuPaola I. Angulo-BejaranoDapeng ChenPaola LenziDapeng PengPaola LondeiDaqian GaoPaola ManiniDardiotis EfthimiosPaola MaycotteDarek GoreckiPaola ParenteDaria LytkinaPaola PiomboniDaria PoshinaPaola PiscopoDaria TokicPaola RinchettiDaria V. VasinaPaola RizzoDaria Y. RomanovaPaola RoncadaDariga AkashevaPaola RotaDarin ZertiPaola SassiDarío Acuña-CastroviejoPaola SemeraroDario BalestraPaola SenaDario BrunettiPaola Sinibaldi VallebonaDario Di NardoPaola StoriciDario FinazziPaola TuranoDarío LirussiPaola VendittiDario MizrachiPaola ViganòDario PaoloPaola ZigrinoDario RuscianoPaolo AbondioDario SiniscalcoPaolo AccorneroDarius BalciunasPaolo ArosioDarius HäuslerPaolo AseniDariusz BorońPaolo BoffanoDariusz JedrejekPaolo BonaldoDariusz KałkaPaolo CapparèDariusz KucharczykPaolo CavorettoDariusz KulusPaolo D. PigattoDariusz LatowskiPaolo EdomiDariusz Maciej PisklakPaolo FagoneDariusz MikołajewskiPaolo GabrieliDariusz PańkaPaolo GovernaDariusz PiesikPaolo GuglielmiDariusz RakusPaolo ImmovilliDariusz SitkiewiczPaolo IovienoDariusz T. MlynarczykPaolo LimoliDariusz Wojciech MazurkiewiczPaolo MonardoDariusz ŻurawekPaolo NataleDarja LavoginaPaolo NevianiDar-Jen HsiehPaolo OrsariaDarko BosnakovskiPaolo PalmiscianoDarko ModunPaolo PastorinoDarko PreinerPaolo PellegrinoDarlene MitranoPaolo ScarpelliDarrell RickePaolo ScudieriDarren M. GordonPaolo SeverinoDarren MossPaolo SpinnatoDarshan TrivediPaolo TortoraDarshna YagnikPaolo TotiDarya NovopashinaPaolo TrucilloDarya TishkevichPaolo Vincenzo PedoneDaryl O. SchwenkePaolo ZardiDaryoush Shahbazi-GahroueiPapanikolaou DimitriosDas PoushaliParamasivam NithyanandDaša StupicaParameswaran G. SreekumarDat HaParamita ChakrabortyDaumantas MatulisParamjit TappiaDave GilmoreParas Singh MinhasDavi Marco Lyra LeiteParaskevas LybérisDavid A. BenfieldParaskevi KarousiDavid A. DavisParaskevi KritsiligkouDavid A. DeanParaskevi L. TsiolakiDavid A. GewirtzParaskevi TrivilouDavid A. WadaParasuraman PavadaiDavid AbbottParasvi S. PatelDavid Aguilar-PérezPardo-Medina JavierDavid AlexanderParesh K. SamantarayDavid Arranz-SolísParesh ShrimaliDavid Baez-NietoParés-Matos ElsieDavid BasantaParham Jabbarzadeh KaboliDavid BeversdorfParimalan RanganDavid BotequimParis D. N. SvoronosDavid BrooksParisa GazeraniDavid C. KennedyParisa YousefpourDavid CabañeroParizad Parisa AbbasiDavid ClaxtonParna SahaDavid CluetParra Maria TeresaDavid DayParracino AntoniettaDávid DeriteiParskson Lee Gau ChongDavid DixonPartha BasuDavid DolivoPartha Narayan DeyDavid DoraPartha Pratim SahuDavid DormanPartha Sarathi AddyDavid DouchesParthiban SubramanianDavid DraperParvaneh PakravanDavid E. OchayonParvathaneni Naga SrinivasuDavid E. StecParvaze SofiDavid EscorsParveen ParasarDavid FelicianoParvez AlamDavid FierliParvez KhanDavid GagneulParviz VahediDavid GaulPascal BrioisDavid GloverPascal ColosettiDavid GraingerPascal DarbonDavid GreenhalghPascal PeraldiDavid HaigPascal RowartDavid HaložanPascal SchlosserDavid HarmanPascale FanenDavid HartPaschalis TheotokisDavid HearnPasquale AuricchioDavid HillPasquale De LucaDavid HoodPasquale DitonnoDavid HorvathPasquale Fabio ProvenzanoDavid J. KukulkaPasquale LongobardiDavid J. LundyPasquale MastrorobertoDavid J. McgeePasquale MoneDavid J. MontefuscoPasquale ParibelloDavid JamesonPasquale PisapiaDavid José Casimiro AndradePasquale SaggeseDavid K. OrrenPasquale ScarciaDavid KinderPasquale SimeoneDavid KnazovickyPasqualina BuonoDavid KomatsuPasqualina D'UrsiDavid KovacsPasseri ElodieDavid KumpPatcharawalai WhongsiriDavid LambPathak BhuvanDavid LawPatial VikramDavid LeavesleyPatric DelhantyDavid Lee PhillipsPatrice BourgeoisDavid LiberlesPatrice GaurivaudDavid Li-KroegerPatrice Nsangou MimcheDavid M. WithallPatrice RollDavid MachadoPatrice X. PetitDavid MamprePatricia A. BoleyDavid MedinaPatricia Agudelo-RomeroDavid Medina-CruzPatricia BordesDavid MillsPatricia DiogoDavid MuPatrícia FaíscaDavid N. DanforthPatricia Gálvez-MartínfDavid NegusPatricia García GarcíaDavid NelsenPatricia HuebbeDavid NelsonPatricia J. ArmatiDavid P. GallowayPatricia J. LardoneDavid PagliaPatricia J. MclaughlinDavid PoynerPatricia LalorDavid R. HuffPatricia Losada-PerezDavid RamonetPatrícia MacielDavid Ross SibsonPatricia P. WadowskiDavid S. GreenbergPatrícia PiresDavid S. GyöriPatricia Sebastián-LeónDávid S. GyöriPatricia T JimenezDavid S. WeberPatricio MenesesDavid Sánchez-MolinaPatricio MuñozDavid SchuermannPatrick BradshawDavid SemerilPatrick CarberryDavid SergeevichevPatrick CelieDavid ŠilhaPatrick D. FischerDavid SinclairPatrick DansetteDavid SprayPatrick DimarioDavid StudholmePatrick F DowdDávid SzatmáriPatrick G. ArndtDavid T. StuartPatrick GeraghtyDavid TulasnePatrick HaubruckDavid VancePatrick HerrDavid Vicente-ZurdoPatrick HillesheimDavid W. DyerPatrick KwongDavid Wai ChanPatrick McnuttDavid WelchPatrick NarbonneDavid ZehPatrick NasarreDavide BarbagalloPatrick PoucheretDavide BarrecaPatrick RonaldsonDavide BianchiPatrick SüβDavide BochicchioPatrik Ferreira VianaDavide BorroniPatrik SobolciakDavide CostaPatrina ParaskevopoulouDavide FirinuPatrizia AgrettiDavide Giuseppe RibaldonePatrizia AmadioDavide LauroPatrizia BovolinDavide LeccaPatrizia CancemiDavide MarangonPatrizia CasellaDavide NocitaPatrizia FalabellaDavide PorrelliPatrizia FerraboschiDavide PradellaPatrizia GaleffiDavide RoncaratiPatrizia GenaDavide SartiniPatrizia GuidiDavide SeruggiaPatrizia HreliaDavide TagliazucchiPatrizia LicataDavide ZellaPatrizia LoprestiDavood Domiri GanjiPatrizia PignattiDavood ToghraiePatrizia RomaniDawei JiangPatrizia SabatelliDawei SongPatrizia VaccinoDawei WangPatrizia ZaramellaDawei ZhouPatrycja KaczaraDawid GawlińskiPatrycja KleczkowskaDawid MadejPatrycja MakośDawid PrzystupskiPatrycja MichalskaDawid StefaniukPatrycja RedkiewiczDawid SzwedowskiPatrycja S. MatusikDawn CoatesPatrycja WińskaDax Aaron HoffmanPatrycja ŻakDaxesh P. PatelPatryk FrąckowiakDayane AlvaresPatryk KoniecznyDaylan A. Tzompa-SosaPatryk LipinskiDaylon JamesPatryk TarkaDayong ShiPattama WongsirisinDa-Yuan ChenPaul A FosterDayun YanPaul A. CorrisDay-Yu ChaoPaul AirsDe Gaulle I ChigbuPaul AyersDe GongPaul B. KaplowitzDe La Luna SusanaPaul C. EngelDea SladePaul Chi Lui HoDean G. PhelanPaul ClaytonDeanna SosnowskiPaul ColemanDeb Dutta SayanPaul CullenDebabrata BanerjeePaul CummingDebabrata MandalPaul D'AgostinoDebanjali DasguptaPaul DalhaimerDebasis BagchiPaul DevlinDebasis ChattopadhyayPaul DijkwelDebasish BandyopadhyayPaul F. Rugman-JonesDebasish Kumar DeyPaul F. SmithDebatosh DasPaul F. WilliamsDebdeep DuttaPaul G. FurtmüllerDebipreeta BhowmikPaul G. LayerDebjani PalPaul G. SeyboldDeblina BiswasPaul GissenDebmalya BarhPaul GooleyDebora CapelliPaul H. M. HarrisonDebora ColangeloPaul J. DysonDebora CurciPaul JaraDebora Decote-RicardoPaul JenningsDébora FerreiraPaul JoyceDébora LevyPaul KirchbergerDebora NapoliPaul M. HasegawaDeborah A. SarkesPaul M. StemmerDeborah CharlesworthPaul MatejtschukDeborah CroteauPaul MozdziakDeborah J. KeszenmanPaul R. AndreassenDebottam SinhaPaul RöschDecheng RenPaul S. AmieuxDeclan P. MckernanPaul S. HafenDeepak B. Thimiri Govinda RajPaul ScheierDeepak KulkarniPaul SloweyDeepak KumarPaul TownsendDeepak Kumar KhajuriaPaul WadsworthDeepak Kumar YadavPaula A. OliveiraDeepak SharmaPaula Alexandra PostuDeepak Singh ChauhanPaula AmatoDeepak VangalaPaula BoaventuraDeepali P SundraniPaula C. JacksonDeepani FernandoPaula Christine JimenezDeepanjan PaulPaula Corte-LeonDeependra Kumar SinghPaula D. PrinceDeepika AroraPaula DietrichDeepika AwasthiPaula DoboszDeepika JoshiPaula FernandezDeepika SirohiPaula FordDeepshikha MishraPaula M. FerreiraDeepti ChaturvediPaula MuloDeepti NigamPaula NuñezDeftereos SavasPaula PierozanDeguan LvPaula PintoDehong YuPaula Sáez-EspinosaDeike Hesse-WiltingPaula TavaresDejan AgićPaula VagosDejan BudimirovicPaul-François GalletDejan GeorgievPaulina BednarczykDejan MilenkovicPaulina BorkowskaDejan StojkovićPaulina Carmona-MoraDel Amo CristinaPaulina CarribaDelfa Radić-KrištoPaulina CzaplewskaDelia Chillura MartinoPaulina GlazińskaDelia HorhatPaulina KazimierczakDelia TitPaulina KoczurkiewiczDelicia Shu Qin OoiPaulina Latko-DurałekDelilah F. WoodPaulina MertowskaDelio José MoraPaulina MisztakDémène HélènePaulina Niedźwiedzka-RystwejDemeter TzeliPaulina SobierajskaDemetra GiuriPaulina StachulaDemetra SocolovPaulina TokarzDemetrios ArvanitisPaulina WignerDemetrios G. VavvasPaulina WlasiukDemin CaiPaulina ŻelechowskaDemitrios VyniosPauline AntonDénes DuditsPauline E. JullienDénes LőrinczyPauline EstevesDenes TothPauline PannetierDengchao CaoPaulino Gómez-PuertasDeng-Guang YuPaulius GrigaravičiusDenis BaranenkoPaulius RuzgysDenis BaranovskiiPaul-Mihai BoarescuDenis BazhinPaulo BettencourtDenis BeliaevPaulo Cesar De MoraisDenis BourgeoisPaulo Eduardo TeodoroDenis J WakehamPaulo GasparDenis KorneevPaulo GavaiaDenis N. SilachevPaulo J. PalmaDenis PolancecPaulo Lordelo Guimaraes TavaresDenis RychkovPaulo Magno Martins DouradoDenis S. KudryavtsevPaulo MartinsDenis SerenoPaulo MascarenhasDenis SilachevPaulo MatafomeDenis SoulièresPaulo MatosDenis VivienPaulo SanchesDenisa MarginǎPaulo SantosDenise ArrickPaulo ValeDenise Freitas Siqueira PetriPaulo ZainiDenise SchramaPaulraj KanmaniDenise ToscaniPavan Anil KumarDenitsa TeofanovaPavan Kumar DhanyamrajuDeniz Tanıl YücesoyPavel A NazarovDennis FlanaganPavel A. ZykinDennis GerloffPavel AbramovDennis HarrerPavel Anatolyevich NikolaychukDennis LevinsonPavel BabicaDennis SimpsonPavel BouchalDennis V. CokkinosPavel DemakovDennis WelkerPavel FeduraevDenys A. KhaperskyyPavel H. Lugo-FabresDeo SinghPavel HasalDeokho LeePavel KerchevDeok-Yong ParkPavel KomarovDepei LiPavel KotrbaDeqin YangPavel KroupinDequan LiPavel LafataDerek B. OienPavel LobachevskyDerek HausenloyPavel M. BorodinDerek J. B. KleinveldPavel MakarevichDerek ParsonagePavel MakarevichDerick HanPavel PadnyaDerick WansinkPavel PashkovskiyDerrick GlascoPavel RehakDerry K MercerPavel SemenyukDerval Dos Santos RosaPavel SolopovDer-Yen LeePavel ŠtarhaDesalegn D. SerbaPavel UrbanekDeshayes SamuelPavel V. AvdoninDesheng LiangPavel V. AvdoninDesire Molina AlcaidePavel V. PogodinDésirée WünschPavla Maňásková-PostlerováDesislava Georgieva TenevaPavla PostlerovaDespina A. TataPavlina DolashkaDespoina BerisPavlinka DolashkaDessislava TodorovaPavol ZelinaDetlef NeumannPawan KumarDevadas KrishnakumarPawan Kumar MishraDevapriya SinhaPawel Antoni KolodziejskiDeven PatelPawel BorowieckiDevendra H. ShahPaweł ChmielarzDevendra KumarPaweł CyplikDevesh PantPawel GawlinskiDevi AtukorallayaPawel K. LorkiewiczDevi Prasan OjhaPawel KleczynskiDevin StauffPaweł KordowitzkiDevin W. McbridePaweł KowalczykDevina UngPawel KrupaDevis BenfaremoPawel KwiatkowskiDeying LuoPaweł MarciniakDeyse Christina VallimPawel MozejkoDezsö ModosPawel NakielskiDhana GorasiaPaweł OlczykDhananjay PandeyPaweł PomorskiDhananjay YadavPaweł RamosDhanarajan RajakumarPaweł SiudemDhanasekaran SugapriyaPawel StaszekDhanesh Sivadasan BinduPaweł ŚwitDhanush HaspulaPawel SzczesnyDhara SharmaPaweł TurekDharmendra BhatiaPawin PongkorpsakolDharmendra ChoudharyPayal GangulyDharmendra Kumar YadavPayam BehzadiDharmendra Pratap SinghPayam SadeghiDharmeshkumar PatelPayam SoltanzadehDhiman PalPayam ZarrintajDhimant DesaiPayaningal R. SomanathDhiraj P. MuralePediaditakis IosifDhirendra KumarPedro A. González-MorenoDhriti SinhaPedro A. Jiménez GómezDhruba PathakPedro A. JoseDhruv DesaiPedro Aguilar-ZarateDhruvajyoti RoyPedro AraujoDhwani KansalPedro BullonDi LiuPedro D. VazDi Miceli MathieuPedro EsbritDi WuPedro FernandesDi ZhangPedro FerreiraDiaa E. DaghmaPedro FonteDiaa MoneimPedro Gonzalez-MenendezDiaga DioufPedro Luis Ramos-GonzalezDiana Andreia Tavares PintoPedro M. E. ManciniDiana BedollaPedro M. SantosDiana Camelia NuţǎPedro Martín-AcostaDiana Castro-RodríguezPedro Martínez-GómezDiana CenariuPedro Ojeda-MayDiana CheshmedzhievaPedro Ostoa-SalomaDiana CiolacuPedro PellicerDiana Cláudia PintoPedro Perez-ManceraDiana Cristina Pérez-IbavePedro PiedrasDiana Crus-TopetePedro RoblesDiana Cunha-ReisPedro Santana Sales LauriaDiana DiasPedro SilvaDiana DonatellaPedro TavaresDiana Drago-GarcíaPeep PalumaaDiana FedunovaPeeyush LalaDiana GuimarãesPeeyush RanjanDiana H TaftPegah JohanssonDiana KataPeggy LafusteDiana KolbePeggy MarconiDiana LupuPei Yuin KengDiana Luz Juárez-FloresPei-Cheng HuangDiana Marcela CastilloPei-Fen YenDiana MartinsPeifeng SuDiana ObistioiuPeigao LuoDiana Ovejero CrespoPei-Gen RenDiana Panayotova-DimitrovaPeiguo YuanDiana PortanPei-Hui LinDiana Rodríguez-EspinosaPeijiang LiuDiana SalikhovaPeijin LiDiana VellutoPei-Jung LuDiana Zepeda-OrozcoPei-Ling HsiehDianbao ZhangPei-Luen LuDianchang ZhangPei-Min ChaoDiane EvrardPeiqi YinDiane Mcvey WardPeisong GaoDiane V. LefleyPeitao LüDianne FordPei-Yi ChuDianne WebsterPei-Yin TsaiDibyajyoti PramanikPeiyu CaiDidier ColinPejman RohaniDidier DesmaëlePellegrino MazzoneDidier F. PisaniPenélope AguileraDidier PicardPenelope IoannidouDidier VincentPenelope Kay-FedorovDiego A. RojasPenelope Perkins-VeazieDiego Alvarez De La RosaPeng ChenDiego Alzate CorreaPeng GaoDiego BonattoPeng GuoDiego CortezPeng LiDiego CotellaPeng LiaoDiego F. TiradoPeng WangDiego FerreroPeng WeiDiego Franco JaimePeng YuDiego García-AyusoPeng ZhaoDiego Hernán SánchezPeng ZhouDiego HojsgaardPengbo WangDiego KrapfPengbo ZhangDiego La MendolaPengcheng XuDiego LuceroPengda LiuDiego Luís CostaPengfei ZhangDiego MoraPenghao LiDiego QuirogaPenghua WangDiego RaimondoPengpeng LiuDiego Romano PerinelliPengxiang ZhuDiego SampedroPengyu ZongDiego SerraPeng-Yuan WangDieter LottPengyuan ZhengDieter SchinzerPenny AsbellDieter SteinhagenPer Amstrup PedersenDietmar MansteinPer Jr GreisenDietmar ZaissPer SvenningssonDigamber RanePer UhlenDijana Žukovec TopalovićPeramaiyan RajendranDik Van GentPere CastellDikaia XenakiPere PuigdomènechDilara R. MaslennikovaPereira RubenDileep SharmaPerez-Valle ArantzaDilek KilliPeriasamy SelvarajDilip ShahPeriyasami GovindasamiDillon MintoffPerla D. MaldonadoDima SabbahPernille HolmagerDimiter N. PetsevPero LucinDimitra GkikaPerrie F O'Tierney-GinnDimitra HouhoulaPershina OlgaDimitra MilioniPersio Dello SbarbaDimitra MitsiouPerveen SummiaDimitri AnzelliniPeta HarveyDimitri PoddighePetar H. LambrevDimitrios A. GiannakoudakisPetar LambrevDimitrios A. VrachatisPetar OzretićDimitrios BikiarisPetca Razvan-CosminDimitrios KapsokalyvasPetek KorkusuzDimitrios KaragiannakisPeter A. AjibadeDimitrios KontogiannatosPeter A. SlominskyDimitrios KouretasPéter Ábrányi-BaloghDimitrios P. BogdanosPeter AbujaDimitrios PanagopoulosPéter ÁcsDimitrios PapoulisPeter AntalDimitrios PatouliasPeter AskjaerDimitrios SioutopoulosPeter BackxDimitrios SkoufiasPeter BoagDimitris A. GougoulisPeter BrossDimitris EmfietzoglouPeter C. GainesDimitris KardassisPeter Charbel IssaDimitris P. PapadopoulosPeter ChrenekDimitris TatsisPeter CrispDimitris TsikasPéter CsécseiDimitry Arkadievich AfonnikovPeter DurdikDimitry SchepetovPeter EngelmannDimitry VasilyevPeter GänglerDimosthenis KizisPeter GoedegebuureDina AbdellatifPeter GöttleDina DeynekoPeter GraumannDina FarrakhovaPeter GuttmannDina KassemPeter H KingDina V. DudinaPeter HauDinara KhayrutdinovaPeter HauserDinesh BabuPeter HemmerichDinesh BhalothiaPeter HuberDinesh Kumar VermaPeter JanssensDinesh MittalPeter JarcuskaDinesh UpadhyaPeter John JervisDineshkumar KandasamyPeter JonesDingbang MaPeter KaoDingding RenPeter KaplanDingying ZhouPeter KerrDinithi PeirisPeter KoulenDino AquilanoPeter KrugDino LeporiniPeter KubiszDino LuethiPéter L. KanizsaiDinora F. González EsquivelPeter LarssonDiogo Alves GalicoPeter LaufDiogo Onofre SouzaPeter LipkeDionysios ChartoumpekisPeter LittleDipak BanerjeePeter LuntDipak Kumar SahooPeter LyonsDipak PanigrahyPeter M. AndersonDipak RanaPeter MachnikDipali SrivastavaPeter MarotiDipanjan BasuPeter MassányiDipankar AshPeter MikušDipankar RoyPéter MonostoriDipayan BosePeter NagyDipto SinhaPeter O'ConnellDirk BellstedtPeter PaalDirk GeertsPeter ParsonsDirk HenrichPeter Physick-SheardDirk HubmacherPeter PikoDirk MontagPeter Ping LinDirk RadesPeter PolčicDirk ReinholdPéter PoórDirk SpitzerPeter R LaityDirk SteinritzPeter RiedererDirk TheilePeter RoseDirk TischlerPeter RuthDisha MalaniPeter SchemmerDisha SharmaPeter SchuDishary BanerjeePeter SchubertDisheet ShahPeter SetlowDitte C. AndersenPeter Sol ReinachDivya KalraPeter StadlerDivya MishraPeter TimashevDivya SinhaPeter TimminsDivya VimalPeter TomčíkDixit BhalaniPeter UppstuDjanaguiraman MaduraimuthuPeter Van DamDjawid HashemiPeter Van LuijkDjenisa RochaPeter Van VeldhuizenDmitri KramerovPeter VeranicDmitri S. KudryashovPeter ZhukovskyDmitri ShchekochikhinPēteris TretjakovsDmitri SobolevPetia DatcharyDmitrii A. PavlovPetko AlovDmitrii A. TanianskiiPetr BenesDmitrii KorzhevskiiPetr BušekDmitrii LevitskyPetr ChlapekDmitrii MazhukinPetr DráberDmitrii S. MaltsevPetr FilipDmitrii ShekPetr KadlecDmitris XirodimasPetr KozlíkDmitriy ShcherbakovPetr MlejnekDmitriy SmolenskyPetr NachtigalDmitriy V MazurovPetr O. IlyinskiiDmitry A. GorbachevPetr SedláčekDmitry A. LioznovPetr SergievDmitry A. SuplatovPetr SherinDmitry A. TikhonovPetra AmchováDmitry B ZorovPetra De GraafDmitry D. ZhdanovPetra DunkelDmitry E. PostnovPetra HartmannDmitry GruzdevPetra HeffeterDmitry I. KlimovPetra I. LorenzoDmitry KarpovPetra ImhofDmitry KhochenkovPetra J. KlugerDmitry KiesewetterPetra KoraćDmitry KostyushevPetra KosutovaDmitry LapinPetra KotnikDmitry M. ShayakhmetovPetra M. WiseDmitry NamgaladzePetra PopovicsDmitry PelageevPetra SchlingDmitry S. MikhaylenkoPetra VaskoDmitry SelishchevPetras PrakasDmitry SergeevPetre IonitaDmitry ShulgaPetrick Ramesh A. L. K. PeriyasamyDmitry StetsenkoPetro E. PetridesDmitry VerbenkoPetronella KettunenDmitry WainsteinPetros DinasDmitry YarmolinskyPetros KolovosDmitry Yu. IvkinPetros RafailidisDmitry ZhdanovPetros SountoulidesDmitry ZimnyakovPetrova BoryanaDo Kyung KimPetya BergerDoaa H. ZineldeenPetya ChaveevaDobrin VassilevPeyman EbrahimiDobromira ShopovaPhil ChilibeckDoina A. TodeaPhilip C TrackmanDoina LuticPhilip DoganisDoina Olga Afina DimoniePhilip EffraimDolly KhonaPhilip El-DuahDolores Gutierrez-AlanisPhilip HewittDolores Pérez-SalaPhilip HinchliffeDolores R. SerranoPhilip HublitzDolunay GülmezPhilip J. HastingsDomagoj GlavinaPhilip J. YoungDomagoj VidosavljevicPhilip James FarabaughDomenica D'EliaPhilip OwensDomenico AielloPhilip R. MuskinDomenico Azarnia TehranPhilip SarajlicDomenico CerulloPhilip SerwerDomenico CucinottaPhilip W. WertzDomenico DalessandriPhilipp ChetverikovDomenico De BerardisPhilipp E. ChetverikovDomenico De RasmoPhilipp LutzDomenico FrancoPhilippe B. WilsonDomenico GalantePhilippe BielefeldDomenico GarozzoPhilippe BillialdDomenico IacopettaPhilippe BrabetDomenico LioPhilippe Brunet De La GrangeDomenico MaioranoPhilippe BuletDomenico MontesanoPhilippe De DeurwaerdereDomenico PalumboPhilippe DeschampsDomenico PrisaPhilippe GossetDomenico RussoPhilippe Herman-BausierDomenico ScaramozzinoPhilippe LecomteDomenico TricaricoPhilippe LewalleDomenico VentrellaPhilippe MarteauDominic WalesPhilippe MongetDominik DłuskiPhilippe NgheDominik GeierPhilippe PourquierDominik JańczewskiPhilippe R KoninckxDominik KoszelewskiPhilippe RascleDominik OberthürPhilippe RobertDominik PoradowskiPhilippe RondeauDominik S. SkibaPhilippe SupiotDominik SaulPhilippe-Henri SecretanDominik SkibaPhillip DettleffDominik TomaszewskiPhillip KoechDominika BednarczykPhimon AtsawasuwanDominika Drulis-FajdaszPhiwayinkosi V. DludlaDominika GłąbskaPhlip HaselDominika Idziak-HelmckePhlippe LefebvreDominika MalińskaPhotios A. AnninosDominika SalamonPhu-Tri TranDominika SiwiecPia Lopez-JornetDominika SkolmowskaPia M. VidalDominika ZającPia RagnoDominique DebannePia Rengtved LundegaardDominique HeymannPier Adelchi RuffiniDominique ModrowskiPier Carlo RicciDominique PadovaniPier Cesare CapponiDomiziano TarantinoPier Luigi AntignaniDona SinhaPier Luigi FioriDonald CameronPier Nicola SergiDonald PoirierPier Paolo PiccalugaDonald S. ProughPier Vitale NuzzoDonald SikazwePiera Anna MartinoDonald SteinPierangela TottaDonald WlodkowicPierfrancesco FrancoDonat AlparPiergiorgio La RosaDonatella BattagliaPierluca GalloniDonatella Degl'InnocentiPierluigi CarratuDonatella Delle CavePierluigi ScaliaDonatella MilaniPierluigi StipaDonatella PietrellaPiero BaroneDonatella TaramelliPiero CossuDonato ColangeloPiero FerollaDonato D’AgostinoPiero PaladiniDonato GemmatiPiero PortincasaDonato GerinPierre BarraudDonato MontiPierre BongrandDonato RigantePierre CappyDonato SquillaciPierre CornelisDong AnPierre De MeytsDong ChenPierre DudouetDong HanPierre Fernand NeuenschwanderDong Jun LimPierre JacobDong Jun ParkPierre LafiteDong LiuPierre Le BarsDong Seok KimPierre LeclercDong Wook ShinPierre MoffattDong Woon KimPierre MordantDong ZhaiPierre RougeDong ZhangPierre RustinDongbao ChenPierre TennstedtDongbo XuPierre VacherDongchul KangPierre VogelDongdong CaoPierre-Adrien PayardDongdong WangPierre-Aurélien BeuriatDongdong XuPierre-Emmanuel JoubertDongho KangPierre-Olivier AngrandDong-Hoon JeongPierre-Olivier VidalainDonghyun KimPierre-Simon JoukDonghyun LeePierre-Yves Collart-DutilleulDong-Joo CheonPierre-Yves RescanDong-Keun SongPiet Van Der AarDonglei SunPieter De LangeDonglin YangPietro AsproniDongmei LiPietro CacialliDong-Ming HuangPietro CarotenutoDongqing WeiPietro CiancagliniDong-Seol LeePietro FormisanoDongsheng LeiPietro G. TiscarDongsu ChoiPietro GalinettoDongwei KangPietro MaranoDongwei ZhangPietro MazzeoDong-Wook HanPietro Paolo VitielloDongying LiPietro RanieriDongyue XinPietro ScicchitanoDongyun LeePijus BarmanDongzhu MaPilar CacheiroDonika G. IvanovaPilar GiraldoDonna D'AgostinoPilar Gomez-GarreDora Allegra CarbonePilar LeguaDóra K. MenyhárdPilar M DomínguezDora MehnPilar Rivero-RíosDora V. KaloyanovaPilar Rodríguez-PomboDora ValenciaPilar Sánchez GómezDora-Luz FloresPina ZiranuDoreen LauPinaki P MisraDoria FilipponiPinar Uysal-OnganerDorian MigońPinelopi ArvanitiDorianna SandonàPinelopi SamaraDoriano FabbroPing ChenDoriano LambaPing FangDorin IonescuPing K. YipDorinda Marques Da SilvaPing LiDoris ChenPing SuDoris KrabelPing XieDoris KretzschmarPing ZhangDoris Mangiaracina BenbrookPing ZhengDoris Rosero-GarciaPing ZhouDoris RusicPing-Chih HsuDorit AvniPing-Hui TsengDoron ShkolnikPing-Li SunDoron TeperPingping HanDorota BabilasPingxian ZhangDorota DymkowskaPin-Wen ChenDorota Fopp-BayatPio ZeppaDorota Gołąbek-SulejczakPiotr AlsterDorota JuchnoPiotr BatysDorota KwiatkowskaPiotr BojarskiDorota NieoczymPiotr BruździakDorota Piasecka–KwiatkowskaPiotr CysewskiDorota Rutkowska-ZbikPiotr DąbrowskiDorota RybaczekPiotr DobrowolskiDorota SulejczakPiotr DobrzynskiDorota Tichaczek-GoskaPiotr DuchnowskiDorota Tomaszewska-ZarembaPiotr DzięgielDorota WłogaPiotr F. J. LipińskiDorota Wójcik-PastuszkaPiotr FormanowiczDorota WojniczPiotr GawdaDorota WrześniokPiotr GębaraDorota ŻelaszczykPiotr GolecDorota Zięba-PrzybylskaPiotr IwaniukDorothea TsekouraPiotr K. KrajewskiDorothee KieferPiotr KaminskiDouglas C. HooperPiotr KapustaDouglas G BrownfieldPiotr KiełbasińskiDouglas G. GoodinPiotr KonopelskiDouglas G. WalkerPiotr KoprowskiDouglas LeamanPiotr KraskaDouglas Maya-MilesPiotr KuropkaDouglas Rafaele Almeida SilveiraPiotr LulińskiDouglas Silva DominguesPiotr MatczakDragan NikolicPiotr MichelDragana BartolićPiotr MorasiewiczDragana KomnenovPiotr MuchaDragana LazarevićPiotr Oskar CzechowskiDragana MaricPiotr OstaszewskiDragana RadoševićPiotr PoznanskiDragana Samardžija NenadovPiotr RatajczakDragana Vasic AnicijevicPiotr RobakowskiDragica SelakovicPiotr RozentrytDrago BesloPiotr SalachnaDrago DolinarPiotr SetnyDrago JelovacPiotr SleczkowskiDragos Catalin JianuPiotr StefanowiczDragos CretoiuPiotr ŚwiątekDragos HorvathPiotr SzwedaDragos MedianPiotr SzymczykDrashya SharmaPiotr WlazDražan KozakPiotr WlaźDrenka TrivanovicPiotr WychowańskiDrew NassalPiotr ZabierowskiDritan SiliqiPiotr ZielenkiewiczDriton VllasaliuPipsa HirvaDror MalkaPishan SungDrozdstoy StoyanovPiumi LiyanageDrucilla G. RobertsPiya TemviriyanukulDu YuchunPiyush BaindaraDuane P. GrandgenettPiyush SharmaDuangjai TungmunnithumPiyusha PagareDubravka Negovetić-VranićPlacido BruzzanitiDubravka Švob ŠtracPlacido NavasDubravko HabekPlamen KirilovDuc Cuong BuiPloenthip PuthongkingDuc-Cuong BuiPo-An HuDuc-Thang VoPo-Hsien LiDulari JayawardenaPo-Jen HsiaoDumith JayathilakaPolavarapu Bilhan Kavi KishorDumitru CiolacPolina GundorovaDumitru Constantin-TeodosiuPolina Yu. VolkovaDuncan ThomasPoliraju KalluruDung Nguyen TrongPolosa Paola LoguercioDunja Maria Baston-BüstPolydefkis HatzpolousDunja ŠamecPolyxeni NikolakopoulouDuo ZhangPoming ChowDuran CanatanPonlapat RojnuckarinDurga Praveen MekaPonnuraj Nagendra PrabhuDuried AlwazeerPonnuswamy VijayaraghavanĐuro JosićPooja DixitDušan BranýPooja MandkeDušan CmarkoPooja SinghDušan DimićPoonam C. SinghDušan MalenovPoonam PhalakDusan RackoPoornananda M. NaikDusan VelkovicPopa Stefan-LucianDušanka A. PopovićPornsiri PitchakarnDusanka Savic-PavicevicPo-Ting WuDushyant DamaniaPoulami ChatterjeeDusica PahorPoulami SarkarDusko BlagojevicPoushali DasDuško LainščekPouya DiniDustin GreenPovilas IgnataviciusDuygu AğagündüzPo-Wen ChenDuygu Sari-AkPo-Yuan KeDyana OdehPrabakaran MayakrishnanDylan BurgerPrabakaran NagarajanDziedzic ArkadiuszPrabath G BijuDzung H. DinhPrabhakar BusaE Scott SillsPrabhakar OrsuE. A. SafonovaPrabhakaran SoundararajanE. A. SmirnovaPrabhanjan GiramE. E. TzirtzilakisPrabhat K. TalukdarE. F. KolesanovaPrabhu PonnandyE. M. PedonePrabhu ThirusanguE. P. BarantsevichPrabhugouda SiriyappagouderE. Sylvester ViziPrabodh SadanaE. T. Sakamoto-HojoPrabodhika MallikaratchyE. V. Savvateeva-PopovaPrabu GnanasekaranEarl PrinslooPrachi GuptaEbba BråkenhielmPrachi UmbarkarEberhard SchlickerPradeep ChopraEbrahim BanitalebiPradeep DwivediEbubekir YükselPradeep KoppoluEdel Perez-LopezPradeep KumarEder Carlos Rocha QuintãoPradeep Kumar BollaÉder Ricardo PetryPradeep Kumar PandaEdgar CambazaPradeep LamichhaneEdgar Gustavo Ramos-MartínezPradeep R. VaradwajEdgar Gutiérrez-FernándezPradeep RamalingamEdgar Julian Paredes-GameroPradeep SinghEdgar López-LópezPradeep VaradwajEdgar RuedaPradeepkiran Jangampalli AdiEdgar Soria-GómezPradeesh SivapalanEdgar Tavares-Da-SilvaPradip DeEdgars ElstsPraful R. NairEdiane SilvaPrafull SalviEdijs VaversPragneya DemeEdina A. Wappler-GuzzettaPragya TiwariEdina Gyukity-SebestyénPrajish IyerEdina PandurPrakash D. NallathambyEdina PolettoPrakash G. KshirsagarEdio MaldonadoPrakash GangadaranEdirisingha Dewage Nalaka Sandun AbeyrathnePrakash KharelEdit CsapoPrakash LingasamyEdit HermeszPrakash M Gopalakrishnan NairEdit HorváthPrakash PatilEdith HummlerPrakash RaiEdmar Oliveira-FilhoPrakash VenglatEdmund KoziełPramit ChowdhuryEdmundo Bonilla GonzálezPramod Kumar SahuEdna Regina AmantePramod RamtekeEdna YamasakiPramod SubediEdoardo Marco NapoliPranabananda DuttaEdoardo MonfriniPranay SrivastavaEdoardo PeroniPranjali NaikEdor KabashiPranothi MulintiEdouard GerbaudPranshu SahgalEdson SantanaPrapansak SrisapoomeEdson SilvaPrasad ParchuriEdson Silva FilhoPrasad TrivediEduard BardajíPrasanna KattiEduardo CilliPrasanna Kumar SanthekadurEduardo CiresPrasanna NeelakantanEduardo Cyrino Oliveira-FilhoPrasanna RamaniEduardo Domínguez MedinaPrasannavenkatesh DuraiEduardo Fuentes QuinterosPrasanta K SubudhiEduardo González-ZamoraPrasanth ManoharEduardo Gutiérrez-AbejónPrasanth PuthanveetilEduardo GuzmánPrasenjit SahaEduardo José Fernández RodríguezPrashant KaushikEduardo Larriba TornelPrashant KumarEduardo ListikPrashant NighotEduardo López-UrrutiaPrashant RajbhandariEduardo Montoya-DiazPrashant S. DeoreEduardo OsunaPrashant SinghEduardo Rivadeneyra-DomínguezPrashant TaraleEduardo SalidoPrashanth Kumar Bhusetty NageshEduardo TizzanoPrashanth SuravajhalaEduardo Yoshio NakanoPrashanth ThevkarEdvard EhlerPrateek ShettyEdvard SmithPrathap SomuEdvardas DanilaPratheepa RasiahEdward BaidooPrathima Perumal ThirugnanasambandamEdward ChernPratibha KumariEdward EisensteinPratibha PandeyEdward J. FoxPratik JoshiEdward KrzyżakPratima GuptaEdward LoukopoulosPratyush RoutrayEdward MunteanPratyusha GhantaEdward N. HarrisPraveen AranyEdward R. SauterPraveen BarrodiaEdward StephensPraveen Gopalakrishna PaiEdward T EngPraveen KogantiEdwige VoissetPraveen Krishna VeerasubramanianEdwin WanPraveen KumarEdyta BiskupPraveen P. N. RaoEdyta Gendaszewska-DarmachPraveen P. SinghEdyta MakuchPraveen VasudevanEdyta Marta BorkowskaPravej AlamEdyta PasickaPravin B. SehgalEdyta ReszkaPravin IngoleEero CastrenPravin KaldhoneEffendi Tri BahtiarPravin ParajuliEfrat KesslerPrawej AnsariEfstathia K KapsogeorgouPredrag CudicEfstathios MichalopoulosPredrag KojicEfstratios StratikosPredrag NovakEgidio IorioPreetish Kadur Lakshminarasimha MurthyEgizia FalistoccoPreetish MurthyEgon HeusonPrem Chandra PandeyEgor DzyubenkoPrem Haridas MenonEgor OsidakPrem KushwahaEhab AlshamailehPrem Lal KashyapEhsan RazmaraPrema Latha MallipeddiEiichi KumamotoPremashis KarEiichi MomotaniPremila LeiphrakpamEiichi SatoPrenilla NaiduEiichi TokudaPrerna DabralEiichi WatanabePriit VäljamäeEiichiro NagataPrim B. SinghEiji ArakawaPrimoz RozmanEiji KiyoharaPriscila BarbosaEiji KubotaPriscila Krebsbach KandalskiEiji MiyoshiPriscila Pereira Silva-CaldeiraEijiro AkasakaPritam GangulyEijiro JimiPritam SadhukhanEiki KoyamaPritha MajumderEileen MaturiPrithwi GhoshEiman AleemPriti Prasanna MaityEinas YousefPritpal KaurEinat Shemesh-MayerPriya BalasubramanianEirini KaramichaliPriya Ranjan SahooEirini MartinouPriyadarshi ChakrabortyEirini PanagopoulouPriyam PatelEirini TrompoukiPriyanka LahiriEirini VasarmidiPriyanka MittapellyEisa TahmasbpourPriyanka SandalEishi AshiharaPriyanka SinghEishin MoritaPriyanka SiwachEisuke KatoProkopios GeorgopanosEitan FibachProsanto Kumar ChowdhuryEitaro KodaniProsenjit MondalEjaife AgbaniProttoy HasanEkaitz Errasti-MurugarrenProvvidenza AbruzzoEkarat MeechoowasPrzemyslaw ChmielewskiEkaterina A. LitvinovaPrzemysław CzeleńEkaterina A. TrifonovaPrzemysław D. GilunEkaterina BadaevaPrzemysław DanekEkaterina BalaianPrzemysław DudaEkaterina BaranovaPrzemyslaw GrudnikEkaterina FinkinaPrzemysław KołodziejEkaterina GeorgievaPrzemysław Ł. ZalewskiEkaterina GrafskaiaPrzemysław NucEkaterina I. MakarovaPrzemysław PączkowskiEkaterina KoltsovaPrzemyslaw PlonkaEkaterina KozuharovaPrzemysław PłonkaEkaterina L. TrukhanovaPrzemyslaw SitarekEkaterina N. LyukmanovaPrzemysław SołekEkaterina NaumenkoPrzyklenk KarinEkaterina RudnitskayaPu DuanEkaterina ShchegravinaPu XiaEkaterina SukhovaPuey OunjaiEkaterina V ZubarevaPugazhendhi SrinivasanEkaterina VorotelyakPui Wah ChoiEkaterina Yu. BezsudnovaPumtiwitt MccarthyEkaterina YurchenkoPuppo FrancescoEkaterina-Michaela TomouPuran S. BoraEkaterini ChatzakiPurushoth EthirajEkrem OzluPurushothaman NatarajanEkrem ÜnalPushp Sheel ShuklaEl Chérif IbrahimPushpa JoshiEl Khalil CherifPushpa Raj JoshiElad LaxPu-Ting DongElaine A. GayPuvanalingam AyyaduraiElaine DunlopPyung-Hwan KimElaine GavioliQaisar MahmoodElaine NortonQaisar MaqboolElaine Yee Lin ChungQaiser RiazElbini-Dhouib InesQamar AbbasElcio GuimaraesQamar AhmedEldon RajQamar U ZamanEleanor LeungQari Muhammad ImranEleftherios Terry FarmakisQazi Mohammad Sajid JamalEleftherios TouloupakisQi CuiElena AlkalaevaQi GaoElena AmbrosiniQi QiaoElena AndreucciQi WangElena ApostolovaQi XiangElena B. AverinaQian ChenElena BeloglazkinaQian LiElena BeltramoQian QinElena BernadQianbin ZhangElena BochukovaQianfen WanElena BoggioQiang FuElena BonciuQiang HuangElena BottaQiang LiuElena CantoneQiang RuanElena CassinerioQiang TongElena ChavesQiang WangElena ChekmenevaQiang XieElena ChiricozziQiangying YiElena ChugunovaQianqian SongElena CicheroQiantao ZhengElena CodriciQianyun XiElena CsernokQiao ZhangElena D BazhanovaQibin MaElena De CarolisQicheng FengElena De FeliceQie ShuoElena Di PierroQi-En WangElena DilonardoQien YangElena DinteQifei LiElena DonettiQikui WuElena EfremenkoQiliang HuangElena ErmilovaQimin HaiElena EscubedoQin XuElena F. ShevtsovaQin ZhaoElena FedorovaQing ChenElena FicoQing Rong ChenElena Gallo MacfarlaneQingbin CuiElena GiordanoQingbo KeElena GiorgiQing-Fu SunElena GirichQinghua CaoElena GrakovaQinghui WangElena GultyaevaQingjun PanElena I. KovalenkoQingming FangElena I. RyabchikovaQingsen ShangElena IsakovaQingwei LiaoElena Iuliana BîruQingxin YaoElena Ivanovna ShramovaQingxiong YuElena Jiménez-MartíQingyu ZhouElena KaschinaQingyun YanElena KashubaQinqin HanElena KovalenkoQinyu SunElena L. VodovozovaQinzhe WangElena LastraioliQiong GuoElena Lo PrestiQiong XuElena M. KondaurovaQiong ZhangElena MakarovaQiongyao PengElena ManakovaQiongyu LiElena McbeathQiqin WangElena MitsiQiumin LuElena MontiQiushi WangElena N. IkkonenQiuwang ZhangElena NiccolaiQixuan ZhengElena NikitkinaQiyan JiangElena NikolskayaQi-Yin ChenElena PalmaQiyu TianElena PiccininQize ZhangElena PomariQuang Minh NgoElena Ponomarenko Quanjiu WangElena PopugaevaQuanwei ZhangElena Porumb-AndreseQuentin PerraudElena PudovaQuentin ThommenElena RamírezQun CaoElena Ramírez-ParraQun HuoElena RampazzoQun TangElena RezusQun ZhouElena Rodríguez-SánchezQunxiang OngElena RogozinaQunzhou ZhangElena S. BelykhQurban AliElena SalviQuyen Vu ThiElena SarropoulouQuynh Anh LeElena SavvateevaQuynh Nhu DinhElena ShakhtshneiderR. A. IlyasElena ShashovaR. Dan LittleElena ShuyskayaR. Rama SureshElena SorokinaR. William CaldwellElena SvirshchevskayaRabab MohammedElena TarcaRabia AyubElena TchetinaRabia FaridiElena TrombettaRabiul IslamElena TsourdiRachana D. ShahElena V. EremeevaRachana PradhanElena V. GerasimovaRachana TrivediElena V. MitroshinaRachel AustinElena V. OrlovaRachel Bar-ShavitElena V. SvirshchevskayaRachel KalmannElena V. ZemlyanskayaRachel L. WashburnElena VendraminiRachel LiElena Victorovna DeinekoRachel OldershawElena Yu. TupikinaRachel SmithElena ZakharovaRachele GarellaElena ZavyalovaRachele RossiElena ZharkikhRachid HsissouElena ZhitovaRachmat HidayatElena ZhurikovaRachy AbrahamEleni BozinouRade M. BabićEleni DovolouRadek MalikEleni MavrogonatouRadek PohlEleni PalliRadhia M’KacherEleonora AlfinitoRadhia M'KacherEleonora CataldoRadhika PuttaguntaEleonora CianfloneRadim VrzalEleonora FavuzzaRadivoje JevtićEleonora LaiRadka Svobodová VařekováEleonora LoiRadka VaclavikovaEleonora NuceraRadomir JasinskiEleonora PorcuRadomir SavićEleonóra SpekkerRadoslaw B. MaksymEleonora TimperiRadosław DembczyńskiEleonora VirgilioRadoslaw DrozdEleonore FröhlichRadosław KempińskiEleuterio A. Sánchez-RomeroRadoslaw KitelElham AhmadianRadosław KujawskiElham KayvanpourRadu Adrian Crisan-DabijaElham KezarooniRadu AlbulescuElham NikbakhtRadu Claudiu FierascuElia Diego-GarcíaRadu HristuElia GattoRadu Liviu SumalanElia RanzatoRadu MafteiElia StahlRadu MineaEliana CostanzoRadu RacovitaEliana Dell'OlmoRadu TamaianEliana LeoRaed Abu-ReziqEliana MariñoRaed AlkowniEliana PintusRaeesa GupteEliano CascardiRafael Antón-HerreroElias A. El-HabrRafael Baltiérrez-HoyosElias A. KouroumalisRafael Camacho-CarranzaElias ChristoforidesRafael Casuso-PérezElias DrakosRafael CoveñasElías Herrero-GalánRafael CuestaElias SakellisRafael DariolliEliecer Jimenez CharrisRafael EscateEliel Ruíz-MayRafael Fernandez-BotranElif Tekin IftarRafael Fernández-MartínElif YildizRafael G. Campos-MontielElijah PetersenRafael Guillén-BejaranoElikanah Olusayo AdegokeRafael MorcilloElisa AndresenRafael Moreno-LunaElisa BelluzziRafael Reis DominguesElisa BindaRafael Ríos-TamayoElisa CairrãoRafael Salto-GonzalezElisa CeccheriniRafael SamaniegoElisa FrezzaRafael Scaf De MolonElisa GarzoRafael Tapia-RojoElisa GiovannettiRafaela VasiliadouElisa Gonzalez-DominguezRafał BielasElisa HerraezRafal BuldakElisa LozanoRafal KaminskiElisa MartellaRafal KocylowskiElisa MenegattiRafal KowerdziejElisa OltraRafal OgorekElisabet Navarro-TapiaRafał OgórekElisabete M. S. CastanheiraRafal P. PiprekElisabeth BillardRafal RakoczyElisabeth G. VichayaRafał StarzyńskiElisabeth HofmannRafal ZielinskiElisabeth JacobsenRafel M. PrietoElisabeth PinartRaffaele BoniElisabeth SeebachRaffaele CapassoÉlisabeth TraiffortRaffaele De FrancescoElisabeth WyartRaffaele FrazziElisabetta AntonelliRaffaele IevaElisabetta BenedettiRaffaele PalmirottaElisabetta BucalettiRaffaele PastoreElisabetta CanettaRaffaele RiccoElisabetta CatalaniRaffaele SimeoliElisabetta CerbaiRaffaella AdamiElisabetta FerraraRaffaella CoccoElisabetta FranchiRaffaella ComitatoElisabetta GrilloRaffaella De PaceElisabetta MartiniRaffaella PetruzzelliElisabetta MeacciRaffaella PippaElisabetta MennaRafi UllahElisabetta MurruRafiq LoneElisabetta OddoRagavanantham ShanmugamElisabetta PalamaRaghav KaliaElisabetta ProfumoRaghavendra YadavalliElisabetta RazzuoliRaghbendra Kumar DuttaElisabetta TabolacciRaghu P. KataruElisabetta ZanolettiRaghu VemugantiElisavet GrouziRaghunath SinghElisavet StavropoulouRaghunathreddy AnuguElise Des LignerisRaghupathy VengojiElise Gomez-SanchezRaghuram KandimallaElise M. A. SlobRaghuveer SinghElise SchieckRaghuveera GoelElisenda Alsina-SanchisRaghvendra BoharaEliseo Amado-GonzálezRaghvendra DubeyEliseo EugeninRaheleh RoudiElissavet NtemouRahul ChandnaniEliza OpreaRahul D. JawarkarElizabeth A CooperRahul DattaElizabeth A. WhitcombRahul GauttamElizabeth Bartolak-SukiRahul KanumuriElizabeth C. CottrellRahul KavtheElizabeth Carvajal-MillanRahul KrishnanElizabeth ChenRahul KumarElizabeth CzislowskiRahul Mahadev ShelakeElizabeth FernandesRahul SanawarElizabeth GibsonRahul SrinivasanElizabeth L. KordyumRaiko BlondonnetElizabeth M. McneillRaimondas ŠiukštaElizabeth Podlaha-MurphyRaimondo GaglioElizabeth R. GilbertRainer De MartinElizabeth S. FernandesRainer MikschElizabeth SewardRais AnsariElizabeth ShumbayawondaRaj BhullarElizabeth SokolRaj KarthikElizabeth ThomasRaj KumarElizabeth VafiadakiRaj SewduthElizabeth WeretilnykRaj YadavElizabeth WoodwardRaja Ben-LaouaneElizabeth-Barbara TatsiRaja GanesanElizaveta FofanovaRaja Gopal Reddy MooliElka R. GeorgievaRaja VeerapandianElke ButtRajaguru PalanichamyElke HammerRajamma MathewElla AtlasRajan ChoudharyEllen H. KangRajan LamichhaneEllen M. WesterhoutRajanikanth VangipurapuElli KoskelaRajashekhar GangarajuElliot M. LevineRajat WaliaElliot R. BernsteinRajbir SinghElma BaronRajdeep BanerjeeElmar KirchesRajeev SinglaElmer MauritsRajeev YadavElmina Mammadova-BachRajender MotianiElnaz ZeynalooRajendra AcharyaEloá Cristina Passucci AmbrosioRajendra KurapatiElod NagyRajendra Prasad SettemElodie DesroziersRajendran Jc BoseElodie LafontRajendran SelvakesavanEloisa RomanoRajesh AminEloy FernandezRajesh AngireddyEloy Navarro-LeónRajesh BhardwajElrashdy M. RedwanRajesh HaldharElsa C. ChanRajesh KarElsa M. GonçalvesRajesh KasamEl-Saadony MohamedRajesh P. ShastryElsayed E. HafezRajesh PandeyElsayed MansourRajesh SharmaElsayed MehanaRaji Paul GrewalElvira E. ShultsRaji Rajesh LeninElvira PantusoRajia BahriElvira RozhinaRajith Singh MananElvyra JarienėRajiv K. PruthiElwy AshourRajiv Kumar JhaEly Oliveira-GarciaRajiv Kumar KarElżbieta BudziszRajkumar DevasenathipathyElzbieta Grza̧DkaRajkumar P. ThummerElżbieta GrządkaRajkumar VutukuriElzbieta JandaRajna MinićElžbieta Jankovska-BortkevičRajneesh JaswalElzbieta JankowskaRajneesh PaliwalElzbieta KlewickaRajneesh SrivastavaElzbieta KolaczkowskaRajni VermaElzbieta Kompanowska-JezierskaRajnish S. DaveElżbieta Łodyga-ChruścińskaRajtilak MajumdarElżbieta Łopieńska-BiernatRaju Senthil KumarElżbieta Lorenc-KociRaju TimsinaElżbieta Pac-KożuchowskaRakesh AmetaElżbieta PatkowskaRakesh BhaskarElżbieta Poniedziałek-CzajkowskaRakesh GanjiElżbieta PoniewierkaRakesh K. SindhuElzbieta SalinskaRakesh K. TiwariElzbieta SarnowskaRakesh K. UpadhyayElżbieta Studzińska-SrokaRakesh SinghElżbieta SuchowilskaRakeshkumar B PatelElzbieta SzaconRakkiyappan ChandranElżbieta TrafnyRaksawan DeenonpoeElżbieta Wałajtys-RodeRalf AnkenElżbieta WyskaRalf BlosseyElzbieta ZieminskaRalf BraunEma OzakiRalf GutzmerEmad A. AhmedRalf KircheisEmad Al-DujailiRalf OelmullerEmad MadyRalph FrancesconeEman FayadRaluca DumacheEman KhalifaRaluca Șuică-BunghezEman M. OthmanRaluca Van StadenEman ToraihRaluca-Maria HlihorEmanuel BaltagRam Babu UndiEmanuel Hernández-NúñezRam JiwariEmanuel K. ManesisRam PrasadEmanuel Loeza AlcocerRam SagarEmanuel Sanz-LuqueRamachandran SrinivasanEmanuel StrehlerRamachandran VinayagamEmanuela BerrinoRaman BekarevichEmanuela BostjancicRaman DhariwalEmanuela ChiarellaRamana VakaEmanuela EspositoRamanjaneyulu AllamEmanuela LeonardiRamar ThangamEmanuela PedrazziniRamasamy Prem KumarEmanuela RuggieroRamaswamy KannappanEmanuela StamponeRamces Falfan-ValenciaEmanuele AlbanoRamcharan Singh AngomEmanuele BernardinelliRamendra Pati PandeyEmanuele BobbioRamesh ChingleEmanuele CapraRamesh DhandapaniEmanuele GiurisatoRamesh KandimallaEmanuele MondaRamesh KatariaEmanuele PanzaRamesh KumarEmanuele PravatàRamesh L. GardasEmanuele ScalaRamesh MukkamalaEmel BalogluRamesh PeriasamyEmely VerweyenRamesh RijalEmer FerroRamesh S YadavaEmerson KrockRamhari KumbharEmerson S. BernardesRami S. NajjarEmi HaladjovaRamin HakamiEmiel Van Der VorstRamin RahmaniEmil BulatovRamireddy BommireddyEmil E. KhavkinRamiro Palazón-GarcíaEmil KhisamutdinovRamón A. LorcaEmil MalucelliRamon AyonEmil RudolfRamón Garduño-JuárezEmila SiciliaRamon GonzalezEmile MassimaRamón GuevaraEmili BesalúRamón Moreno-TostEmili CidRamón R. BarralesEmilia GligoricRamón Suárez-RodríguezEmilia IłowskaRamona ClemenEmilia L. ApostolovaRamona D'AmicoEmilia LecuonaRamona DanacEmilia WiechecRamona Gabriela UrsuEmiliano DallaRamona LattaoEmiliano TesoroRamona Năstuță HuzumEmilie Dubois-DeruyRamona PalomboEmilie WidemannRamya BillurEmilija ZdravevaRamya Lakshmi RajendranEmilio CervantesRan WangEmilio MartinesRan ZhangEmilio MartinezRan ZichelEmilio ParisiniRana El RawasEmilio VareaRanajay SahaEmilly Schlee VillodreRanda EshaqEmily CaponeRandal S. StahlEmily GravesRandall G. WorthEmily KnottRandall J. KimpleEmma Adriana OzonRandi VitaEmma BleachRando TuvikeneEmma Burgos-RamosRandy CowlingEmma C. CacciolaRandy DinkinsEmma DesplandRandy NelsonEmma EvergrenRangaramanujam KannanEmmanouil MagiorkinisRanhua XiongEmmanouil MalandrakisRanjeet DhokaleEmmanouil P. BenisRanjini SankaranarayananEmmanouil RizosRanjit DeEmmanuel A. PilaRanjit GuravEmmanuel AndresRanjit ShresthaEmmanuel J FavaloroRanjita SinhaEmmanuel KontomanolisRanjith K. PapareddyEmmanuel M. PapamichaelRanjith KamityEmmanuel MoyseRanjith Kumar ManoharanEmmanuel Silva MarinhoRanjith Kumar RajamaniEmmanuel TaillebourgRanjith KumavathEmmanuel TetaudRanko GacesaEmmanuel ValjentRanko RomanićEmmanuelle BignonRanulfo LemusEmmanuelle BourneufRaphael CiumanEmmanuelle LeratRaphael GollnischEmmy RogakouRaphael GorodetskyEmoke PallRaphael Jay WitorschEmrah CaylkaRaphael ReutenEmre AksoyRaphaël ThuillierEmre KarakusRaphael WurmEmyr BenbowRaphatphorn NavakanitworakulEnade Perdana IstyastonoRappa FrancescaEnas A. Abdel-HadyRaquel AlvesEnbo MaRaquel Bello-MoralesEndler BorgesRaquel BoiaEndre CzeiterRaquel De Cássia Dos SantosEndrit ShahiniRaquel Fernandez-GarciaEnedina Jiménez-CardosoRaquel Guillamat-PratsEng Guan ChuaRaquel HernandezEnnoury AbdelhamidRaquel HerreraEnoch A. MensahRaquel Iglesias FernándezEnoch Odame AntoRaquel L. BernardinoEnol LopezRaquel Moreno LoshuertosEnquan XuRaquel OliveiraEnric I. CanelaRaquel P. F. GuinéEnrica TorrettaRaquel Requejo-AguilarEnrica UrciuoliRaquel Sanabria-De-La-TorreEnrico BraccoRaquibul HasanEnrico CarusoRasa BanieneEnrico De SmaeleRasa PauliukaiteEnrico DoriaRasha A. MsallamEnrico Felice GherloneRashid NazirEnrico GarattiniRashmi RashmiEnrico GherloneRashmi YadavEnrico GiordanRasim AlosmanovEnrico LemmaRasoul GhasemiEnrico MarsiliRasoul MirzaeiEnrico MoroRastislav JendželovskýEnrico OddoneRastko AjtićEnrico PerrinoRatheesh Kumar MeleppatEnrico PierantozziRathinam BalamuruganEnrico RadælliRatko SukaraEnrico RagniRatnadeep MukherjeeEnrico RizzarelliRatnakar TripathiEnrico Vito PerrinoRätsep MatthewEnrique BalderasRaudah LazimEnrique BoldoRaúl Capote-PuenteEnrique ContrerasRaúl Carrera CerritosEnrique Domínguez-ÁlvarezRaul CassiaEnrique Fernandez‐CaldasRaúl Díaz-MolinaEnrique GomezRaul OrtizEnrique Gomez GomezRaul PironaEnrique JaimovichRaunak Kumar DasEnrique Meléndez-HeviaRavi Chakra TuragaEnrique Méndez BolainaRavi Kumar CheedaralaEnrique MontesRavi Kumar KomaravoluEnrique MorettRavi Kumar SharmaEnrique Urrea-MendozaRavi MuralEnshirah Da'NaRavi P SahuEnver Cagri IzguRavi S. LankalapalliaEnxiang ZhangRavi ShahEnza MozzilloRavi Suresh DevaniEnzo IerardiRavi V. MuralEnzo Maria VingoloRavikumar K. JimmidiEoghan MulhollandRavinder KumarEpameinondas LeontidisRavindra DeshpandeEphraim YavinRavindra ThakkarEphrem EngidaworkRavindran Caspa GokulanEraldo Sanna PassinoRawil F. FakhrullinErasmia SidiropoulouRay KuErben JakubRay LuoErcument DiriceRay MccarthyErdem BangiRay OwensErden AtillaRay-Chang WuErez EitanRaymond Scott DuncanErfan OliaeiRaymond WangErfan RezvaniRaymond WongErgun KayaRazieh KebriaeiEri IshikawaRazvan BoiangiuEri S. SrivatsanRăzvan-Ionuț PopescuEric A. AlbrechtRebeca BustoEric AuclairRebeca ValeroEric BeersRebecca A. KeoghEric BenoistRebecca BaillieEric BlommeRebecca GrumetEric BoncompagniRebecca HillEric C. BeyerRebecca K. MartinÉric ChastreRebecca ScheinEric ChristensonRebekka WildEric D. JensenRebuma FirdessaEric De Camargo SmidtRecep LimanEric De GrootRedi RahmaniEric F. KarlinReece Andrew SophocleousEric HuetReed B WicknerEric J. WoolfReem T. AtawiaEric JohnsonReena VsEric MetzenReet KurgEric Minwei LiuReet MändarEric PiletRegina Fölster-HolstEric RuellandRegina M. DayEric S. HoRegina Niñoles RodenesEric Santoni-⁠RugiuRegina P MarkusEric TothRegina PutsErica BazzanReginald GorczynskiErica Di MartinoRegis BobeErica PackRegis MoreauErica SalvatiRégulo López-CallejasErich CosmiReham HamidaErick Sierra-CamposRehan KhanEride QuartaReinaldo Campos-Vargas Campos-VargasErifili P. NikaReinaldo GeraldoErifyli TsagkariReindert DevlamynckErik A. C. WiemerReinhard BauerErik D. HolmstromReinhard BüttnerErik De ClercqReinhard GruberErik FraunbergerReinhard KoppErik L. JensenReinhard LacknerErik RikkerinkReinhard Schweitzer-StennerErik SchoenmakersReinhard WetzkerErik SwensonReinhard ZelenyErik T. YuklReinier AkkermansErika Bevilaqua RangelReisel MillánErika BustosReka BarabasErika CastrignanòRéka MizseiErika CioneReka VarnaiErika López-ArribillagaRemi PeyronnetErika OrbanRemigijus JuskenasErika PalmieriRemigiusz BąchorErika PonziniRemo CampicheÉrika Said Abu EgalRenan DanielskiErin G Reed-GeaghanRenan FalcioniErin H. SeeleyRenan Pedra De SouzaErin RehrigRenata BartesaghiErkan BahçeRenata CiccarelliErlinda TheRenata Coelho Rodrigues NoronhaErna KaralijaRenata De Almeida CoudryErnestina Castro-LongoriaRenata Del CarratoreErnesto Beltrán-PartidaRenata D'IncàErnesto Chigo-AnotaRenata FinelliErnesto GargiuloRenata GiacominErnesto QuesadaRenata GržićErnő ZádorRenata Matraszek-GawronErnst OberlechnerRenata Novak KujundžićErnst WimmerRenata PerlikowskaEros Di GiorgioRenata Rajtar-SalwaErshuai ZhangRenata SahaErsin GümüşRenata SiedleckaErtugrul FilizRenata SistoErwan DelbarreRenata SlomnickaErwan GuyotRenáta ŠvubováErwan MortierRenáta SzabóErwin BrosensRenata TeparićErwin D. SchleicherRenata TisiErzsébet MernyákRenate KunertEsaú Bojórquez-VelázquezRenato Alberto Rodrigues-PousadaEshan KhanRenato SantosEshwar Reddy TammineniRené BuchetEsmaeil Doustkhah HeraghRené FerreraEsmaeil MiraeizRené HägerlingEsmat AliRene HuberEsmeralda DelgadoRené KöffelEsperanza R. MatarredonaRené Massimiliano MarsanoEspinosa EricRené WenzlEspinosa Martín Juan CarlosRenee J. ChosedEsra AǧelRen-Fang ChaoEsrat Jahan RupaRengasamy RamamoorthyEssa M. SaiedRenier Velez-CruzEstanis NavarroRenlei JiEstanislao Nistal-VillánRenpeng GuoEsteban Alejandro Araya-HermosillaRenuka RamanEsteban C. GabazzaRenxun ChenEsteban ManriqueRenying ZhuoEsteban Obrero-GaitánRen-You GanEsteban OrellanaRenzo GuarnieriEstéfani García-RíosRevaz SolomoniaEstefanía Burgos-MorónRex FitzgeraldEstefania ContrerasRex T. NelsonEstefania NuñezReynald GilletEstefany Ingrid Medina-ReyesReza AhmedianEstela Maldonado BautistaReza ArezoomandanEstela SangüesaReza ArjmandiEstelle LeclercReza HaghbakhshEster Beltrán FrutosReza JafariEster GuausReza KiaEstera RintzReza MortazaviEsther C. De JongReza NorooziEsther J. OcolaReza PiriEsther JuliánReza RahimianEsther K. ElliotReza ShafieiEsther L. SabbanRezwan TariqEsther LanteroRiad El KebbajEsther Martínez MartínezRiadh BadraouiEsther MorenoRiadh IlahyEsther OcolaRicardo A. MosqueraEsther U. KadieneRicardo F. FraustoEstrela NetoRicardo González-RezaEswar ShankarRicardo ParrondoEszter BerekmériRicardo SilvaEszter EmriRicardo Vivas-ReyesEszter Mária HorváthRiccardo BientinesiEszter Várkonyi PatakinéRiccardo CastagnaEtana PadanRiccardo FerraciniEthan AndersonRiccardo GhidoniEthan MorganRiccardo IentileÉtienne WeissRiccardo LeinardiEtsuko MiyagiRiccardo MastroianniEtsuro OhtaRiccardo MoiaEttore OlmoRiccardo OrusaEttore SilvagniRiccardo RonzoniEttore VarricchioRiccardo VigneriEuan ParnellRicciarda GalandriniEufrânio N. Da Silva JúniorRicha SachanEugeen VanmechelenRichard AltschulerEugen DomannRichard BinghamEugen Mircea AnitasRichard BowenEugene Haydn WaltersRichard D. NoyesEugene S. KandelRichard Douglas HarveyEugene SurdutovichRichard DrevetEugene SverdlovRichard EgelEugene V. RadchenkoRichard FunkEugenia BezirtzoglouRichard GordonEugénia De AndradeRichard GuyerEugenia Rita LaurianoRichard HamplEugenia V. BroudeRichard HartshornEugenio MartelliRichard HaynesEugenio NotomistaRichard HrabalEugenio ProvenzanoRichard Inho JohEugenio RastelliRichard J. HoneywellEugenio Velasco OrtegaRichard JägerEugenio VilanovaRichard John Bruce FrancisEui Tae KimRichard L. BennettEui-Man JungRichard LebaronEun Jeong ParkRichard LeeEun Ju ShinRichard LuEun Kyung LeeRichard M NilesEun LeeRichard MilneEunhee KimRichard NgombaEun-Ho SohnRichard P. FahlmanEunice CarrilhoRichard ProctorEunju KangRichard S. LeeEun-Ju KoRichard T. PensonEun-Kyung BaeRichard T. TaylorEunsu ParkRichard ThomasEurico CabritaRichard W. BohannonEustace Y. FernandoRichard W. BrillEustachio TarascoRichard W. WongEuthymia VlachakiRichard WangEva AltrockRichard WeissEva Ausó MonrealRicheng ChianEva BagyinszkyRi-Cheng ChianEva BartovaRichie R. BhandarehEva BernalRickey CothranEva Češ̈KováRidwan P. PutraEva CoreyRie YatsunamiEva CunhaRiedemann ThereseEva De LagoRigers BakiuEva DominguesRihui LiEva DražanováRiikka MartikainenEva FenyvesiRika OchiEva Fischer-FodorRiki KurokawaEva Garcia-RuizRikke BirkedalEva KissRikki CorniolaEva KnuplezRikky RaiEva Krieghoff-HenningRiku KawasakiEva KudovaRima HajjoEva M. Medina-RodríguezRina BandopadhyayEva MartinsRina Dhirajlal KoyaniEva NüskenRina HamajimaEva ObermayrRina KamenetskyEva RamosRinaldo PellicanoEva RealiRinesh GodfreyEva S. LiuRintu ThomasEva SapiRio BoothelloEva SlabákováRipa Akter SharminÉva SzabóRipon SarkarEva TsaousidouRishabh DevEva TvrdaRishi NageshanEva TvrdáRishi PalEva ZusinaiteRishikesh GhogareEvaldo FaviRita B. SantosEva-Maria Zangerl-PlesslRita BusinaroEvan R. DelgadoRita CarrottaEvangelia AvramidouRita CasadioEvangelia ChronopoulouRita CortesiEvangelia GoliaRita De ZorziEvangelia KoutelouRita KissEvangelia PantazakaRita MarulloEvangelia StavridouRita MatosEvangelos DaskalakisRita Nogueira-FerreiraEvangelos J Giamarellos-BourboulisRita PavasiniEvangelos KoustasRita Payan-CarreiraEvans M. Nkhalambayausi ChirwaRita Restano CassuliniEvanthia BletsaRita RezzaniEvanthia T. GouveriRita Teixeira-SantosEvdokia SyranidouRita ValenzuelaEvelyn OrsoRitama PaulEvelyne ManetRitesh MenezesEvert F. S. Van VelsenRitesh ThakareÉverton N. AlencarRitesh ThekkedathEvgenia I. DeryushevaRitin SharmaEvgenia Korzhikova-VlakhRitu GuptaEvgenia MitsouRituraj PurohitEvgenia SitnikovaRituraj SinghEvgenii BelykhRitwik DattaEvgenii GusevRivas-Santisteban RafaelEvgenii KuzinRi-Yuan TangEvgeniia StriukovaRizaldy ZapataEvgeniia VikulovaRizkita Rachmi EsyantiEvgeniy ChistyakovRizwan Ali NaqviEvgeniy S. BalakirevRizwan WahabEvgeniya BurkovaRizwanullahEvgeniya OshchepkovaRob LaneEvgeniya V. EfimovaRob T. StrikerEvgeny BarykinRob W. H. RuigrokEvgeny E. AndronovRob W. J. CollinEvgeny E. BezsonovRobert A. CornellEvgeny ErmakovRobert A. KirkenEvgeny LodyginRobert A. NicholasEvguenia BekmanRobert AncuceanuEvich MarinaRobert AxtellEwa A. GrzybowskaRobert B. RuckerEwa Bielczyk-MaczyńskaRobert BiczakEwa Boniewska-BernackaRobert Brawura-Biskupski-SamahaEwa BrzozowskaRobert CarleerEwa CiszkowiczRobert ColvinEwa FlorekRobert D. Mitchell IiiEwa Gibuła-TarłowskaRobert DaileyEwa GrudzińskaRobert DempskiEwa Gurgul-ConveyRobert EckelEwa Hanus-FajerskaRobert EckenstalerEwa IwanekRobert EkartEwa JówkoRobert Emmett KellyEwa Juszyńska-GałązkaRobert F. HalliwellEwa LangeRobert GouldEwa LaskowskaRobert H. RiceEwa MajRobert HertelEwa Miller-KasprzakRobert HicknerEwa NiewiadomskaRobert HuberEwa OkoniewskaRobert J. DimarioEwa Olchowik-GrabarekRobert J. KelmEwa OlędzkaRobert J. MeierEwa Pacholska-DudziakRobert J. MorellEwa PlantRobert J. PalmerEwa RopelewskaRobert J. VandenbergEwa SewerynRobert JarretEwa SikoraRobert K. NaviauxEwa SkarżyńskaRobert KiralyEwa SkwarekRobert KissEwa StodolakRobert KosickiEwa Szczepanska-SadowskaRobert KremerEwa SzczukaRobert L. JarretEwa TomaszewskaRobert L. MeiselEwa Wałecka-ZacharskaRobert LevensonEwa WysockaRobert LiefkeEwald LindnerRobert Llewellyn ClancyEwelina BasiakRobert MacgregorEwelina CelińskaRobert MusiołEwelina CzubackaRobert NashEwelina DziurkowskaRobert NawrotEwelina GoryszewskaRobert OssendorffEwelina GrywalskaRobert PazdroEwelina HallmannRobert PhillipsEwelina HumeniukRobert PosseeEwelina MaculewiczRobert PrillEwelina PerdasRobert RekawieckiEwelina Pogorzelska-NowickaRobert S WelnerEwerton Garcia De Oliveira MimaRobert S. RogersEwoud B. CompeerRobert ScheinmanEyal FridmanRobert SilversEyal ShteyerRobert SplinterEylem Kulkoyluoglu CotulRobert StawskiEytan R BarneaRobert Steven EsworthyEzequiel ÁlvarezRobert T. MalletEzequiel M. Vázquez-LópezRobert T. MeansEzio PortisRobert Van WaardenburgEzzat KhanRobert VigerF. David RodriguezRobert W. ReidF. Scott HallRobert W. RobeyFabao LuoRobert W. SigginsFabian B. FahlbuschRobert WeinzierlFabian C. EmrichRobert WerdehausenFabian FernandezRobert WinklerFabian IlleRobert WojcieszakFabián Martínez-HernándezRobert WojtyczkaFabian MohrRobert Y. L. WangFabian ZanellaRobert YoukerFabiana GeraciRobert ZdanowskiFabiana MorroniRoberta AngioniFabiana PassaroRoberta AntonelliFabiana Pereira Da CostaRoberta CazzolaFabiano CimminoRoberta Elisa RossiFabien CailliezRoberta FenoglioFabien ChevalierRoberta ForlanoFabien DelaspreRoberta FuscoFabien DelerueRoberta GasparroFabien LecailleRoberta GonnellaFabien P. BlanchetRoberta IbbaFabienne BessacRoberta La PianaFabienne LamballeRoberta Magnano San LioFabio BottariRoberta MancusoFabio BrambillaRoberta ModicaFabio CacciapagliaRoberta MoschiniFabio CavaliereRoberta OkamotoFabio CianferoniRoberta ParisFabio CiccaroneRoberta RicciarelliFabio Di NardoRoberta RoccaFábio Filipe Fereira GarrudoRoberta SpadacciniFábio G. TeixeiraRoberta TittarelliFabio Giuseppe MasuccioRoberto AdachiFabio GrizziRoberto BaroneFabio MalavasiRoberto BottinelliFabio MiraldiRoberto Bravo-SaguaFabio NudelmanRoberto BudaFabio PalumboRoberto CalistiFabio PuglisiRoberto CampagnaFabio SantacaterinaRoberto CannataroFabio ScarpaRoberto CarnevaleFabio SciubbaRoberto CastiglioneFabio TasceddaRoberto CesareoFabio TimeusRoberto ChiarelliFábio TrindadeRoberto CiampagliaFabíola Zakia MónicaRoberto CiarciaFabiola ZapataRoberto CodellaFabrice CognasseRoberto ColasantiFabrice ConfalonieriRoberto ColluFabrice HombléRoberto CostaFábris KossoskiRoberto De La Torre-MartinezFabrizio BertelloniRoberto FabianiFabrizio BiundoRoberto Farina De AlmeidaFabrizio CarboneRoberto GiacomelliFabrizio CartaRoberto GiovannoniFabrizio Dal PiazRoberto González-GarduñoFabrizio DamianoRoberto GramignoliFabrizio FontanaRoberto LigroneFabrizio GentileRoberto LópezFabrizio GuzzettaRoberto Lopez-PírizFabrizio MartoraRoberto MacovezFabrizio MontecuccoRoberto MarraFabrizio SignoreRoberto Martínez BeamonteFacundo IbanezRoberto ParedesFada GuanRoberto PastoreFadi CharcharRoberto PerrisFadi KhasawnehRoberto PiergentiliFadia Ali KamalRoberto PiluFadia YoussefRoberto PradoFadilah AleanizyRoberto RamoniFaezeh VakhshitehRoberto RizzoFahad AlamRoberto S FrancoFahad KhanRoberto SalaFahad RashidRoberto ScandurraFahiel CasillasRoberto ScarpioniFahimeh ShahinniaRoberto ScatenaFahmi KhalifaRoberto ScicaliFahri SaatciogluRoberto SorrentinoFai-Chu WongRoberto ToniFaisal AlzahraniRoberto VerucchiFaisal H. M. KouaRoberto ZefferinoFaisal IsmailRoberto Zenteno-CuevasFaiyaz ShakeelRobin KinnelFaiz ArithRobin Lewis CooperFaizul AzamRobson Carlos AlnochFan PanRobson Coutinho-SilvaFan YuRobson De FariasFan ZhangRocchetti VincenzoFan ZhuRochelle DsouzaFang (Rose) ZhuRocío BenitoFang BaiRocio Canovas MartinezFang HuangRocío Llamas-RamosFang JunRocio Nuñez-CalongeFang LiuRocío RedónFang TongRocío Valenzuela-NarváezFang WeiRocío Vila-BedmarFang XieRoddy HiramFang ZhengRoddy WalshFang-Cheng LiangRodica MargaoanFangcong DongRodica Maricela AnghelFangfang QiaoRodica VârbanFangfang QiuRodica-Mariana IonFang-Ying ChiuRodney E. ShackelfordFangyuan ZhangRodney Edwin ShackelfordFani PapagiannouliRodney G. BowdenFanica CimpoesuRodney J. OuelletteFanju MengRodney RitzelFanny Monteil-RiveraRodolfo Abarca-VargasFanwang MengRodolfo NegriFanzani AlessandroRodolfo Ramos GonzálezFarah AsaadRodolfo RedaFarah AzizRodolfo ValtuilleFaraz AhmadRodolfo ZentellaFares E. M. AliRodrigo A LoiolaFarhad HossainRodrigo A. CunhaFarhad Masoomi-AladizgehRodrigo Coutinho De AlmeidaFarhad OstovanRodrigo CunhaFarhad VahidRodrigo Diaz-EspinozaFarhan NabiRodrigo FrançaFarid KhalloukiRodrigo Herrera-MolinaFarid MenaaRodrigo Ligabue-BraunFarid RahimiRodrigo MachadoFarid SroorRodrigo MoralesFarida TripodiRodrigo PessoaFarooq SyedRodrigo Romero-NavaFarshad MirzaviRodrigo TorresFaruk HadziselimovicRodrigo ValenzuelaFarzad KermaniRodrigo Wladimir ValenzuelaFarzad KianersiRoeland BoerFarzad Taghizadeh-HesaryRogelio Rodríguez-SotresFarzana SabirRoger BiringerFarzaneh KordbachehRoger ChongFarzaneh SabbaghRoger E. ThomasFasih IlhamiRoger GrandFatehi FoadRoger JacobsFatema BhinderwalaRoger LengFatemeh Azadegan-DehkordiRoger MooreheadFatemeh DabbaghRoger Olofsson BaggeFatemeh Ghorbani-BidkorpehRoger ThilmonyFatemeh KhatamiRogério Leone BuchaimFatemeh MovahediRogerio R. Sotelo-MundoFatih DemirRohit JainFatiha NassirRohit JoshiFatima AlshboolRohit KumarFatima CerqueiraRohollah EbrahimiFatima CvrčkováRohollah NasiriFátima García-VillénRoksana MarkiewiczFatima JesusRoland GeisbergerFatima MacedoRoland MalliFátima Milhano SantosRoland P. BouretteFatima MucceeRoland R. NetzFatimah KhalafRoland StauberFatma AydinogluRoldán-Roldán GabrielFatma O. KhalilRolf BrennerFatme AlanoutiRolf HeumannFausta LuiRolf SchlegelFaustine DubarRolf SprengelFausto RigoRolf TeschkeFavián TreulénRolf TurkFawzi BoumezbeurRomain DuvalFawzi Khoder-AghaRomain GautierFawzia Bardag-GorceRomain GuinamardFayna María García-MartínRomain LevayerFayyaz Ur Rehman MuhammadRomain RoncagalliFazal UllahRomain Veyron-ChurletFazlurrahman KhanRomain Vuillefroy De SillyFederica BuccinoRoman B. LesykFederica CampoloRoman BoroznjakFederica CherchiRoman FedunovFederica ChiapporiRoman HobzaFederica CirilloRoman HrstkaFederica CollinoRoman K. PuzanskyFederica De CastroRoman KostyuchenkoFederica Della RovereRoman KotlowskiFederica Di SpiritoRoman KozlovFederica FlamminiiRoman LesykFederica FogacciRoman LysiukFederica GiordanoRoman ShendrikFederica IavaroneRoman SmolarczykFederica InvernizziRoman SzewczykFederica MagagnoliRoman TsukanovFederica ManninoRomana Roje-BusattoFederica MarascaRomana VulturarFederica MartoranaRomanas ChaleckisFederica MontagneseRomeo Petru DobrinFederica PapaccioRomeo RomagnoliFederica ReyRómulo S. SobralFederica SabuziRon KedemFederica SodanoRon SaulnierFederica TorricelliRon ShapiroFederica VeroneseRon UngerFederico BrandaliseRona GrahamFederico Castro-MunozledoRona KarahodaFederico Gastón BaudouRonald A. LubetFederico GiannuzziRonald B. BrownFederico GulluniRonald J. ParchemFederico HerreraRonald M. IorioFederico LazzaroRonald NelsonFederico ManaiRonald WandersFederico Manuel GiorgiRonan BureauFederico PercheRonan MurphyFederico Pio FabrizioRonan TanguyFederico QuainiRong WangFederico SebastianiRong ZhouFederico UlianaRongcai JiangFedor KryuchkovRongjin SunFedor SimkoRongkung TsaiFedor V. SubachRong-Kung TsaiFedora GrandeRongyu LiFehmi DamkaciRoni NugrahaFei HeRonit Sagi-EisenbergFei PengRonit SionovFei TengRonn GoeiFei XingRonny MartínezFeilong LiRony SegerFeilong WangRoohallah Saberi-RisehFeipeng YangRoozbeh Abedini NassabFeiyue TengRosa AlduinaFelice LorussoRosa AmorosoFelice PetragliaRosa BonaventuraFelicia Ramona BirauRosa CalvelloFeliciana Real-FernándezRosa Cristina Simoes FernandesFelicitas BucherRosa Della MonicaFelicite Kamdem NoubissiRosa Di LiddoFelipe Andrés Cordero Da LuzRosa DivellaFelipe EltitRosa FontanaFelipe Girotto CamposRosa GaglioneFelipe J ChavesRosa LeónFelipe Klein RicachenevskyRosa Lo FranoFelipe López-HernándezRosa MancinelliFelipe Martinez-PastorRosa María Camacho-RuizFelipe ParedesRosa María Giráldez-PérezFelipe PezoRosa María Oliart-RosFelipe Siqueira De Souza Da RosaRosa MolfettaFelipe Valenzuela-RiffoRosa PurgatorioFelipe VielaRosa RoyFelisa Reyes-OrtegaRosa SuadesFelix A. RuizRosa VonaFelix AmissahRosalba MinelliFelix BeckerRosalba ParentiFelix ClanchyRosalba PerroneFelix EllettRosalba SiracusaFelix FluriRosalia CrupiFelix GoeserRosalía Fernández-AlonsoFelix Javier Jiménez-JiménezRosália Mendez-OteroFélix Javier Jiménez-JiménezRosália SáFelix Martin WernerRosalin MishraFelix NchuRosalinda SorrentinoFelix SchmittRosamaria LappanoFelix StahlRosana AlvesFelix SternbergRosana RibićFelix YemanyiRosanna Del GaudioFemi J. OlorunnijiRosanna Di PaolaFeng ChengRosanna MallamaciFeng GuoRosanna MarinoFeng HeRosaria ArconeFeng JunRosaria GrecoFeng PanRosaria IngrassiaFeng QueRosaria MeccarielloFeng TanRosaria SciarrilloFeng TangRosario AmatoFeng WangRosario CaltabianoFeng XuRosario DonatoFeng YuRosario Francesco DonatoFengchao LangRosario GulinoFeng-Cheng ChouRosario LinaceroFenghai GuoRosario MareFengjie SunRosario NicolettiFenglei YangRosario PivonelloFenglin ZhangRoselyn CerutisFengping DongRosemarie C. RosellFengqun YuRosemary WalzemFengyun MaRosemeire M. Kanashiro-TakeuchiFengzhen LiuRosenkran Sina CathérineFengzhi LiRoshan KumarFerdinand AlthammerRoshan Thotagamuge KumaraFerdinando BrancaRosilda Mara MussuryFerdinando ClarelliRoss C LarueFerdinando PalmieriRoss CarruthersFerdinando PaternostroRoss ChelohaFerdinando RomanoRoss F. ColleryFerdows AfghahRoss T. BarnardFerenc A. AntoniRossana C. ZepedaFerenc BoldizsárRossana CecchiFerenc GallyasRossana MorabitoFerenc MarkusRossana SolettiFerenc OroszRossella CacciolaFerenc PappRossella DoriaFerenc SiposRossella MieleFerenc TóthRossella PuglisiFerhunde AysinRossella SgarzaniFerman ChavezRossella SvigeljFernanda Guerra Lima Medeiros BorsagliRossella TozziFernanda Raya TonettiRossella TuplerFernanda SeixasRossitza LazovaFernanda VilarinhoRouf BandayFernández-Ocaña AnaRoumita MoulickFernando CalzadaRoushu ZhangFernando Capela E SilvaRousset RaphaëlFernando CardonaRoxana Cristina PopescuFernando CardosoRoxana LucaciuFernando CiveiraRoxana Racoviceanu (Babuta)Fernando De La CuestaRoxana VidicanFernando DiazRoxana WasnickFernando FerreiraRoxana-Maria AmărandiFernando GómezRoxane PouliotFernando Gomez-MerinoRoy A. QuinlanFernando LarcherRoy Byung Kyu ChungFernando Leiva-CepasRoy Chun-Laam NgFernando LidonRoy KennedyFernando López-GatiusRoy KessousFernando Lopitz OtsoaRoy ShenharFernando M. Araujo-MoreiraRoyce CliffordFernando MoragaRoyce MohanFernando PorcelliRozangela Curi PedrosaFernando RojoRozina KhattakFernando Sánchez-DávilaRuairidh J. H. SawersFerran Acuña-ParésRuben C PetreacaFerreira FernandoRuben DagdaFerreira Nelson FerreiraRuben Meana PanedaFerri Elida NoraRuben PérezFeten Zar KalaiRuben Soto-AcostaFevzi BardakciRubens NodariFidel Gonzalez TorralvaRubina JibranFidele TugizimanaRuby LawFilbert TotsinganRuchi BhartiFilimon AncaRuchi RoyFilip BergquistRuchi ShahFilip BoyenRuchika BajajFilip DabrowskiRuchika BhawalFilip De SomerRuczyński JarosławFilip Franciszek KarugaRudi H. EttrichFilip ŠeniglRudi PodgornikFilip VitoševićRudian ZhangFilip Y. De VosRüdiger KlapdorFilipe BuarqueRüdiger LawaczeckFilipe Carvalho De VictoriaRudolf KorinthenbergFilipe PintoRudolf LikarFilippo AntoniacciRudy J. ValentineFilippo BaldacciRudy OrtizFilippo BasagniRuei-Nian LiFilippo BiamonteRui ChenFilippo DragoRui HuangFilippo FratiniRui LiuFilippo M. SantorelliRui M. Borges Dos SantosFilippo Maria SantorelliRui M. S. CruzFilippo MaselliRui ShiFilippo MinutoloRui SilvaFilippo PieriniRui VitorinoFilippo RossignoliRui YanFilippo SamperiRuibo WuFilippo TorrisiRuichi ZhangFilippos BantisRuifang WeiFilippos TriposkiadisRuifeng CaoFiliz OnatRuifeng HuFilomena Anna DigilioRuiguang GeFilomena FonsecaRuiguo YangFilomena RistoratoreRuihua DongFina Martínez-SolerRuijie D. ZhangFinn Edler Von EybenRuilong ShengFiona AngrisanoRuipu MuFiona E. HarrisonRuiqing ShenFiona MccartneyRuitu LyuFiona McdonnellRuiwu LiuFiona WhelanRuizhong WangFiora ArtusioRu-Jeng TengFiorella TonelloRujun GongFiorentina RoviezzoRukang ZhangFiras KobeissyRukmani PandeyFirdous A. KhanRukmini MukherjeeFiroz AkhterRulin ZhuangFisayo OlotuRumana RanaFisun HamaratoǧluRumen IvanovFlavia AntonucciRumiana TzonevaFlávia Aparecida GraçaRumyana SimeonovaFlávia CastroRunchen FangFlavia FerrantelliRunchen ZhaoFlavia FontanesiRune MatthiesenFlávia Leão BarbosaRung-Yi LaiFlavia NastriRunhua HanFlavia TrionfettiRuojun WangFlavia ZacconiRuoli ChenFlavio RizzolioRuoxing WangFleming GudaguntiRupal I. MehtaFleming Martinez RodriguezRupali GuptaFlemming AndersenRupendra ShresthaFleur GartonRupert L. MayerFlor De Fátima Rosas CárdenasRupesh DeshmukhFlor María Pérez-CampoRupesh Kumar ChikaraFlora N. BalievaRupesh Kumar SinghFlóra SzeriRupesh SrivastavaFlorbela PereiraRupesh TayadeFlore MasRupinder KaurFlorence LegendreRusan CatarFlorence SessomsRushendhiran KesavanFlorence VorspanRuslan E. AsfinFlorenci V. GonzálezRuslan MariychukFlorencia RosettiRussell D'SouzaFlorent MorfoisseRussell GordonFlorentina Monica RadulyRusso EleonoraFlorentina PluteanuRüstem KeçiliFlorentina-Daniela MunteanuRuta MucenieceFlorian BeaumatinRute PereiraFlorian EmmerichRuth A. SchwalbeFlorian GembardtRuth Birner-GrünbergerFlorian GesslerRuth Dudek-WicherFlorian K EbnerRuth G. PérezFlorian LangerRuth GriffinFlorian SchulzRuth HalabanFloriana Della RagioneRuth NussinovFloriana VolpicelliRuth PrasslFlorin CiolacuRuth Prieto-MonteroFlorin OanceaRuti ParvariFlorin SalaRuwei LinFlorin TriponRuy Ribeiro CamposFlorina M. BojinRwik SenFlorina TeodorescuRyan A. MaddoxFloris SchoetersRyan BisbeyFnu SurakshaRyan J AndresFoad GhasemiRyan LimbockerFong-Yu ChengRyan LoganFor Yue TsoRyan MaillouxForough JahandidehRyan McgrathFortunata CarboneRyan Mead-HunterFortunato FernandaRyan RanalloFoteinos-Ioannis DimitrakopoulosRyan SartorFotini BoufidouRyan WuebblesFotis BaltoumasRyo MatsushimaFouquet GuillemetteRyo NakamaruFranca AnglaniRyo SagaFrancesc BorrásRyo TanakaFrancesc García GonzaloRyohei TatsunoFrancesc MirallesRyoichi AsakaFrancesc MoresoRyoji MitsuiFrancesc Xavier AvilésRyoko SaitoFrancesca AgriestiRyoma TakeshimaFrancesca AmatiRyoma YonedaFrancesca Anna CupaioliRyong Nam KimFrancesca BonviciniRyosuke MegaFrancesca BosciaRyota HosomiFrancesca BufalieriRyota MasuzakiFrancesca CantiniRyota ShizuFrancesca CattoniRyszard GołdynFrancesca CianiRyszard GrendaFrancesca CitronRyszard PlutaFrancesca Daniela FranzosoRyszard SmolarczykFrancesca De FalcoRyu ImamuraFrancesca De FeliceRyu WatanabeFrancesca De VitoRyuhjin AhnFrancesca Di SoleRyuji HayashiFrancesca DiomedeRyuji TakedaFrancesca GadoRyushiro D. KasaharaFrancesca GattoS Sylvester DarvinFrancesca GuarinoS. Balakrishna PaiFrancesca IommelliS. D’IppolitoFrancesca LazzaraS. F. El GioushyFrancesca Levi-SchafferS. H. ShinFrancesca MackenzieS. M. Shatil ShahriarFrancesca MancusoS. MahadevakumarFrancesca MapelliS. P. IyerFrancesca MaradonnaS. Patricia StockFrancesca MiselliS. Udhaya KumarFrancesca MontaroloS. V. Prabhakar VattikutiFrancesca MottarliniSaad El-Din HassanFrancesca PasiSaad FaroukFrancesca RappaSaad ShaabanFrancesca ReggianiSaak V OvsepianFrancesca ResentiniSabata MartinoFrancesca RicciSabbatini MaurizioFrancesca RiganoSabina GaliniakFrancesca RiuzziSabina PodlewskaFrancesca Romana MariottiSabina PurkrtováFrancesca S. ForiniSabina SblanoFrancesca SanguedolceSabina SevcikovaFrancesca SantilliSabina SkrgatFrancesca SciandraSabine BothaFrancesca SciontiSabine BrandtFrancesca SilvagnoSabine Chapuy-RegaudFrancesca StanzioneSabine GröschFrancesca SusaSabine KässmeyerFrancesca UbertiSabine Kuchler-BoppFrancesca VasileSabine PellettFrancesca ZaraSabine WindhorstFrancesca ZitoSabino RussiFrancesco AmentaSabinus Oscar Onyebuchi EzeFrancesco BellantiSabiruddin MirzaFrancesco BellinatoSabit HusseinFrancesco BennardoSabita SaldanhaFrancesco BertoliniSabrin IbrahimFrancesco BiancoSabrina BattistaFrancesco BoccalatteSabrina EhnertFrancesco BoccellatoSabrina Gensberger-ReiglFrancesco BrunoSabrina LisiFrancesco BuonocoreSabrina ManniFrancesco CacciolaSabrina OlivaFrancesco CadarioSabrina TaitFrancesco CanonicoSabrina ValenteFrancesco CeiSabrina VinzónFrancesco ChemelloSabyasachi DashFrancesco CiardelliSabyasachi MoulikFrancesco CiccareseSachchida Nand RaiFrancesco ClapsSachie NakataniFrancesco CorattiSachiko Toma-FukaiFrancesco D'AlòSachin ChauguleFrancesco De NuccioSachin Kumar SinghFrancesco Di GennaroSachin SarodeFrancesco Di MarioSachin VermaFrancesco FeoSachio FushidaFrancesco FerragutiSachio TakenoFrancesco FerraraSachio TsuchidaFrancesco FiorentinoSachit AnandFrancesco FornaiSadaf GilaniFrancesco FortarezzaSadaf JahanFrancesco FusoSadanori AkitaFrancesco GirolamoSadaruddin ChacharFrancesco LiguoriSaddam HussainFrancesco LodolaSaddam SaqibFrancesco LopsSadegh BalotfFrancesco M. PacificoSadia AfrinFrancesco MaioneSadiya ParveenFrancesco MannelliSaed KhaliliehFrancesco MaramponSaeed KaboliFrancesco Maria CalabreseSaeed KabrahFrancesco MarottaSaeed TarighiFrancesco MercatiSaei Ghare Naz MarziehFrancesco MerlinoSaeid GhavamiFrancesco MessinaSaeid HosseinzadehFrancesco MilanoSaeid KargozarFrancesco MusianiSaeko Tada-OikawaFrancesco NicoliSafaa KaderFrancesco OrienteSagar MaitraFrancesco PallottiSagar ManeFrancesco Paolo BusardòSagar RayamajhiFrancesco PellicciaSagar RoyFrancesco PepeSagarika BiswasFrancesco PezzellaSagarika MishraFrancesco PiccirilloSagrario Martin-AragonFrancesco PotiSahadev A. ShankarappaFrancesco PuosiSahar I. MostafaFrancesco RossellaSahdeo PrasadFrancesco SacconSaheem AhmadFrancesco SaltonSahi JasminderFrancesco SalvatoreSahib ZadaFrancesco SanvitoSahil InamdarFrancesco SartiniSahiti ChukkapalliFrancesco ScavelloSahnawaz AhmedFrancesco SessaSahra UygunFrancesco SestiliSai Pranathi Meda VenkataFrancesco SmedileSaid AudiFrancesco SpinozziSaid DermimeFrancesco StellatoSaida OrtolanoFrancesco SunseriSaied SolimanFrancesco TarantiniSaifei LeiFrancesco TavantiSaiful Amri Bin MazlanFrancesco TondoloSaiful IslamFranchi MarcoSaiful Izwan Abd RazakFrancis J. OsongaSaiful MalookFrancis ManSaifur RahmanFrancis William (Bill) LuscinskasSaigopalakrishna S. YerneniFrancisc BodaSaila VarisFrancisca Arán AisSaima ImranFrancisca Ferrer-MarínSaima RubabFrancisca M. AcostaSaima ZameerFrancisca RodriguesSainitin DonakondaFrancisco Alexandrino JúniorSairam BeheraFrancisco AmadoSaiyang ZhangFrancisco BandeiraSajal SenFrancisco C Franco, Jr.Sajid AliFrancisco CastilloSajid ShokatFrancisco CayabyabSajjad AhmadFrancisco Cortés-BenítezSajjad ShiraziFrancisco Cruz-SosaSajjad U. AhmadFrancisco De AzambujaSajjan GroverFrancisco DomínguezSaket PatelFrancisco DonosoSakthi RajendranFrancisco E. NicolasSalam IbrahimFrancisco EnguitaSalar ShaafFrancisco EpeldeSalar VaseghiFrancisco Eroni Paz Dos SantosSalatul Islam MozumderFrancisco EspinosaSalej SoodFrancisco ExpósitoSalesh Kumar JindalFrancisco Fueyo GonzálezSalil DesaiFrancisco Gabriel Vázquez-CuevasSalim AlbukhatyFrancisco González-ScaranoSalim HeddamFrancisco Guillen-GrimaSalim MorammaziFrancisco J MedranoSally CattFrancisco J. BlancoSally FreemanFrancisco J. Flor-MontalvoSally MolloyFrancisco J. NicolásSally TemrazFrancisco J. Vega-VeraSally WasefFrancisco Javier Alvarez-MartinezSalma AbedelmalekFrancisco Javier GrijotaSalma BhyanFrancisco Javier Marcos‐TorresSalman AhmedFrancisco Javier MedinaSalman AliFrancisco Javier Navas GonzálezSalman WaqasFrancisco Javier SilvestreSalva Reverentia YuristaFrancisco José González-MineroSalvador BorrosFrancisco Juan Martinez NavarroSalvador Dueñas CarazoFrancisco Lázaro-DiéguezSalvador MáñezFrancisco LeonSalvador MireteFrancisco M. Martin-SaavedraSalvador PérezFrancisco MarcoSalvador SorianoFrancisco Mendoza-CarreraSalvatore AuricchioFrancisco Nadal-NicolasSalvatore BaldinoFrancisco Navarro-TriviñoSalvatore FaillaFrancisco RiveroSalvatore FiorinielloFrancisco Rodrigues PintoSalvatore GalloFrancisco Sánchez-BuenoSalvatore Giovanni De SimoneFrancisco Sandro Menezes-RodriguesSalvatore NesciFrancisco SautuaSalvatore NovelloFrancisco SolanoSalvatore SacconeFranck ChiappiniSalvatore SantamariaFranck CosteSalvatore ScaccoFranck Farzad RahaghiSalvatore SorrentiFranck MartinSalvatore ZaffinaFranck MeyerSalvio Suárez-GarcíaFranck MorelSam KimFranco BisceglieSam LoflandFranco CardoneSam MerlinFranco CervellatiSam VirtueFranco D’OrazioSamanta MazzettiFranco LucchiniSamanta ZelascoFranco MusioSamantha BrunoFrançois ChevalierSamantha KendrickFrançois De MetsSamantha M. LoiFrancois FaitotSamantha ShanFrancois FenailleSamar AzizighannadFrançois GuerineauSamar G. ThabetFrancois IchasSamar ImbabyFrançois JamarSamar M. HammadFrançois KarchSamar TharwatFrançois KrierSambantham ShanmugamFrancois Le NormandSambit MishraFrancois MeurensSambuddha BasuFrancois MoisanSameena IqbalFrançois MorvanSameera AbeyrathnaFrançois PerreauSameh A. KormaFrançois SaucySameh El SayedFrançoise LenfantSameh GehaFrancoise MartzSameh SelimFrançoise PorteuSami Abou FayssalFrançois-Xavier LegrandSamia ElseginyFrane PaicSamina AlamFrank A. DucaSamir AhmadFrank AboubakarSamir Al-AdawiFrank KoSamir BarghoutFrank KrauseSamir ChtitaFrank LovicuSamir KumarFrank LykoSamira FerreiraFrank MüllerSamira MalekmohammadiFrank NicholasSamira Samiee-ZafarghandyFrank OechslinSamira TarashiFrank R GreerSamiya Al-RobaiyFrank Richard SchubertSamkelo MalgasFrank RyanSamntha MacchiFrank S. BarnesSampath Dakshina Murthy AchantaFrank SchnütgenSampath VemulaFrank SiebenhaarSamrita DograFrank ThevenodSamsad RazzaqueFrank Vinholt SchiødtSamson AisidaFrank ZauckeSamson Mathews SamuelFranka Klatte-SchulzSamuel De VisserFranklin W. N. ChowSamuel E. AdunyahFrantisek HnilickaSamuel E. SchrinerFranz Felix KonenSamuel Fernández-ToméFranz Joachim HilkeSamuel GanFranz L. DickertSamuel J. FountainFranz ZehentmayrSamuel Ken-En GanFranziska ErsoySamuel Lara-ReynaFranz-Josef SchingaleSamuel NutileFrauke StankeSamuel PhillipsFred EyaseSamuel PushparajFred StevensSamuel SilvestreFreddy Fernandes GuimarãesSamuel X. ShiFrédéric BantigniesSamuele GhezzoFrederic ChibonSan HadžiFrédéric CoutantSan Ming WangFrédéric DumurSana MajidFrédéric EbsteinSananda MondalFrédéric GoormaghtighSanath KumarFrédéric Huppé-GourguesSanchari BhattacharyyaFrederic JaisserSandeep KondakalaFrédéric LaumonnierSandeep KumarFrederic LezotSandeep Kumar BarodiaFrederic MarsolaisSandeep Kumar MishraFrederic MelinSandeep Kumar Reddy AdenaFrédéric Nicolas DaussinSandeep LohanFrédéric PicouSandeep MalampatiFrédéric RaymondSandeep SinghFrederic TessierSander RatsoFrederick H SilverSandesh PanthiFrederick J. FullerSandi Baressi ŠegotaFrederick LiaSandile Phinda SongcaFrederick WilliamsSandino Ana MaríaFrederico B. De SousaSandip Ashok SonarFrederico PittellaSandip GhugeFrederico PontesSandip K. BasuFrédérique A. O. HilliouSandip M. VibhuteFrederique LisacekSandipan DattaFredrick J. RosarioSándor GóbiFredy A. SilvaSándor GondaFreiss GillesSándor KékiFrida BelinkySándor Kunsági-MátéFrida LoriaSandra AckermannFriederike Berberich-SiebeltSandra BarbalhoFriederike KlempinSandra BurattaFriedrich JungSandra BurgstallerFrikkie J. W. CalitzSandra CavacoFrithjof C. KüpperSandra CitiFritz BoegeSandra ClarkeFruke SeemannSandra DonniniFu LuoSandra Garcia-GallegoFu-Chen HuangSandra GavaFuencisla Pilar-CuéllarSandra GhelardoniFugo TakasuSandra GómezFuhua HaoSandra LeiszFujie ZhaoSandra M. Martin GuerreroFu-Jung LinSandra Maria Carmello-GuerreiroFulgencio AlatorreSandra McfaddenFulvio CelsiSandra MosqueraFulvio MassaroSandra Oramas-RoyoFumiaki UchiumiSandra PinaFuming TsaiSandra PintoFuminori TokunagaSandra PucciarelliFumio KawamuraSandra RebeloFumio MatsudaSandra RecueroFumitaka KogaSandra ReznikFumiyuki ItoSandra Ribeiro MacedoFun-In WangSandra RistoriFuquan LiuSandra RychelFurong TianSandra Sanchez SalcedoFusheng SiSandra SimoesFu-Yin HsuSandra SoaresFuyou FuSandrine BarbauxFuyuki SatoSandrine BetuingG AnbuchezhiyanSandrine EveillardG BrajuškovićSandrine HormanG. A. Castillo-RodríguezSandrine Huclier-MarkaiG. GnanamoorthySandrine LauranceG. Ian GallicanoSandro MicheliniG. Suresh KumarSandro OrrùG. V. DedkovSang Chul ParkG. W. Gant LuxtonSang Geon KimGabin GbabodeSang Kyu KimGabina Calderón-RoseteSang Yeop LeeGabor GalibaSang-Bae LeeGabor HòlloSangbum ParkGábor János SzebeniSang-Han LeeGábor K. TóthSangho RohGábor KocsySang-Hwan HyunGábor KökénySang-Ik OhGábor KonczSangjune KimGábor L. KovácsSangmi LeeGabor MikalaSang-Nam KimGábor NagySangpil KimGábor Nagy-GróczSangram SamalGábor PethőSangsu BangGábor RigóSang-Won LeeGábor SomlyaiSang-Wook KangGábor SzénásiSanika SuvarnapathakiGábor TernákSanja BaricGábor TuruSanja DabelicGabriel AnesettiSanja DabelićGabriel CharestSanja Fabek UherGabriel Costa A. Da HoraSanja StankovićGabriel EstrelaSanja Zoričić CvekGabriel GarcíaSanjairaj VijayavenkataramanGabriel Goetten De LimaSanjay AroraGabriel GonzalezSanjay DeyGabriel HancuSanjay GuptaGabriel IturriagaSanjay KumarGabriel Matos-RodriguesSanjay MishraGabriel PetreSanjay NagarajanGabriel PlavanSanjay PatelGabriel SosneSanjaya Kumar SahuGabriel WajnbergSanjida Sultana KeyaGabriel ŽoldákSanju GuptaGabriela Angélica Martínez-NavaSankar JagadeeshanGabriela AniteiSanket MishraGabriela Batitucci MirandaSanket P. AnaokarGabriela BurgosSanna MeriläinenGabriela Castañeda‐CorralSanne Van LithGabriela CiapettiSanower HossainGabriela DudekSantanu MajiGabriela GarciaSantanu MukherjeeGabriela González MariscalSanthosh ArulGabriela HrckovaSantiago BonanadGabriela IonitaSantiago Garcia-VallvéGabriela KowalskaSantiago J. BallazGabriela Medina-PérezSantiago Martínez-CalvilloGabriela Nérida Condezo CastroSantiago P. AubourgGabriela PetrofSantiago RodríguezGabriela QuirogaSantiago RubenGabriela RosaSanto Raffaele MercuryGabriela Tomas JerônimoSantosh A. MisalGabriela VlaseSantosh BashyalGabriela Vollet MarsonSantosh GeorgeGabriela-Dumitrita StanciuSantosh K. YadavGabriele BaniulyteSantosh KumarGabriele CarulloSantosh Kumar KunchaGabriele CeccarelliSantosh PeddiGabriele CervinoSantosh PothulaGabriele CheliniSantosh UpadhyayGabriele CiascaSaowaluck TibprommaGabriele GiachinSaptarshi RoyGabriele MadonnaSaqib HassanGabriele MeloniSara B. PereiraGabriele MeroniSara BozziniGabriele MorucciSara CaldrerGabriele NibbioSara CaratelliGabriele RocchettiSara Casado ZapicoGabriele RuffoloSara ChiappalupiGabriele SaretzkiSara ContiGabriella ChieffiSara CorreiaGabriella CsíkSara CrucianiGabriella D’OraziSara Duarte-SilvaGabriella GulyasSara FranceschelliGabriella HorváthSara FranziGabriella MilanSara GaspariniGabriella PanuccioSara Guerrero-AspizuaGabriella SarmaySara Gutierrez-EnriquezGabriella SerioSara I. Al-GhadbanGabriella ZitoSara Luz Morales-LázaroGadi PelledSara MarçalGadi SchusterSara MassironiGadi TurgemanSara MoradiGaetan GlauserSara OppenheimGaëtan GuignardSara PagottoGaetana CostanzaSara PalumboGaetano CataneseSara PedronGaetano GalloSara Pérez-LuzGaetano La MannaSara PerteghellaGaetano Magnano Di San LioSara PintoGaetano MarvertiSara R. Martins-NevesGaetano SantulliSara RamírezGagan D. GuptaSara RavaioliGagan FloraSara Remuzgo-MartínezGagandeep SagguSara Rodríguez-AcebesGagandeep SinghSara Rosalía Morcuende FernándezGaganpreet KaurSara S. ReisGaia BarisioneSara SaviniGaia CodoloSara SestiliGaia FaustiniSara TombelliGaia PellegriniSara YasipourtehraniGail A. CornwallSara ZocherGaiti HasanSaradha VenkatachalapathyGajanan GhodakeSarah A. CummingGalal MetwallySarah BeckGalaxia M. RodriguezSarah BouzroudGali PragSarah DuinGalia Ramírez-TolozaSarah Elizabeth TaylorGalina A. KovalenkoSarah J. AllgoodGalina DegtjarevaSarah L. ReaGalina NikolovaSarah L. WaltonGalina RalduginaSarah LamereGalina ShepelkovaSarah Louise Haywood-SmallGalina T. YahubyanSarah MazzottaGalina V. KopylovaSarah Santos GonçalvesGalina V. KurlyandskayaSarah StraussGalip AkaySarah VergultGalkin Konstantin I.Sarah White-SpringerGalli Gabriela MiottoSarah-Lena PuhlGalushko AlexanderSaral DesaiGalya Ivanova GanchevaSarantis GagosGalya StanevaSaratchandra KhumukchamGamal BadrSarath VijayakumarGanapathi KandasamySaravanan DevendranGanesan GovindhanSaravanan JanakiramGanesh Dattatraya SarataleSaravanan KmGanesh JedheSaravanan KolandaiveluGanesh KoyyadaSaravanan MuthupandianGanesh MohanSarfaraz IqbalGanesh MurhadeSarfraz ShafiqGanesh NikaljeSargam KapoorGanesh Ram VisweswaranSargis KarapetyanGanesh SwamySargis SedrakyanGanesh UmapathySari MattilaGanesh YadaigiriSarika ChaudhariGang ChenSarina KajaniGang LiSarit PalGang WangŠárka JelínkováGang WeiSarka NemeckovaGang WuSarkar M. Abe KawsarGang YeSarmistha TalukdarGangarapu KiranSaroj AmarGannon C. K. MakSaroj Kumar SahGao XiaolongSaroja VorugantiGaofeng WangSarthak SinghalGaosen ZhangSarunporn TandhavanantGarcía-Esquinas EstherSarvesh Pratap KashyapGareth MorganSarwar BegGarikoitz AzkonaSaša FrankGarima ArvikarSaša KazazićGarrett EllwardSaša KostićGarry KerchSasa RajsicGarry LavertySascha BenekeGary D. NovackSascha DrewloGary PehSashi DebnathGary SteinmanSashi KantGaryfallia KapravelouSasidharan SreenivasanGaskon IbarretxeSaskia PreissnerGąsowska-Bajger BeataSatheesh Kumar SengodanGaston PizzioSatheesh SengodanGatikrushna SinghSathish K. R. PadiGauhar RehmanSathiyaseelan AnbazhaganGaurav DhawanSatilmis BilginGaurav JoshiSatish GandhesiriGaurav YogeshSatish KumarGauri PrasadSatish Kumar RajasekharanGausal KhanSatish Kumar SainGautam AnandSatish RainaGautam N. ShenoySatish ToteyGautam PareekSatohiro MasudaGautam SethiSatoru KobayashiGaweł SołowskiSatoru KyoGaya Punnia-MoorthySatoru NoguchiGayathri NatarajanSatoru OnizukaGayatri DevrajSatoru TamuraGayatri SharmaSatoshi FukuchiGea-Ny TsengSatoshi KishigamiGederst IevinshSatoshi KomatsuGederts IevinshSatoshi MigitaGee Jun TyeSatoshi NagaokaGeert Jan SterkSatoshi ObikaGeert PottersSatoshi SaitoGeert RaesSatoshi TakahashiGeert Van Den BogaartSatoshi TakasugiGeertje Van Der HorstSatoshi TanakaGege YanSatoshi TateishiGeir BjørklundSatoshi WaguriGelei XiaoSatoshi YamaguchiGema Frühbeck MartínezSatoshi YuguchiGema Martinez-NavarreteSaturnino Marco LupiGema Martínez-NavarreteSatwant KumarGemaria Addolorata MariggiòSatya KotaGemma ArderiuSatya P. KunapuliGemma Caterina Maria RossiSatyabrata NandaGemma GutiérrezSaul Herranz-MartinGemma Jiménez-GuerraSaura R SilvaGemma MarfanySaurabh ChaturvediGemma Oms-OliuSaurabh ChaudharyGemma PalazzoloSaurabh DubeyGemma VillorbinaSaurabh GautamGemma ZernaSaurabh MishraGen KanekoSaurabh PandeyGen LiSaurabh SinghGen ZouSaurav Kumar JhaGenevieve C. SparagnaSaveg YadavGenevieve Mary SullivanSaveria FemminòGennady ChuevSaverio CandidoGennady SemisotnovSaverio MuscoliGennaro EspositoSavin TreantaGennaro RuggieroSavina ApolloniGenny Del ZottoSavio Domenico PandolfoGenny OrsoSavvas LampridisGenqiao LiSawako YamashiroGenwei ZhangSawan Kumar JhaGeo PaulSawsan A. ZaitoneGeoff HolmSawsan ZaitoneGeoffrey C. LiSayak DasguptaGeoffrey HudsonSayaka MiuraGeoffrey HutinetSayan ChakrabortyGeoffrey MitchellSayan GangulyGeoffrey ThieleSayanta BeraGeok Chin TanSayantanee DuttaGeorg AuburgerSayantani ChowdhuryGeorg BreierSayantani Sarkar BhattacharyaGeorg DewaldSayed Haidar Abbas RazaGeorg GelbeneggerSayed K. GodaGeorg HettichSayem MiahGeorg JohnenSazan RasulGeorg KranzScarfì FedericaGeorg KuenzeSchohraya SpahisGeorge A. TruskeyScot HandyGeorge A. TsihrintzisScott AllenGeorge Alexandru MafteiScott C. SchuylerGeorge BaylissScott CanfieldGeorge C. StewartScott E. FullerGeorge Calin DindeleganScott E. WenderferGeorge CarnellScott JohnstoneGeorge D. WilsonScott LevickGeorge E. FroudakisScott MccombGeorge FthenakisScott OlejniczakGeorge G. AbashevScott SouthernGeorge H. JonesSe Eun HaGeorge HsiaoSean Chun Chang ChenGeorge J. DugbarteySeán O'LearyGeorge Konstantinos PapadimasSean P. McbrideGeorge KontogeorgosSean R. AsselinGeorge KoobSean R. ScottGeorge KunosSebastià Galmés MonroigGeorge LambrinidisSebastiaan WertenGeorge LeondaritisSebastian BöttgerGeorge LiouliosSebastian FindekleeGeorge M. SavvaSebastian GnatGeorge Mihai NitulescuSebastian GranicaGeorge Mihail VlăsceanuSebastián HormigoGeorge N. BelibasakisSebastian HuthGeorge Nikov ChaldakovSebastian KłosekGeorge NotasSebastian KruppertGeorge P LaliotisSebastian KuśmierzGeorge P. DinosSebastian MertowskiGeorge P. ParaskevasSebastian NiestępskiGeorge PanosSebastian O. DeckerGeorge PsomasSebastian P. SchwamingerGeorge SourvinosSebastian PawlusGeorge TavartkiladzeSebastian PiłsykGeorge Th TsangarisSebastian SchloerGeorge TherapondosSebastian SchnaubeltGeorge TzotzosSebastian TancoGeorge ValasoulisSebastian VogtGeorge VassilopoulosSebastian WawrockiGeorge VerrosSebastian YuGeorge W. LiechtiSebastian ZahnreichGeorge ZanazziSebastian Ӧther-Gee PohlGeorges LeclercqSebastiano Alfio TorrisiGeorgeta Violeta TruscaSebastiano CampisiGeorgi KostovSebastiano CavallaroGeorgia GomatouSebastiano CiccoGeorgia KarpathiouSebastiano Di BucchianicoGeorgia KournoutouSebastiano Ettore SpotoGeorgia M. ParkinSebastien AlphonseGeorgia MandolesiSébastien BalmeGeorgia OuzounidouSébastien BonnetGeorgia-Eirini DeligiannidouSébastien BrittonGeorgiana NitulescuSebastien CalboGeorgios A. ChristouSebastien LacroixGeorgios AndroutsopoulosSébastien PenninckxGeorgios ArnaoutakisSebastien RinaldettiGeorgios BokiasSébastien Sébastien WieckowskiGeorgios D. PanosSeçil VuralGeorgios E. ArnaoutakisSeda KeleştemurGeorgios GermanidisSeda KoyuncuGeorgios PampalakisSeema GuptaGeorgios PapadopoulosSeemann MyriamGeorgios S MarkopoulosSeethi Reddy Reddisekhar ReddyGeorgios SkretasSegula MasaphyGeorgios StamatasSegun OgundareGeorgios TheodorouSehrish MananGeorgios TsaniklidisSeifollah GholampourGeorgios TsiavaliarisSeiichi FurumiGeorgy BakalkinSeiichi IshidaGeorgy GrancharovSeiichi KitagawaGeorgy K. FukinSeik-Soon KhorGeovanna Quiñonez-BastidasSejung KimGera SoniaSeka LazareGerald RimbachSekar VijayakumarGerald TomkinSekyung OhGerard ClarkeSelda OezkanGerard DumancasSelim KuçiGerard F HoyneSelma Osmanagic-MyersGerard MartensSelma Soares De OliveiraGerardo BykSelman UluisikGerardo CasucciSelvakumar SubbianGerardo FatoneSelvaraj Rajesh KumarGerardo Fernández BarberoSemih Can AkıncılarGerardo FerrerSenay UstunelGerardo GuillenSenka MeštrovićGerardo MusuracaSenol PiskinGerardo SalinasSenthil ChenrayanGerardo Z. LederkremerSenthil Kumar ThamilarasanGerasimos DarasSenthilkumar PalanisamyGerben J. SchaafSenwei TsaiGerd HeuschSeogsong JeongGerdi TuliSeong Beom ChoGereon PoschmannSeong Soo A. AnGergana DeevskaSeong-Hee Maria KoGergely FeherSeong-Hoon KimGergely GyimesiSeongjae JangGergely ZachárSeon-Yong JeongGergő TóthSepand RastegarGerhard FranzSepanta HosseinpourGerhard HaidlSepideh AliasghariGerhard HesslerSepideh Zununi-VahedGerhard P. PüschelSerafeim PapadopoulosGerhard PüschelSerafí CambrayGerhard RammesSerap Özer YamanGerhard StegerŞerban Mihai BǎlǎnescuGerhild EulerSerdar SatarGermán EbenspergerSerena AcetoGermana MeroniSerena AmmendolaGermana ZaccagniniSerena ArnaboldiGermano GuerraSerena BianchiGernot KriegshauserSerena BoninGernot PosseltSerena CarpentieriGernot ZollnerSerena CarraGero BenckiserSerena CecchettiGerrit BegemannSerena DantiGerrit JansenSerena DuchiGerry LushingtonSerena GasperiniGershon GolombSerena LattanteGerson Aparecido Foratori-JuniorSerena MazzucchelliGerwin HellerSerena MilanoGesmi MilcovichSerena MirraGeun-Shik LeeSerena VeschiGewei LianSerge BressonGéza SáfránySerge M. RozovGhada AbughanamSerge NatafGhadir S El‐HousseinySerge PerezGhana ChallaSergei A. SubbotinGhasem FarahmandSergei F. ChekmarevGhasem SargaziSergei G. GaidinGhasem SolgiSergei KulinichGhassem EmamverdianSergei ManzhosGhazala MustafaSergei NechaevGhazala NawazSergei NekhaiGheorge IliaSergei Y. GrishinGheorghe IliaSergej PirkmajerGheorghe NechiforSergejs GaidukovsGholamreza AbdiSergey A. AdoninGhulam Md AshrafSergey A. DenisovGiacinto BagettaSergey A. DyshlovoyGiacomina BrunettiSergey A. MileninGiacomo BusoSergey BaykovGiacomo CanesinSergey BrodskyGiacomo DonatiSergey ChetverikovGiacomo LazzarinoSergey DyshlovoyGiacomo TiniSergey FedosovGiacomo TondoSergey IordanskiyGiada BianchettiSergey IvanovGiamberto CasiniSergey KasparovGiampaolo MorcianoSergey KiselevGiampiero CaiSergey KolomeichukGiampiero MeiSergey KozlovGian Carlo BellenchiSergey KulemzinGian Luigi RussoSergey LevitskiiGian Marco PoddaSergey MayorovGianandrea PasquinelliSergey MikhailovGiancarlo CondelloSergey MoskvinGiancarlo ParentiSergey N. KonchenkoGiancarlo RipabelliSergey NetesovGiandomenico AmicoSergey SedykhGiandomenico CorradoSergey ShvetsovGianfranca CartaSergey SkublovGianfranco FrigerioSergey TimofeevGianfranco NataleSergey UspenskiiGianfranco PannellaSergey V. DorozhkinGianfranco PintusSergey V. OkovityiGianfranco SantovitoSergey V. StasenkoGianluca AguiariSergey YastrebovGianluca BaldanziSergi CésarGianluca CampoSergi MaicasGianluca CarusoSergii BabichevGianluca CatucciSergii KrysenkoGianluca CiardiSergio Aguilera-AlbesaGianluca GaidanoSergio CominciniGianluca LattanziSergio ContiGianluca LavancoSergio Crespo-GarciaGianluca NazzaroSergio Cuevas-CovarrubiasGianluca PaventiSergio Daishi SasakiGianluca RizzoSérgio F. SousaGianluca TedaldiSergio FacchiniGianluca TellSergio FucileGianluca TettamantiSergio García-GarcíaGianluca TosiniSergio Gastón CaspeGianluca ViscusiSergio GiannatasioGianluigi CalifanoSergio Gómez-GrañaGianluigi MazzoccoliSergio HidalgoGianmarco AbbadessaSergio M. Granados-PrincipalGianmarco LugliSergio MartíGianmaria SalvioSergio MolinariGianna DipalmaSérgio P CamõesGianna Serafina MontiSergio Piñeiro-HermidaGianni Gori SavellinSergio PontejoGianni TestinoSergio Posada PérezGiannis D. ParaskevopoulosSergio Rius-PérezGianpaolo PapaccioSergio RoyuelaGianpaolo RossiSergio Ruffo RobertoGianpaolo ZerbiniSerigo AcostaGianpiero GarauSerk In ParkGiau VoSerkan YílmazGideon GroganSeth CoreyGiedrius KalesnykasSeth E. FrietzeGijung KwakSeth FrietzeGil FraquezaSeth GrantGilbert BellangerSeth HallströmGilbert GallardoSetsuko ShimadaGilbert Nchongboh ChofongSeung Hee LeeGilberto Gonzalez-AvalosSeung Hwa YooGilles BreuzardSeung Min NamGilles DietrichSeung Myung MoonGilles FlouriotSeung Youn LeeGilles KauffensteinSeung Yun NamGilles LemaîtreSeung-Bok ChoiGilles LemercierSeunghwan YangGilles MonifSeung-Hyun RoGilles SalbertSeunghyung LeeGillian LoweSeungil RoGinka K. ExnerSeung-Joo LeeGino GrifoniSeungkeun ChoiGino SeravalleSeung-Min ParkGin-Shin ChenSeungwon JungGintautas BagdziunasSéverine BärGioele CapilloSevero Vázquez PrietoGiorgia ColomboSevinci PopGiorgia FedericoŞeyda BostancıGiorgia GioacchiniSeyed Alireza SalamiGiorgia MoschettiSeyed Arash AlawiGiorgia PallafacchinaSeyed Ehsan EndermiGiorgia SimonettiSeyed Hossein HoseinifarGiorgina SpecchiaSeyed Khosrow TayebatiGiorgio BergaminiSeyed Mohammad Ali Hosseini RadGiorgio Della SalaSeyed Mohammad Kazem AghamirGiorgio DieciSeyed Mohammad Nasir MousaviGiorgio FacchettiSeyede Raheleh YousefiGiorgio FilosaSeyit Ahmet ErolGiorgio GambinoSeyyed Alireza HashemiGiorgio GiorgiSeyyed Mehdi KhoshfetratGiorgio LenazSeyyed Mojtaba MousaviGiorgio MalpeliSezai ErcisliGiorgio SantoroShabbir AhmedGiorgio SonninoShabir WaniGiorgio TregliaShadab MdGiorgos P. TsironisShadi H. TabibianGiou-Teng YiangShafi M. KuchayGiovanna BaronShaghayegh NavabpourGiovanna CalabreseShagufta PerveenGiovanna CasiliShah Alam KhanGiovanna Cassone SalataShahab UddinGiovanna D’EliaShahar ShellyGiovanna D'AndreaShahbaz KhanGiovanna DesandoShaheen MowlaGiovanna FranciosaShahenda MahgoubGiovanna FrugisShahid AdeelGiovanna GalloShahid KhanGiovanna GrimaldiShahid MansuriGiovanna LiguoriShahid SarwarGiovanna MontanaShahid UmarGiovanna MurmuraShahidul IslamGiovanna OrsiniShahin ImranGiovanna PontarinShahriar KhanGiovanna RigilloShahriar ShahiGiovanna TrainaShahrokh GhobadlooGiovanni AbatangeloShahryar JafarinejadGiovanni BarillariShahzad MunirGiovanni BarosiShahzaib AhamadGiovanni CannarozzoShaid AliGiovanni CenciShaikhul IslamGiovanni ChiariniShailendra GoelGiovanni CimminoShailendra Kumar Dhar DwivediGiovanni CrisafulliShailendra Pratap SinghGiovanni CugliariShailendra SinghGiovanni DamianiShailesh KumarGiovanni Dell’Aversana OrabonaShajahan AnverGiovanni Di LibertoShakeel AhmadGiovanni Di ZenzoShakeel IjazGiovanni GalloShakhawan AlzanganaGiovanni GhigoShakina Yesmin SimuGiovanni L. BerettaShambhabi ChatterjeeGiovanni La PennaShameem LadakGiovanni LanteriShamik MajumdarGiovanni LongoShamil LatypovGiovanni Maria IannantuonoShaminie AthinarayananGiovanni MergoniShamir CassimGiovanni Nicolao BertaShamroop Kumar MallelaGiovanni PalleschiShams TabrezGiovanni PallioShamus CarrGiovanni PietrograndeShan HeGiovanni RibaudoShan ZhengGiovanni RollaShanbeh ZienolddinyGiovanni SalzanoShancy JacobGiovanni ScambiaShane A. BobartGiovanni SignoreShanfeng ZhuGiovanni StelitanoShangru LyuGiovanni TarantinoShangting YouGiovanni TazzioliShangwu DingGiovanni TisciaShan-Ju YehGiovanni TossettaShankar Jaikishan EvaniGiovanni TulipanoShanmuga S MahalingamGiovanni VintiShannon ConleyGiovanni VitaleShannon M. BaileyGiovanni VozziShantanu GuptaGiovanni William OliverioShantanu PradhanGiovanni ZifarelliShanthi G. ParkarGirdhari RijalShanti GangwarGiridhar SekarShanyi ShenGirish BeedesseeShao ChunlinGirish C. MelkaniShao Jung HsuGirish PattappaShao LiGirolamo CasellaShao Y. LuGirum GetachewShaoan WangGisela SantosShaobo RuanGisela SlaatsShao-Fang NieGisele PicoloShao-Hsuan KaoGisella VischiniShaohua ChenGislane Lelis Vilela De OliveiraShaohua JiangGitta TurowskiShao-Hung WangGiulia AbateShaojuan ChenGiulia BernardiniShao-Wen HungGiulia BertoliniShaowu XueGiulia BrunelloShaoxun WangGiulia CantiniShaoyong LuGiulia De RisoSharad GuptaGiulia DegiacomiSharat PradhanGiulia FestaSharath MadasuGiulia FreerShariq NajeebGiulia GaggiShariq QayyumGiulia GuarnieriSharkerGiulia LunghiSharmila FagooneeGiulia ModicaSharmila GhoshGiulia QuattrocoloSharmila MasliGiulia RioloSharmin HasanGiulia RussoSharmistha BarthakurGiulia SelvoliniSharon FordGiulia SitaSharon K. MichelhaughGiuliana ManninoSharon MiksysGiuliana MuzioSharyn PerryGiuliano CallainiSha-Sha ChengGiuliano RamadoriShasha YinGiuliano ZabucchiShashank KeshavmurthyGiulio ArcangeliShashank PandeyGiulio CeolottoShashi Shekhar SinghGiulio FerreroShashikant TiwariGiulio FracassoShashikumar SalgarGiulio FrontinoShatavisha DasguptaGiulio GabbianiShathiyah KulandaveluGiulio MalucelliShaun LeiversGiulio PilusoShaun S. SandersGiulio RossiShauna KaplanGiulio VernaShawn BurgessGiuseppa GraceffaShawn LyonsGiuseppe AlloattiShawn MclaughlinGiuseppe AlonciShay CovoGiuseppe AmorusoShazia RehmanGiuseppe BiaginiShazid Md. SharkerGiuseppe BroggiSheau-Chiann ChenGiuseppe BronteShebli AtrashGiuseppe BrunettiShehwaz AnwarGiuseppe CalamitaSheida MoghadamradGiuseppe CelentanoSheik Pran Babu Sardar PashaGiuseppe CirilloSheikh Mohammad Fazle AkbarGiuseppe D’AngeloSheila NielsenGiuseppe DamanteSheila SpadaGiuseppe Della PepaSheila V. GrahamGiuseppe Di GiovanniSheiliza CarmaliGiuseppe DionisioShek Man ChimGiuseppe Domenico ArrabitoSheng FuGiuseppe D'OnofrioSheng WangGiuseppe Federico AmodeoSheng ZhangGiuseppe FerrandinoSheng-Dan WuGiuseppe FiermonteSheng-Heng ChungGiuseppe GavazziSheng-Hua WuGiuseppe GiudiceSheng-Hung ChenGiuseppe GrazianoShengjie FengGiuseppe IettoShenglin MeiGiuseppe LanzaSheng-Ming WuGiuseppe LazzaraSheng-Nan WuGiuseppe Lazzarino‪Shengqian Xia‬Giuseppe LosurdoShengshuai ShanGiuseppe LucarelliSheng-Ta TsaiGiuseppe MaccagnanoShengyi (Iris) SunGiuseppe ManninoSheng-Yow HoGiuseppe MartelliSher Bahadur PoudelGiuseppe MasciaSherif KaramGiuseppe MaulucciSherif MoussaGiuseppe Maurizio CampoSherif T. S. HassanGiuseppe MazzarellaSherren HamzaGiuseppe MiloneSherri L. ChristianGiuseppe MurdacaSherry S. CollawnGiuseppe MusumeciSheyda ShakibaGiuseppe NovelliShi YaoGiuseppe PalmieriShiang-Jong TzengGiuseppe PassarinoShianjang YanGiuseppe PernaShian-Ren LinGiuseppe PeruginoShiao-Wei KuoGiuseppe PezzottiShiba DandpatGiuseppe PiccioneShibin ChengGiuseppe PignataroShiblur RahamanGiuseppe Pio AnastasiShibo ChengGiuseppe PizzolantiShibo WangGiuseppe PortellaShibo YuGiuseppe ProcinoShichao LvGiuseppe PugliaShicheng GuoGiuseppe RemuzziShigeharu UekiGiuseppe RivaShigehiro KarashimaGiuseppe SarpietroShigehisa AokiGiuseppe SciamannaShigekazu SuginoGiuseppe SiragoShigeki KamitaniGiuseppe SolarinoShigeki MatsubaraGiuseppe SuariaShigeki SakaiGiuseppe TalaniShigeki SetoGiuseppe Trusso SfrazzettoShigeki SugawaraGiuseppe ZanottiShigemi SeoGiuseppe ZardoShigeo S. SuganoGiuseppina AndreottiShigeru HiranoGiuseppina BasiniShigeru KakutaGiuseppina CantarellaShigetoshi YokoyamaGiuseppina MalcangiShih Hsiung WuGiuseppina MartellaShih Jie ChouGiuseppina NicoliniShih-Che SueGiuseppina PalladiniShih-Feng ChouGiuseppina RaffainiShih-Heng ChenGiuseppina SabatinoShih-Hsien HsuGiusy TassoneShiho NaitoGiusy TornilloShihori TanabeGizak AgnieszkaShi-Hua XiangGizella JahnkeShihuo LiuGladys BlockShih-Yi HuangGladys KoShih-Yu HuangGladys Oluyemisi Latunde-DadaShijie HeGlatz AttilaShijie YuGlauco ChisciShijun ZhongGleiston DiasShiliang LiuGleitz Hélène F. E.Shilie PanGlen BorchertShilpa BishtGlenn J. ThorlbyShilpi AgrawalGlenn R. ValdezShilpi GiriGloria Álvarez-SolaShilpi JainGloria ArriagadaShimi MeleppattuGlória ConceiçãoShimon EfratGloria E. O. BorgstahlShimpei HigoGloria Huerta-AngelesShin EnosawaGloria ManzottiShin GotoGloria MelziShin HamamotoGloria PascualShin MatsubaraGlória QueirozShin MorizaneGloria SalazarShin TakasawaGniewko WięckiewiczShin TaketaGobinath ShanmugamShindu ThomasGobinda SarkarShingen NakamuraGodwin W. NchindaShing-Hwa LiuGoedele MaertensShingo HatakeyamaGoh Eun ChungShin-Ichi AizawaGohar FakhfouriShin-Ichi AkanumaGokce Altay TorunerShinichi EnamiGökçe Şeker KaratoprakShin-Ichi HiranoGökçen Dilşa TuğcuShinichi MiyazawaGokhan GorgisenShin-Ichi NayaGokhan OzunerShinichi SakamotoGoki SudaShin-Ichi YokotaGoncalo BarretoShin-Ichiro FujiiGonçalo DiasShinichiro HayashiGonçalo GarciaShin-Ichiro KarakiGonçalo J. JustinoShinichiro KashiwagiGonçalo ValeShinichiro MorichiGong ZhangShinichiro OchiGongqin SunShinichiro OgawaGontzal García Del CañoShin-Ichiro OzawaGonzalo Joaquín Mena RejónShining LooGonzalo MardonesShinji NagataGonzalo Martínez-RodríguezShinji OhtaGonzalo Recabarren GajardoShinji SakaiGopal Lal KhatikShinji TakamatsuGopal SinghShinji YokoyamaGopalakrishna AkurathiShinjini GangulyGopichand GuttiShinn-Jyh DingGopinath KrishnasamyShinnosuke NishimuraGopinath M. SundaramShinobu OhnumaGopinathan JanarthananShinobu SatoGoran BaranovićShinobu TakizawaGoran GajskiShinsaku ItoGöran LarsonShinsuke NirengiGöran LaurellShintaro PangGoran MitulovicShintaro SukegawaGoran SedmakShinu PottathilGordana ĆetkovićShin-Wei WangGordana GajicShinya HasegawaGordana KočićShinya ImadaGordana Wozniak-KnoppShinya WadaGordon L. KleinShiqiang GaoGordon T. YeeShiran ShettyGordon W. IrvineShiri LiGoreti PereiraShiro MizunoGorkem MemisogluShisong RongGosteva AlevtinaShiu Cheung LungGöte SwedbergShiv Dutt PurohitGotthold GäbelShiv Prakash VermaGour Mohan DasShiv VermaGourav BhardwajShiva AziziniaGouse Peera ShaikShiva Hadi EsfahaniGoutam MukherjeeShivalee DuduskarGouveia Zelia LiciniaShivanand M. PudakalakattiGovinal Badiger BhaskaraShivanand PudakalakattiGovind ChilkoorShivananda KandagallaGraça BaltazarShivangi InamdarGraça SoveralShivani BansalGrace FarhatShivankar AgrawalGrace OdongoShivendra KumarGraciel D. DiamanteShivshankar ThanigaimaniGraciliana LopesShiwani SharmaGracjan MichlewskiShiwei ZhuGracjana Krzysiek-MaczkaShixiang SunGrady NelsonShixing YangGraeme BolgerShixiong WangGraham BurgessShixue WangGraham O'NeillShiyi YinGraham R. WallaceShiyong WuGranger SuttonShizuka UchidaGrant R. GordonShlomo SelaGrasiele SausenShoaib FreedGratiela PircalabioruShobini JayaramanGrazia FazioShofiul AzamGrazia Giuseppina PolitanoShogo NakanoGrazia SerinoShohei MatsuuraGraziana Da RoldShohei MitaniGraziana GattoShoib BabaGraziano GrugniShoichiro TangeGraziella TuratoShoji MaeharaGrażyna BudrynShoji OdaGrażyna DąbrowskaShoji OuraGrażyna JanikowskaShokouh AttarilarGrażyna Jarząbek-BieleckaShona MookerjeeGrazyna LietzauShoshana Revel-VilkGrazyna NiewiadomskaShosuke ItoGrażyna NowickaShouguo GaoGrażyna Odrowaz-SypniewskaShoukui HeGregg RomanShovan NaskarGregor NorcicShowkat Ahmad GanieGregorio HuerosShow-Mei ChuangGregorio Orozco-ArroyoShozo OzakiGregorio VonoShozo YanoGregory ArmstrongShraddha ParateGregory BarshteinShravan GiradaGregory BowdenShravan MorlaGregory CaputoShreesh K. OjhaGregory F. WeberShreyas GaikwadGregory GrafShri Mohan JainGregory J. TsayShrikant PradhanGregory JohnsonShrikanth GadadGregory MichelottiShriram VenkatesanGrégory PouriéShrishti SaxenaGreice K. B. Da CostaShruti AryaGreta FaccioShruti ChandraGrigorios KorosoglouShtaywy AbdallaGrigorios L. KyriakopoulosShu FangGrigoris AmoutziasShu WangGrinshpun AlbertShuai GaoGrisha PirianovShuai MaoGrissell TiradoShuai RenGrit WaltherShuaiming HeGrosshans JörgShuangxi WangGrzegorz BartoszShubha NageswaranGrzegorz CholewinskiShubha Ranjan DuttaGrzegorz CiesielskiShubhagata DasGrzegorz CzernelShubhmita BhatnagarGrzegorz GrynkiewiczShubing LiuGrzegorz GrześkShu-Ching HsuGrzegorz J. LisShu-Chuan LiaoGrzegorz JakubiakShue WangGrzegorz KarwaszShuen-Ei ChenGrzegorz KowalukShufa XuGrzegorz ŁysiakShu-Fen ChuangGrzegorz MikiciukShu-Fen PengGrzegorz PanasiewiczShu-Hao HsuGrzegorz PawlikShuhng SunGrzegorz SumaraShuhua BaiGrzegorz TeresińskiShu-Huei KaoGrzegorz TrybekShuichi NakamuraGrzegorz WegrzynShuichi SakamotoGrzegorz ZapotocznyShuijin HuaGrzegorz ŻurekShujaat AhmadGuadalupe Alvarez HernanShuji MizumotoGuadalupe HerreraShuji OginoGuadalupe Rojas-SanchezShuji OzakiGualtiero AlvisiShuji Tachibanaki　Gualtiero GualtieroShujiro HayashiGuan WangShujuan ChenGuanbin SongShu-Jui KuoGuan-Feng WangShukho KimGuang LiShu-Lan YehGuang ShiShuleiko DmitriiGuang YangShu-Ling TzengGuangda PengShun MaekawaGuangde JiangShun SongGuangkui XuShunbin XiongGuangsheng YuanShun-Fa YangGuangwu ZhaoShunichiro AsaharaGuangzhao LiShunichiro ItoGuanhong WangShunji HasegawaGuan-Jhong HuangShunji NatsukaGuannan WangShunnichi KashidaGuan-Ting PanShun-Qing LiangGudrun KoehlShunqing TangGudula SchmidtShunsuke YajimaGuendalina LucariniShunyi JianGuendalina ZuccariShunyi ZhuGuennadi SaikoShuohui LiuGuglielmo DurantiShuowei CaiGuglielmo SorciShuqi LiGuidenn SulbaranShusei KanieGuido BispingShusuke TodenGuido DurianShuvadeep MaityGuido GembilloShuvashis DeyGuido MollShuvasree SenguptaGuido PanzarasaShu-Yen LinGuido VanhamShveta BathlaGuido VeitShweta Anil KumarGuido VielShweta Pankajkumar LavaniaGuido ZavattaShweta RamdasGuihong BiShweta TripathiGuilherme Volpe BossaShwu-Chen TsayGuillaume E. CourtoyShwu-Jen LiawGuillaume FichesShyam Kishor SahGuillaume GiraudShyam Kumar GudeyGuillaume St-JeanShyam MoreGuillaume VaresShyam S. SamantaGuillaume VivesShyamala ThirunavukkarasuGuillermina BurilloShyamsundar Pal ChinaGuillermina Ferro-FloresShyh-Jiun LiuGuillermo AhumadaShyh-Shin HwangGuillermo Fuentes-DavilaShyi-Long LeeGuillermo GervasiniShymaa EnanyGuillermo HinojosaShyng-Shiou F. YuanGuillermo López LluchShyue-Fang Battaglia-HsuGuillermo Padilla-GonzalezSi ChenGuillermo PetzoldSi Hua JinGuillermo Santos-SánchezSi Ming ManGuiping ZhaoSi Nian CharGuiseppe PoliSiamak Shirani BidabadiGuiying HeSian FrosiniGu-Jiun LinSibhghatulla ShaikhGül Fatma YarımSibin YuGulab Chand AryaSiddharth JaggavarapuGulam Mohmad RatherSiddharth Kaushal TripathiGulam RabbaniSiddharth NarayananGulevich AlexanderSiddhartha KunduGulzar Ahmad BhatSiddhita D. MhatreGulzhanat AimagambetovaSideris FotiadisGuna LaganovskaSidi ZhuGunawan Setia PrihandanaSidney M. Gospe IiiGundula Schulze-TanzilSiemowit MuszyńskiGunendra Prasad OjhaSiew-Keah LeeGunhild Von AmsbergSigit PrastowoGunjan AroraSigita JurkonienėGunjan PurohitSigne Holm NielsenGunnar HouenSigrid HeuerGunnar PejlerSihai ZhaoGunnar RonquistSihong SongGunnar StèineckSihui MaGunnar WichmannSijin GuoGünter MüllerSijo MathewGunter SchmidtkeSikandar I. MullaGünther GerischSi-Kao GuoGünther MuthSildivane Valcácia SilvaGünther SchmalzingSilva Katuŝić HećimovićGünther ZeckSilvana AlfeiGuochong JiaSilvana ZanlungoGuodong ShenSilvania LanfrediGuofu HuSilvano BertelloniGuo-Fu LiSilvano DragonieriGuohao HeSilvano GeremiaGuohao WangSílvia A. SousaGuohua LuoSilvia ActisGuoli ZhengSilvia AlbertGuo-Liang LuSilvia Alberti-ViolettiGuoquan LiuSilvia Alice LocarnoGuosheng CaoSilvia BarbonGuotao PengSilvia BianchiGuowei YinSilvia BistiGuoxiang JiangSilvia BusomsGuoxun ChenSilvia CapannaGuo-Zhang ZhuSilvia CarlottoGuozheng WangSilvia CarvalhoGurdeep MarwarhaSilvia CasadeiGurdeep SinghSilvia CastanyGurinder SinghSilvia CondeGurioli GiorgiaSilvia CorrochanoGurkan MollaogluSilvia Del RyGurpreet Kaur AroraSilvia Di AgostinoGurtej DhootSilvia Di GiacomoGuru Prasad SharmaSilvia DottiGurudeeban SelvarajSilvia DragoniGurunathan ThangavelSilvia FilippiGurusamy SaravanakumarSilvia FischerGustavo Alberto ChiabrandoSilvia Gatti-McarthurGustavo AmoresSilvia GazzinGustavo ArocaSilvia Gómez-JiménezGustavo EgeaSilvia GrassilliGustavo FerminSilvia Jiménez-MoralesGustavo FernandesSilvia LandiGustavo GlusmanSilvia LeonciniGustavo SantoyoSilvia LiskovaGustavo VarcaSilvia LiuGutkowska MałgorzataSilvia Lucena LageGuus KochSilvia MarracciGuy GartySilvia MartuGuy J. SmaggheSilvia NatoliGuy LadamSilvia NovíoGuy LemaySilvia PampanaGuy RostokerSilvia PanseriGuy ServantSilvia PasquiniGuzel ZiyatdinovaSilvia PiantoniGvozden RosicSilvia Portela-BensGwang Hyeon EomSilvia ProiettiGwendolyn Vaughn ChildsSilvia RiondinoGyan Prakash MishraSilvia RussoGynendra Kumar RaiSilvia SavastanoGyöngyi ZoltánSilvia SvegliatiGyörgy BaloghSilvia ToricesGyörgy DormánSilvia TortorellaGyörgy KeglevichSilvia TurcoGyörgy KemenesSilvia VasiliuGyorgy PanyiSilvia Vidal-AlcorisaGyörgy Tibor BaloghSilvia WürstleGyörgyi MűzesSilvia ZorrillaGysbert Botho Van SettenSilvio TundoGyudo LeeSimcha Lev-YadunGyula BattaSime UkicGyungsoon ParkSimhachalam GurugubelliH M Arif UllahSimin SharifiH. Dayton WildeSimm StefanH. Denis AlexanderSimon BoudreaultH. M. M. Abdel-AzizSimon BucherH. M. Prathibhani C. KumarihamiSimon C. AndrewsH. M. Prathibhani Chamidha KumarihamiSimon Chi Chin ShiuH. Michael G. LattorffSimon DannerH. PortierSimon FoxHabib MotieghaderSimon Gracia LorenaHabib PathanSimon John Christoph SoerensenHabibu MugerwaSimon KajaHabib-Ur-Rehman AtharSimon PfistererHabil. Agnieszka Dettlaff-PokoraSimon RichardsonHadi HadshemzadehSimon SteinbergHadi Pirasteh-AnoshehSimon VölklHadi RastinSimon W. RabkinHaeng-Hoon KimSimón Yobanny Reyes-LópezHae-Rahn BaeSimona AlibrandiHaewon ByeonSimona BernardiHae-Won KimSimona BuelliHaeyong KweonSimona BungauHafiz Muhammad Salman AjmalSimona CameroHafiz Umer JavedSimona CapsoniHafize Nagehan KoysurenSimona CeccarelliHagir B SulimanSimona ClichiciHai An TruongSimona ConcilioHai DuSimona DanieleHai LiSimona De De VitaHai LiuSimona Delle MonacheHai Long WangSimona DinicolaHaibo YangSimona ErricoHaichao ZhaoSimona FerraroHaidong GuSimona GalloHaidong YanSimona JancikovaHaifei ShiSimona LucioliHaigh TonySimona MartinottiHaim BreitbartSimona MirelHaim WernerSimona NicoaraHaipeng LiuSimona RapposelliHaipeng XiaoSimona SagonaHaipeng ZhangSimona SerratìHaiqing ZhaoSimona TafuriHaiqiu HuangSimona VarrialeHairul Islam Mohamed IbrahimSimona ZaamiHaissi CuiSimone BattagliaHaisu ZengSimone BeninatiHaitao LuanSimone BiniHaiteng DengSimone BrogiHaitham E. M. ZakiSimone CantamessaHaiwei LuSimone CarottiHaixiao HuSimone CarradoriHaiyang MaoSimone Ciofi-BaffoniHaizhen Andrew ZhongSimone Di MiccoHaizhou LiuSimone FattoriniHajeewaka C. MendisSimone GalloHajer AmmarSimone GrassiHaji A. AisaSimone LandiHajime KonoSimone LutiHajime NagasuSimone MaestriHajime YanoSimone MirabiliiHak Jun KimSimone SavastanoHakan AldskogiusSimone SavinoHakan F. ÖztopSimone SforzaHakim MahammediSimone WajnerHakim ManghwarSimonetta FornariniHakim Ouled-HaddouSimonetta MucciforaHala ChaabanSimonida TomićHala M. F. MohammadSimons SvirskisHala ShokrŠimons ŠvirskisHalil I CiftciSimote FoliakiHalil Ibrahim CeylanSimran VenkatramanHalima KerdjoudjSina NaserianHalina AntonSina RashediHalina DobrzynskiSina ShadfarHalvard BönigSina TaefehshokrHamdan Z. HamdanSindy KuehHamed GhafarifarsaniSinead O’DonovanHamedreza JavadianSingh AkHamid IrannejadSiniša DodićHamid MaadiSinu KumariHamidreza Montazeri AliabadiSiok-Fong ChinHamidreza PirayeshSiqi HuoHamish S. SutherlandSiqi WuHamutal MazrierSiranjeevi NagarajHamza El HadiSireesha ManneHamzeh AlipourSirijan SantajitHamzeh GhorbaniSiro LuvisettoHan HanSiroj BakoevHan PengSishuo WangHan WangSiti Maznah KabebHan ZhangSiti Nooraya Mohd TawilHana AlharbiSiti Nuurul Huda Mohammad AzminHana DrahovskáSitong ZhouHana FakhourySitthichok ChaichuleeHana KolesováSittinan ChanaratHana PopelkaSiu Hong Dexter WongHana Van CampenSiu Hong WongHanae Naceiri MrabtiSiva Karthik VaranasiHanan Hassan Abd-ElhafeezSiva Kumar SolletiHanan Osman-PonchetSiva Prasad PandaHan-Fang WuSiva S. PandaHang HeSivanesan DakshanamurthyHang Soo ParkSivaraj Mohana SundaramHang XingSiwapon SrisonphanHang YangSiwei ZhangHan-Gang YuSiyi GuHanguang ZhangSiyong KimHanguo XiongSiyuan ZhanHani Nasser AbdelhamidSiyuan ZhaoHanish JainSiyuan ZhengHan-Jia LinSj Sijie ShenHan-Joo MaengSjoerd RijpkemaHan-Jun KimSjoerd T. T. SchettersHankyu LeeSlađan PavlovićHanley N. AbramsonSladjana JevremovicHanmant GaikwadSladjana TanaskovićHan-Mou TsaiSlavica KranticHanna FuchsSlavica VaselekHanna HuebnerSlavko RadenkovićHanna Jaaro-PeledSławomir BorekHanna JulienneSławomir CzarneckiHanna KmitaSławomir DreslerHanna KuligSlawomir GonkowskiHanna MannellSławomir GonkowskiHanna SulewskaSławomir JakiełaHannah K. DrescherSławomir KociraHann-Chorng KuoSławomir KujawskiHanne DiliënSławomir KulaHannes De DeurwaerderSławomir MichałekHanno M. WitteSlawomir MilewskiHanno SchäferSławomir MilewskiHanns MoshammerSlawomir OrzechowskiHanqing ChenSlawomir SekHanquan SuSławomir ŚwierczyńskiHans BakkerSławomir ZduńczykHans Christian HenniesSławomir ZmonarskiHans De CockSławomira Drzymała-CzyżHans G. DrexlerSmail Hadj-RabiaHans Georg MannherzSmaoui SlimHans H. HendriksSmaranda BuduruHans Henning Von HorstenSmijin Karthully SomanHans KnechtSmijin SomanHans ReinkeSmita NairHans Van LeeuwenSmita S. KulkarniHans VliegenthartSmrithi PadmakumarHans-Christian SiebertSmriti PandaHans-Gert BernsteinSmriti PriyaHanshi XuSmriti SinghHansi WeissensteinerSmrutisanjita BeheraHans-Joachim SchulzeSneha PillaiHansjörg LehnherrSneha RatnapriyaHans-Jörg MaiSneha SaxenaHansjörg SchwertzSnehal K. ShuklaHans-Matti BlenckeSnehal NirgudeHanwei JiaoSnejana GrozevaHany Abd El-LateefSnežana D. SavićHany Abdel-LatfSnezana DjordjevicHany Ezzat KhalilSnezana Ilic-StojanovicHany G. Abd El-GawadSnežana M. MiloševićHany H. ArabSnježana KerešaHany Hamdy ArabSnježana Mardešić-BrakusHany I. SakrSo Hee KwonHany M. R. Abdel-LatifSo Yon ParkHany MareiSobhi F. LamlomHanyi DuanSobhi M. GomhaHanying ChenSobia NiaziHanyong PengSobia NoreenHao ChenSobuj MiaHao LiSocorro Retana-MarquezHao NieSofia AlmqvistHao TanSofia Barbosa-GouveiaHao TianSofia CanolaHao YangSofia Costa-De-OliveiraHao ZhangSofia FranciaHao-Ai ShuiSofia GuedesHao-Ching WangSofia KhaitlinaHao-Jui WengSofia M. E. WeilerHao-Kun LiuSofia MitakouHaomin LiuSofia MorozovaHaoshen ShiSofia NolascoHao-Wen SimSofia PavanelloHaoyi WangSofia RangouHaq Abdul ShaikSofia SaltaHaradhan KolyaSofia SoaresHaralampos M. MoutsopoulosSofia SousaHarald KellerSofia VasilakakiHarald PlattaSofie ZehentnerHarald SchulzeSofya MorozovaHarald W. PlattaSoha AhmadiHardik AminSohail AhmadHardy RideoutSohail MumtazHari Krishna NamballaSohail NadeemHari Prasad DevkotaSohail QaziHarichandra TagadSohail YasinHarini RamalingamSohan SethHarish MudilaSoheb Anwar MohammedHarit PandaSoheil Abbaspour-RavasjaniHarlan J. ShiauSoheil MohammadiHarley L. WorthySohel M. JuloviHarminder SinghSoher Nagi JayashHarold J G MeijerSohi KangHarold W. DavisSohini ChakrabortyHaroon ButtSohrab RahimiHaroutioun AskanianSohsuke YamadaHarpreet KaurSoibam Khogen SinghHarpreet SinghSoichiro MurataHarri H. HakovirtaSoichiro NaganoHarriet Wikman-KocherSoile Jokipii-LukkariHarris PratsinisSokhee JungHarry SteinbuschSolange MagalhaesHarrys Kishore Charles JacobSoleiman MoslehHarshal NandurkarSolmaz MalekiHarshani WijerathneSolomon AfelikHarsharn GillSolveigh C. KoeberleHarshraj ShindeSom DevHartmut KuhnSoma GhoshHaruaki KitagawaSomaira NowsheenHaruhide MoriSomashekar G. KrishnaHaruhiko AsakawaSomasundaram PrasadhHaruhiko SugimuraSomayyeh AsgariHaruki KoikeSomchai ChutipongtanateHaruki UsudaSomdet SrichairatanakoolHarumasa YokotaSomdutt MujwarHaruyasu AsaharaSomiranjan GhoshHarvey G. RowethSomnath PandeyHasan DemirciSomnath SinghHasan F. AlesarySompit WanwongHasan Nudin Nur FatihahSomya AggarwalHasan SadeghifarSoňa ČiernikováHaseeb ZubairSoňa LegartováHashaam AkhtarSonal DalviHassan AlbarqiSonal DeshpandeHassan DastsoozSonal ManoharHassan El-RamadySonal SukreetHassan KassassirSonali P. BarweHassan YousefiSonam KumariHassan ZafarSonam ShakyaHassen S. WolleboSonata JarmalaiteHatasu KobayashiSondavid NandanwarHatem FouadSondra T. BlandHathal HaddadSong CaiHauet ThierrySong Cheol KimHåvard GautnebSong YangHåvard J HaugenSongbo WangHaw-Ming HuangSonghui JiaHaya Lorberboum-GalskiSongli ZhuHayato TerayamaSonglin YuHaydar AkcaSonglin ZhangHaydeé Rosas-VargasSoni DhruvkumarHayley L LetsonSonia A. MeloHayrettin O GulcanSonia AroniHazel QuekSónia C. CorreiaHazel SiveSónia CarabineiroHazem ElkadySonia Claudia Nascimento De QueirozHazem S. ElshafieSonia Di GaetanoHe YinSonia EirasHe ZhuSonia FantoneHeather HundleySónia G. PatrícioHeather M. BurrowSonia KiranHeather M. HowerSonia L. AlbarracínHeather Stevenson-LernerSonia LeviHeather WilkinsSonia MartialHeba A. GadSonia Navas-MartinHeba AbdelrazikSonia PinhoHeba EbeedSonia RochaHeba M. IsmailSonia Torres-SanchezHeba MohamedSonia ValletHeba SahyonSonja BuvinicHebah Al UbeedSonja GrljušićHebat-Allah A. HusseinSonja HeiblHéctor BurgosSonja HinzHéctor CandelaSonja Milic KomicHéctor Capella-MonsonísSonja SucicHector EscrivaSonu Menachem Maimonides BhaskarHéctor García-OrtegaSonu SinghHéctor M. Mora-MontesSonu SubudhiHector ValenzuelaSoo Je ParkHedia ZitouniSoo Min KimHedwig Sutterlüty-FallSoochong KimHedwin Kitdorlang DkharSoo-Hyun SungHee Kyoung JooSoon Jae KwonHee-Joon KimSoon Wook KwonHee-Moon ParkSopan WaghHeewon JungSophia BorisevichHeeyoun HwangSophia ChatziantoniouHeeyoung ChaeSophia M. SchmitzHefei ZhaoSophie BrouilletHehuang XieSophie Groux-DegrooteHeidi KaljunenSophie ThenetHeidi StöhrSophie Thetiot-LaurentHeike EndepolsSorana BolboacaHeike KnottSorana Maria BucurHeike KölbelSoraya L. VallesHeike KroegerSoraya PazHeike NiessnerSoraya ScuderiHeiko H.W. HenningSorel Sagu TchewonpiHeiko SorgSorin HostiucHeinrich JungSorin MunteanHeinz D. OsiewaczSorina DinescuHeinz SillSorina Mihaela PapucHei-Sung KimSotaro FujiiHelber Enrique Balaguera-LópezSotirios ApostolakisHelen BirchSotirios G. ZarogiannisHelen BoldinghSotirios KakavasHelen FogartySotirios PopeskouHelen IrvingSotirios SotiriouHelen KaliraiSotiris AmillisHelen VidotSotiris K HadjikakouHelena BarrosoSoubantika PalchoudhuryHelena BujdákováSoumaya KouidhiHelena CoelhoSoumen DasHelena FelgueirasSoumen GhoshHelena FernándezSoumen SahaHelena G. AzinheiraSoumita GhoshHelena KorpelainenSoumya IyerHelena MartynowiczSoumyadev SarkarHelena Mertlíková-KaiserováSoumyananda ChakrabortiHelena NejezchlebováSounak SahuHelena OstolazaSourav ChowdhuryHelena PortaSourav RejHelena SoaresSourav RoyHelena Susana Costa Machado FerreiraSourish GhoshHélène Dubois-Pot-SchneiderSousuke ImamuraHélène LemieuxSouvick RoyHélène Niculita-HirzelSouvik DharHélène RoumesSouzan Vergkizi-NikolakakiHelge AmthorSovetgul AsekovaHeli KangasSowjanya ThatikondaHélia CardosoSowmya PoosapatiHélio SantosSowmya ViswanathanHellevi PeltoketoSowmyalakshmi SubramanianHelmut H. TelleSpaska StanilovaHelmy YoussefŠpela KonjarHeloisa Sobreiro Selistre De AraujoSpencer GibsonHem Dutt ShuklaSperanza EspositoHemant KardileSpiridon MantzoukasHemant KulkarniSpiro M. KonstantinovHemant Kumar MishraSpiros G. PneumaticosHemant PrajapatiSpiros ParamythiotisHemerson Iury Ferreira MagalhãesSpomenka ManojlovićHemn MohammadpourSpring R. FarrellHend AbdelhakimSpyridon A PetropoulosHeng LuoSpyridon StavrouHeng ZhaoSpyros D. GeorgatosHengbin WangSpyros E. ZographosHengwu JiaoSravani AdusumalliHenk KoppelaarSrba MijailovichHenk S. BrandSrecko GajovicHenning ArltSree V. ChintapalliHenning ReisSreejith SivaramapanickerHenri GruffatSreelakshmi SreenivasamurthyHenrich FrielinghausSreelakshmi VasudevanHenricus MutsaersSreepoorna UnniHenrik C. BäckerSreerama ShettyHenrik LauridsenSreten TerzićHenrik PedersenSridhar ChandrasekaranHenrike LucasSridhar MuthusamiHenrique Andrade FonsecaSridhar VadahanambiHenrique FernandesSridhar VemulapalliHenrique Leonel GomesSriganesh Ramachandra RaoHenrique SilvaSrihari KonduriHenry Hang Fai KwokSriharsa PradhanHenry HirschbergSrijani BasuHenry J. ThompsonSrijon BanerjeeHenry P. GodfreySrinath PalakurthiHenry PleassSrinath PashikantiHenry SutantoSrinivas AjjarapuHenryk GrajekSrinivas Gorur-ShandilyaHerbert Matthias RieplSrinivas MummidiHerbert Ryan MariniSrinivas NellimarlaHerbert Ryan MariniSrinivas PittalaHeriberto CastroSrinivas SistlaHeriberto Hernández-CocoletziSrinivasa Rao RaoHerlinda Bonilla JaimeSrinivasa Reddy BonamHerman K. EdskesSrinivasan KrishnanHerman SchreuderSrinivasan RamalingamHerman SintimSrinivasrao GanipisettiHermann AmmerSrivastava SudhakarHermann KreyenbergSrjit DasHermann Von GrafensteinSruthy Maria AugustineHermona SoreqSsu-Wei HsuHernan Diego FolcoStan KailisHernan Mauricio RomeroStanescu Ana Maria AlexandraHernandez Paula A.Stanislav A. NikolaevskiiHerrera-Guerra AngelStanislav FilipHerson González-PonceStanislav KafkaHerui WangStanislav KatinaHerve LestunffStanislav KotlyarovHervé PerronStanislav MicudaHesham El –EnshasyStanislav N NaryzhnyHesham El-SeediStanislav RubakhinHesham HaffezStanislav S. TerekhovHeui-Soo KimStanislav VasilyevHicham LabaziStanislava PankratovaHideaki IjichiStanisław CzuczwarHideaki NakajimaStanisław DuerHidehiko KikuchiStanisław KaliszHidehiro OkuStanisław KrompiecHidekazu HiroakiStanisław MlekoHideki AitaStanislaw OłdziejHideki AmiiStanislaw SłomkowskiHideki KishimuraStankevičius VaidotasHideki ShibataStanley M HilemanHideki SugiiStathis FrillingosHideko SoneStavros J. BaloyannisHidenori SuganoStavroula TsitkanouHideo HashizumeStefaan W. Van GoolHideo KatoStefan ArnholdHideo NishitaniStefan BroerHideo SuzukiStefan Cristian VesaHideo TsukadaStefan De FolterHideo WadaStefan DüsterhöftHidetomi TeraiStefan Ernst SeemannHidetoshi KassaiStefan HadzicHideyuki InuiStefan HarsanyiHideyuki J. MajimaStefan HeberHideyuki KanematsuStefan HümmerHideyuki KinoshitaStefan LinderHideyuki SasakiStefan MöhlenkampHideyuki TakahashiStefan NicolauHieronim JakubowskiStefan PetersHie-Won HannStefan PuschHigashisaka KazumaStefan ReberHikaru SotomeStefan ReuterHilal Ahmad RatherStefan RiwaldtHilal TayaraStefan RöderHilario VidalStefan SimmHilda E. GhadiehStefan ThorHilde K. GaltungStefan W. VetterHimadri BiswasStefan WegerHimadri Sekhar SarkarStefania BrunoHimangshu SonowalStefania CantoreHimani PuniaStefania De LucaHimanshu BatraStefania GaldieroHimanshu GoelStefania GessiHimanshu KathuriaStefania GonfloniHimanshu Sekhar JenaStefania HanauHimashu SinghStefania LoretiHinojal ZazoStefania MarcuzzoHipolito Hernandez-HernándezStefania MarianoHira FatimaStefania MarsiliHiram Hernández-LópezStefania MerighiHiroaki InabaStefania Mirela MangHiroaki KitoStefania MocciaHiroaki KodamaStefania ScaliseHiroaki MaedaStefania SciallaHiroaki NekiStefania TegliHiroaki TaguchiStefania TroiseHiroe TobaStefania VitaleHirofumi ImaiStefania ZappiaHirofumi SawaiStefanie FenskeHirohiko TaniStefanie FlunkertHirohisa KishinoStefanie KleinHirohisa TamagawaStefanie LeschHirohito IshigakiStefano AlbertiHirokazu FukuiStefano AmenteHiroki IdeStefano BellucciHiroki SatoStefano BonassiHiroki TeraokaStefano BorocciHiroki ToyodaStefano CagninHiroki WakabayashiStefano CanosaHiroki YokotaStefano CasalottiHiroko Ishiwata-EndoStefano CastellaniHiroko KodamaStefano CivolaniHiroko TsudaStefano FaisHiromi SakaiStefano Forte ForteHiromi SatoStefano GarofaloHiromi ShimojoStefano GastaldelloHiromichi YumotoStefano GrolliHiromune AndoStefano GuandaliniHironobu AsaoStefano LeporattiHironori YoshinoStefano LettieriHiroo ImaiStefano LuinHiroshi FukushimaStefano MartellucciHiroshi HaraStefano MarulloHiroshi KeinoStefano MazzoleniHiroshi KitagakiStefano MusardoHiroshi MaitaStefano P. PiottoHiroshi MiyanishiStefano PepeHiroshi NonoguchiStefano PiazzaHiroshi SakaueStefano QuartaHiroshi ShimodaStefano RavaioliHiroshi TakemoriStefano RestainoHiroshi TanahashiStefano SalgarelloHiroshi WakabayashiStefano SantabarbaraHirotaka IshiiStefano SotgiuHiroto OhguchiStefano TambuzziHirotomo KatoStefano TizianiHiroyasu YamaguchiStefano VarrellaHiroyoshi Y. TanakaStefanos KikionisHiroyuki AonoStefanos LeontopoulosHiroyuki FukuiSteffen DaubHiroyuki KanzakiSteffen JunkerHiroyuki KoshinoSteffen MaakHiroyuki KurataSteffen PockesHiroyuki NakamuraSteffen WagnerHiroyuki OkadaSteffi M. UrbschatHiroyuki OkamotoStefka D. SpassievaHiroyuki TanakaStela DimitrovaHiroyuki TobitaStelian Sergiu MaierHiroyuki TomitaStelian VladHiroyuki TsuchiyaStella CascioferroHiroyuki WakiguchiStella LeeHisahide NishioStella María Fuensalida NovoHisako FujiharaStella Mitrani-RosenbaumHisashi AshidaSteluta Carmen CiobanuHisashi HaradaSten AndréassonHisashi IzasaSten SiikavuopioHisashi KawasakiStepan DzhimakHisashi MatsudaStepan FeduniwHisashi NishiwakiStepan GambaryanHisato YagiStephan FuhrmannHisayuki KomakiStephan GrosseHisham AltayebStephan ImmenschuhHisham BahmadStephan KellenbergerHishinuma ShigeruStephan LeisengangHitendra Singh SolankiStephan LudwigHitler LouisStephan PhilippHitoshi OzawaStephan ReshkinHitoshi ShirakawaStephan SchmidHiu Chuen LokStephan Von HörstenHiu-Yee KwanStephan WittHla Myo TunStephane BelinHo Bang KimStephane BolducHo Jin LeeStéphane BolducHo Leung NgStéphane ChavanasHo Soo KimStephane LemiereHo TsoiStéphane MandardHoai TranStephane R. GrossHoang Thi Minh HienStephane RetyHoang-Long DuStephane ServaisHoi Leong Xavier WongStéphane TéletchéaHo-Kyung LimStephanie A. BoothHolger A. LindnerStephanie BaudHolger SchulzeStephanie DauthHolger ZetzscheStéphanie DavalHolly A. ShillStephanie DuguezHomira BehbahaniStephanie G. ConeHon Cheong SoStephanie KingHon Yeung ChanStephanie LhezHong ChengStephanie McardleHong DuStephanie MenikouHong DuanStéphanie RaymondHong GuoStephanie Rodríguez BesteiroHong HeStéphanie ToméHong JinStephanie WattsHong LiangStephen AndersonHong LuStephen B. CarrHong WanStephen BadylakHong YuStephen BoultonHongbin ZhangStephen C. HardiesHongbing FanStephen ChimHongbing LiuStephen CrookeHongbo ChenStephen D BarrHongbo JiangStephen FairweatherHongchang CuiStephen H. PoorHongda CaoStephen J BushHong-Gu JooStephen J TerryHonghe LiuStephen L. CameronHongju JianStephen LewisHong-Ju YinStephen MathewHonglian YeStephen P. FicklinHongliang HeStephen PearceHongmei YangStephen S. PalmerHongmin WangStephen ThankachanHongmin WuStephen WorrallHongnan CaoStepheny ZaniHongping ChenStergios BoussiosHongping JinStergios KatsiougiannisHongqiao ZhangSteve ElderHongsheng MenSteve GameiroHongsu WangSteve JefferyHong-Viet V. NgoSteve LeuHongwei LiSteve ScheinerHongwei ZhangSteve ShnyderHongxia WangSteve T RussellHongyan RenSteve WalkerHongyan ZouSteve WiltonHongyang XuSteven A. BloomerHongyi ZhouSteven BradfuteHongyue ZhouSteven F. GameiroHongzhe SunSteven FeldmanHongzhu CuiSteven HeatonHonoo SatakeSteven LevyHooi-Leng SerSteven M. GravesHoon HurSteven M. PascalHoon KimSteven N. FieringHoracio BachSteven PattersonHoracio FirminoSteven R. DuncanHoratiu OlteanuSteven R. Van DorenHorst OlschewskiSteven RothHortensia De La FuenteSteven SilversteinHortensia T. Sanchez-TocinoSteven SuHosam O. ElansarySteven T. LeachHoson LamSteven W. BucknerHossam DonyaSteven WhittenHossein AhangariSteven WillowsHossein AziziSteven WolfHossein HosseinkhaniSteven ZielskeHossein MolaviStig LinderHossein SabouriStojan PetkovićHossein Tabatabaei-JafariStojanovska VioletaHou ZehaoStone ElworthyHou-Guang ChenStoyan PetkovHoupu LiStrzemski MaciejHouria DaimiStuart CretonHouwen PanStuart DryerHoward A. YoungStyliani GeronikolouHoward E. BoudreauStylianos PouliosHoward LindsaySu Fern TanHrvoje LepedušSu LiHrvoje RimacSubba Rao CheekatlaHsi ChangSubban KathiravanHsiang-Ruei LiaoSubbarayalu PanneerdossHsiaohang ChungSubendu SarkarHsiao-Hsuan KuoSubhadeep ChakrabartiHsien-Ming WuSubhadip MukhopadhyayHsien-Wei YehSubhajit GhoshHsien-Yuan LaneSubhash TripathiHsin-An ChangSubhashree SubramanyamHsin-Chen LeeSubhasis BiswasHsin-Chih LaiSubhasish SahaHsing-Yin ChenSubhrajit SahaHsin-Yao WangSubir Kumar JuinHsin-Yu HoSubodh DubeyHsin-Yuan ChenSubodh K. SamratHsiu-Chuan ChouSubogh SinhaHsiu-Mei ChiangSubramanian DhandayuthapaniHsiuying WangSubramanya PandruvadaHsiyuan HuangSubramanyam DasariHsuanping ChangSubrat BhattamisraHsueh-Ping ChuSubrat Kumar PattanayakHsueh-Wei ChangSubrata DebHsu-Heng YenSubrata MondalHu HuangSubroto ChatterjeeHu LiSuchismita RahaHu ZhaoSuchismita RoyHua HuangSuchithra Ashoka SahadevanHua LiSuchitra JoshiHua SuSudarshan BhattacharjeeHua WangSudarson Sekhar SinhaHua YangSudeshna SahaHua ZhuSudha KumariHuacheng ZhangSudhanshu RaikwarHuadong PengSudheendran MavilaHuafeng WangSudhir AggarwalHuai-An ChenSudhir K. UpadhyayHuaijun SiSudhir PandeyHuaiping ShiSudhirkumar YanpallewarHuaizhe YuSudi PatelHuajin ShengSudip BanerjeeHuali SuSudip DuttaHuan WangSudip MondalHuan ZhangSudip SenHuanan ShiSudip ShethHuang BinquanSudipta PanjaHuang Chung-HsiungSue NangHuanhuan FengSue Rutherford SiegelHuanmin ZhangSue W. LiebmanHuan-Xiang ZhouSufang LiuHuaqiang YangSufang ZhangHuarong ChenSuhail AkhtarHua-Sheng ChiuSuhail MohdHua-Tao WuSuhrid BanskotaHua-Tao XieSui Kiat ChangHuaxin ShengSujay GuhaHuayu QiSujeenthar TharmalingamHubert SytykiewiczSujeet KumarHubert Van Den BerghSujin HyungHucheng ZhaoSujit SuklabaidyaHuei-Jane LeeSujung YeoHuei-Tzu ChienSukhbir KaurHueng-Chuen FanSukhvinder SinghHuey-Chun HuangSukhyun RyuHuey-Jen LinSukkum ChangHuey-Wen ChuangSulev KoksHugo A.L. FilipeSultan AkhtarHugo Arias-PulidoSultan DarveshHugo FragaSuman AlishettyHugo GermainSuman ChaudharyHugo Martínez-RojanoSuman ChowdhuryHugo SantosSuman SamantrayHugues Allard-ChamardSuman WattalHugues PetitjeanSumana GhoshHui DaiSumbul SaeedHui FengSumeet Kumar SinghHui LiSumeet NayakHui LuSumera QasimHui ShenSumesh ParatHui WangSumeyye CavdarliHui YeSumit DasHui ZhaoSumit MazumdarHui ZhuSumit MukherjeeHuib VersnelSumit MurabHuichen GuoSun ChengyiHui-Ching ChuangSun ImHuichun TongSunayana Begum SyedHui-Cong WangSunbin DengHui-Hua HsiaoSundar KunwarHuihua LiSundararajan ParaniHuihui FanSuneet KaurHuihui HuangSunetra SaseHuiling HongSung Eun KimHuilong WangSung Ho SongHuiming LuSung Hwan KiHui-Rong JiangSung Hyun KimHuiting ZhangSung Noh HongHuitong ShiSung Soo AhnHuixian HongSung Ui ShinHuiyin TuSung Un HuhHui-Yun ChengSung Woo LeeHulya CakmakSung-Beom ChoHulya IlbiSunghan KimHumberto Astiazaran-GarciaSung-Ho LeeHumberto Cavalcante JocaSung-Hoon KimHumberto HernandezSunghwan SuhHumberto PrietoSunghyun YoonHumberto Sanchez GonzálezSung-Jae LeeHume StroudSung-Jig LimHumira SonahSung-Ju AhnHung Huey-ShanSungjun JungHung-Chi ChengSung-Kun (Sean) KimHung-Chuan PanSung-Ryul KimHunge Yuvaraj M.Sungsin JoHung-Jen ChenSung-Woon OnHung-Jen LiuSungyul LeeHung-Pin TuSun-Hee LeemHung-Wei YangSunil Kumar SahuHung-Yi ChuangSunil Kumar SinghHung-Yin LinSunil Kumar SukumaranHun-Ju YuSunil S. GangurdeHuong Nguyen ThuSunilgowda Sunnagatta NagarajaHurija Džudžević-ČančarSunita KeshariHusanu ElenaSunney ChanHüseyin CanSunny AhmarHussain Yar KhanSunny Chi Lik AuHussein SabitSunny S. K. ChanHwayong LeeSunny SharmaHye Jin YouSun-Woong KangHye Kwon KimSun-Young LeeHye Kyoung ShinSupandeep Singh HallanHye One KimSupanji SupanjiHye Ryoun KimSuprasanna PennaHyemin KimSupratim BasuHyemin LeeSupravat MukherjeeHyemin LimSupriya ChakrabortyHyeong-Geug KimSupriya MahajanHyeonji KimSupriyo BandyopadhyayHyo Min ChoSupriyo RayHyoung Tae KimSuraj GuptaHyoung-Geun KimSuraj UmdaleHyun Chul ChoiSurajit BhattacharjyaHyun Ho YoonSurajit ChatterjeeHyun JoSuranga KodithuwakkuHyun Joo AnSurbhi VermaHyun Mi PyoSureewan SittijundaHyun Sook HongSurendra RajpurohitHyun Wook JungSuresh GorleHyungseok LeeSuresh KalathilHyun-Jin MinSuresh L MHyunjoong KimSuresh NarvaHyunju LeeSuresh PantheeHyun-Jung ParkSuresh Ponnayyan SulochanaHyun-Seok JinSuresh ThanguduHyunsoo KimSuriya RehmanHyunsuk ShinSuruchi SinghHyun-Woo ParkSurya Jyoti BanerjeeI. B. ObotSurya Vsrk PulavartiI. G. TiganovaSusan A. FarrI. P. KuranovaSusan BrownI. P. Shanura FernandoSusan E. LaflammeI. S. PoperechnySusan HawthorneIacopo GesmundoSusan L. CampbellIain A. M. MacpheeSusan SaraIain LamontSusan ZupIain Parry HargreavesSusana B. BravoIain ScottSusana CardosoIan BloorSusana EnríquezIan CarrSusana García-SilvaIan DanceSusana IbáñezIan FishSusana M. CardosoIan KerrSusana P. PereiraIan Kim B. TabiosSusana Santos BragaIan L RossSusann AuerIan M. RogersSusanna DolciIan M. SimsSusanna MolinariIan MusgraveSusanna PilusoIan PithaSusanne AufreiterIan R. CorbinSusanne GrässelIan RossSusanne HafnerIan TetlowSusanne KaeslerIanasi CatalinSusanne KimeswengerIbolya AndrásSusanne RospleszczIbolya FülöpSusete Nogueira FernandesIbrahim AbdellahSushant BangruIbrahim Ahmed ShaikhSushant K. KachhapIbrahim Al-AshkarSushanta GhoshalIbrahim AlfarrayehSushil DubeyIbrahim El-SayedSushil Kumar ShakyawarIbrahim F. RehanSushil MishraIbrahim M. SayedSushila SinghIbrahim SharifiSushma M. BhosleÍcaro Putinhon CarusoSusmita SilI-Chen PengSusumu KawakamiI-Chi LeeSusumu MinamisawaIchiro TakadaSusumu MitsuyamaIchwaku RastogiSusumu MuroyaIda CariatiSusumu OhyaIda FerrandinoSusumu SuzukiIda MannaSusumu TanakaIddo Z. Ben-DovSutherland MaciverIdit MaharshakSuvo ChatterjeeIdoia PostigoSuyong ReIdris LongSuzan CinarIdris YazganSuzana K. StrausIfat SherSuzanne PonikIghli Di BariSvatava KubickovaIgnacija VlašićSvein-Ole MikalsenIgnacio AlcaldeSven AlmerIgnacio Del CastilloSven Arke LangIgnacio Díez LópezSven BudikIgnacio Martin-GullónSven GöpelIgnacio Torres AlemanSven MånssonIgnatius Ryan AdriawanSven Marcel StefanIgnazio BlancoSven Müller-LoenniesIgnazio Gaspare VetranoSven OttoIgor A. FedorovSven RutkowskiIgor A. YakolevSvend Borup JensenIgor A. ZolotukhinSvenja Illien-JüngerIgor AdameykoSvetla TodinovaIgor BelyaevSvetlana A. DemyanenkoIgor Borisowitsch BuchwalowSvetlana A. LitvinovaIgor BuravlevSvetlana A. MurzinaIgor BuzalewiczSvetlana A. SelishchevaIgor ChesnokovSvetlana A. SorokinaIgor DeyevSvetlana AidagulovaIgor DeynekoSvetlana AntonyukIgor GolubSvetlana BatashevaIgor IvanovSvetlana BoronnikovaIgor JakovcevskiSvetlana BratskayaIgor JurakSvetlana DeryushevaIgor KarlovitsSvetlana DokudovskayaIgor KukhtevichSvetlana FaIgor L. DalingerSvetlana G. KozlovaIgor LukićSvetlana KonnovaIgor M. PaivaSvetlana M. ChernitsynaIgor Moiseyevich KvetnoySvetlana MarkovaIgor N. PavlovSvetlana R. JeremićIgor NabievSvetlana S. EfimovaIgor P. KaidashevSvetlana SarantsevaIgor PottosinSvetlana SimovaIgor PrudovskySvetlana SushkovaIgor ShmarakovSvetlana TamkovichIgor SlivacSvetlana V. GuryanovaIgor SplichalSvetlana V. KlinovaIgor TetkoSvetlana V. VeselovaIgor TurkiewiczSvetlozar VelizarovIgor Y. IskusnykhSvetoslav TodorovIgor YakovlevSvjetlana RausIgor Yu. DolmatovSvyatoslav GadomskyIgor ZhukovSwanand KoliIhsan UllahSwapan ChakrabartyI-Ju YehSwapan Kumar RoyI-Jung TsaiSwapna Priya RajarapuIkram Ullah KhanSwapnil Ganesh SanmukhIksoo HuhSwapnil SanmukhIkuo NakanishiSwarnalatha MoparthiIkuo NobuhisaSwarnali RoyIkuo SuzukiSwarup RoyIl Soo MoonSwarup Roy ChoudhuryIl Yup ChungSwastik PhuleraIlaria CicaliniSwati GargIlaria CosciSwati TyagiIlaria Dal PràSwayam PrakashIlaria Elena Lena PalamàSwayam Prakash SrivastavaIlaria FrassonSwee Keong YeapIlaria GrossiSwee Leong SingIlaria IacobucciSwee-Keong YeapIlaria MarechSwee-Suak KoIlaria MarroccoSweta B. PatelIlaria OttonelliSwetha SunkarIlaria PianoSy Duong-QuyIlaria PuotiSyama SanthakumarIlaria RoatoSyazwan SaidinIlaria TamagnoSybil CharrièreIlavenil SoundharrajanSybille HasseIldikó HorváthSyed A. A. RizviIldiko RaczSyed Ahmad Chan BukhariIldiko SzantoSyed Asad Hussain BukhariIleana C. FarcasanuSyed Azmal AliIleana CocanSyed Bilal HussainIleana Denisa NistorSyed Faraz AhmedIleana IeloSyed Farhan AhmadIleana MicleaSyed Haris OmarIleana Vera ReyesSyed Haroon KhalidIlian BadjakovSyed Husain Mustafa RizviIliana Medina-RamírezSyed J. KhundmiriIlias KounatidisSyed Najmul Hejaz AzmiIlias TravlosSyed Nasir Abbas BukhariIlija CvijetićSyed NazreenIliya PetrievSyed NurulainIliyan IvanovSyed QadriIlja VietorSyed Salman ShafqatIlker BayerSyed Sarim ImamIlkeun LeeSyed Sayeed AhmadIlkka PaateroSyeda Abida EjazIlknur Sur ErdemSyeda ShehzadiIllana GozesSygkliti-Henrietta PelidouIllimar AltosaarSylva PrerostovaIlma Korponay-SzabóSylvain BourgoinIlma NugrahaniSylvain DarnetIlona RissanenSylvain De BreyneIlona TrawczyńskaSylvain FraineauI-Lun HsinSylvain LegrandIlya B. BezprozvannySylvain RoyIlya G. ShenderovichSylvère StörmannIlya GordeychukSylvie AmuIlya KhodovSylvie BabajkoIlya KlabukovSylvie Bégin-ColinIlya KlyukinSylvie Ferrario-MéryIlya KorolkovSylvie Hauguel-De MouzonIlya M. TereninSylvie RimskyIlya TitovSylwester GawinkowskiIlya UlasovSylwester MazurekIlyas AlavSylwester SommerIlze ŠtrumfaSylwia BalutaImad KansauSylwia BudzynskaIman MirmazloumSylwia Dzięgielewska-GęsiakImari MimuraSylwia Męczyńska-WielgoszImen HaddoudiSylwia OkońImke KorfSylwia ProchowskaImmacolata PietraforteSylwia RóżańskaImmacolata SpecialeSylwia SiebielecImran MohamedSylwia SmolinskaImran TariqSylwia TerpilowskaImran UllahSylwia ŻołądekImre HolbSyn YeoImre LengyelSyrine HizemImre VassSzabolcs MuráthImrich BarákSzabolcs RudnóyImtaiyaz HassanSzczepan MogilskiImtiaj HasanSze Shin LowImtiaz KhalilSzénási TiborIn Jun YangSzőke LórántIn Koo HwangSzu-Chuan ShenIna JasutieneSzu-Yuan ChenIna SevicSzymon BocianIna VorbergSzymon JanczarInaam NakchbandiSzymon KubalaIn-Ah LeeSzymon Łukasz DraganIñaki LeteSzymon MalinowskiInbo HanSzymon SękowskiIndira ShrivastavaT. Dianne LangfordIndira SingaramT. J. HollingsworthIndra D. SahuT. P. KukinaIndranath GhosalT. Rinda SoongIndu G. RajapakshaT. T. Souza-ChiesIneke Van Der BurgtT. Taskın-TokInes Bilic-ĆurčićTa Yeong WuInés CanosaTabata Victoria Rosas DiazInes DrenjančevićTabosa José NildoInes KožuhTada-Aki KudoInês L. S. DelgadoTadahisa SugiuraInes MarticTadakazu KondoInês SimõesTadaomi FurutaInes VillanoTadashi NakamuraInese CakstinaTadashi SatohInga KwiecienTadashi WatanabeIngeborg KlaassenTadashi YoshidaIngeborg KlymiukTadayoshi BesshoIngo BauerTadayoshi KagiyaIngo RustenbeckTadeusz InglotIngrid Brust-MascherTadeusz IssatIngrid CipakovaTadeusz KaczorowskiIngrid DijkgraafTadeusz KarczIngrid FalnogaTadeusz KowalskiIngrid Fricke-GalindoTadeusz MalewskiIngrid HausserTae Hun ChungIngrid MartonTae Keun YooIngrid TonhajzerovaTae Kyung HyunIngrida BalnyteTae Sung JungInho ChoiTae-Eun ParkInhye ParkTae-Gyu LimIñigo Saiz-FernándezTae-Ho HamIn-Lu Amy LiuTae-Hong KangInmaculada Conejos-SánchezTae-Jin KimInmaculada López-IzquierdoTae-Ju ChoInmaculada SeguraTae-Kang KimInmaculada YruelaTaerin ChungInna ChabanTaesic LeeInna E. PopovaTae-Sung KimInna LavrikTaesup LeeInna P. GladyshevaTafadzwa KasekeInna Rabinovich-NikitinTaghred M. SaberInna SerganovaTaha Koray ŞahınInna SolyanikovaTaha MehanyInna SzékácsTahira PirzadaInna ZamulinaTahmineh MokhtariInnocence HarveyTai HatoInnokentii E. VishnyakovTaia Abd El-MageedIn-Sul HwangTaichi IshikawaIntan Rosalina SuhitoTaiho KambeIoan HutuTaiko ToIoan PeteanTai-Long PanIoana BoerasTaina T. NieminenIoana CabaTairo OshimaIoana Corina BocsanTaisia RolovaIoana DemetrescuTaisiya KozlovaIoana MaiorTaisiya SukhikhIoana MozosTaisuke TomonagaIoana StanculescuTai-Wei WuIoana-Mihaiela CiucaTaiyo ToribaIoanna KyriakouTaizo UdaIoannis A. GiantsisTaja RamutaIoannis A. TsakmakidisTakaaki AratakeIoannis A. VathiotisTakaaki HirotsuIoannis CharalampopoulosTakaaki SenbonmatsuIoannis D. LeonardosTakaaki ShimohataIoannis G. AviziotisTakafumi HamaokaIoannis GeorgiouTakafumi HaraIoannis GiantsisTakafumi ItohIoannis GrivasTakafumi KatoIoannis K. ToumpoulisTakafumi YokotaIoannis KanakisTakahiro AnzaiIoannis LiampasTakahiro InoueIoannis MorianosTakahiro KannoIoannis Ntanasis-StathopoulosTakahiro NagatakeIoannis PanagisTakahiro NemotoIoannis ParaskevaidisTakahiro UchidaIoannis PartheniadisTakahiro Watanabe-NakayamaIoannis RoussisTakahito OhshiroIoannis S. PaterasTakane Kikuchi-UedaIoannis SamarasTakanori YoshikawaIoannis TheologidisTakao HamamotoIoannis TsamesidisTakao IshikawaIoannis TsivintzelisTakao KanamoriIoannis VagelasTakao KimuraIoannis VamvakarisTakao ShimizuIoannis-Dimosthenis AdamakisTakaoki KasaharaIogann TolbatovTakaoki SaneyasuIolanda FrancoliniTakashi AkihiroIolanda Midea CuccoviaTakashi HimotoIolanda PopaTakashi KanbayashiIolanda Valentina PopaTakashi KokudoIole VendittiTakashi KudoIon BrinzaTakashi MatsumuraIon CristóbalTakashi MisawaIon MîndrilăTakashi MotomuraIon NedaTakashi NomizoIonel MangalagiuTakashi ObamaIonel SamfiraTakashi OkijiIonica MasgrasTakashi OnoderaIonut LuchianTakashi SaitoIonut TudoranceaTakashi TanikawaIonut-Cristian RaduTakashi TsunoIoscani Jimenez Del ValTakashi UebansoIosif MarincuTakashi YazawaIoulia SmonouTakatsune ShimizuIpsita MohantyTakaya HigakiIra JainTakaya MoriguchiIra S. WinerTakayasu KawasakiIraia Muñoa HoyosTakayoshi TagamiIrena BanTakayoshi UbukaIrena Baranowska-BociaskaTakayuki FuruishiIrena H. MaliszewskaTakayuki KatoIrena MajerzTakayuki KawatoIrena Małgorzata Duś-IlnickaTakayuki MasakiIrena NalepaTakayuki MizunoIrena RotermanTakayuki MurataIrena SmagaTakayuki NakagawaIrena WaleckaTakayuki NakagomiIrene Benito-CuestaTakayuki NittaIrene CarusoTakayuki OkamotoIrene Cobo-SimónTakefumi KimuraIrene CorteseTakeharu KanedaIrene Cortés-PérezTakehiko YokoboriIrene Cozar-CastellanoTakehiro SuzukiIrene CuadradoTakehiro TsukadaIrene FaenzaTakehisa NakanishiIrene Gómez-CruzTakeji Takamura-EnyaIrene GörzerTakemi OtsukiIrene Lara-SáezTakemichi FukasawaIrene M. VavasourTakenori HarunaIrene MadrigalTakenori SatomuraIrene RomeroTakeo MukaiIrene Sánchez-AjofrínTakeshi AibaIrene Santos-BarriopedroTakeshi HagioIrene SoderhallTakeshi HarayamaIrene ValasiTakeshi IrieIrene Vázquez-DomínguezTakeshi KikuchiIreneusz Bernard LitwinTakeshi SakamotoIreneusz GrubeckiTakeshi SatoIreneusz SlesakTakeshi SerizawaIreneusz SowaTakeshi TsumurayaIrfan A. RatherTakeshi YamamotoIrfan Ahmad BhatTakeshi ZendoIrfan KhanTaku KouroIria NeriTaku NakayamaIride MascherettiTaku TamuraIrin SirisoontornTaku YoshiyaIrina AnisimovaTakuhiro UtoIrina B. AlievaTakuichi SatoIrina BakloushinskayaTakuji OhyamaIrina BogolyubovaTakuji OkaIrina CazacuTakuji TanakaIrina D. KonstantinovaTakujiro HommaIrina DerkatchTakuma InagawaIrina DoduevaTakumi IshizukaIrina Draga CǎruntuTakumi NishiuchiIrina EliseevaTakumi TakeuchiIrina EsanuTakuomi HosakaIrina GladkikhTakuro NakagawaIrina Goldenkova-PavlovaTakuro ShirasuIrina GradinaruTakuto FujiiIrina HashmiTakuya HayashiIrina I. MohorianuTakuya KoieIrina IelciuTakuya OmoteharaIrina Isakova-SivakTakuya TadaIrina Iuliana CostacheTalib M. AlbyatiIrina KaygorodovaTalita ResendeIrina KondakovaTalma BrennerIrina LeonovaTamako HataIrina M. Le-DeygenTamal SadhukhanIrina Magdalena DumitruTamar Ben-YosefIrina MakarevitchTamar KrugmanIrina MateiTamar ListovskyIrina MirzaevaTamar SztalIrina NaletovaTamara BasovaIrina NesmelovaTamara BotoIrina Neta GostinTamara Fernández CabadaIrina PopescuTamara G. AmstislavskayaIrina PotorokoTamara GulicIrina ProninaTamara Hernández VerdejaIrina RusakovaTamara I. PajpanovaIrina SulaevaTamara K. NowlingIrina V. NesterovaTamara KingIrina V. PinchukTamara WeissIrina VasevaTamas BanyaszIrina-Georgeta SufaruTamas FeherIrini DoytchinovaTamás HegedűsIriny AyoubTamás HofmannIris F. KappersTamás JanákyIris Maria ForteTamás JuhászIris RibitschTamas KálaiIris Z. UrasTamás Kovács-ÖllerIrma ColomboTamás LakatosIrshad Ahmed HajamTamas LazarIrwin R. Donis-GonzalezTamás LetohaIryna A. KhasabovaTamás NagyIryna AntonyshynTamás OláhIryna BenilovaTamás OllmannIryna KovalchukTamás Röszer Habil.Iryna SamarskaTamás SzabóIsa SıdırTamer S KaoudIsaac Armendáriz-CastilloTana TandarićIsaac EliazTanawin NopsoponIsaac F. Céspedes-CamachoTangliang LiIsaac Hyeladi MalgwiTangui BarréIsaac Osei-BonsuTania Cecilia MihaiescuIsaac Yves Lopes MacêdoTania HenriquezIsaak RubinsteinTânia MartinsIsabel BravoTania PenchevaIsabel Cristina BoleliTania Quesada-LópezIsabel DuarteTânia Silvia FrödeIsabel Faria-RamosTânia Soares MartinsIsabel Fort GallifaTania ZagliaIsabel Fuentes-CalvoTanima DeIsabel GálvezTanja GerlzaIsabel LarreTanja Grubić KezeleIsabel LegazTanja J. MiličićIsabel M. CalhaTanja KosticIsabel MarquesTanmay KulkarniIsabel Muñoz-BarrosoTanmoy SanyalIsabel Pereira-CastroTanumoy MondolIsabel RomeroTanushree DangiIsabel Santos CardosoTanveer SinghIsabel Ten-DoménechTanveer TabishIsabel VeladaTanvi SinhaIsabela T. PereiraTanya StratevaIsabella DerlerTanzila SabaIsabella GrishkanTao HuangIsabella PanfoliTao LiIsabella ParoliniTao LinIsabella RimoldiTao LongIsabella RussoTao LuIsabella SaggioTao WangIsabella SalzerTao WuIsabella ZanellaTao XuIsabella ZironiTao YangIsabelle ChantretTao YaoIsabelle ChérelTao ZhangIsabelle LoganTapas MandalIsabelle Mus-VeteauTara McmorrowIsaiah PabuayonTara RobertsIsain ZapataTara SudhadeviIsao NagaokaTaranpreet KaurIsao OtsukaTaras L PanikorovskiiIsao UsuiTaras P. PasternakIsao YamaguchiTarcio Teodoro BragaIsela QuinteroTarek BaatiIsha SharmaTarek H. AfifiIshak ErtugrulTarek KapielIshaq KhanTarek S. IbrahimI-Shiang TzengTareq SalehIshida TakashiTariq MalikIshwarya SankaranarayananTariq MukhtarIsidoro FelicielloTariq PervaizIsidro AbreuTarique BagalkotIşın Yagmur CombaTarita BiverIskander IbrahimTaro NakamuraIslam HusainTaro ShimizuIsmael Martínez-CortésTarr TündeIsmael RodrigoTarryn WillmerIsmael Sánchez-GomarTarsila G. CastroIsmail AbdulazeezTarsis F. BrustIsmail Ben MosbahTarun Jairaj NarwaniIsmini E PapageorgiouTarun Kumar DuaIsopencu GabrielaTasleem ArifIsrael Carreira-BarralTasneem P. SharmaIsrael Pérez-TorresTaswar AhsanIsrael SeklerTat San LauIsrael VlodavskyTat’Yana KorshunovaIsrat AliTatiana A. KorolenkoIssa Sulaiman Al-AmriTatiana AndreaniIssan ZhangTatiana AstrelinaIstván A. KrizbaiTatiana BetakovaIstvan AltorjayTatiana DeminaIstván EgerszegiTatiana DomratchevaIstvan JanossyTatiana EliseevaIstván KissTatiana ErazoIstvan NagyTatiana GolubevaIstván PappTatiana I. ArefievaIstván PetákTatiana IlchibaevaIstván SzokodiTatiana LopatinaIsura NagahageTatiana M. VasilievaIta HadžisejdićTatiana MagdesievaI-Ta LeeTatiana MatveevaItai BenharTatiana MinkinaItalia BongarzoneTatiana N AbashinaItalia Di LiegroTatiana S. KalininaItamar GonçalvesTatiana S. PerovaIti SaraavTatiana ScorzaItziar ChurrucaTatiana V. IlchibaevaItziar Lamiquiz MoneoTatiana V. KomarovaItzik CooperTatiana V. ShelengaIuga MadalinaTatiana V. VygodinaIulia-Ioana Stanescu-SpinuTatiana Yu KuznetsovaIulian AntoniacTatiana ZolotoukhinaIulian Zoltan BoboescuTatijana ZemunikIuliana AproduTatjana DornIuliana MotrescuTatjana G. ShibaevaIuliana NenuTatjana RadosavljevićIuliana SpiridonTatjana SarcevIuliana StoicaTatjana SimićIuliia A. KorolevaTatsuaki TagamiIuliia NovoselovaTatsuhiko FurukawaIurii S. StafeevTatsuhiro SatoIva BarjaktarovićTatsuhito MatsuoIva KiracTatsuji MizukamiIva MozgovaTatsuo KandaIva PavlovićTatsuo KawaiIva SkrinjarTatsuo MiyamotoIva ŠkrinjarTatsuo SawadaIva SovadinováTatsuo ShimosawaIvaldo ItabaianaTatsuro HagiIvan A. BoldyrevTatsuro MutohIvan B. FilippenkovTatsuro YoneyamaIvan BarvikTatsuya AbéIvan BrandslundTatsuya KobayashiIvan BudimirTatsuya MorimotoIvan CampeottoTatsuya MoriyamaIvan Cruz-ChamorroTatsuya ToyokawaIvan DimovTatsuya UsuiIvan GiovanniniTatyana KolesnikovaIvan GitsovTatyana MerkulovaIvan HirschTatyana PasechnikIvan IngelbrechtTatyana ShabatinaIvan Ivanovich StoikovTatyana V. AbramovaIvan KreftTatyana V. SavchenkoIvan Krešimir SvetecTatyana V. VolkovaIvan KushkevychTatyana VetterIván M. MoyaTatyana VlaykovaIvan M. SavicTatyiana I. OdintsovaIvan MalčićTaufiq AhmadIvan MikheevTauqeer Hussain MallhiIvan NombelaTaxiarchis KatsinelosIvan PavlovićTe I. LiuIvan Pires De OliveiraTea GlontiIvan RogovskyiTecla CiociolaIvan S. Mfouo-TyngaTed WilsonIvan S. PetrushinTeerasak DamrongrungruangIvan S. RaginovTeet SeeneIvan S. ZhidkovTejas BarotIvan SalazarTejas BosamiaIvan ShorstkiiTejas KarhadkarIvan Shterev DonevTejeshwar RaoIvan SkvortsovTeka KhanIvan SrejovicTeległów AnetaIvan V. ZelepukinTeleky Bernadette-EmokeIván Valle RosadoTeng J. PengIvan VokřálTengteng YuIvana BilicTeo Jeon ShinIvana BjelobabaTeodor RusuIvana CaputoTeodora Alexa-StratulatIvana DjuricicTeodora MocanIvana DrvenicaTeodorico De Castro RamalhoIvana FridrichovaTeodoro BottiglieriIvana GobinTeodoro Coba De La PeñaIvana GrgićTeofana-Otilia Bizerea-MogaIvana HálováTeofil JesionowskiIvana IvicTerence L. MarshIvana KholováTerenzio CosioIvana LukićTeresa CarbonellIvana NovakovicTeresa CatalanoIvana PajčinTeresa CoutinhoIvana PanžićTeresa FemeníaIvana PeranTeresa G. D'AversaIvana ProdićTeresa Hazubska-PrzybyłIvana SamarzijaTeresa MougaIvana ŠkrlecTeresa PadroIvana Škugor RončevićTeresa PaínoIvana Sopek MerkašTeresa PinheiroIvana StojanovicTeresa PreglejIvana VaneckovaTeresa Rubio-TomásIvana ViolaTeresa RussoIvanka Habuš JerčićTeresa SemedoIvanka SpassovaTeresa SommaIvanka TsakovskaTeresa SummavielleIvar Von KügelgěnTeresa TrottaIvar ZekkerTeresa VinuesaIvayla DinchevaTeresa ŻołądekIvelina DessevaTereza PadrtováIvelina TsachevaTereza TicháIvelisse IrizarryTereza ToralováIves HaifigTerézia KiskováIvette Bañuelos-CabreraTerje RaudseppIvo HornTerje SvingenIvo M. FrancischettiTero A. H. JårvinenIvo StachivTerrance JohnsIvone MartinsTerrence DonohueIvonne Bazwinsky-WutschkeTerrence PivaIvonne OlmedoTerry D. HindsIvy A. W. HoTerry WheelerIwahashi NoriakiTerry-Elinor ReidIwona Cichowska-KopczyńskaTeruaki TozakiIwona CiereszkoTeruyoshi TanakaIwona GabrielTeruyuki MatsunagaIwona Grabowska-KowalikTeshome YehualaeshetIwona KarbownikTessa CarrauIwona KarwaciakTessa CromptonIwona KomanieckaTetsufumi TakahashiIwona Maria ZarnowskaTetsuhiro KajikawaIwona Misztalewska-TurkowiczTetsuo KoshizukaIwona MorkunasTetsuro TamakiIwona RybakowskaTetsuya HirataIwona RykowskaTetsuya HoriIwona RzeszutekTetsuya KawakitaIwona SzydłowskaTetsuyuki TakahashiIwona WertelTetyana NosenkoIyyakkannu SivanesanThais Fernanda De Campos Fraga Da SilvaIzabela DudaThaís Larré OliveiraIzabela JendrzejewskaThais MilessiIzabela Korona-GlowniakThaíse Gonçalves AraújoIzabela Korona-GłowniakThalia MedeirosIzabela Małysz - CymborskaThallapuranam Krishnaswamy Suresh KumarIzabela MatułaThanassis SpeliotisIzabela MichalusThangasamy SaminathanIzabela Nawrot-HadzikThangavelu RamanIzabela PawłowiczThangavelu S. RajanIzabela RaganThanh-Binh NguyenIzabela SabalaThanigaivelan KanagasabaiIzabela SzczerbalThanongsaksrikul JeeraphongIzabela ZakrockaThanos HalazonetisIzabella OlejniczakThasinas DissayabutraIzabella PawluczykThavasimuthu CitarasuIzabella RządThayne KowalskiIzabella Slezak-ProchazkaTheano LazaridouIzhar Hyder QaziTheerut LuangmonkongIztok PrislanTheis Skovsgaard ItenovJ. A. García-HorsmanTheivanayagam MaharajanJ. Adolfo García-SáinzThemistoklis GiannoulisJ. Calvin KouokamThemistoklis KabanosJ. De MouzonTheo HatzipetrosJ. Fielding HejtmancikTheocharis G. KonstantinidisJ. Francis BorgioTheodora FarmakiJ. G. ManjunathaTheodora PapamitsouJ. J. Van Der SmagtTheodora-Venera ApostolJ. Joe HullTheodoros AndroutsakosJ. Kelly SmithTheodoros EleftheriadisJ. L. RollandTheodoros KaralisJ. Michael SorrellTheodoros KarantanosJ. P. MansellTheodoros MavridisJ. RadnikTheofanis ChatzistamatiouJ. RodriguesTheoharis C. TheoharidesJ. Scott LeeTheoni MargaritopoulouJ. Tyson McdonaldTherese BeckerJaafaru Mohammed SaniThérèse CoudercJaap A. JolesThérèse MalliavinJace Jones-TabahThi Ha Châu TranJacek AntonkiewiczThi My Linh HoangJacek DrobnikThiago Almeida PereiraJacek E NyczThiago Barros-GalvãoJacek HoffmanThiago MaiaJacek JemielityThibault GallavardinJacek KamczycThibault ViennetJacek KiezunThibaut LégerJacek KuźmakThien An LeJacek L. KolanowskiThierno Madjou BahJacek LewandowiczThierry CensJacek NyczThierry ClaudelJacek RybkaThierry ClavelJacek SiódmiakThierry HauetJacek SkarżewskiThierry HeitzJacek TabarkiewiczThierry MagnaldoJacek ZebrowskiThierry PrangéJacinta SimmonsThierry RabilloudJack Bee ChookThierry TassaingJack Christopher LeoThilina U. JayawardenaJack H. Z. WuThilini WijesekeraJack LawlerThilo SpeckmannJackson AudleyThirumaran ThanabaluJackson Victor De AraujoThomas BalligandJackwee LimThomas BartnikasJacob B. LandisThomas BodenstineJacob CorcoranThomas BolandJacob FriedmanThomas BrogginiJacob GopasThomas C LamJacob LaugheryThomas CaspariJacob MashiahThomas CunyJacob VanderburghThomas DepaepeJacobo Hernández-MontelongoThomas DickmeisJacopo BurrelloThomas DippongJacopo Junio Valerio BrancaThomas DistlerJacopo MeldolesiThomas DuboisJacopo SabbatinelliThomas E. KuhlmanJacqueline C. MitchellThomas E. MürdterJacqueline CapeauThomas EllingtonJacqueline M. BentelThomas F MooreJacqueline ReinhardThomas F. GeorgeJacqueline T. BernardThomas FalguièresJacquelyn BowerThomas FinkJacques BarbetThomas FreimanJacques CabaretThomas GreitherJacques FantiniThomas GrewalJacques GilloteauxThomas HieronymusJacques IzopetThomas HollemannJacques J. TremblayThomas HorvathJacques PouyssegurThomas HubertJademilson C. SantosThomas J. BachJadwiga LaskaThomas J. OrtonJadwiga ManiewskaThomas John MartinJadwiga SołoduchoThomas KellyJadwiga WyszkowskaThomas KleinJadwiga ŻebrowskaThomas KlonischJae Boum YoumThomas KnopfelJae Hoon BahnThomas KrauseJae Hyuck ChangThomas L. MindtJae Min ChoThomas L. OlsonJae Seung KangThomas LiehrJae Sung LeeThomas LuxbacherJae Wook LeeThomas M. DurcanJae Yong KimThomas M. KüntzigerJaebong KimThomas MarshJae-Bong ParkThomas MüllerJae-Cheol PyunThomas N. SeyfriedJae-Gyu KimThomas NussbaumerJaeho HanThomas O KragJae-Ho LeeThomas OttoJaehong AhnThomas P. LozitoJae-Hwan KimThomas PatersonJae-Hyung ParkThomas Pierre LecompteJaekwon LeeThomas ProftJae-Min ChaThomas R. CaulfieldJaemin JeongThomas RuppertJae-Min OhThomas S. WeissJaeseok HanThomas SandersJae-Seon SoThomas SchmidtJae-Seong YangThomas SciorJaesung KimThomas SimonJae-Won ChoiThomas StocknerJae-Woong YuThomas SvobodaJaeyoung KimThomas TschernigJafar RezaieThomas Von ZglinickiJagathesh Chandra RajendranThomas VorherrJagdish JoshiThomas W. BeckerJagna Chmielowska-BąkThomas WaldhoerJagoda LitowczenkoThomas WeberJahad SoorniThomas WeiheJahan AlamThomas WhiteJahanban-Esfahlan RanaThomas ZoellerJahed AhmedThomas. O. KragJahidul Islam ShohagThong Ba NguyenJai P. RaiThong C. MaJaideep SandhuThorsten BraunJaidip JagtapThorsten KraskaJaime Arellanes-RobledoThorsten KucziusJaime CarrascoThorsten SeidelJaime CatalánThotten Elampilay SheejaJaime FornettiThu Hang NguyenJaime Moisés RomeroThuc-Huy DuongJaime RodríguezTiago Benedito Dos SantosJaime Rubio-MartinezTiago Campos PereiraJaime Santoyo-SalazarTiago NazarethJaime YáñezTiago Severo PeixeJakariaTian LiJake NeumannTian XiaJakir HossainTianbao ChenJakob MorgensternTianfei ZhouJakob NeefTianpeng ZhangJakob TroppmairTianxiang GaoJakub BarbaszTianyu LiJakub DuszczykTianyu YanJakub FamulskiTianyuan ChenJakub Kła̧CzTianyuan ZhangJakub KryczkaTiberiu Paul NeaguJakub RokTibor ErtlJakub RybkaTibor FülöpJakub RydzewskiTibor HortobágyiJakub SawickiTibor JandaJakub SuchodolskiTibor KrenácsJakub SzlękTibor NagyJakub SzperlikTibor PaliJakub VašekTibor ZellesJakub ZdartaTien-Yuan WuJalal TaneeraTifeng JiaoJamal NaderiTiffany BoucheryJames A. CarrollTiffany CookJames A. NiemanTigestu Alemu DesseJames AngelastroTihana DžombetaJames B. HurleyTihana Lenac RovisJames B. KotconTijana MilićevićJames BreretonTijana SubotičkiJames BunceTijana VučićJames C L ChowTilen ZamljenJames CaliTill BraunschweigJames ChowTilman BorggrefeJames CovingtonTilman BreiderhoffJames D. PatroneTim FosterJames EwenTim HuberJames FrelichowskiTim HulsenJames G HeckerTim MaischJames GimzewskiTim PrickettJames GroomeTim SandleJames HiltonTim SchubertJames HunterTim W. RattayJames J. MccormickTimo BursterJames Kumi-DiakaTimo GaberJames La ClairTimo SorsaJames M. GruschusTimofey RozhdestvenskyJames M. HarringtonTimothy D. VeenstraJames MacleanTimothy FlowersJames MorrisTimothy HughesJames NavaltaTimothy J. HibberdJames P. BasilionTimothy J. PetrosJames P. BennettTimothy JobeJames R. SellersTimothy John TranbargerJames S. MeabonTimothy KeifferJames SherwoodTimothy L. MegrawJames StewartTimothy M. ReynoldsJames TeeTimothy N. C WellsJames V. GruberTimothy NottoliJames V.M. HansonTimothy R. HooverJames VesenkaTimothy SimeoneJameson ForsterTimothy SnowdenJamie L. MankiewiczTimur Kh. FatkhudinovJamie O'RourkeTimur M. MirzoevJamioł-Milc DominikaTimur YorganJamir Pitton RissardoTin A. ThanJamshid TanhaTina B. MckayJan A. A. M. KampsTina BeyerJan A. SmalleTina Di PalmaJan AnderssonTina MckayJan BobekTina O'GradyJan BocianowskiTina SabelJan BrabekTina Tičinović KurirJan ChrastinaTing HanJan EggerTing LiJan FílaTing WangJan JežekTing ZhouJan JongbloedTing-Feng WuJan KazenwadelTing-Fung ChanJan KoltermannTinghao ChenJan KorbeckiTingjin ChenJan KormanecTingtao ChenJan KotekTingting ChenJan KucharskiTingting HongJan KueverTingting ShiJan L. MiljkovićTingting YangJan LakotaTingting YuJan LarocqueTingxin ZhangJan LehotskyTingyu ChangJán LehotskýTing-Yu ChengJán LeštákTing-Yu LinJan LukasTinhinane FaliJan MacutkevicTin-Tin Win-ShweJan NedergaardTiphaine MartinJan NovákTito BrambulloJan PaczesnyTitus SanduJan Patrick BoströmTiziana AlberioJan Philipp GötzeTiziana BachettiJan Philipp MenzelTiziana BorselloJan PodkowińskiTiziana CiarambinoJan RomantowskiTiziana De CristofaroJan RožancTiziana GuarnieriJan SmetanaTiziana M. SirangeloJan TesarikTiziana RaiaJan VagedesTiziana VenesioJan Von Der ThüsenTiziano MarzoJan WeberTobias HabisreutherJan YpermanTobias KrojerJan ZabrzynskiTobias LangeJan ŽenkaTobias MeitzelJan ZidekTobias SchraderJan ZivnyTobias StraubJana AntalíkováTodor DudevJana HedrichTofazzal IslamJana JanockovaTohru IkedaJana Jeevan RameneniTohru YanoJana MoravcikovaTojung TsengJana MrázováTolga SoykanJana OklešťkováTom EllisJana PiskTom HuxfordJana RieggerTom IrvingJana SedlaříkováTom L. BroderickJana Šic ŽlaburTom RyboltJana Sopková-De Oliveira SantosToma KeserJana StanicováTomabu AdjobimeyJana ŠtěpanovskáTomas BlancoJana StofilovaTomas BogielJana SvobodováTomas G. VillaJana TchekalarovaTomas NajerJana TimmTomás PinósJana ŽiarovskáTomáš PlachýJanaina FernandesTomas PospisilJanak JoshiTomas SimurdaJanaki IyerTomas SinkunasJanakiram R. VangalaTomas TorrobaJanani ParameswaranTomáš VyhnánekJanay Almeida Dos Santos SerejoTomaso VecchiJandeep SinghTomasz BlicharskiJane A. LeopoldTomasz BogielJane DeverTomasz CzapikJane E AnbinTomasz FrączykJane MurrayTomasz FrancuzJanet A. FooteTomasz FuchsJanet MatanguihanTomasz GambinJanet PiñeroTomasz GóralJanetta NiemannTomasz GrzywaJanez KoselTomasz GubicaJanez MavriTomasz JaneckiJan-Hendrik SchroederTomasz JanowskiJanice Aldrich-WrightTomasz JóźwiakJanice HuntingfordTomasz KloskowskiJanin HenkelTomasz KluzJanin Henkel-OberländerTomasz KockiJanina BahnemannTomasz KonopkaJanina MarkowskaTomasz KowalczykJan-Inge BjuneTomasz KrawczykJanire UrrutiaTomasz KubrakJanis A. MüllerTomasz KulikJanja StergarTomasz LitwinJanja TrčekTomasz M. KarpińskiJanka BábíčkováTomasz OszakoJanna M. AndronowskiTomasz PanczykJanna ShainskyTomasz PłoszajJanne KoivistoTomasz PorażkoJannik PrasuhnTomasz PowrózekJannis KörnerTomasz PrzygodzkiJan-Olof KarlssonTomasz RechcinskiJanos B. NagyTomasz RusakJános DégiTomasz SadkowskiJános FodorTomasz StompórJános KappelmayerTomasz SzczepańskiJanos MinarovitsTomasz SzymborskiJansons JurisTomasz TokarekJantana KeereetaweepTomasz UrbanowiczJanusz BlasiakTomasz W. KaminskiJanusz BłaszczykTomasz ZąbekJanusz NiedojadłoTomasz ZokJanusz SielskiTomi T. LiJanusz StrzeleckiTomislav KizivatJaques WaisbergTomislav MestrovicJared Adolf-BryfogleTomislav TostiJared FischerTomislav ŽivanovićJari IntraTommaso BocciJaris ValenciaTommaso BucciJarl BøgwaldTommaso ErcoliJarmila CelakovskaTommaso FelicettiJarogniew ŁuszczkiTommaso GrecoJaromír MyslivečekTommaso NeriJaromir PastorekTommy AlainJaromír VašíčekTomoaki MuranakaJaroslav FilipTomoaki TakataJaroslav PejchalTomofumi YamamotoJaroslav ŠebestíkTomoharu TakeuchiJaroslava LieskovskáTomohiko AoeJaroslaw CalkaTomohiko KomatsuJarosław CałkaTomohiro IshikawaJarosław CzyżTomohiro MizobataJarosław J. SakTomohisa BabaJaroslaw KrzywanskiTomohisa IshikawaJaroslaw PanekTomokazu FukudaJarosław PanekTomokazu HattoriJarosław PasekTomokazu KonishiJarosław PolańskiTomoki AbeJarosław PrzybyłTomoki HoshinoJaroslaw SerafinTomoki NakamuraJaroslaw SmiejaTomoki SatoJarosław WidelskiTomomi YamazakiJaroslawa RutkowskaTomonori MatsumotoJar-Yi HoTomoo ShibaJasbir UpadhyayaTomoya EsumiJasmin WenderleinTomoya FujieJasmina ĐuretićTomoya MaedaJasmina Lukinac ČačićTomoyuki KawaseJasmine SinhaTomoyuki MurakamiJasna Lenicek KrlezaTomoyuki NakamuraJasna MetovicTonali Blanco AyalaJasna Simonović RadosavljevićTonči RezićJason BeechTone AspevikJason Chia-Hsun HsiehTong ChenJason E. ComerTong WangJason GardinerTong WuJason PaxmanTongcui MaJason S. RadowskyTong-Hong WangJason T. C. TzenTonglu WeiJason T. TzenTongye ShenJason TannyTonino TrainiJason Thomas DuskeyTonny MuvhaliJason Yongsheng ChanTony BurgessJason YuenTope OyeladeJathish PonnuTorben RedmerJaume Alijotas-ReigTorben Sølbeck RasmussenJaume TorresTorbjörn LedinJavad AlizargarTorranis RuttanaphanJavad TavakoliTorsten Asselmeyer-MalugaJaved AhmadTorsten ChristJaved IqbalTorsten LowinJavier Alegre AbarrateguiTorsten MöhlmannJavier Fernández-RuizTorsten ThalheimJavier Francisco-MorcilloToru ItagakiJavier Garcia-PardoToru TaguchiJavier InserteToru TogawaJavier Manzano-LópezToru WakamatsuJavier Menéndez-MenéndezToru YoshitomiJavier Murciano-CallesToshiaki ArataJavier PeñaToshifumi AzumaJavier Redondo MuñozToshifumi TsukaharaJavier Saiz-PoseuToshiharu NagatsuJavier UserosToshiharu OnoderaJavier. CampaniniToshihiko TashimaJawahar MehtaToshihiko YamadaJaw-Yuan WangToshihiro KawaeJay AnandToshihiro NomuraJay H. ChungToshihiro ShimadaJay KrishnanToshihiro TsurudaJay PenneyToshikazu MorishitaJay SinghToshikazu SuzukiJay TrivediToshikuni SasaokaJay V PatankarToshimitsu YamaokaJaya AseervathamToshinungla AoJaya Prakash GollaToshio HattoriJayabalan NirmalToshio MorikawaJayachandran VenkatesanToshio TakahashiJayakumar NairToshio WatanabeJayalakshmi VallamkonduToshiro AraiJayanarayanan SadanandanToshitada YoshiharaJayanthi ParthasarathyToshitsugu FujitaJayapal Reddy MallareddyToshiya NakamuraJaycob D. WarfelToshiyuki KajiJayedul HassanToshiyuki KawaiJayeni Hiti BandaralageToshiyuki MatsuzakiJayesh DudhiaToshiyuki YamaneJayeshbhai ChaudhariTotan GaraiJayong ChungTou Cheu XiongJayshree SamantaToufic ElbeainoJayson W. JayTove AtlungJc LagunaToyoaki OhbuchiJe Hyun SeoToyohisa FujitaJe Hyung HwangTracey L WoodliefJean A. RondalTracey MadgettJean AmiralTracey NewmanJean BopassaTracy A. BrooksJean Carlos BettoniTracy A. WilliamJean Christophe GrisTracy CoelhoJean Christopher ChamcheuTracy Murray StewartJean E. AaronTraian DumitraşcuJean Jacques MonsuezTran Dang KhanhJean Luc E. DarlixTran Dang XuanJean Luc OlivierTran VinhJean Marie BillardTravis Kane JohnsonJean Marie RuysschaertTrevor ArcherJean Michel MaixentTrevor PitcherJean RodgersTriantafyllos DidangelosJean-Claude DesfontisTrias ThireouJean-Claude MartinouTrim LajqiJean-Daniel LalauTrine BlideJean-Denis TroadecTrinidad Montero-VilchezJeanette A. M. MaierTripti SharmaJean-François CôtéTrisha BansalJean-Francois GrossetTristan A. ReekieJean-François HernandezTristan DarlandJean-François PicimbonTrynda JustynaJean-François SubraTs. RadevaJean-Fred FontaineTsai-Te LuJeanine R. Jarnes-UtzTsan-Chi ChenJean-Louis DacheuxTsanka D. DikovaJean-Luc DavignonTsanko GechevJean-Luc MorelTse-Min LeeJean-Marc BarretTso-Ting LaiJean-Marie ExbrayatTsukasa OkiyonedaJean-Marie RuysschaertTsunehiko HiguchiJeanne Wilson-RawlsTsuneo IshidaJean-Paul LatgéTsuneo KatoJean-Philippe PinTsung-Chuan HoJean-Pierre GagnerTsung-Hsien ChenJean-Pierre Saint-JeannetTsung-Hsien LeeJean-René HamonTsung-Jen WangJean-Sébastien RougierTsung-Rong KuoJean-Xavier MazoitTsutomu InokuchiJean-Yves ChattonTsutomu KatayamaJean-Yves PiergaTsutomu MurakamiJebiti HaribabuTsutomu NakazawaJedeliza FerraterTsutomu ShimuraJee-Ching WangTsutomu TakahashiJeevithan ElangoTsuyoshi E. MaruyamaJeff SzerTsuyoshi KimuraJefferson ChenTsuyoshi OhtaJefferson Romáryo Duarte Da LuzTsuyoshi ShutoJefferson SantosTsuyoshi TakataJeffery S. TessemTsveta HristevaJeffrey A. KeenanTsvetelina BatsalovaJeffrey BraultTsvetelina VelikovaJeffrey BuchsbaumTsz Kin Martin TsuiJeffrey EckertTuan Hiep TranJeffrey G. DuckettTuba EsatbeyogluJeffrey HorowitzTuba GideJeffrey J. HsuTudor BorzaJeffrey J. HuangTudor Claudiu SpînuJeffrey K SchachterleTudor Lucian PopJeffrey L BennetzenTuerdimaimaiti AbudulaJeffrey LaurenceTuhin RoychowdhuryJeffrey LiptonTuire SalonurmiJeffrey MacdonaldTullio FlorioJeffrey SchlomTunde AlapiJeffrey SilberzweigTünde FeketeJeffrey V. LeytonTung-Sheng ChenJeffrey WigleTuo MengJeffry B. LansmanTuo ShaoJegan IyyathuraiTuo ShiJe-Hyun YoonTuomas LönnbergJekaterina ErenpreisaTuomas NäreojaJe-Keun RheeTursonjan TokayJelena AćimovićTushar ModiJelena Antić-StankovićTusty-Juan HsiehJelena BanTuuli KaambreJelena BožunovićTvrtko HudolinJelena GavrilovicTyphanie DumontetJelena IlićTyuterev VladimirJelena Korac PrlicTze-Kiong ErJelena MaksimovićTzi Bun NgJelena MarinkovićTzong-Horng LiouJelena Marković FilipovićTzong-Shyuan LeeJelena MartinovicTzong-Yuan WuJelena MijalkovićTzu Hsuan ChiangJelena Nesovic OstojicTzu-Cheng SungJelena SavićTzu-Cheng SungJelena T PetrovićTzung-Wen ChiouJelena TrajkovićTzu-Ping KoJelena VekicTzvetanka D. DinkovaJelica Grujić-MilanovićUdaiyappan JanakiramanJemaa EssemineUday BagaleJen-Chang YangUday Chand JhaJenchih TsengUday ChintapulaJen-Chih TsengUdishnu SanyalJenette CreaneyUdo JeschkeJenifer C. CroceUdo SchumacherJenifer Pendiuk GonçalvesUehara TomokoJenifer WohlersUģis CābulisJeniffer CarrilloUgo BastollaJennifer A. LemonUgo CarraroJennifer AltomonteUgo D'AmoraJennifer C. GibsonUgo Lionel MoensJennifer FangUgo TestaJennifer FazzariUgolino LiviJennifer HochscherfUgur CakilciogluJennifer J. JohnstonUğur ŞenJennifer LeeUiju ChoJennifer Martins NoroUjendra KumarJennifer MaynardUjjal BhawalJennifer MytychUjjal Kumar BhawalJennifer OrnelasUjjwal LayekJennifer PattersonUlf AndereggJennifer R. FlemingUlf LernerJenning TsaiUlf LindahlJenn-Ming YangUlf SchottJen-Pin ChuangUlises UrzúaJens André HammerlUlisses Moreno-CelisJens GeorgUllrich ScherfJens HarderUllrich WüllnerJens Von EinemUlrich Alfons MüllerJens-Ulrich RahfeldUlrich BaumannJenu Varghese ChackoUlrich HofmannJeong DongtakUlrich RassJeong Hee KimUlrich Severin DischingerJeong Hwan LeeUlrich VasconcelosJeong Jin BooUlrich WalterJeong Uk ChoiUlrike BacherJeong-An GimUlrike BaranyiJeong-Chae LeeUlrike Muscha SteckelingsJeong-Hae ChoiUlrike ReschJeongho HwangUlrikke VossJeongho KimUlyana ZubairovaJeong-Hoon LimUma K. AryalJeonghyun AhnUma S. SajjanJeong-Mo ChoiUmamahesh BalijapalliJeongsik YongUmberto GaragiolaJeppe PrætoriusUmberto LaforenzaJera JerucUmberto MalapelleJer-An LinUmberto TarantinoJere PaavolaUmesh GhoshdastiderJeremie D. OliverUmesh KalathiyaJérémie TopinUna RiekstiņaJeremie VitteÜner KolukisaogluJeremy ChienUpasana RayJeremy LagrangeUpendar GollaJeremy LeitzUpendra SinghJeremy TameUr Rehman NaveedJeremy WidemanUrban BrenJernej JakseUrmi RoyJernej JorgačevskiUrminder SinghJernej StareUrna KansakarJérôme CavailléUroš ČakarJerome DenisUroš GlavinićJerome DevyUroš M. GašićJérôme DuisitUrška Vrabič BrodnjakJerome E. TannerUrsula PanznerJerome LowensteinUrsula QuittererJerome MontnachUrsula StochajJerome P. FerranceUrsula WindbergerJérôme RobertUrsula WynekenJerome ThibonnetUrszula CytlakJérôme ZodiakisUrszula HohmannJerónimo Aragón VelaUrszula JankiewiczJeronimo AuzmendiUrszula Kalinowska LisJeronimo BravoUrszula LisieckaJerónimo González-BernalUrszula OleksiewiczJerry HeindelUrszula StachewiczJer-Yiing HoungUrszula UlkowskaJerzy AdamskiUrszula WachowskaJerzy BeltowskiUrszula WojdaJerzy BertrandtUrszula WydroJerzy ChojnackiUrtė BubnienėJerzy ChudekUsama Ramadan AbdelmohsenJerzy DropińskiUsman Khalid ChaudhryJerzy JuśkiewiczUsman ZulfiqarJe-Seung JeonUte RömlingJesse DeereUte WoehlbierJesse HayUte WölfleJessica A. CanterUtkarsh H. AcharyaJessica BlackburnUwe HansenJessica Costa-GudaUwe WolinaJessica CusatoUzma HaseenJessica D. Murillo-SaichV. Mohan Murali AcharyJessica Da Gama DuarteVaclav BabuskaJessica Dal ColVaclav PrajzlerJessica Finlay-SchultzVaclav VetvickaJessica HoffmanVadanasundari VedarethinamJessica IorioVadde RamuJessica K. AbbottVadim B. KrylovJessica LowethVadim ByvaltsevJessica StewartVadim ElaginJessica Z Kubicek-SutherlandVadim GaponenkoJessy A. SlotaVadim KhlestkinJessy EtienneVadim KlimontovJesús A. BallesterosVadim Le JoncourJesús Alberto Olivares-ReyesVadim LipinJesus BeltranVadim V. GenkelJesús Cosín-RogerVadim VasilyevJesus Davila-BarbozaVadim VolkovJesus Duque-AfonsoVadivel GanapathyJesús García-ColungaVahid FarzanehJesus Jiménez-BarberoVahid JahedJesus M. BertomeuVahid KheirollahiJesus M. Martin-CamposVahid RakhshanJesús Manuel De Los SantosVahid SerpooshanJesús María Gómez-SalineroVahideh RabaniJesús María López-ArrietaVaibhav A. DixitJesús María VielbaVaibhav UpadhyayJesus Martín-GilVaideesh ParasaramJesús MengualVaidotas StankevičiusJesus Munoz RojasVaishakh NairJesús PalomeroVaishnavi MuralikrishnanJesus PastorVakul MohantyJesús Puente-CórdovaValdir F. Veiga JuniorJesús Siquier CollValentin A. SemenovJesus V Jorrin-NovoValentin CoppolaJesús-María García-MartínezValentin LobyshevJetty Duffy-MatznerValentin RomanovskiJe-Wen LiouValentina AudritoJey-Jau LeeValentina BassareoJhaleh AmirianValentina BonettoJhy-Der ChenValentina BravatàJi Chang YouValentina CastelliJi Hoon JungValentina CerratoJi HuangValentina CossigaJi Hyeon AhnValentina Dell'OsteJi Hyeong BaekValentina Di FeliceJi Hyun KimValentina Di IorioJi LiValentina Di NisioJi WangValentina DincaJi ZhangValentina GiorgioJia (Joe) Long ZhuoValentina GrossiJia LiuValentina KovalevaJia Xian LawValentina LatinaJia XiongValentina LulliJiabing FanValentina MarassiJiacheng SunValentina MarchicaJia-Feng ChangValentina MassaJiahai ZhangValentina N. PolivtsevaJiahe LiValentina NaefJiahe WuValentina NikolićJiajia MaoValentina NotarstefanoJiajia YinValentina Oana BudaJiajun ShiValentina OpancinaJiajun ZhuValentina PallottiniJiale YangValentina PileczkiJialei DuanValentina PozziJiali LiuValentina RovelliJiali YuValentina ScattoliniJiali ZhaiValentina ŠpaničJia-Lin FanValentina V. ChebodaevaJialong WenValentina VasilevskayaJian LiValentina VassalloJian SangValentina VelleccoJian ShenValentyn OksenychJian ShiValeria BachioccoJian XuValeria ContiJian YeValeria De MatteisJian ZhangValeria De PasqualeJian ZhouValeria Di DatoJianan ZhaoValeria GiorgiJianbo LiValeria GriecoJianbo WangValeria LallaiJianbo YaoValeria LotitoJianbo YuValeria LucariniJianchao ZhangValeria LucchinoJianfeng HuaValeria ManganelliJianfeng HuangValeria Maria PintoJiangang ChenValeria MazziJiangbin WuValeria PanzettaJiangchuan ShenValeria PecceJiangjiang ZhuValeria PistorioJiangqi WenValeria PolzonettiJiangtao ChengValeria Rachela VillellaJianhao CaoValeria SogosJianhui QiuValeria SpagnuoloJianing XiValéria Stefania Lopes-CaitarJianing YuValeria TaurisanoJianjun WenValerian DormoyJiankai WeiValerie AmargerJiankun XuValerie BolivarJianling XieValerie CarpenterJianmei W. LeavenworthValérie ChoumetJianmin ChaiValerie CopiéJianmin SuValerie Diane ValerianoJianming KangValerie J. CarabettaJiann-Chu ChenValérie Jouan-HureauxJiann-Her LinValerie Le SageJiann-Ruey HongValerie PetegniefJianping ChenValerie TreyerJianqiang XuValerie WalkerJianwei ZhouValerio CaputoJianxiang XueValerio CicconeJianxun DingValerio FulciJianxun QiValerio LeoniJian-Zhang ChenValerio MaisJianzhou CuiValério Monteiro-NetoJianzhu LiuValerio PazienzaJiaqian LuoValeriu V. CoteaJiaquan YuValeriy LoktevJiawei WangValery E. TarabankoJiawei XiangValery PavskyJiawen YongValery ZinchenkoJiayi PeiValter LubranoJiayi WangVamshidhar Reddy VangoorJiayu LeongVan A. DozeJiayue QinVan Bon NguyenJiban ShresthaVan Eimeren ThiloJibin ZhangVan Giau VoJidi XuVan Khanh NguyenJie ChengVan Lun LowJie DongVan Thanh NguyenJie HeVan Thanh Tien NguyenJie HuVan Tu DuongJie JiangVan-An DuongJie LeeVance G. NielsenJie LiuVance Girard NielsenJie Miao HuVanderlei Salvador BagnatoJie SunVandermoere FranckJie WangVanesa CepasJie YangVanesa GalassiJie YeVanesa Martín FernándezJiekai YinVanessa GilJiemei WangVanessa Liévin-Le MoalJien Jiun ChenVânia GonçalvesJienyu DingVanja TadićJieqing LiuVarisa PongrakhananonJietang ZhaoVaromyalin TipmaneeJieyin ChenVarsha BhargavaJie-Ying LiangVarsha JainJiffin K. PauloseVartika SharmaJi-Fu WeiVartika TomarJih Ru HwuVarun KumarJihan XiaVarun MaturiJih-Huah WuVarun MongaJi-Hong LimVasco Sampaio-PintoJihoon NahVasia S. MavrogianniJihua ChenVasil AtanasovJihwan HaVasile PotopJiin Cherng YenVasile SimulescuJikui GuanVasile-Adrian SurduJilei ZhangVasileios G. PapatsirosJill A. JenkinsVasileios PapatsirosJillian H. HurstVasileios PapavasileiouJim BonhamVasileios ZiogasJim FouracreVasileiou PanagiotisJim ParkerVasilia FasoulaJimena MuciñoVasilica ȚucureanuJin BaiVasiliki LeventakiJin GaoVasiliki SyngounaJin JiaVasilios PergialiotisJin WangVasiliy ChernyshevJin XuVasily AleshinJin ZhangVasily AshapkinJin ZhongVasily KashtalapJinan LiVasily KlimovJin'An WangVasily YakovlevJinbin XuVassilia Ismini AlexakiJinbo YangVassilios PapadopoulosJinchao LouVassilios SikavitsasJin-Chul KimVassilios TriantafyllidisJin-Chung SinVassilis AthanasiadisJindong XieVassilis L. TzounakasJindrich CitekVassiliy TsytsarevJinesh PatelVasudeva IyerJinfeng LiaoVasudevan ManiJing CaoVasudevan RamachandranJing FangVeera Csr ChittepuJing GuoVeera Ganesh YerraJing LiVeera Manohara Reddy YenuguJing MaVeerupaxagouda PatilJing ShenVega Villar-SuárezJing TangVelia CassanoJing WangVelimir MladenovJing WeiVelmarini VasquezJing XuVenediktova NatalyaJing Yuan TanVenera I. TyukmaevaJing ZhangVenkat YedavalliJing ZhaoVenkata Naga L. R. SureJing ZhuangVenkata Rajesh YellaJingan LiVenkata Reddy ChirasaniJingang LiuVenkatachalam KarthikkumarJingbo PangVenkatakrishnan RengarajanJingen ZhuVenkataswarup TiriveedhiJingfeng HuangVenkatesan JayaprakashJinghua WangVenkatesh ChelvamJingjing GuoVenkatesh P. ThirumalaikumarJingjing WuVenkatesh YarraJingjing XuVenkateshwar MadkaJingrui ChenVenkateswara Reddy GogulamudiJingshu GuoVenkateswarlu KanamarlapudiJinguang HuangVenkatraman GaneshJingwei YuVenu RamanJing-Wen ShihVenus JoumaaJingxia LiuVera AlferovaJingya LiVera GusmanJingyi ChiVera KempJingyi LiVera S. BogdanovaJingyu FanVerena LabiJingyu ZhangVerena WallyJing-Yun WuVeria VacchianoJinhae KimVernadeth AlarconJin-Hee LimVernell WilliamsonJin-Ho KohVeronica AlcoleaJinhong MengVeronica Alexandra AntipovaJin-Hong ShinVerónica BerrielJinhu GuoVeronica BildJinli WangVerónica Chico GrasJinlian HuVeronica De PaolisJinming HanVeronica GregisJinn-Rung KuoVerónica Hurtado-CarneiroJinoh KimVeronica MarchanteJinpeng LiVeronica MarrellaJinrong MaVeronica MartiniJinsong ShenVerónica Moreno-ViedmaJin-Soo ParkVeronica Perez-De La CruzJinwei XieVeronica Sanda ChedeaJinwei ZhangVeronica SansoniJin-Won ParkVeronica SolovevaJinxiang XiVerónica TruccoJin-Yih LowVeronica WileyJinyong ZhangVeronik SomozaJinyu LiVeronika AleksandrovychJinzhi NiuVeronika BrezovakovaJiong LiVeronika HuntosovaJiong WangVeronika HuntošováJiongwei WangVeronika HyskovaJiranuwat SapudomVeronika KarcagiJiří CzernekVeronika KhairullinaJiří FajkusVeronika Kralj-IglicJiri FrimlVeronika PavliňákováJiri KassaVeronika VymetalkovaJiří PacherníkVéronique BlanquetJiřina BartůňkováVéronique De ContoJirko KühnischVéronique DelesalleJiro KikuchiVéronique KemmelJiro KishimotoVéronique StovenJiro TakitoVesa Cosmin MihaiJiru HanVeselin R. MaslakJishnu KrishnanVesna A. PerićJiten JaipuriaVesna JacevicJitendra KanshanaVesna MusaniJitendra KumarVesna PeršićJitendra Kumar SinhaVesna TepavčevićJitendra Kumar TripathiVesna Tumbas ŠaponjacJitendra MishraVesna V PanićJitendra TripathiVesna V. KojićJitesh KumarVesna Vranić MandušićJithine Jayakumar RajeswariVesna VucicJitka PracharovaVessela Tsakova-StanchevaJiuann-Huey Ivy LinVesta SteiblieneJiufeng WangVeymar Guadalupe Tacias-PascacioJiunn-Wang LiaoViacheslav NikolaevJiun-Wen GuoViatcheslav BykovJiwang ZhangViatcheslav MartemyanovJi-Won ParkVibhav GautamJiyang CaiVibhor MishraJi-Ye KeeVibor MilunovićJiye YanVicent ArbonaJiyeon HamVicente Calixto Zanón-MorenoJiyoung LeeVicente Faus-MatosesJizhong ChengVicente NotarioJo Anne StrattonVicente RamírezJo SourbronVicente RubioJoab ChapmanVicente Seco-RoviraJoachim ClementVicky SophianopoulouJoachim KrebsVictor A. ArrietaJoachim NeumannVictor GaultJoachim RassowVictor H. Pérez-LunaJoamin Gonzalez-GutierrezVictor HernandezJoan Carles Escolà-GilVictor I. BandJoan GilVictor I. TsetlinJoan JorgensenVictor Ivanovich SeledtsovJoan Marquez-MolinsVictor KrivenkovJoan OlivaVictor LemeshkoJoan Rosellò CatafauVictor M Bolanos-GarciaJoana BarbosaVíctor M. RiveraJoana CaetanoVíctor M. Sánchez-PedregalJoana Faria MarquesVictor M. VictorJoana GasparVictor Manuel MeseguerJoana GonçalvesVictor MartinJoana L. C. SousaVictor MatheuJoana MarquezVictor MultanenJoana MesquitaVictor NavaJoana RelatVictor PulgarJoana SilvaVíctor Quesada PérezJoan-Lluis Vives-CorronsVictor RizzoJoann S. RobertsVíctor Ruiz-Valdepeñas MontielJoanna Bajsa-HirschelVictor ThijssenJoanna Bartkowiak-WieczorekVictor V. TcherdyntsevJoanna BartosińskaVictor Van BeusechemJoanna BierłaVictoria Campos-PeñaJoanna BogusławskaVictoria E. González-RodríguezJoanna BoniorVictoria GrishkoJoanna BrzeskaVictoria K. BaxterJoanna CybińskaVictoria ManeuJoanna Czapla-MasztafiakVictoria P. ConnaughtonJoanna CzechowskaVictoria Ramírez GonzálezJoanna DąbrowskaVictoria RichmondJoanna Dagmara FedorowiczVictoria RoshchinaJoanna DipnallVictoria S. SarafianJoanna FedorowiczVictoria ShneyerJoanna FilipowskaVictoria VillegasJoanna FolwarcznaVictoriya A. GeorgiyantsJoanna GajewiakVida DemarinJoanna Gdula-ArgasińskaVida JuozaitienėJoanna Gromadzka-OstrowskaVida StegelJoanna GrzelczykVida StrasserJoanna KaminskaVidhya RaoJoanna KarbowniczekVidmantas BendokasJoanna Karbowska-BerentVidyalakshmi SethunathJoanna KazimierowiczViera KaraffovaJoanna KlepackaViera SchwarzbacherováJoanna KolmasViet-Khoa Tran-NguyenJoanna KruszewskaVignesh A. RathinasamyJoanna L. FoxVignesh DhandapaniJoanna LeszczyńskaVignesh MuthusamyJoanna M. WardlawVignesh SundararajanJoanna MoraczewskaVijai BhadauriaJoanna Pławińska-CzarnakVijay GahlautJoanna Popiolek-KaliszVijay KumarJoanna RaczkowskaVijay Kumar JidigamJoanna RossowskaVijay Kumar PatraJoanna RoszakVijay Kumar SiripuramJoanna RydzVijay PandeyJoanna RyszkowskaVijay ParasharJoanna SikoraVijay Rakesh Reddy SanikommuJoanna Śliwa-DominiakVijay Singh GondilJoanna SolichVijay TripathiJoanna StefanowiczVijaya VaikariJoanna StragierowiczVijayakumar MayakrishnanJoanna TimminsVijayakumar SukumaranJoanna WezgowiecVik MeadowsJoanna ZarzynskaVikas KumarJoanna Zawacka-PankauVikas MalikJoanna ZemłaVikas MalojiraoJoanne BoldisonVikas SaxenaJoanne Murphy-UllrichVikash KumarJoanne TobacmanVikash SinghJoanne Van RynVikkie MustadJoannis KallitsisVikram JoshiJoão Antônio Leal De MirandaVikram ThakurJoão Augusto FerreiraVikramjit RatheeJoão Azevedo-SilvaVikramjit S. BajwaJoão BassoVikrant RaiJoão BotelhoViktor DrganJoao DuarteViktor N. SerezhkinJoão Gama MarquesViktor PilepićJoão Gaspar-MarquesViktor ReshetnikovJoão Guilherme De Moraes PontesViktor VíglaskýJoão JúniorViktória HornokJoao LoboViktória KoroknaiJoão M. C. TeixeiraViktoria SarafianJoão MachadoViktória VengloveczJoão MartinsViktorie VlachovaJoão P. L. DaherViktoriya S. SidorenkoJoao Paulo LongoVilceu BordignonJoão Paulo Rodrigues MarquesVilhelmova-Ilieva NeliJoão PeçaVilko MandićJoao R GomesVilma Yuzbasiyan-GurkanJoao Ramalho-SantosVimal Kumar BalasubramanianJoão Ramalho-SantosVimalraj ManiJoão Renato SebastiãoVinal MenonJoão RodriguesVinay Kumar TyagiJoão SantosVinay MandatiJoão SilvaVinay NagrajJoao Werneck De CastroVinay RandhawaJoaquim C. G. Esteves Da SilvaVinayak JoshiJoaquim CarrerasVinayak KhodadeJoaquim Manuel Trigo MarquêsVinayak M JoshiJoaquim Miguel VieiraVinaykumar IdikudaJoaquim Murray Bustorff-SilvaVincent C. LombardiJoaquim PinheiroVincent C.O. NjarJoaquin BarrosoVincent HascallJoaquin De NavascuesVincent KellyJoaquin GadeaVincent Njung’E MichaelJoaquin Gomis-CebollaVincent P. DiegoJoaquín J. SalasVincent PaillèJoaquín María CamposVincent Picher-MartelJoar SvanvikVincent PonsJochen PölingVincent Quoc-Huy TrinhJody K. MaysVincent R. PantaloneJoe SongVincent ReyesJoe Truong NguyenVincent V. BekkerJoel EllwangerVincent WongJoel GraberVincent YeungJoel M. HarpVincente Javier Clemente-SuarezJoel S. GreenbergerVincenza BarresiJoëlle V. F. CoumansVincenza CrupiJoerg BurgstallerVincenza GragnanielloJoerg DurnerVincenzo ArizzaJoerg ReindersVincenzo BarnabaJohan HoebekeVincenzo CarcangiuJohan LedinVincenzo CondelloJohan O. PaulssonVincenzo CoppolaJohan SukweenadhiVincenzo FiorentinoJohanan Christian PrasanaVincenzo LaportaJohann GoutVincenzo NasilloJohann Helmut BrandstätterVincenzo NicolettiJohann SchinnerlVincenzo NobileJohanna CastañoVincenzo Ostilio PalmieriJohanna Lena TheruvathVincenzo ParrinoJohanna MoraczewskaVincenzo QuagliarielloJohanna P. LaakkonenVincenzo SummaJohanna WestraVincenzo TufarelliJohannes Francois FahrmannVincenzo VaianoJohannes FrankVineet KumarJohannes GräffVineeth M. VijayanJohannes HoferVineetha MukundanJohannes HojaVinesh VinayachandranJohannes JansenVinícius OsterneJohannes NossentVinicius RosaJóhannes ReynissonVinicius TraganteJohannes SchmidtVinit RajJohannes SchulzeVinit ShanbhagJohannes StadlmannVinita ChauhanJohannes StoecklVinita ChittoorJohannes StraussVinny NegiJohannes WachVinod JakkulaJohan-Owen De CraeneVinod K NarayanaJohari Yap AbdullahVinod KadamJohji KatoVinod KumarJohn A TayekVinod SureshJohn Albert RavenVinod VellorathekkaepadilJohn AshtonViola Maria PopovJohn B. VincentViola PrevitaliJohn Bertram VincentVioleta Calle-GuisadoJohn CarpenterVioleta G. TruscaJohn CrabbVioleta MakarevicieneJohn D. ChisholmVioleta MelinteJohn D. ClaytonVioleta PopoviciJohn D. PerryVioletta BorelliJohn DetermanVioletta KozikJohn DotisVioletta MacioszekJohn DzimianskiVioletta Opoka-WiniarskaJohn E. PintarViorel CircuJohn F. NewmanViorica Railean-PlugaruJohn G. HardyVipin Chandra KaliaJohn H. WhiteVirag VasJohn HulmeViraj IchhaporiaJohn J. NemunaitisVirender SinghJohn J. ParrishVirginia BrancatoJohn JosephVirginia BrighentiJohn K. JacksonVirginia CorazziJohn Kim ChoiVirginia Di PaoloJohn KoskinasVirginia Moreno-GarcíaJohn L ReaganVirginie JorisJohn L. HartmanVirginie NeirinckxJohn Lalith Charles RichardVirginie RappeneauJohn LamarVirginija JankauskaiteJohn M. PerryVirpi SiipolaJohn Mackay SøftelandVisarut BuranasudjaJohn MackrillVishal AhujaJohn ManionVishal ChavdaJohn MitrofanisVishal KhairnarJohn MooreVishal NegiJohn O. OnukwuforVishma Pratap SurJohn O. WarnerVishnu D. RajputJohn Patrick AlaoVishnu HosurJohn Paul DelanoVishnu RajputJohn PowersVishnu RevuriJohn PresleyVishnuprabu Durairaj PandianJohn RodgerViswanath DasJohn S. ChoyViswanathan NatarajanJohn S. MitchellViswas Raja SolomonJohn StanleyVít GloserJohn T. BatesVita GolubovskayaJohn TsiligkaridisVitalie LisnicJohn V. ForresterVitalii ZablotskiiJohn VakrosVitaliy G. ShevchenkoJohn W SteeleVitaliy GarginJohn W. NicholsonVitaly A. MorozovJohn W. ScottVitaly GurskyJohn WardVitaly KozinJohn WilliamsVitaly SineshchekovJohn WuVitaly TseluikinJohn XuVitaly V. KushnirovJohnatan Torres-TorresVito CalderoneJohnny D FigueroaVito GuarnieriJohnny PaduloVito TerlizziJohnson M. LiuVitolds MackevicsJohura AnsaryVitor CostaJoji OtakiVitor OliveiraJolán CsiszárVittoria BorgonettiJolanta B. ZawilskaVittoria CammisottoJolanta BatogVittoria ColottaJolanta Behnke-BorowczykVittoria D’EspositoJolanta DorszewskaVittoria Di MauroJolanta GroszykVittoria PerrottiJolanta Jaroszuk - ŚcisełVittorio AbbonanteJolanta Jaroszuk-ŚcisełVittorio BassoJolanta Krzysztoń-RussjanVittorio CalabreseJolanta KutkowskaVittorio ChecchiJolanta MierzejewskaVittorio FineschiJolanta RzymowskaVittorio GentileJolanta TarasiukVittorio SaponaroJolanta WawrzyniakVittorio ScardaciJolene ZhengVittorio UnferJon BeeverVivek A. BhadriJon HazeldineVivek AnandJon M. HuibregtseVivek DograJon P. WoodsVivek Hamse KameshwarJonah BeenstockVivek NarisettyJonas Amstrup FunderVivek PecheJonas CicenasVivek PuriJonas HauserVivek SaraswatJonas KolbenschlagVivian Capilla-GonzálezJonas Von HofstenVivian De WaardJonas ŽiaukaVivian Paraskevi DouglasJonasz Jeremiasz WeberViviana BarraJonatan MirandaViviana MarescaJonathan AriasViviana MeravigliaJonathan BarlowViviana OnofreiJonathan BenzaquenViviana VergaroJonathan BirdViviane Delghingaro-AugustoJonathan D. HumphriesVivien Coulson-ThomasJonathan K. WilliamsVivienne ReeveJonathan LecureuxVlad AzovJonathan M. BeckelVlad Constantin TofanJonathan M. CordeiroVlad GorduzaJonathan N. FlakVlad HerleaJonathan Puente-RiveraVlad PădureanuJonathan R. DimmockVlad SocoliucJonathan R. KellerVladan OndřejJonathan ReichnerVladan PopovićJonathan S. CalvertVladimir A. KorshunJonathan S. LindseyVladimir A. MitkevichJonathan SoboloffVladimir A. ZhukovJonathan WislerVladimir AleksandrovJonathon Edward MohlVladimir BabenkoJong Gug KimVladimir BilimJong Hee LeeVladimir BregadzeJong Hoon RyuVladimir BuchmanJong Myong ParkVladimir CherniavskihJong-Eun KimVladimir D. SkirdaJonghan KimVladimir DvoretskyJonghoe ByunVladimir DyakonovJong-Hwei S. PangVladimir I. ArkhipovJonghyun OhVladimir I. GridnevJongki ChoVladimir I. KalininJongkyeong ChungVladimir Igorevich MakarovJongmin KimVladimir IsachenkoJong-Sup BaeVladimir JurisicJong-Tae ParkVladimir KashoJong-Wha JungVladimir KenisJoni D. MottVladimír KryštofJoni H. YlostaloVladimir L. GabaiJoo Hyun OVladimir LazarevicJoo Shun TanVladimir LozanovskiJoo Young HuhVladimir LupashinJoohee JungVladimir NeplokhJooho ParkVladimir OslovskyJooman ParkVladimir P. BarannikovJoon Hyuk ChoiVladimir PacutaJoon W. ShimVladimir PalyulinJoonho JeonVladimir PisarevJoonhong ParkVladimir RiсhterJoon-Young JunVladimír ScholtzJoo-Youn ChoVladimir SerezhenkovJora ManassesVladimir SergeyevJordane PretoVladimir SobolevJorddy Neves CruzVladimir SukhorukovJordi Creus-MuncunillVladimir TesarJordi Pérez TurVladimir TodorovJordi Querol AudíVladimir UverskyJordi TamaritVladimir V. BezuglovJörg FuchsVladimir V. EgorovJörg KleeffVladimir V. KuznetsovJörg MarotzVladimir V. ZarubaevJörg Thomas BittenbringVladimir V. ZhivonitkoJorge A. Berlanga-AcostaVladimir VaksJorge A. LópezVladimir VladimirovJorge Alegre-CebolladaVladimir YussupovJorge AntunesVladimir ZhabinskiiJorge CervantesVladimir ZhemkovJorge De La RosaVladimirs PilipenkoJorge FeitoVladislav FomenkoJorge FuentealbaVladislav SemakJorge GonçalvesVladislav TomišićJorge Góngora-RodríguezVladislav VolarevicJorge Gonzalez-AguileraVladislava GusarJorge Gonzalez-GarciaVlad-Olimpiu ButiurcaJorge Gutiérrez-CuevasVlastimil KuldaJorge H. LeitãoVlatka LajnertJorge Hernández-VaraVlatko GalićJorge Hugo VillafañeVojislava BursićJorge L. Folch-MallolVola M. AndrianarijaonaJorge LopezVolker BrözelJorge LoraVolker M. LauschkeJorge M. CanhotoVolker NickeleitJorge M. CharcoVolkhard HelmsJorge Moreno CharcoVolodymyr ShvadchakJorge N.R. MartinsVolodymyyr KravetsJorge NietoVrinda GoteJorge PadrãoVrushali AgasheJorge R. Soliz-RuedaVrushank BhattJorge Serment-GuerreroVsevolod V. GurevichJorge SimonVuanghao LimJorge Verdú-AndrésVyacheslav DyachukJorge VieiraVyacheslav RyabovJörgen MölmannVyacheslav Sergeevich AnisimovJorlan FernandesW Peter VandertopJorma HinkulaW. Jean DoddsJørn Bolstad ChristensenW. M. KenkelJörns FickelW. Steven WardJorunn BörveWael AttJos Cornelius MieogWael HegazyJos H.T. RohlingWael ToukabriJos L. EerselsWael TraboulsiJos M.B.M. Van Der VossenWagdi SolimanJosanne VassalloWagner TenfenJosé A BarbutoWaheed AnwarJosé A. BritoWaheed RiazJosé A. CañasWai San CheangJose A. Cancelas PerezWajid Ali JatoiJosé A. GaviraWajid ZamanJosé A. LupiáñezWakako FujitaJosé A. MonrealWakako KawarazakiJose A. O. SimoesWakiro SatoJosé A. RianchoWaldemar GoldemanJose A. TapiaWaldemar KuligJosé AgudeloWaldemar TurskiJosé Alberto Choreño-ParraWaldemar WagnerJose Alegre-MartínWaldo CerpaJosé Ángel ArmengolWaleed Bakry SuleimanJose Angel FontenlaWaleed MareiJosé Antonio BárcenaWaleska C DornasJosé António BeloWalid AzabJose Antonio Lopez-EscamezWalid M. I. HassanJosé Antonio López-GuerreroWalid Mahmoud KhaliliaJosé Antonio Morales-GonzálezWalied AbdoJosé Antonio Paullada SalmerónWallace ThoresonJosé Antonio Picó-MonllorWalter AtwoodJosé Avendaño-OrtizWalter CabriJosé Bañuls-RocaWalter CanonJosé Bernardo Noronha MatosWalter CaseriJosé BlancoWalter F. RiesenJosé Bruno Nunes SilvaWalter G. BradleyJose C. CorchadoWalter HanelJose C. SouzaWalter L. StrohmaierJose Caparros-MartinWalter WahliJosé Carlos Bozelli, JrWalther BildJosé CastellWan NamkungJosé Correa-BasurtoWan-Chi TsaiJosé Cruz-ValderramaWanda LattanziJosé Daniel Aroca-AguilarWanda MączkaJosé Daniel CaminoWanda WadasJosé DieWang LeiJose Donato, Jr.Wang ZhengJosé Enrique Barquera-LozadaWanguo LiuJosé Enrique O'ConnorWan-Long ChuangJosé Enrique Torres VaamondeWanting NiuJosé Ernesto BelizarioWan-Wan LinJose Felix. Moruno ManchonWan-Yu TsaiJose Fernández-SáezWaqar ShafqatJosé Fernando FierroWaquar AnsariJose Garcia SanzWarren B NothnickJosé Gerardo González-GonzálezWarren TateJose Gomez-SanchezWaseem El-HuneidiJosé Jaime Martínez MagañaWasim A. DarJosé Javier Fernández CastroWasim H. ChowdhuryJose Javier LopezWasu Pathom-AreeJose Javier Miguel-HidalgoWasuke MoriJosé Joaquín GarcíaWataru MiyazakiJose L FusterWataru OtsuJosé L. AriasWayne Brian HunterJosé Luis Cabrera-PonceWayne CabralJosé Luis Castañeda-CabralWayne CarterJose Luis Marco-ContellesWayne CarverJosé Luis Montañez-SotoWayne GilesJosé Luis Pérez-CastrillónWayne LinJosé Luis ReyesWayne RobertsJosé Luis Rodríguez GutiérrezWee Yin KohJosé Luis SanzWei Boon YapJose Luis Valverde PiedraWei Chih LinJose Luis Villalpando-AguilarWei CuiJosé Luis ZugazaWei FeiJosé M. FradeWei Guang SeetohJose M. MuletWei GuoJose M. RojoWei HanJosé MancheñoWei LiJose Manuel AgeitosWei Long NgJose Manuel BreaWei NiJose Manuel Guevara-VelaWei QinJosé Manuel López GarciaWei Seong TohJosé Manuel Pérez-CañadillasWei ShaoJosé Manuel Rodríguez-PérezWei TongJosé Maria SantosWei WeiJosé María Santos-PereiraWei WenJose Maria ValpuestaWei Wen LimJosé Martínez-GonzálezWei WuJosé Martínez-HernándezWei XiaohongJosé Martín-NietoWei XuJosé Max Barbosa De Oliveira-JúniorWei YanJosé Miguel GuzmánWei YangJosé Miguel Seguí-RipollWei YuanJose Oñate-GarzónWei ZhangJosé Pedraza ChaverriWei ZhengJosé Peña-AmaroWei ZouJose PerdomoWei-Chi KuJose Perez-CastineiraWei-Chia LeeJose PietriWei-Chiao HuangJosé PinelaWei-Chieh LeeJosé R. PinedaWei-Chih ChenJosé Rafael De AlmeidaWei-Chun LinJosé Ramón González-PorrasWeichun WengJosé Ramos-VivasWei-Chyou WeiJosé Reyes-GasgaWei-Dong HeJosé Ricardo De Arruda MirandaWeidong WangJose Sanchez-AlcazarWeifang HanJosé SilvaWeifeng ZengJosé TeixeiraWeiguang ZengJose ThekkiniathWeihao YuanJose Trinidad . Ascencio-IbáñezWei-Hsiung YangJosé X. SoaresWei-Hsuan YuJosé YélamosWeihua DingJose-Antonio Mirón-CaneloWeihua TangJosé-Arturo Prada-OliveiraWeihua TianJoseba AlbizuriWei-Hwa LeeJosefa Isasi MarínWeijun QinJosefa SteinhauerWeikang MaJosefina CasasWeikun XiaoJosefina PonsWeilue HeJose-Maria CarazoWei-Lun HungJosep A. RosselloWei-Lun TsaiJosep GalceranWei-Min ChenJosep Marí AlexandreWeina KongJosep RomaWeiqian WangJoseph (Sifis) PapamatheakisWeiran ShanJoseph A. DiverdiWei-Sheng LinJoseph A. RocheWeisi LiuJoseph A. WestrichWeitao LiJoseph B. McpheeWei-Ting ChaoJoseph BarbiWeiwei ChenJoseph Calvin KouokamWeiwei HanJoseph ChoukrounWeiwei XueJoseph FurgalWeiwei ZhangJoseph GovanWei-Wen HuJoseph KannerWeiyan YinJoseph L. RobertsWei-Yi WuJoseph LovecchioWeiyu JiangJoseph M RutkowskiWeizhong WangJoseph M. ChalovichWellington V. CardosoJoseph Michael Ochieng' OduorWellison Jarles Da Silva DinizJoseph NassifWells UtembeJoseph P. HornakWen ChenJoseph PancrazioWen Hai ShaoJoseph PikkelWen JiangJoseph S. MerolaWen ShiJoseph W. LowdonWen Tsan ChangJoseph ZaiaWen WangJoseph ZiegelbauerWen ZhangJosephine AlbaWen-Bin WuJosephine FerreonWen-Bin YangJosh SharpWenbin ZhouJoshua A DublandWenbo ZhangJoshua A. BauerWenbo ZhiJoshua BarzilayWencheng LiJoshua EdwardsWen-Chi SuJoshua G. JacksonWen-Chin LeeJoshua KennedyWendel SilvestreJoshua LittleWen-Der WangJoshua M. AbbottWendy BollagJoshua M. HareWenfei WangJoshua R. BillerWen-Fu LaiJoshua T MorganWenheng ZhangJosiah OchiengWen-Hong LiJosip A. BorovacWenhua ZhangJosip BrnicWen-Hua ZhengJosipa BukićWenji PiaoJosipa KulesWenjie WangJosipa VlainićWenjuan ShiJosko BozicWen-Jun ShenJosu OrtegaWenjun WangJosue SznitmanWenjun XieJosy Anteveli OsajimaWen-Kuan HuangJoszef DudasWenli ChenJovana BradicWen-Li HsuJovana S. VukovićWen-Lii HuangJovanny GomezWenming ZhuJoy ArcherWenning WangJoy IrobiWenqiang FanJoy MitraWenqing JiangJoyce A. CartagenaWen-Sen LeeJoydeep BhaduryWen-Ta SuJože GrdadolnikWentao DongJozef KováčikWen-Tao SunJozef SowinskiWen-Wei ChangJozsef BallaWen-Wei SungJozsef DudasWenwei ZhengJózsef KalmárWenwen TangJózsef PetrikWen-Wu GuoJozsef SimonWenxin TongJózsef TímárWenyan XuJózsef TőzsérWenyi WeiJozsef VighWenying LuJózsef VirgaWen-Yu PanJózsef Z. FarkasWenyuan HanJu Hyun ParkWenzhen YuanJuan A. BernalWerner Giehl GlanznerJuan A. Fafián-LaboraWerner GlanznerJuan Adrián Ramos GuivarWerner KilbJuan AndresWerner ZwerschkeJuan Antonio DuránWeronika GorajJuan AyalaWesam S. ShehabJuan BarcenaWesley Luzetti FotoranJuan Bautista De SanctisWesley Lyeverton Correia RibeiroJuan BorreroWessam H. Abd-ElsalamJuan Bosco López-SáezWhady Armindo HuebJuan Carlos Alías GallegoWhitney A. BullockJuan Carlos AlonsoWiebke SchlörmannJuan Carlos Cuevas BernardinoWiesław BielasJuan Carlos De La Cruz-MárquezWiesław KacaJuan Carlos DominguezWieslaw SwietnickiJuan Carlos GardónWijnand J. C. Van Der VeldenJuan Carlos Hernández-GonzálezWiktor ZierkiewiczJuan Carlos Rodríguez-ManzanequeWilfred R. HagenJuan Carlos SaézWilfried A. KuesJuan CastilloWilfried Le GoffJuan David Ospina-VillaWilfried MeijerJuan DuWilhelm BlochJuan Francisco MartínWilhelm MistiaenJuan GómezWilliam A. CantaraJuan González-FernándezWilliam B. GrantJuan Ignacio VílchezWilliam Bart BryantJuan Jose AcevedoWilliam BlalockJuan José Galano-FrutosWilliam BrunoJuan José Hernández MoranteWilliam Bryan TerzaghiJuan José Martínez NicolasWilliam CamuJuan Jose Nogueira PerezWilliam CaudleJuan José Ramos-RodríguezWilliam DoerrlerJuan LiWilliam DuddyJuan Luis Delgado-GallegosWilliam H KonigsbergJuan Luis Garcia HernandezWilliam Judson HerveyJuan M. Fernández-CostaWilliam K. F. TseJuan M. GonzalezWilliam M. GwinnJuan M. Serrador PeiróWilliam M. TrumanJuan Manuel Gonzalez RosaWilliam Mark ErwinJuan Manuel López-AlcorochoWilliam Michael CaudleJuan Manuel PericasWilliam MoarJuan Maza-SolanoWilliam MorelloJuan Monribot-VillanuevaWilliam ParkerJuan Mozas-MorenoWilliam R. ReedJuan Pablo DamiánWilliam SchwanJuan Pablo NicolaWilliam T. HellerJuan Pablo RigalliWilliam TerzaghiJuan PasantesWilliam WilsonJuan Pedro FernándezWilliam WuJuan Pedro HolgadoWilliam Y. RaynorJuan Pedro Martinez-BarberaWilliam ZaragozaJuan PeragónWilliam ZergesJuan R. De Los ToyosWillian KenyonJuan Ramón TejedorWillie PeijnenburgJuan Rodriguez-VitaWilly BienvenutJuan RosadoWilly CarrasquelJuan SanabriaWilson CardonaJuan SanjuánWinda AriyaniJuan TorrasWindy McnerneyJuana Serrano LopezWinfried L. NeuhuberJuan-José R. CoqueWinfried M. K. AmoakuJude Juventus AweyaWing Kee LeeJudit DanisWing Shing HoJudit OláhWinglueng WongJudit OláhWioletta BielJudit SymmankWioletta PawlukowskaJudit TóthWioletta Rozpedek-KaminskaJudith DavieWirginia Kukula-KochJudith HagenbuchnerWirginia TomczakJudith Irina PagelWirongrong TongdeesoontornJudith Klein-SeetharamanWissem MnifJudith LukaszukWito RichterJudith MihalyWitold UhrynowskiJudith MihályWladimir Bocca Vieira De Rezende PintoJudith MontagWladyslaw KordanJudith NaamalaWładysław LasońJudith SchwartzbaumWladyslaw WeglarzJudith WaltersWłodzimierz BuchowiczJudyta Cielecka-PiontekWojciech BaranJuei-Tang ChengWojciech BialekJuhee AhnWojciech BielskiJui-Che LinWojciech FabianowskiJui-Cheng ChangWojciech GłuszewskiJuichih ChangWojciech JachećJuilee RegeWojciech JelskiJuin-Hong CherngWojciech KalasJui-Yang LaiWojciech KujawskiJui-Yen HuangWojciech MrozJujiao KuangWojciech PaslawskiJules Tianyu Zhang-YinWojciech PawlowskiJulia A. HorsfieldWojciech SmułekJulia Alvarez-MalmagroWojciech WakulińskiJulia CassaniWolfgang BergerJulia D. FineWolfgang EberhardtJulia DahlmannWolfgang EisenreichJulia FitzgeraldWolfgang FischerJulia Monjarás FeriaWolfgang GraierJulia RomanoWolfgang HolnthonerJulia Sanchez-CeinosWolfgang Michael BruecklJulia SchuelerWolfgang SattlerJulia ShanksWolfgang SchweigkoflerJulia V. NikolenkoWolfgang SiposJúlia Vallvé-JuanicoWolfgang SipplJulián AragonésWolfgang WeigandJulian EvansWolf-Michael WeberJulian Freen-Van HeerenWolfram AdlassnigJulian FriebelWolfram AntoninJulian SwierczynskiWolfram KunzJulián ValdésWolko ŁukaszJuliana CotabarrenWon Kyong ChoJuliana De Oliveira SilvaWonchung LimJuliana Degenhardt-GoldbachWoo Hyun PaikJuliana M. IvanovaWoojin KangJuliana Maria Navia-PelaezWoolfolk SandraJuliana Moura LunaWoong Sub ByunJuliane Daßler-PlenkerWoon-Man KungJulianna KardosWöstemeyer JohannesJulianne C. GriepenburgWouter KokJulie BonhommeWu Chih-LungJulie BrillWufei TangJulie ConstanzoWufu ZhuJulie DardareWuh-Liang HwuJulie DumonceauxWuyi MingJulie GrahamWytske Van WeerdenJulie GuignotXando Díaz-VillamarínJulie Hernandez-SalmeronXandra PereiroJulie Pires Da SilvaXanthopoulos KonstantinosJulie RageulXavier Bofill-De RosJulie SabaXavier Domingo-AlmenaraJulie TuckerXavier DuranJulien AncelXavier FigueroaJulien BarbierXavier FranchJulien DiharceXavier Gallart-PalauJulien FagetXavier GasullJulien FreitagXavier MontanéJulien Giron-MichelXavier Navarro-AcebesJulien GiustinianiXavier Sánchez-SáezJulien ParkXavier ThuruJulien PernierXavier Vidal-GómezJulien RossignolXenos PetridisJulien Saint-PolXesús FeásJulien SebagXi ChenJulien VignardXi YangJuliet Haarbauer-KrupaXi ZhaoJulieta AfonsoXiakun ChuJulieta BarchiesiXian GuoJuliette E. MoreauXian ZhangJuliette TinkerXiang GaoJuliette Van-DijkXiang LiJulija ErhardtXiang ShengJulijus BogomolovasXiang WangJulio Bastos ArrietaXiang ZhangJulio EscribanoXiang ZhaoJulio MoralesXiang ZhouJulio Plaza DíazXiangdong LiuJulio Ramirez-CastellanosXiangli ZhaoJulio Romero NogueraXiangnan LiJulio SoteloXiangpeng LiaoJulita KulbackaXiangping ChuJulius BogomolovasXiang-Qun HuJulius LiobikasXiangyu ZhangJulius RablXiangyu ZhaoJumpei SaitoXianhai ZhaoJun Cong GeXiansheng ZhangJun DangXianwen WangJun EguchiXiao HuJun FangXiao KuangJun HamazakiXiao LanJun HeXiao LinJun Hyoung ParkXiaobo DongJun ImaiXiaochen HeJun InoueXiaochuan ChenJun MaXiaodan DingJun Min SuhXiao-Dong ChenJun S. WeiXiaodong GeJun ShirakawaXiaodong WangJun Sung MoonXiaoen HuangJun Sung SeoXiaofan WeiJun UedaXiaofei LiJun WanXiaofeng HuangJun WatanabeXiaofeng LiuJun WengXiaoguang LiuJun WuXiaohe YangJun YangXiaohong LiuJun YoshinagaXiaohong WangJun ZhangXiaohong ZhouJun ZhouXiaohua YeJunaidi KhotibXiaohui ZhangJunaith S. MohamedXiaojie LiuJunfeng MaXiao-Jing WangJung Eun KimXiaojun (Gene) ShanJung Sook ChoXiaojun WeiJung Su RyuXiaokang DaiJung Woo LeeXiao-Kun OuyangJung Yong KimXiaolei TangJung-Heun HaXiaolei XuJung-Ho YoonXiaoli ZhangJung-Hoon KwonXiaoliang QiJung-Hyun ParkXiaoliang WangJung-Jae KoXiaolin FanJung-Jae LeeXiaolin TianJungoo JeeXiaolong YangJung-Suk SungXiaolu CambronneJunhao LiXiaolu WeiJunho ChungXiaoman ZhouJun-Hong LiuXiaoming LyuJun-Hui ChoiXiaoming XiaJunhui PengXiaonan ZhangJunichi HanaiXiaoqian ChangJun-Ichi HanaiXiaorong LiuJun-Ichi KadokawaXiaotang MaJun-Ichi SawadaXiaotu MaJunichi ShimizuXiao-Wen ChengJun'Ichi YoshidaXiaoxi MengJun-Jen LiuXiaoxiao HuangJunjie LiXiaoxing KouJunkai RenXiaoxiong WangJunki MaruyamaXiaoyan DuJunko AkadaXiaoyan WangJunlei ChangXiaoyang YiJunli ZhaoXiaoyong TongJunling ZhuangXiaoyong ZhangJunmei WangXiaoyu CuiJunmin HeXiaoyu LiJunnan FangXiaoyu SongJunn-Liang ChangXiaoyu WangJunping YuXiaoyu XieJunping ZhangXiaoyu ZhangJunqi HeXiaoyue ZhuJunran ZhangXiaxia DiJunsei MimuraXiaxiang ZhangJunseo OhXibi FangJunsheng LiXie BingJunsoo ParkXifei LiJunxi WuXiming YuanJun-Xia ZhangXin ChenJunyan JinXin CuiJunyan TaoXin GaoJunzi WuXin GeJu-Ock NamXin GuanJuozas LazutkaXin LiJu-Pi LiXin PanJuraj MajtanXin SunJuraj PiestanskyXin WangJurca TündeXin XingJure KnezXin ZhangJürg NüeschXin ZhaoJürgen BartelXin ZhouJürgen BehrensXinchao WangJürgen BriegerXinchen WuJurgen EngelberthXinda LiJürgen GeislerXing ChenJürgen KnauerXing LiuJürgen KreiterXing YangJürgen SchillerXing ZhangJurij TronteljXingang GuanJuris BurlakovsXinglu WangJuš KšelaXingping QinJu-Seop KangXingwang YuJustas ZilinskasXingyou WangJustin Claude Kemmegne-MbouguenXingyu ChenJustin DiangeloXingyu ShenJustin WilsonXingyu WuJustina Georgiana MotașXingzhou TianJustus G. GarwegXinhai YeJustyna AgierXinlei LiJustyna BonieckaXinlu DingJustyna BroniarczykXin-Luan WangJustyna DunajXinming SuJustyna FidlerXinxu YuanJustyna GilXinyang ZhaoJustyna HermanowiczXinye LiuJustyna Koc-JurczykXinyu WangJustyna KowalskaXinyue DongJustyna MaliszewskaXinzhong CaiJustyna Małyszek-TumidajewiczXinzhong ZhangJustyna MiłekXisha WangJustyna MiszczykXiu LiuJustyna Mozejko-CiesielskaXiufang GuoJustyna Niderla-BielińskaXiufeng YanJustyna NowakowskaXiujian LiuJustyna PaprockaXiujun ZhangJustyna StrycharzXiuli LiJustyna SzumiłoXiulian SunJuthaporn CowanXiumei HongJutiporn ThussagunpanitXiuxin LiuJutta EichlerXixi ZhouJutta Ludwig-MüllerXiyue SongJuulita KulbackaXosé Luis Pérez-FernándezJuxun WuXu Dong ZhangJu-Yang JungXu WangJu-Yi ChenXu ZhangJwakyung SungXuan FangJya-Wei ChengXuan LiJyh-Fei LiaoXuan XuJyh-Horng SheuXuan-Mei PiaoJyh-Jeng WuXudong HuangJyh-Yeuan LeeXue YongJyothi F NagajyothiXuebin QinJyothi PadiadpuXuechao LiJyoti Bala KaushalXuefei TanJyotirmoy DasXuehua XuK. G. PrasanthXuehui PangK. GulyaXuejie ChenK. H. Katie ChanXuejin ZhangK. KenryXuejun LiK. L. ChanXuelin ZhouK. R. AbhilashXuemei NiuK. S. Clifford ChaoXueming ZhangK. Thanigai ArulXuenan GuKa KimXueqing XuKa L. HongXueqing YangKa YangXuesong YangKaalak ReddyXuewen DengKaare GautvikXuexian YangKabirullah LutfyXuexiu ZhengKadir OzaltinXueyan LiKafait MalikXuezhao LiuKa-Hei, Kenneth LoXuezheng SongKai CaiXufeng ChenKai DongXujie ZhangKai GriebenowXuliang DengKai KammersXuliang ZhuangKai LiXuming MaoKai Min YangXun SongKai P. HoefigXuqiao ChenKai TaoY. Andi TrisyonoKai WangY. FujitaKai ZhangYaacov Ben-DavidKai ZhaoYachang ChouKai ZhengYachin CohenKai-Chiang YangYa-Ching ChouKaida AtsushiYacine BoulaftaliKaihui LuYadav S. BajagaiKai-Jun LuoYaewon ParkKaikai ZhuYafeng LiKailash SinghYafeng MaKailin TangYagmur Demircan YalcinKaining HuYahaya TajudeenKaio AleviYahia Z. HamadaKaio Cesar Chaboli AleviYahsin HsiaoKaisa HuhtinenYa-Hsuan ChangKaisa Leea SunelaYa-Hui ChouKaisa SunelaYahya SohrabiKaisu Rantakokko-JalavaYajie GuKaitlyn DykstraYajing HaoKaivalya MoluguYajuan SuKai-Wen HsuYajun XieKaiyang ZhangYalda JamshidiKai-Yin LoYalda Rahbar SaadatKajal KhodamoradiYali SunKajetan DabrowaYa-Ling HsuKalaivani ParamasivanYamaoka ToshimitsuKalathil K SureshkumarYameng LiKa-Leung WongYamini KrishnanKaliannan DurairajYan ChenKalichamy AlagarasuYan HongKalina AtanasovaYan JinKalipada PahanYan LiKaliyan BarathikannanYan WangKalliopi A. Roubelakis-AngelakisYan XuKalliopi StratigiYan YangKallol DasYan ZhouKallol PurkaitYan ZubavichusKalman ImreYana AnfinogenovaKálmán ImreYana CenKalpana RajaYana FeodorovaKalyan C. VinnakotaYana Roka-MoiiaKalyani ChaubeyYana ZavrosKamal A. M. Abo-ElyousrYanan LiuKamal Abo-ElyousrYa-Nan WangKamal AwadYanbo PanKamal ChowdhuryYanfang ZhaoKamal JnawaliYang BoguangKamal JoshiYang ChenKamal Kant SahuYang Jae KangKamal ShahYang JiangKamal SharmaYang LiKamalakar ChatlaYang LiuKamaldeep VirdiYang MeiKamalendra SinghYang QuKamariah IbrahimYang Ri-YaoKamel Abd-ElsalamYang SongKameyama ToshikiYang Wen-BinKamil GareevYang Yun-JungKamil GrubczakYang YushunKamil J. KuderYang ZhangKamil KaminskiYang ZhaoKamil KarolczakYang ZhouKamil KrawczynskiYanghsiang LinKamil RomanYangjin BaeKamila MyszkaYangpeiwei HuangKamila Rybczyńska-TkaczykYangrong CaoKamilla GrzywaczYangxin YuKamol DeyYangyiran XieKamrul HasanYangzhi ZhuKamyar KhoshnevisanYan-Hong CuiKamyar ZahediYanhong GuoKan SaitoYanhui HanKanae K. MiyakeYanis Toledano-MagañaKanakaraju ManupatiYan-Jye ShyongKandahalli Venkataranganayaka AbhilashaYanka KaramalakovaKandasamy SaravanakumarYanlin YuKandasamy SelvamYanmin WanKandi SridharYanming WangKangkang ChenYan-Ming XuKang-Mo KuYann GarciaKangxu WangYann S. GallotKankadeb MishraYannan YangKankan WangYannick PoitelonKannan ManiYannick RossezKannan Vrindavan ManianYannick ValléeKannappan ArunachalamYannis KalaidzidisKan-Sen ChouYannis KaramanosKanshi MinamitaniYanqi WuKaori UedaYanqiang LiKaoru YoshidaYan-Qing WangKaoutsar NasrallahYan-Ruide LiKapil D. PandeyYanshu ShiKapil D. PatelYan-Wen TanKara CervenyYanxian LinKaran AroraYanyun LiuKaran MalhotraYao JiangKaranovic DanijelaYao WangKaransher SandhuYao-Chang ChiangKarel AlcedoYao-Ching HungKarel AllegaertYaochun YuKarel KotaškaYaodong LiuKarel KrálovecYao-Feng ChangKarel SmetanaYaowu HeKarel TymlYaoxiang LiKarem A. CourtYaping WangKaren A. BoehmeYaqiu LinKaren BentleyYa-Qun ZhouKaren BlythYara M. MichelacciKaren BohmwaldYaron Shav-TalKaren E KempsellYaroslav AndreevKaren Harris-SchultzYasemin M. AkayKaren JohnstoneYaser GamallatKaren McleanYaser PeymanfarKaren SeiterYash GuptaKaren SkriverYashavantha VishweshwaraiahKaren SweazeaYasir HaleemKari KopraYasmina JuarranzKari L. StoneYasmine GhantousKari NaylorYasser HeakalKarim ZuhraYasser MajeedKarima DjabaliYasser S. MoursiKarima ZitouniYasser VasseghianKarin BoomarsYasufumi MurakamiKarin HufnaglYasuhiko KizukaKarin MalíčkováYasuhiko TabataKarin MartinYasuhiro KanataniKarin N WestlundYasuhiro KosugeKarina B. XavierYasuhiro KubotaKarina JouravlevaYasuhiro MatsunagaKarina Kubiak-OssowskaYasuhiro NishidaKarina LezirovitzYasuhiro TakenakaKarina VargovaYasuhisa KobayashiKarine GoussetYasuhito IshigakiKarine MichaudYasuka MatsunagaKariona GrabinskaYasuko FujitaKarl BunneyYasuko HonjoKarl DrlicaYasumasa OkazakiKarl G. WagnerYasunari MatsuzakaKarl Henrik GrinnemoYasunori YaoitaKarl J. SchreiberYasuo SudaKarl MillerYasuo TakeuchiKarl RobinsonYasuo TerauchiKarl SeydelYasuo WatanabeKarl Uwe PetersenYasuo YoshitomiKarl VandepoeleYasushi AdachiKarl-Heinrich LinkYasushi HiraokaKarlheinz PeterYasushi OkadaKarl-Josef DietzYasushi SakoKarmen StankovYasushi ShibataKarmveer SinghYasushi TsujiKarnika De SilvaYasushi YabukiKarol Alí Apaza AlccayhuamanYasushi YukawaKarol FijałkowskiYasushige ShinguKarol Kaziród-WolskiYasutaka KitagawaKarol SteckiewiczYasutaka KitahamaKarol SzubertYasuyuki IshikawaKarol WróblewskiYasuyuki S KidaKarola Vorauer-UhlYasuyuki TanahashiKarolina Anna Wojtunik-KuleszaYaswanth KuthatiKarolina ChilickaYatao RenKarolina GołąbekYatta BoakariKarolina Kaczor-UrbanowiczYavar VafaeeKarolina KotYayoi KobayashiKarolina KowalczykYa-Yun WangKarolina KowalskaYe LiuKarolina KulaYe MeiKarolina LabusYe PengKarolina M. StepienYe WangKarolina SłoczyńskaYee Kit TaiKarolina Szewczyk-GolecYee-Shan KuKarolina TkaczYegor E YegorovKaroly HebergerYegor VassetzkyKarsten GroteYehong ZhuoKarthick SubramanianYekbun AdiguzelKarthik H. ShankarYen Po WangKarthik KesirajuYen-Chien LeeKarthik SelvarajuYen-Chun PengKarthik TappaYen-Liang LiuKarthik VishwanathYen-Po ChenKarunakar Reddy PothulaYen-Wen LiuKarzova Maria M.Yeon Sun LeeKasandra BélangerYeongho KimKashif ShamimYeong-Min YooKashmir SinghYeong-Renn ChenKasi GopinathYeon-Ju LeeKasim KhanYerong ZhuKasper L. AndersenYesim Gokmen-PolarKassapa EllepolaYessica S. Tapia-GuerreroKasturi BanerjeeYeu SuKasturi PalYevgeniya IoffeKasturi RangannaYhiya AmenKata CsekoYi (Eva) WangKatalin BuriánYi FangKatalin MeszarosYi HeKatalin SchlettYi Hsuan OuKatanasaka YasufumiYi HuangKatarina BaralićYi Hui WuKatarina HančevićYi JinKatarina ItrićYi LuKatarina LukšićYi QiuKatarína OndreičkováYi ShanKatarína RažnáYi WangKatarina SebekovaYi XiaoKatarina StinglYi XuKatarína ValachováYi ZhaoKatarina VukojevicYi ZhuKatarzyna SzkudelskaYi ZuoKatarzyna A. PirógYibo QiuKatarzyna AugoffYi-Chen HsiehKatarzyna Bialik-WąsYichen LiKatarzyna Bober-MajnuszYi-Chen LiKatarzyna BulatYi-Cheng ChenKatarzyna Cieślik-BoczulaYi-Chou HouKatarzyna CurzytekYidong YuKatarzyna D. SluzalskaYifan BaoKatarzyna Donskow-ŁysoniewskaYifan MaKatarzyna Dorota RaczyńskaYifan ZhuKatarzyna GęcaYifei JinKatarzyna GieczewskaYi-Fen ChiangKatarzyna GolanYifeng QiKatarzyna GroborzYigal CohenKatarzyna GrochowskaYige HuangKatarzyna HolcmanYihan XiaKatarzyna J. BandyraYihenew Simegniew BirhanKatarzyna JanczakYiheng WangKatarzyna JaszczYi-Hsing ChenKatarzyna JelonekYi-Hsuan HsiaoKatarzyna KabalaYi-Huan LeeKatarzyna KaczyńskaYi-Hung ChiangKatarzyna Kiec-KononowiczYi-Jang LeeKatarzyna Kieć-KononowiczYi-Ju ChenKatarzyna KoziakYi-Lin ChiuKatarzyna KrawczykYilong WangKatarzyna KrukiewiczYimeng KongKatarzyna KurzatkowskaYimin HuangKatarzyna KuterYiming LiKatarzyna KwiatkowskaYiming WangKatarzyna LewandowskaYin LiKatarzyna ŁubiechYin Ping WongKatarzyna ŁudzikYin Quan TangKatarzyna LukasiukYing GaoKatarzyna M. BłażewskaYing HuangKatarzyna M. LisowskaYing LiuKatarzyna MikołajczykYing LuoKatarzyna Mleczko-SaneckaYing QingKatarzyna NucYing SuKatarzyna OtulakYing WuKatarzyna Otulak-KoziełYing YangKatarzyna Palińska-ŻarskaYing ZhangKatarzyna PalusYingang GuiKatarzyna Patrycja SzymańskaYingchun SuKatarzyna Pawińska-WąsikowskaYinghuai ZhuKatarzyna PobiegaYinghui LiKatarzyna PotrykusYingjun CuiKatarzyna RobaszkiewiczYingli GuKatarzyna RoszekYingmiao LiuKatarzyna RymuzaYing-Ming TsaiKatarzyna SobierajskaYingpeng HuaKatarzyna StachowiczYingqi ZhaoKatarzyna Sykłowska-BaranekYing-Shuo HsuKatarzyna SzewczykYingtao LiuKatarzyna Szot-KarpińskaYing-Tong DiKatarzyna TandeckaYingwei MaoKatarzyna TkaczYingxiang LiKatarzyna WalkiewiczYingying GuoKatarzyna WigluszYi-Ning ChenKatarzyna WińskaYining JinKatarzyna Winsz-SzczotkaYino WuKatarzyna Wojciechowska-DurczynskaYinyin BaoKatarzyna Wójcik-PszczołaYinyin WangKatarzyna Wolny-KoładkaYinying WangKatarzyna WozniakYi-Ping YangKatarzyna WróblewskaYiqun GanKatarzyna ZaorskaYi-Sheng ChengKatarzyna ZawadzkaYit Lung KhungKatarzyna ZiemnickaYi-Ting ChiangKatarzyna ZorenaYi-Tung ChenKate EarlYiwei QiangKate R. SecombeYi-Wen ChenKate WeeksYiwen ZhangKateel G. ShettyYixing DuKaterina AntoniouYixuan GuoKaterina ChlichliaYi-Yin DoKateřina DadákováYiyuan HanKaterina KambouriYizhou ZhangKaterina KatsaniYngve StenstrømKaterina KatsarakiYoana Kiselova-KanevaKaterina LaskariYoel OhayonKaterina NikolouliYogananda S MarkandeyaKateřina PetříčkováYogesh SrivastavaKaterina SpyridopoulouYogesh VikalKateřina VávrováYogitha N. SrikhantaKaterova Zornitsa IvanovaYohannes HagosKateryna FalYohei HayashiKateryna LystvanYohei IkezumiKatharigatta N VenugopalaYohei MiyanoiriKatharina DollYohei SekinoKatharina FrommYoichi KakutaKatharina HerkendellYoichi NabeshimaKatharina JähnYoichiro HoriiKatharina K. RallYoichiro OtakiKatharina KubatzkyYoko FukuharaKatharina KusejkoYoko HirataKatharina RumpYoko YagishitaKatharina SchallmoserYokthongwattana ChotikaKatharine CarterYoland C. AntillKatharine M. LodgeYolande RamosKatherine A. B. KellettYonathan GarfiasKatherine Amberson HajjarYong ChenKatherine CarvalhoYong Chool BooKatherine FantauzzoYong GuoKatherine KarakoulaYong Hwa CheongKatherine Mercedes HolleranYong JiangKatherine N. ThekenYong JinKathleen (Katie) DorrisYong Joo ParkKathleen HaleyYong LongKathleen Laura HefferonYong TengKathleen M. SakamotoYong WangKathleen RichardsonYong Weon YiKathleen WalterYong XieKathrin MädlerYong XuKathrin MarheinekeYong YangKathryn E. ColeYong ZhaoKathryn E. RoyseYong-Ai XiongKathryn L WotmanYong-Bin YanKathryn LeyvaYongbo BaoKathryn Y. BurgeYongbo XueKathy TaylorYongcai LiKati Juuti-UusitaloYongchao ZhangKatia BarbaroYongfeng LiuKatia GrilloneYonggang LuKatia LiburdiYong-Gu ChoKatia PaneYonghe MaKatia SparnacciYong-Hwan MoonKatie AldredYongji ZengKatja J. TeerdsYong-Jie XuKatja KalanYongjin ParkKatja Salomon JohansenYong-Jin ParkKatja SchefflerYongjun WeiKatja VasićYongkang YangKatja WitschasYong-Keun ChoiKatri LindforsYonglan LiuKatrien PoelaertYongliang ZhangKatrin BundkirchenYongming BaoKatrin CzogallaYong-Moon LeeKatrin Fischer-SchraderYong-Pil CheonKatrin JanikYongping BaoKatrin KoehlerYong-Qiang AnKatrin S. ReinersYongqiang ChenKatrin SchäferYongsheng LiKatrina TraberYongwen GuoKatsuhiko ArigaYongwoo JangKatsuhiko ItoYongxi LiangKatsuhiko MurakiYongyong YangKatsuki TsuchiyamaYongzhen LiuKatsumi IizukaYoni HaitinKatsunori NonogakiYoon Hae KwakKatsunori OkazakiYoon Young KimKatsunori YoshidaYoona KimKatsura TakanoYoong Kit LeongKatsutoshi YayamaYoonjung LeeKatsuya GomiYoon-Seok ChungKatsuyuki IchitaniYoo-Ri ChungKattathu MathewYordan MuhovskiKaumudi BhaweYordan YordanovKausar Sadia FakhruddinYorito HattoriKaushik GhoseYosef ShaulKaushik PandaYoshiaki HiranoKausik ChakrabartiYoshiaki KumagaiKavindra Kumar KesariYoshiaki MinakataKavita RawatYoshiaki SoejimaKavita SharmaYoshiaki TaniyamaKavitha S. RaoYoshiaki YuraKavya AnnuYoshiaki ZaizenKawaguchi NanakoYoshifimi TamuraKay Dietrich WagnerYoshifumi HamasakiKay OhlendieckYoshifumi ItohKay PerryYoshifumi SaishoKay WagnerYoshifumi TakahataKay-Dietrich WagnerYoshiharu FujiiKa-Yee Grace ChoiYoshiharu MitomaKazeem AdeyemiYoshihide HashimotoKazem NazariYoshihiko ItoKazi Lutful KabirYoshihiro EndoKazım KöseYoshihiro FukumotoKazimierz KuliczkowskiYoshihiro HayakawaKazimierz LatkaYoshihiro IkuraKazlauskas AndriusYoshihiro KinoKazuaki SyutsuboYoshihiro ShidojiKazuaki YoshimuneYoshihiro TaguchiKazuei IgarashiYoshihisa IkedaKazufumi TakanoYoshihito ShimaKazuhide OhtaYoshihito SudaKazuhiko HashimotoYoshikane YamauchiKazuhiko KibayashiYoshikazu KitanoKazuhiko KotaniYoshikazu OhyaKazuhiko MaedaYoshikazu UchidaKazuhiro IshibashiYoshiki KatayamaKazuhiro NishiyamaYoshimi NakagawaKazuhiro P. IzawaYoshimichi OkayamaKazuhiro TamuraYoshimitsu FujiiKazuhisa WatanabeYoshinari UeharaKazuhito AsanoYoshinobu KariyaKazuhito SuzukiYoshinobu OdakaKazuki KurimotoYoshinori InagakiKazuki YasudaYoshinori MikamiKazuko Kaneda-NakashimaYoshinori SatoKazumi HiranoYoshinori ShiraiKazumi NakabayashiYoshinori UtsumiKazumoto MurataYoshinosuke UsukiKazunori KawaguchiYoshiro SuzukiKazunori NagasakaYoshishiro HiramatsuKazuo ItoYoshitaka HishikawaKazuo KatohYoshitaka HosokawaKazuo KitamuraYoshitaka NakayamaKazuo KobayashiYoshiteru MorioKazuo YudohYoshiteru NoutoshiKazuomi SatoYoshitoshi KasuyaKazutaka KusunokiYoshiya OdaKazuto TajiriYoshiyuki NakamuraKazutoshi NishijimaYoshiyuki ShibukawaKazuya ToriumiYoshiyuki SueharaKazuya YamagataYosra A. HelmyKazuyoshi KuwanoYosra ToumiaKazuyuki DoiYosuke FukutaniKazuyuki KitataniYosuke MinamiKazuyuki MatsushitaYosuke OkamotoKazuyuki NakagomeYotaro OchiKe CaoYou LuKe LiYouakim SalibaKe MaYoudong MaoKe XiaoYouhua TanKe YangYoulian PanKe ZhangYoung Bok LeeKe-Cheng ChenYoung Eun YoonKecheng LeiYoung Han LeeKee Huong LaiYoung Ho KohKee Siang LimYoung Il JeongKeefe T. ChanYoung M. KwonKeehoon LeeYoung Whan ChoiKee-Lung ChangYoung Woo KimKeenan J. MintzYoung-Hag KohKeerthi MuruganYounghoon KimKees NoordamYoung-Hwa SoungKei GotoYoung-Il JeongKei IidaYoung-Ji ShiaoKei MaruyamaYoungjoo KwonKeigi FujiwaraYoung-Kwon SeoKeigo IkedaYoung-Kyo SeoKeiichi ItoYoung-Min YeKeiko HosohataYoung-Ok SonKeiko IwaisakoYoung-Sang AhnKeiko KashiwagiYoungsoo KimKeiko MiyoshiYoung-Su SeoKeila Acevedo-VillanuevaYoung-Su YiKeisuke HitachiYoun-Il ParkKeisuke IshizawaYoun-Jin ChoiKeisuke NakanoYounkee PaikKeisuke YanagisawaYouping WangKeita KaiYouqiang KeKeita TamuraYousef Al-EbiniKeitaro KanieYousef NaserzadehKeitaro SouYousif SubhiKeith Alan CrutcherYousry BayoumiKeith E. ShearwinYoussef KhamisKeith EriksonYoussef RouphaelKeith I TomlinsYouwen ZhangKeith MartinYouyoun MoonKeith RochfortYouzhong GuoKeittisak SuwanYouzhong LiuKeizo AnzaiYraima CordeiroKeizo TokuhiroYssel Mendoza MaríKelse Tibau De AlbuquerqueYu Aaron AnKelsy RobinsonYu AnKen AraiYu CaiKen KomatsuYu Chan ChangKen MillsYu ChenKen S. RosenthalYu H. SunKen ShiratoYu HuangKen TakashimaYu KoyamaKen TeterYu Kyoung KimKen ValenzanoYu LiuKendal HirschiYu PanKendrick Co ShihYu TangKeng Ioi VongYu Yuan ZhangKengo AzushimaYu ZhangKengo SatoYu ZhuKenichi GotoYu. V. IoniKenichi IkedaYuan ChengKen-Ichi KimuraYuan GaoKen-Ichi SaitohYuan HeKenichi TakayamaYuan HuKenichi YamadaYuan Hua WuKenichi YoshikawaYuan LiKen-Ichiro InoueYuan LiuKen-Ichiro MinatoYuan Xiang MengKenji FukuiYuan XueKenji FukunagaYuan ZhongKenji HayashidaYuanchun WangKenji IzumiYuancong XuKenji KanaoriYuanda SongKenji KitamuraYuangang LuKenji KojimaYuan-Ti LeeKenji NashimaYuanting XuKenji OhbaYuanwei ZhangKenji OkiYuanyan XiongKenji OsabeYuan-Yen ChangKenji YamadaYuanyuan ShiKenjiro BandowYub Raj NeupaneKenjiro OnoYubin MaKenkichi BabaYu-Chan ChangKenneth C. EhrlichYu-Che LinKenneth Francis RodriguesYu-Chen LoKenneth I. OnyedibeYuchen LongKenneth K. KiddYucheng XiaoKenneth R. HarkinYuchi LeeKenneth W. McmillinYu-Chia HsuKenshi WatanabeYu-Chieh ChenKensuke KumeYu-Chih WangKenta IwasakiYuchin LienKenta MasuiYu-Chung ChangKenta MoritaYu-Chung ChiangKentaro FukushimaYuda ChenKentaro OkamotoYu-De ChuKentaro YoshidaYudi RosandiKentaro YoshiokaYudong CaiKenza E. BenzeroualYudong ShenKenzo KoikeYue FengKepa Belloso-UribeYue HaoKeqiang WuYue ShengKeren LevanonYue WangKeren RabinowitzYue XingKermit L. CarrawayYue YangKerong ShiYueh ChoKerry McdonaldYuemin BianKerry SmithYueming JiangKersten ChristianYuen ClementKerstin JunkerYuening LiuKerstin MenckYueqi WangKeshlav Lall MaharjanYueqiao HuangKęstutis RimaitisYuequan ShenKeting ChenYuerou ZhangKeun-Sik KimYufeng GuoKeunsub LeeYufeng HuangKeun-Yeong JeongYufeng XiaoKevin BownYugo AshinoKe-Vin ChangYugo IshinoKevin D. PavelkoYugo KatoKevin GastonYuguang WangKevin HuynhYuguang ZhaoKevin KennaYuh BabaKevin LooiYuhan HaoKevin SchalinskieYuhao LiKevin Sheng-Kai MaYu-He YuanKevin TaburyYuhei GotoKevin WelchYu-Heng LaiKevin WillyYuhong GaoKeyang WuYuhong ZhengKeying ZhuYuh-Pyng SherKeyvan Asefpour VakilianYu-Hsiang KuanKfir SharabiYu-Huei LiuKhac-Minh ThaiYuichi HoriKhadija BanuYuichi NishiokaKhadija El HadriYuichi TadaKhadine HigginsYuichi TogashiKhairulmazmi AhmadYuichi TsuchiyaKhaled A ShaabanYuichiro HatanoKhaled AzizYuichiro J. SuzukiKhaled ElokelyYuichiro NishidaKhaled GiasinYuichiro YamashiroKhaled HabasYuji KabasawaKhaled R. AlkharsahYuji MikataKhaled SalemYuji ShimizuKhaled SaoudYuji TakedaKhaled TrabelsiYujia WangKhalid DoudinYujia XuKhalid ElsaafienYujiang FanKhalid ElwakeelYujiao HanKhalid F. AlmutairiYujie FuKhalid KarrouchiYujie SuKhalid MehmoodYu-Jin JungKhalid MuhammadYujiro NishiokaKhalid Rehman HakeemYu-Jung ChengKhalid UmarYuka TanakaKhalisanni KhalidYuki FukudaKhandoker SalemYuki IwasakiKhawar Sohail SiddiquiYuki KataokaKhemlal NirmalkarYuki MondenKhizar HayatYuki OhkawaKhosrow KashfiYuki Takechi-HarayaKhosrow RezvaniYuki YoshinoKhurram MunirYukihiko MomiyamaKhursheda ParvinYukihiro FurusawaKhurshid AhmadYukihiro WadaKhushdeep BandeshYukinori YabutaKhushwant Singh BhullarYukio FujiwaraKi Hyun NamYukio KuriharaKi Jun JeongYukitomo AraoKiarash Jamshidi GoharriziYuko HaradaKibeom KimYuko IwataKi-Cheong ParkYuko OhtaKid TörnquistYuko SatoKielan Darcy McalindenYuko T. HanbaKieran G. MeadeYüksel KorkmazKietsuda LuengwilaiYuksel PekerKieu The Loan TrinhYulema ValeroKieu Thi Xuan VoYulia DylevaKihak GwonYulia I. DeryabinaKi-Ho SonYulia K. KomlevaKihwan ChoiYulia M. AlexandrovskayaKijin KimYulia Yu StroylovaKi-Jun YoonYuliang WuKil-Soo KimYulii ShidlovskiiKim B. SeroogyYulin LiKim KampenYuling ShiKim M. Van VlietYuliya CherednichenkoKim S. MckimYuliya E. RyzhkovaKimberly JasmerYuliya I. RaginoKimberly MccallYuliya KorolkovaKimberly RowlandYuliya S. KrasikovaKimihiro YamashitaYulong RenKiminori OhtaYulong TangKimmo JensenYulong ZhongKinga BociongYuma FuseKinga GawelYu-Min HuangKinga GawlinskaYun HuangKinga GawlińskaYun Jin KimKinga K. TurzóYun LiKinga Kostrakiewicz-GierałtYun ShiKinga MolnárYun Suk HuhKinga MusiałYun Wah LamKinga TurzoYunchen YangKinjal MajumderYundi FengKira BehrensYunfeng LiuKira V. DerkachYunfeng ZhaoKira V. VyatkinaYung Hsiang ChenKiramage ChathurangaYung-An TsouKiran KunduYung-Chieh ChangKiran PanickarYung-Chieh LinKiran SandhuYung-Chih ChenKiran SapkotaYung-Hao TsouKiriaki ThermouYung-Luen YuKiril BahcevandzievYung-Ray HsuKirill LarionovYung-Tsu ChoKirill M. SkupovYunhui GeKirill TkachenkoYu-Ning TengKirill V. PeskovYunjo SohKirill Vitalievich AzarinYunli ZhouKirk GastonYun-Ming WangKirk HabeggerYunn-Hwa MaKirsi M. IsoherranenYunping LimKirsti YtrehusYunseok HeoKirstine Nyvold Bojsen-MøllerYun-Shen ChanKirsty J. McleanYunsong LiuKirsty JensenYuntao HuKirthana Kunikullaya UYunting PuKirtikar ShuklaYunus AlapanKirtikumar Ramesh KondhareYunwen TaoKirtiman Deo MalviyaYunxiang MuKiruphagaran ThangarajuYunxiao ZhangKishore CholkarYupeng LiKishu RanjanYuqi ChenKishwor PoudelYuqian LiuKit Leong CheongYuqing ZhangKi-Tae HaYuri A. VorotnikovKitman TsangYuri GogolevKi-Yeon YooYuri KobozievKiyofumi TakabatakeYuri KorystovKiyohide IshihataYuri Lvovich OrlovKiyong NaYuri MotorinKiyonori KawasakiYuri NesmelovKiyoshi AsadaYuri TokarevKiyoshi TakagiYuri TrusovKiyosumi HoriYurii KalyuzhnyiKiyotaka HitomiYurii KistenevKiyotaka ShibaYurii S. BorovikovKiyotaka TokurakuYuriy AlekseyevKiyotaka UchiyamaYuriy F. ZuevKiyotake IshikawaYuriy G. DenisenkoKjell Hansson MildYuriy IlinskiyKjetil AndressenYuriy Lvovich OrlovKlára CsekeYuriy MalyarKlára KosováYuriy OrlovKlaudia BanachYuriy PankratovKlaudia BarabásYuriy ShulgaKlaudia DopytalskaYurong LiangKlaudia KocurekYury LadilovKlaus BrusgaardYury LoikaKlaus CichutekYury P. RubtsovKlaus FortscheggerYury ZhernovKlaus GaseYusen ZhouKlaus GeisslerYusheng LiangKlaus H. HoffmannYushu GeKlaus HantkeYushun ZengKlaus HolzmannYusof KamisahKlaus Peter LattéYusuf SertKlaus PetryYusuke EchigoyaKlaus ReuterYusuke KatoKlaus WandeltYusuke MatsunoKlaus-Dieter SchlüterYusuke MurakamiKlaus-Peter GötzYusuke MurataKlemen BohincYusuke NishimuraKo FujimoriYusuke OjiKo KoYusuke OkumaKo OkumuraYusuke YamamotoKobayashi DaisukeYuta TanizakiKobayashi NatsukoYuta TsujiKodjovi D. MlagaYuta YoshizakiKoel ChaudhuryYutaka ItokazuKoen BartholomeeusenYutaka KurodaKoen VandenbroeckYutaka MatsudaKogiku ShibaYutaka OhsedoKoh SugamataYutaka TamaruKohei HommaYutian MaKohei OhtaYuting YuanKohei OkuyamaYuushou NakayamaKohei TorikaiYuval ShagamKohei UchimuraYuvaraj M. HungeKohei YuyamaYu-Wei FangKohji MoriishiYuya KumagaiKohji TakeiYuya NagasawaKoichi FujisawaYuyan HanKoichi KawadaYuyan JiangKoichi TakakiYuyoung JooKoichiro ShiomoriYuyue ZhongKoji MikamiYuzuru TozawaKoji MiyamotoYvan VandenplasKoji MuraiYves BorbélyKoji OohoraYves GouriouKoji OtaniYves MenezoKok Hian TanYves RoussignéKok Siong ChenZacharoula IatridiKoki KosamiZachary BurtonKoki MakabeZachary F. BurtonKolandaswamy AnbazhaganZachary S. ClaytonKoldo Garcia-EtxebarriaZachary SmithKo-Lin KuoZachery OestreicherKollur Shiva PrasadZackary FitzsimondsKomal KalaniZafar IqbalKomal RainaZahra AshkavandKomal RamaniZahra HemmatKomivi DossaZaid YounesKomuraiah MyakalaZaigang ZhouKomwit SurachatZając MirosławKonda KumaraswamiZakhar O. ShenkarevKonda Mani SaravananZakia AkterKondababu KurakulaZala LuznikKondalarao BankapalliZalba SaraKongju ZhuZanda BakaevaKönig ChristianŻaneta Kimber-TrojnarKonlin ShenZaneta SwiatkowskaKonrad A. SzychowskiŽanka Bojić-TrbojevićKonrad CelińskiZarezade VahidKonrad HuppiZarrin BasharatKonrad Kamil HozyaszZasha WeinbergKonrad StępieńZaza KhuchuaKonrad SzychowskiZbârcea Cristina ElenaKonstantin A. LukyanovZbigniew BrzozkaKonstantin A. ProsolovZbigniew GarncarekKonstantin BelosludtsevZbigniew J. WitczakKonstantin ChekanovZbigniew MiszalskiKonstantin I. GalkinZbigniew RaszewskiKonstantin Johannes ScholzZbigniew RybakKonstantin KozlovZbigniew SrokaKonstantin LukyanovZbigniew ZającKonstantin MikhelsonZdenek HodnyKonstantin N. YaryginZdenék KejíkKonstantin V. KiselevZdeněk KejíkKonstantin V. KudryavtsevZdeněk TůmaKonstantin V. MoiseenkoZdenek WimmerKonstantin VolchoZdeňka BendováKonstantina PanoutsopoulouZdzisław LatajkaKonstantina StathopoulouZeenat FarooqKonstantina TsigkouZeeshan BandayKonstantinos C. MakrisZeeshan HamidKonstantinos DimasZehra EdisKonstantinos DimopoulosZehuan HuangKonstantinos DimosZehuan LiaoKonstantinos EvangelouZélia SilvaKonstantinos GkirkasŽeljana FredotovićKonstantinos GkrintzalisZeljka LoncaricKonstantinos KoudounasZeljka PetrovicKonstantinos NtetsikasŽeljka TrumbićKonstantinos S. MylonasŽeljka Večerić-HalerKonstantinos SousounisŽeljko AndabakaKookhui RyuŽeljko AntićKorah Pushpamangalam KuruvillaŽeljko JukićKoraljka Gall TroseljŽeljko M SvedružićKoraljka Gall-TrošeljŽeljko PetrovskiKoraljka HusnjakZeljko SundovKorbinian BrandZengcai LiuKoren ArielZengqian ShiKornanong YuenyongchaiwatZengyou HeKornél KirályZening WangKornelius KerlZenon CzubaKosar Hama AzizZerrin OnadimKosmas HaralampidisZetian YangKosta PopovićZeyan LiKostantin ChegaevZeynep Alpay SavasanKostas BethanisZhan ShuKostiantyn TorokhtiiZhang LiKosuke AritakeZhangjiang LiuKosuke InoZhanguo XinKosuke NishiZhanhui ZhangKotapati Kasi ViswanathZhanjun GuoKoteswara Yerra RaoZhanquan ZhangKoteswararao GarikapatiZhanyou XuKouhei YamadaZhao ChuntaoKouichi YoshinariZhao DingKouji MiyazakiZhao JingKoyal GargZhao LvKozo NakamuraZhao ZhangKranow CharlesZhaohan LiKranthiraja KakaraparthiZhaohui ChuKranti A. MapuskarZhaoming QiKrekwit ShinlapawittayatornZhaoqian TengKresimir DolicZhaoqin WangKrijt JanZhaoxi SunKrinio GiannikouZhaoxiong WanKrish ChandrasekaranZhaoyang DingKrishan K. VermaZhaoyuan LyuKrishan RaiZhe JinKrishna BhattaraiZhe KanKrishna ChaitanyaZhe LiKrishna Chaitanya SuddalaZhe YangKrishna KantZhen ChengKrishna Kishore UmapathiZhen DaiKrishna Mohan Padmanabha DasZhen LuoKrishna Mohan SurapaneniZhen YuanKrishna PanthiZhen ZhangKrishna PatelZhenbo HanKrishna SepuruZhenbo TuKrishna SharmaZheng Zachory WeiKrishnaji R. KulkarniZhenghe WangKrishnanand P. KulkarniZhenglin GuKrishnapriya VengavasiZhenglin YangKrishnarjuna BankalaZhengrong GuanKrishnaswamy Gopalan TirumurugaanZhengsheng ZhangKrishnendu PalZhengtao XiaoKrista M. HeinonenZhengwei HuangKristen BradyZhengwen AnKristen E. FunkZhengxiang HuangKristen VarneyZhengxin CaiKristiaan J. LenosZhengxiong XiKristian HaanesZhenhong HeKristian LeisegangZhenhua ShaoKristin L. Eckel-MahanZhenhui ZhongKristin SchubertZhenlin LiKristina A. SharloZhenshan WangKristina EndresZhenzhen JiaKristina GervinZhenzhen ShiKristina GrabušićZhenzhen ZhaoKristina KopevaZheqi LiKristina LekavičienėZhexenbek ToktarbayKristina NesporovaZhi LiKristina PavicZhi YangKristina PerošZhi YueKristina SepcicZhibin JiKristine KrajnakZhibin LiangKristine M. KrajnakZhichen SunKristóf Endre KárolyZhi-Cheng LiuKristofer AndréassonZhicheng PanKristopher R. GenschmerZhicheng YaoKristy SwiderskiZhide DingKrisztián GáspárZhidong ZhouKrisztián KatonaZhifeng DuKrisztián KvellZhigang LiKrisztina BelaZhigang LyuKrisztina MonoryZhiguo ZhangKrisztina NikovicsZhihai QinKrisztina PohóczkyZhihao ChenKrisztina S. NagyZhihao SunKrunoslav T. StinglZhihong ZhangKruthi SuvarnaZhihui LiuKrutika PatelZhijian SuKrystin KrauelZhijie QiKrystyna OlczykZhijie WangKrystyna Pierzchała-KoziecZhijun PuKrystyna RybkaZhila IzadiKrystyna Skwarlo-SontaZhili PangKrzyminski KarolZhili ZuoKrzysztof Artur BogdanowiczZhimin XuKrzysztof BilminZhimin YinKrzysztof BobrowskiZhiming PanKrzysztof BryniarskiZhipeng CaoKrzysztof BrzezińskiZhipeng TaoKrzysztof ChmielowiecZhiping FengKrzysztof ChwastekZhi-Ping LiuKrzysztof FornalskiZhiqiang HuKrzysztof GomułkaZhiqiang QinKrzysztof GorynskiZhiqiang WuKrzysztof KowalZhiqing LiuKrzysztof KrawczykZhirong LiuKrzysztof KrystaZhisheng ZhangKrzysztof KusZhitao QiKrzysztof NawaraZhi-Ting YeKrzysztof PielichowskiZhiwei WenKrzysztof SitkoZhiwei YangKrzysztof SztankeZhixin WangKrzysztof SzyfterZhixiong LiuKrzyżak EdwardZhiyong ChenKsenija DurgoZhiyong HeKshitiz KshitizZhiyong LeiKuaifei XiaZhiyuan MengKuan Yu HsiehZhong ZhengKuan-Chen ChengZhongbing LuKuang-Hung ChengZhonggang FengKuang-Sheng YehZhonghua LiuKuang-Tai KuoZhonghua SunKuang-Wen TsengZhongping ZhangKuan-Hsing ChenZhong-Ru XieKuan-Ting LinZhongtian LiuKubilay YıldırımZhongwei HuangKuei-Yang HsiaoZhongwei LiuKui Ming ChanZhong-Wei ZhouKui ZhangZhongwu ZhouKuikun YangZhoufei WangKuldeep AttriZhoumeng LinKuldeep MakwanaZhousheng XiaoKulwinder KaurZhoutong SunKumar GanesanZhuangwei ShiKumar KatraguntaZhujin YangKumar MuruganZhuo XiongKumar SomyajitZhuoran MaKumar VikrantZhuoxun WuKumaran NagalingamZhuqiu JinKumi Sato-NaraZia Ul MustafaKumika TomaZibing JinKumiko SaekiZidan YuKun HeZifei QinKun LuZigmantas GudžinskasKun LyuZihang LiuKun Song



